# An innovative approach to development of new pyrazolylquinolin-2-one hybrids as dual EGFR and BRAF^V600E^ inhibitors

**DOI:** 10.1007/s11030-025-11127-4

**Published:** 2025-03-08

**Authors:** Mohamed M. Hawwas, Ahmed S. Mancy, Mohamed Ramadan, Tarek S. Ibrahim, Ashraf H. Bayoumi, Mohamed Alswah

**Affiliations:** 1 Department of Pharmaceutical Organic Chemistry, Faculty of Pharmacy, Al-Azhar University, Assiut, Egypt; 2 Department of Pharmacology and Experimental Neuroscience, University of Nebraska Medical Center, Omaha, NE 68198 USA; 3 Department of Pharmaceutical Sciences, College of Pharmacy, University of Nebraska Medical Center, Omaha, Nebraska USA; 4 Department of Pharmaceutical Chemistry, Faculty of Pharmacy, King Abdulaziz University, Jeddah, 21589 Saudi Arabia; 5 Department of Pharmaceutical Organic Chemistry, Faculty of Pharmacy, Zagazig University, Zagazig, 44519 Egypt; 6 Department of Pharmaceutical Organic Chemistry, Faculty of Pharmacy, Al-Azhar University, Cairo, Egypt

**Keywords:** Quinoline, Pyrazole, Antiproliferative, EGFR, BRAF^V600E^

## Abstract

Novel quinoline-based derivatives **2a–e** and **4a–j** have been designed and synthesized as potential antiproliferative agents. The designed compounds were screened for their antiproliferative activity against sixty cell lines according to NCI protocol. The promising hybrids **4d**–**g** are screened by MTT assays on three cancer cell lines: leukemia (MOLT-4), lung cancer (HOP-92), and breast cancer (T47D), with IC_50_ values ranging from 4.982 ± 0.2 to 36.52 ± 1.46 µM compared to Staurosporine, with compound **4e** being the most effective. Derivatives **4d–g** were evaluated for their inhibitory activity on EGFR and BRAF^V600E^. Compound **4e** exhibited the highest inhibitory activities, with IC_50_ values of 0.055 ± 0.002 μM for EGFR and 0.068 ± 0.003 μM for BRAF^V600E^, compared to the reference drugs erlotinib (IC_50_ 0.06 ± 0.002 μM) and vemurafenib (IC_50_ 0.035 ± 0.001 μM), respectively. Cell cycle analysis of the HOP-92 manifested that pre-G1 apoptosis signaling took place after **4e** treatment. Docking simulations were employed to analyze the modes and scores of compounds **4d–g** with respect to EGFR and BRAF^V600E^. The results revealed that compound **4e** exhibited strong affinity for both EGFR and BRAF^V600E^ compared to the reference drugs with values of − 3.226 and − 3.474 kcal/mol, respectively.

**Supplementary Information:**

The online version contains supplementary material available at 10.1007/s11030-025-11127-4.

## Introduction

**Fig. 1:**
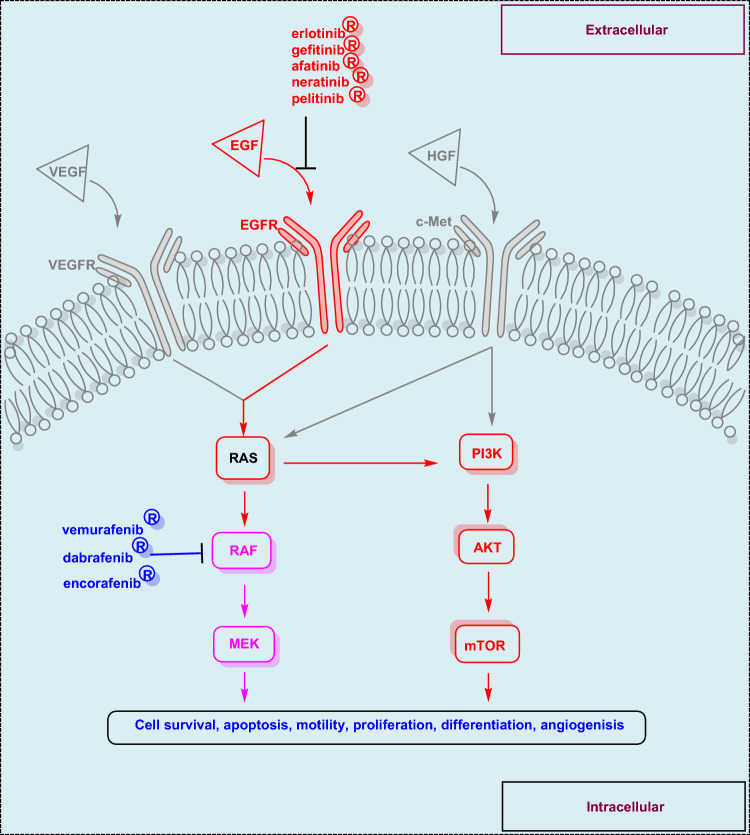
Cell regulation due to EGFR, VEGFR, and c-Met downstream signals through RAS/RAF/MEK pathways

Based on the revised projections from the International Agency for Research on Cancer (IARC), which breaks out worldwide cancer statistics by globe region for 2022, there were about 20 million new cases of cancer and 9.7 million cancer-related deaths [1]. Unfortunately, according to statistics, one in five men and women will have cancer at some point in their lives [2]. Biochemically, protein kinases are a family of enzymes that control a range of cellular biological processes, including apoptosis, proliferation, migration, metabolism, and finally normal cell growth and division [3]. Protein kinases regulate the previous biological process through interaction with its cell membrane-localized receptors, such as EGFR, VEGFR, c-Met, etc. [4]. Growth factors activate the EGFR, triggering a cascade of downstream waves within the cell that activate the RAS/RAF/MEK pathway. Consequently, the parallel pathways of PI3K/AKT/mTOR are stimulated by the activated intracellular RAS (Fig. 1) [5]. Overexpression, mutation, or disintegration of this tangled process results in uncontrolled cell division and proliferation [6]. Regarding cancer treatment strategy, blocking EGFR leads to RAS/RAF/MEK and PI3K/AKT/mTOR signaling cascade suppression [7]. In addition, BRAF^V600E^ is a significant intracellular protein that has recently been extensively studied as a distinct oncogenic element [8]. Unfavorable mutations in the BRAF^V600E^ gene accelerate tumor growth, leading to uncontrolled cell proliferation [7]. The known BRAF^V600E^ mutation was anticipated to serve as a resistance mechanism to much chemotherapy [9]. The development of resistance in cancer has also been linked to the feedback stimulation of EGFR signaling [10]. Hence, EGFR/BRAF (cetuximab/vemurafenib) was used to alleviate these issues according to studies on metastatic cases with BRAF^V600E^ mutations [11]. Therefore, the EGFR activation problem might be resolved by sequentially inhibiting the two kinases. One tactic for coincident blocking of more than one target is combined chemotherapy [12]. Unfortunately, dangerous drug interactions and toxicity can occur while taking several drugs concomitantly [13]. Therefore, combining two medications into a single molecule that hits several targets may help with these problems [14]. Based on the above-mentioned importance of EGFR and BRAF^V600E^ in controlling cell division and proliferation, dual targeting of EGFR and BRAF^V600E^ kinases revealed a successful strategy to control cancers [15]. The low efficacy, resistance, or toxicity related to many single-target or combination-based medicines were overcome by these dual mechanistic medications [16], which also had a more acceptable posology. Subsequently, investigating this dual mechanism could result in a treatment that works well and has fewer adverse effects.

**Fig. 2:**
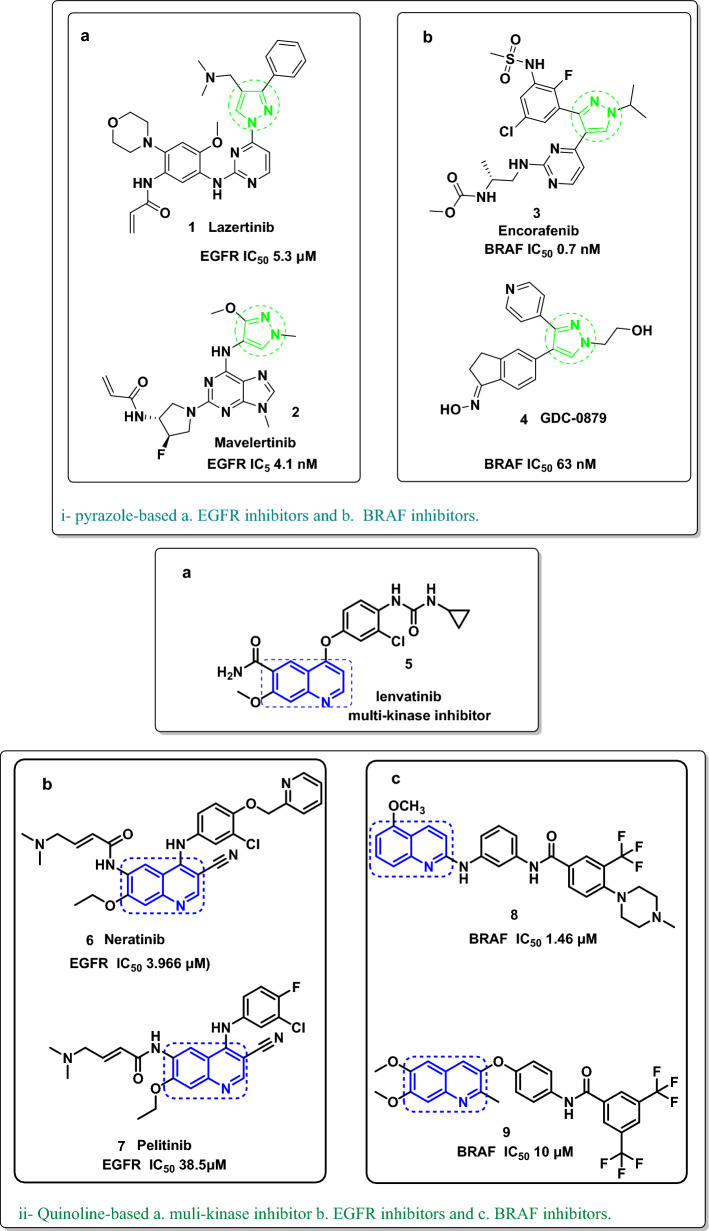

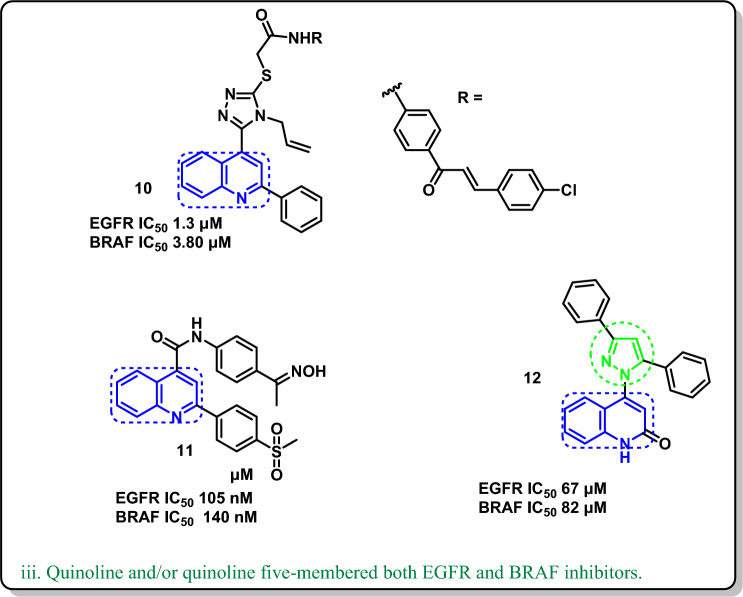
Quinoline/or quinoline five-membered ring EGFR/BRAF single and/or dual mechanism

In this context, pyrazole derivatives (Lazertinib) **1** [17] (Mavelertinib) **2** [18] acted on EGFR as a single target (Fig. 2i-a); other pyrazole derivatives **3** (Encotafenib) [19] (GDC-9879) **4** [20] have the potential to inhibit BRAF^V600E^ (Fig. 2i-b). On the other side, quinoline in general is a wellknown as multi-kinase inhibitory scaffold such as in lenvatinib **5** which works by inhibiting several tyrosine kinase receptors, including VEGFR-1,2,3, FGFR-1,2,3,4, RET, and c-KIT [21]. Notably, the quinoline-bearing compounds (Neratinib) **6** [22] (Pelitinib) **7** [23] were published as EGFR inhibitors (Fig. 2ii-a). Moreover, the quinoline-derived **8** and **9** [24] revealed BRAF^V600E^ inhibitory activities (Fig. 2ii-b). In addition, the quinoline/triazole hybrid **10** [25] and the quinoline/amide-bridged **11** [26] showed promising dual inhibition of both the EGFR and BRAF^V600E^ oncogenic proteins. Further, as a possible twin inhibitory mechanism, the quinoline/pyrazole hybrid **12** [26] worked on both EGFR and BRAF^V600E^ targets (Fig. 2iii). Therefore, searching for a hybrid of quinoline and pyrazole may be the shortest way to obtain a dual-mechanism anticancer.

**Fig. 3:**
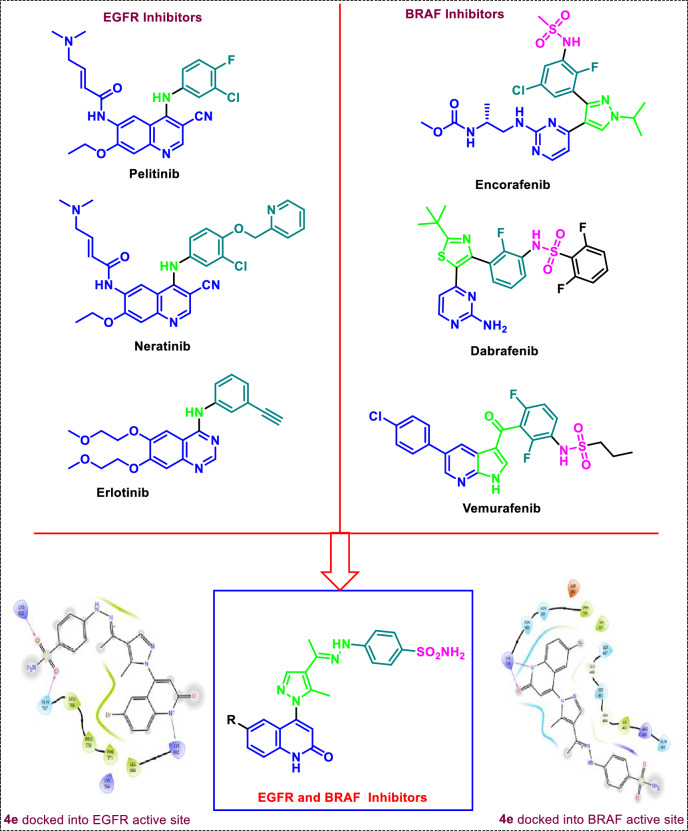
Rationale design for the final compounds **4a–j** inspired from the reported EGFR and BRAF^V600E^-active-marketed anticancer drugs

Considering the Structure–activity relationship of the EGFR-TKIs [27] (Fig. 3); pelitinib, erlotinib [28], and neratinib could be summarized as follows: (a) the adenine pocket is occupied by a heteroaromatic scaffold; (b) a NH spacer that interacts with the amino acids inside the linker region; (c) a terminal hydrophobic moiety; and (d) a hydrophobic tail at the end that settles into the hydrophobic area. On the other hand, the requirements for BRAF^V600E^ inhibitors such as Encorafenib, Dabrafenib [29], and Vemurafenib [30] could be encapsulated in (a) a sulfonamide moiety or alkylated sulfonamide moiety which is essential for interacting with the important amino acid residue; (b) a phenyl moiety occupies the extended side of the pocket; (c) C=O, pyrazole, or imidazole linker; and (d) heteroaromatic system like azaindole or pyrimidine (bioisosteric quinoline as represented in our target compounds) which occupied the adenine pocket. Consequently, considering the essential requirements for EGFR and BRAF^V600E^ inhibitors and utilizing the bio versatility of both quinoline and pyrazole moieties, we designed a novel quinoline-pyrazole-based derivatives that have the main features and pharmacophores for both EGFR and BRAF^V600E^ inhibitors such as a quinoline heteroaromatic system, modified NH linker, or spacer with hydrazone attached to the pyrazole and phenyl sulfonamide moiety.

## Results and discussion

### Chemistry

The intermediates 4-hydrazineylquinolin-2(1*H*)-one derivatives **1a–e** were synthesized according to the reported method [31].

**Scheme 1:**
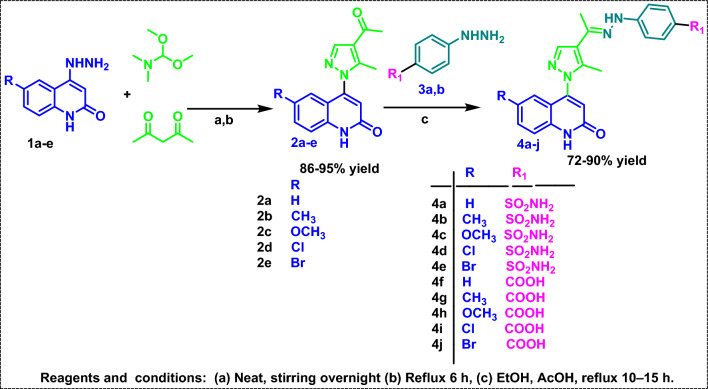
Synthetic approach to target compounds **4a–j**

**Fig. 4:**
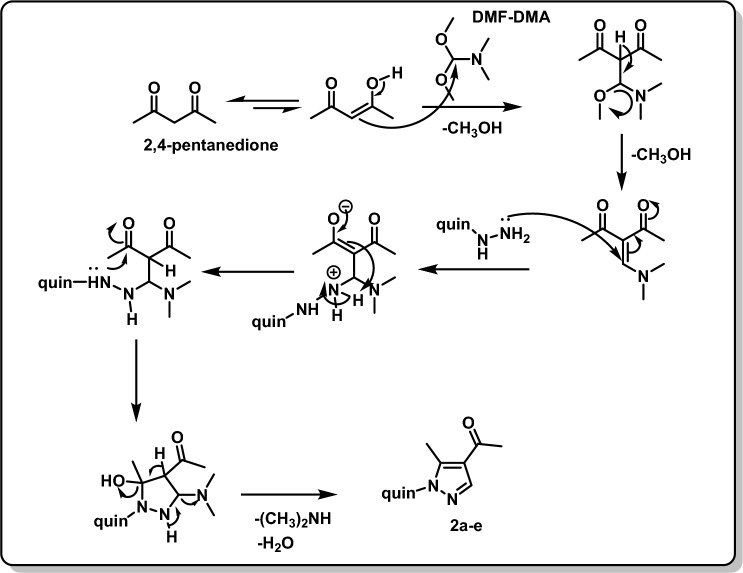
The proposed Michael-addition mechanism for the synthesis of compounds **2a–e**

The final compounds have been synthesized using the procedures outlined in Scheme 1. An equimolar amount of 2,4-pentanedione and *N,N*-dimethylformamidedimethyl acetal (DMF-DMA) was stirred overnight. Then, 4-hydrazineylquinolin-2(1*H*)-ones **1a–e** were added to form intermediates **2a–e** according to the proposed Michael-addition mechanism (Fig. 4) [32]. The final compounds **4a–j** were obtained by condensing acetyl pyrazolyl quinoline derivatives **2a–e** with phenyl hydrazine derivatives **3a-b**.

Along with the expected compounds, confirmatory ^1^H NMR, ^13^C NMR, and HRMS analyses were confirmed. Compound **4e**’s ^1^H NMR revealed a set of twelve protons between 9.61 and 6.78 δ ppm that were related to aromatic and sulfonamide protons, two methyl groups in the aliphatic region, and one proton at 12.29 δ ppm that corresponded to the NH amidic. In addition to the precise number of carbons, ^13^C NMR revealed two peaks in the aliphatic region that were ascribed to methyl carbons. Furthermore, compound **4e** showed molecular ion peaks at m/z 515.0499, which corresponds to its molecular formula conforming its purity.

### Biology

#### National Cancer Institute (NCI) screening

Ten final compounds **4a–j** and five intermediates **2a–e** have been selected by the National Cancer Institute (NCI) for anticancer screening over a panel of 60 cancer cells that cover a variety of histological tissues, including the central nervous system, leukemia, lung, colon, kidney, ovary, breast, and prostate, using the protocol established by the National Cancer Institute (NCI) [33]. In a primary screening, each compound was screened at a concentration of 10 µM. The results were displayed in the supplementary data and expressed as the mean GI% of the treated cells relative to the control cells [34]. The following heatmap (Table 1) highlighted that compounds **4d–g** have promising activity on HOP-92 lung, MOLT-4 leukemia, and T-47D breast cell lines. Interestingly, compound **4e** showed remarkable activity over the most NCI cancer cell lines.

**Table 1:**
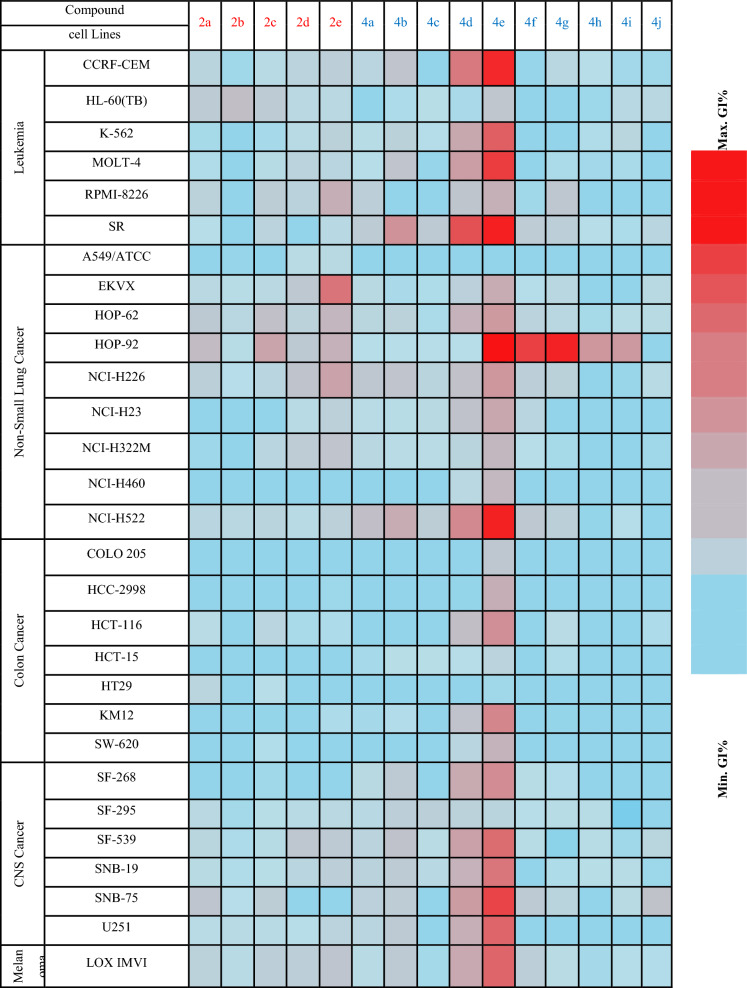

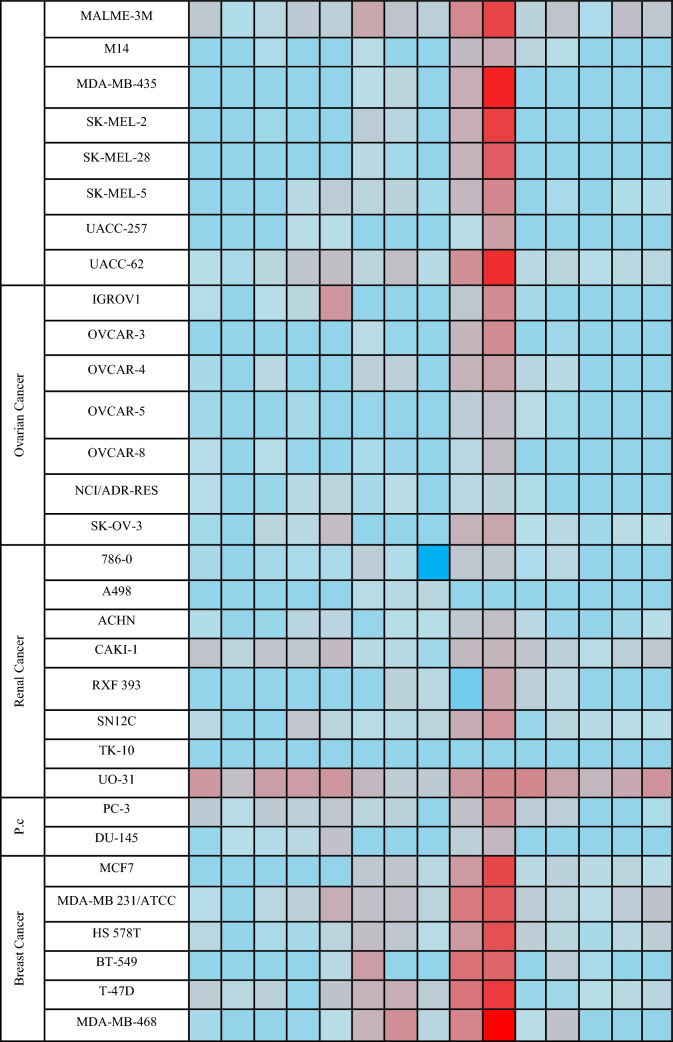
Heatmap diagram of NCI-60 inhibition by compounds 2a–e and 4a–j at 10 µM

*p.c. prostate cancer

#### Structure activity relationship (SAR)

**Fig. 5:**
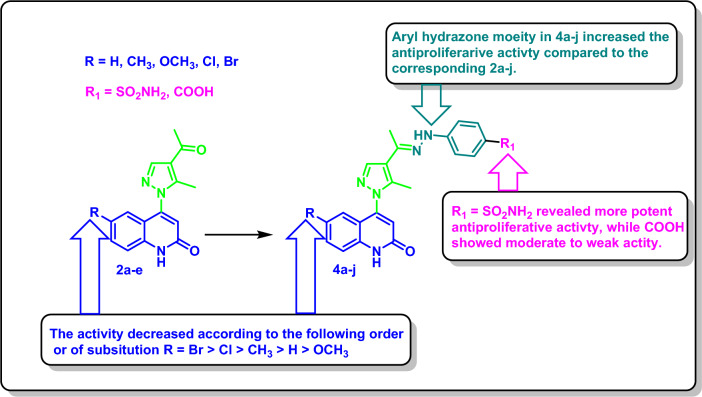
Structure activity relationship (SAR) of **2a–e**, and **4a–j**

The data on in vitro NCI screening findings (Table 1) show above points to the following main trend: (a) regarding to the electron-donating groups, quinolin-2-one’s activity was enhanced by halogen substitution on **C-6**. (b) The antiproliferative effect was more pronounced in the substituted phenyl hydrazone **4a–j** than in the acetylated intermediates **2a–j**. (c) Sulfonamide-substituted compounds **4a–e** are more active compared to the carboxylated compounds **4f–j** (Fig. 5).

#### Antiproliferative assay

**Fig. 6:**
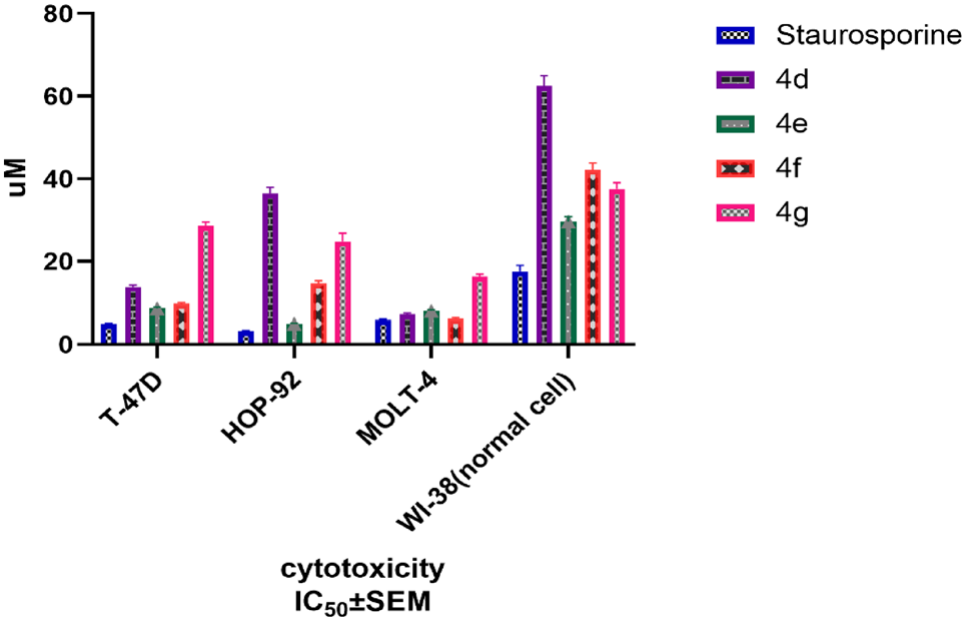
In vitro cytotoxicity of compounds **4d–g** on MOLT-4, HOP-92, and T47D cancer cell lines and cytotoxicity on the normal WI-38 cell line

To a greater extent, the MTT assay [35] was employed to assess the antiproliferative effect of the previously screened promising hybrids **4d–g**. The MTT assay results reassured the promising antiproliferative activity of compound **4e** on the three human cancer cell lines: leukemia (MOLT-4), lung cancer (HOP-92), and breast cancer (T47D) cell lines with mean inhibitory concentration IC_50_ ± SEM 8.62 ± 0.34, 4.982 ± 0.2, and 8.023 ± 0.31 µM, respectively, in comparison to the Staurosporine reference [36] (a broad-spectrum protein kinase inhibitor), which has IC_50_ ± SEM µM 4.94 ± 0.19, 3.172 ± 0.19, and 5.856 ± 0.23, respectively, on the same cell lines. Moreover, compound **4e** did not reveal significant cytotoxicity to the normal cell line WI-38, with IC_50_ ± SEM µM 29.62 ± 1.18 compared to the reference compound with IC_50_ ± SEM µM 17.54 ± 1.5 (Table 2, Fig. 6).

**Table 2:** In vitro cytotoxicity of compounds **4d–g** on MOLT-4, HOP-92, and T47D cancer cell lines and cytotoxicity on the normal WI-38 cell line

Compound	Antiproliferative activity IC_50_ ± SEM (µM)			
	T-47D	HOP-92	MOLT-4	W1-38
**4d**	13.85 ± 0.48	36.52 ± 1.46	7.24 ± 0.29	62.46 ± 2.49
**4e**	8.62 ± 0.34	4.982 ± 0.2	8.023 ± 0.31	29.62 ± 1.18
**4f**	28.56 ± 0.99	24.93 ± 1.9	16.31 ± 0.65	42.14 ± 1.68
**4g**	9.71 ± 0.34	14.72 ± 0.66	6.17 ± 0.25	37.57 ± 1.5
Staurosporine	4.94 ± 0.19	3.172 ± 0.19	5.856 ± 0.23	17.54 ± 1.5

#### EGFR inhibitory activity

**Fig. 7:**
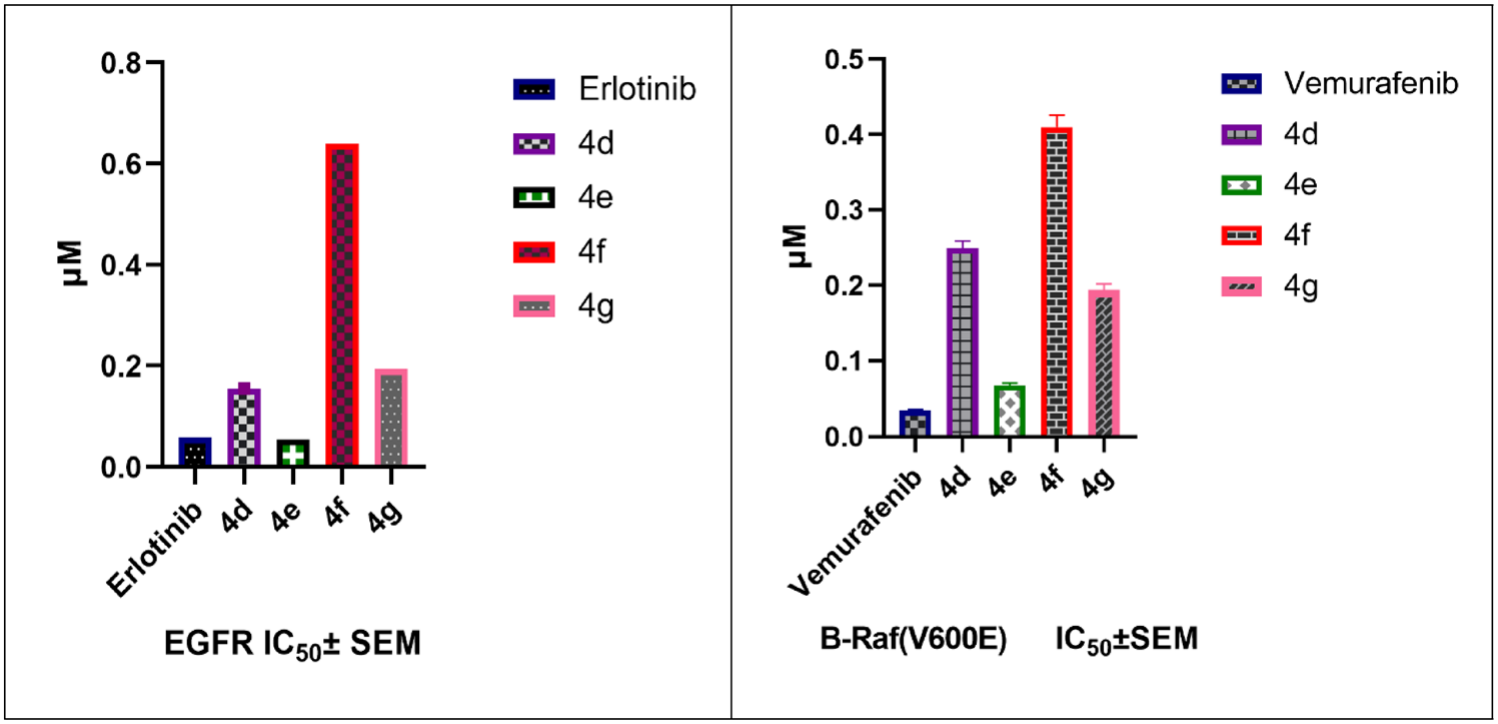
The IC_50_ values of compounds **4d–g** against EGFR and BRAF^V600E^

The inhibitory effect of the most potent antiproliferative derivatives **4d–g** was examined against EGFR as a potential molecular target. The compounds’ IC_50_ values are shown in Table 3. The antiproliferative assay and this inhibitory assay produced identical results. As before, the most effective antiproliferative agent **4e** (R = Br, R^1^ = SO_2_NH_2_) had an IC_50_ value of 0.055 ± 0.002 μM, which was much higher than the reference erlotinib IC_50_ of 0.06 ± 0.002 μM. According to these results, **4e** may be a potential EGFR inhibitor with antiproliferative activity, which supports our previous theory on the mechanism of action of **4e** (Table 3, Fig. 7). See Appendix B.

**Table 3:** The IC_50_ values of compounds **4d–g** against EGFR and BRAF^V600E^

Compound	EGFR inhibition	BRAF^V600E^ inhibition
	IC_50_ ± SEM (μM)	IC_50_ ± SEM (μM)
**4d**	0.156 ± 0.006	0.249 ± 0.01
**4e**	0.055 ± 0.002	0.068 ± 0.003
**4f**	0.64 ± 0.023	0.410 ± 0.016
**4g**	0.194 ± 0.007	0.194 ± 0.008
Erlotinib	0.06 ± 0.002	ND
Vemurafenib	ND	0.035 ± 0.001

N.B: (ND) = Not Determined

#### BRAF^V600E^ inhibitory activity

Using Vemurafenib as the control medication, Compounds **4d–g** were further assessed for their inhibitory activity against BRAF^V600E^. Compounds **4d–g** showed moderate anti-BRAF^V600E^ activity (IC_50_ values ranging from 0.194 ± 0.008 to 0.410 ± 0.016 μM) compared to Vemurafenib (IC_50_ = 0.035 ± 0.001 μM). On the other hand, compound **4e**, with an IC_50_ of 0.068 ± 0.003 μM, was the most potent inhibitor of BRAF^V600E^. These results imply that compound **4e** may act with dual inhibition for EGFR and BRAF^V600E^ as an antiproliferative agent (Table 3, Fig. 7). See Appendix B.

#### Cell cycle analysis

**Fig. 8:**
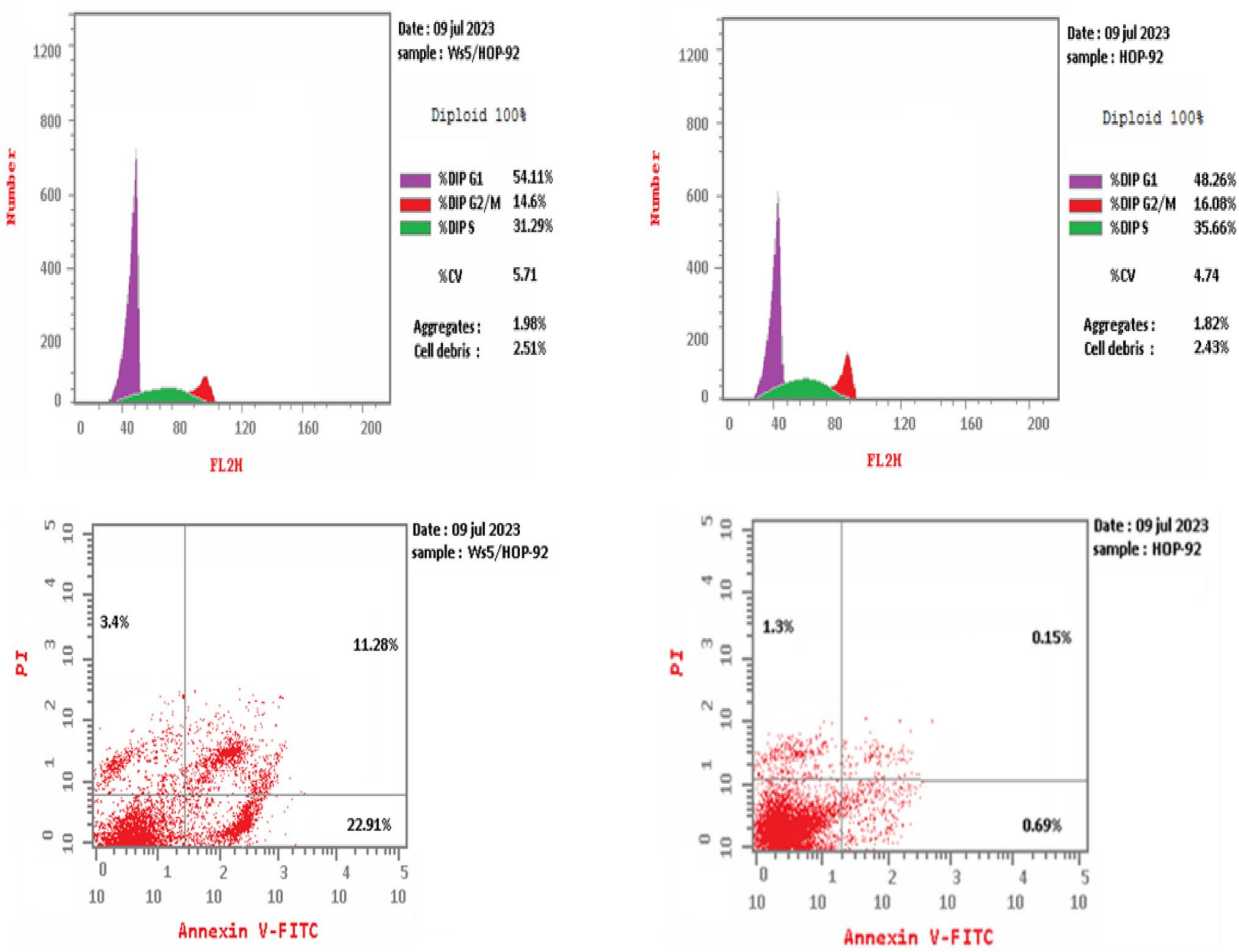
Cell cycle analysis and apoptosis detection of **4e** in HOP-92 cancer cell line

Research has been done on how the most powerful antitumor compound, **4e**, affects the HOP-92 cell cycle’s growth and apoptosis. HOP-92 cells were treated for 24 h at an IC_50_ value of **4e** (4.982 μM). The results of the study (Table 4, Fig. 8) reveal that a higher apoptosis rate at the pre-G1 phase for compound **4e** on HOP-92 with a percent of cell accumulation of 54.11% indicates that cell growth is arrested at the G1 phase. In S and G2/M phases, an unnoticeable change percent of cell accumulation was observed in HOP-92 treated with **4e** (31.3%) and (14.6%), respectively (Table 4).

**Table 4:** Cell cycle analysis of compound **4e** in the HOP-92 cancer cell line

		DNA content			Comment
	IC_50_ µM	% G0–G1	% S	% G2/M	
4e/HOP-92	4.98	54.11	31.3	14.6	Cell growth arrest at G1phase
Cont. HOP-92	–	48.26	35.7	16.08

#### Apoptosis assay

**Fig. 9:**
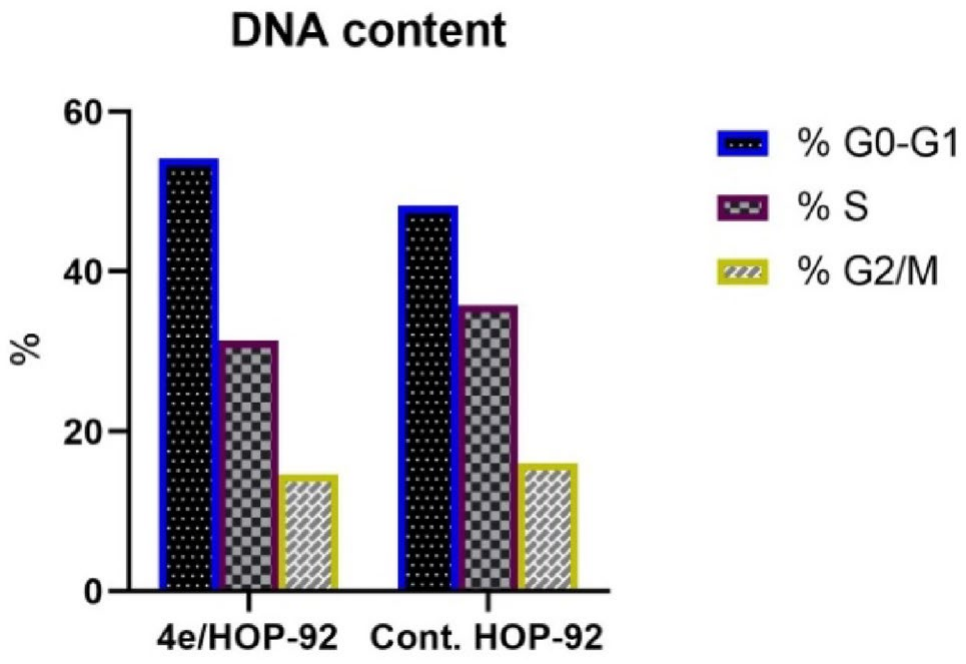
DNA content of **4e** in the HOP-92 cancer cell line

**Fig. 10:**
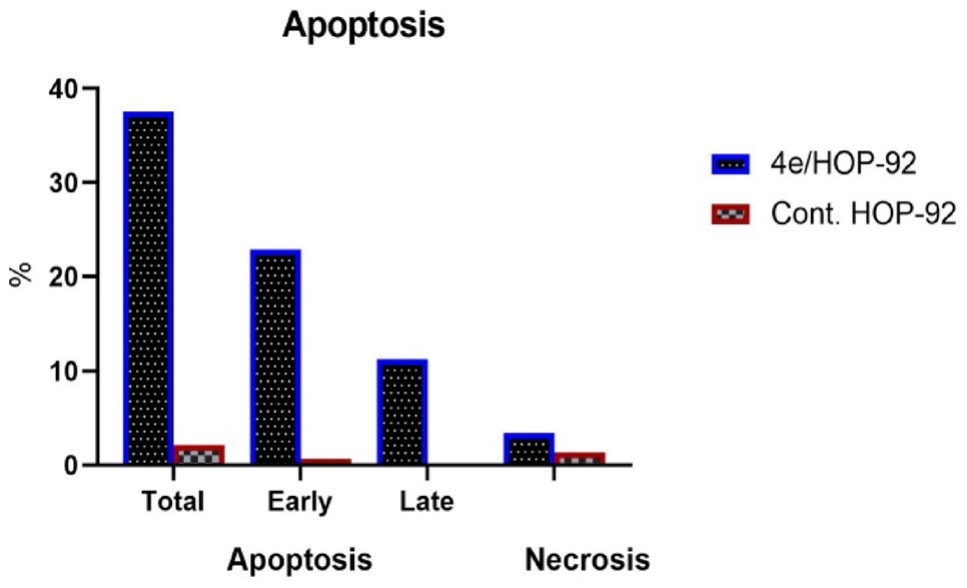
Apoptosis detection of **4e** in the HOP-92 cancer cell line

Using the Annexin V-FITC/PI test [37], the tendency of compound **4e** to cause apoptosis and its potential association with cytotoxic action were evaluated. The cells were labeled with annexin V-FITC/PI and cultured for 24 h to detect any signs of apoptosis. The HOP-92 cell cycle analysis revealed that **4e** treatment was followed by pre-G1 apoptotic signaling. According to research on early and late apoptosis and DNA content (Figs. 9, 10), **4e** with 3.4% necrosis is probably going to result in a large amount of apoptosis (Table 5 and Fig. 10).

**Table 5:** Apoptosis detection of **4e** in the HOP-92 cancer cell line

	Conc	Apoptosis			Necrosis
	IC_50_ μM	Total	Early	Late	
4e/HOP-92	4.982	37.59	22.91	11.28	3.4
Cont. HOP-92	–	2.14	0.69	0.15	1.3

#### Apoptotic indicators

**Fig. 11:**
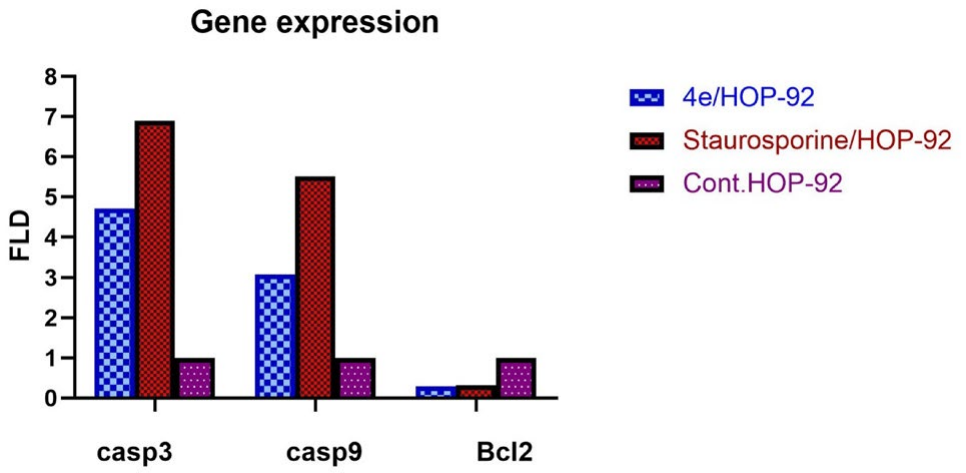
Effect of **4e** on Bcl-2, Caspases-3, and Caspases-9 levels inside the HOP-92 cells

In the depicted findings, compound **4e** affected Bcl-2, caspase-3, and caspase-9, increasing the expression of caspase-3/9 and decreasing the expression of Bcl-2, confirming the apoptotic status [27]. Compound **4e** was found to increase the levels of several mitochondrial apoptotic proteins, such as the main executioner protease (caspases-3/-9) and anti-apoptosis marker (Bcl-2). The results displayed in Fig. 11 showed that the levels of caspases-3 and -9 in HOP-92 cells treated with **4e** were greater than those in the untreated control group by 4.716 and 3.082-fold, respectively (Table 6, Fig. 11). On the other hand, in comparison to the untreated reference, **4e** administration of HOP-92 successfully decreased expression of the anti-apoptotic Bcl-2 protein to one third.

**Table 6:** Effect of 4e on Bcl-2, Caspases-3, and Caspases-9 levels inside the HOP-92 cells

RT-PCR Fold Change		FLD		
Code	IC_50_	Casp-3	Casp-9	Bcl-2
4e/HOP-92	4.982	4.716	3.082	0.299
Staurosporine/HOP-92	3.172	6.906	5.514	0.326
Cont.HOP-92	–	1	1	1

### In silico studies

Docking analyses of the most stable poses of quinolone-based hybrids **4d–g** along with the selected reference ligands erlotinib and vemurafenib were accomplished against EGFR and BRAF^V600E^-active sites.

#### Molecular docking simulation of the quinolone-based hybrids 4d–g inside the EGFR-active site

**Fig. 12:**
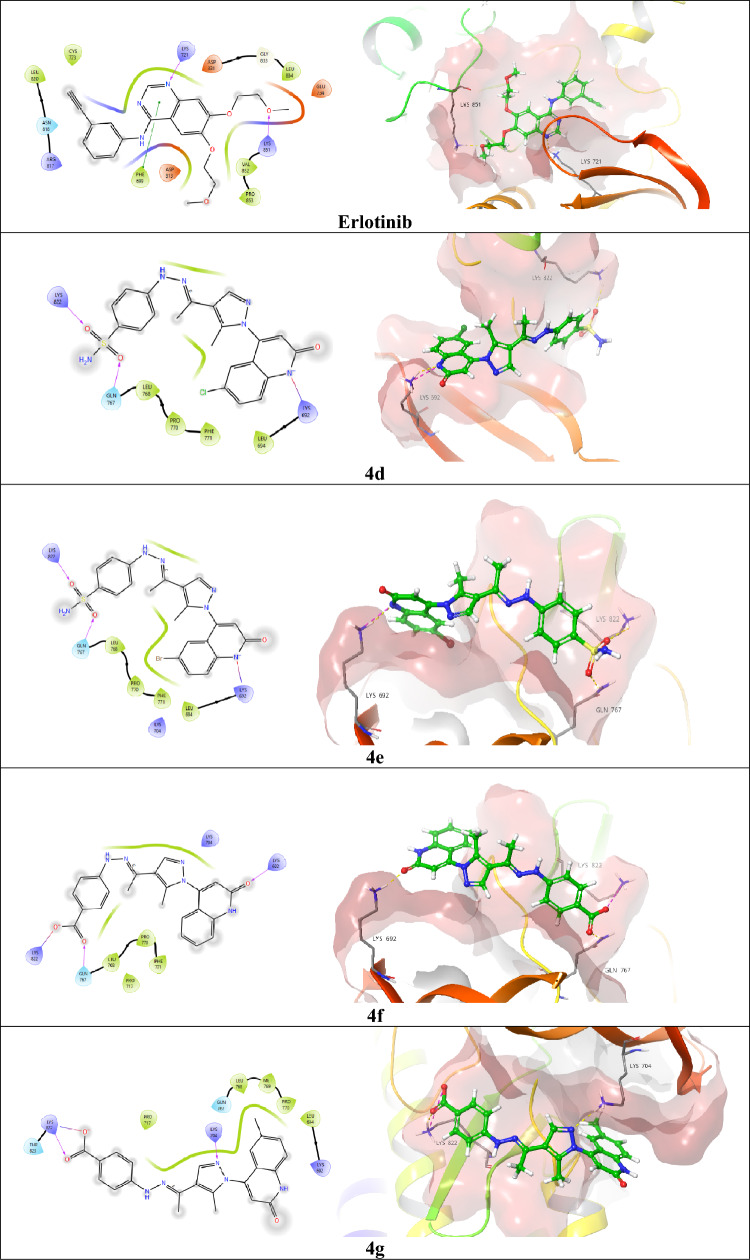
Two-dimensional and three-dimensional (green ball and sticks) surface representation of the novel investigated quinolone-based hybrids **4d–g** besides the reference compound erlotinib in the active site of EGFR

Believing on, the EGFR kinase domain (EGFRK) adopts an NH2-terminal lobe (N-lobe) that is mainly composed of β-strands and one α-helix (αC), and larger COOH terminal lobe (C-lobe) that is typically α-helical. Moreover, researchers have discovered that a cleft, which divides the two lobes, combines ATP, ATP analogs, and ATP-competitive inhibitors [38, 39]. Also considering that, the ATP-binding pocket at the intermediate cleft is divided into three main binding regions: (1) The adenine region, or hinge region, where the adenine free amino group accommodates the H-bond donor effect with the Gln767 residue, and the adenine N1 position establishes H-bond acceptor effect with the neighboring Leu768 and Met769 residues, (2) The sugar region: where the ATP ribose is located and H-bonded by its hydroxyl groups with the Asp86 residue, and (3) the phosphate-binding region: where the triphosphate part of the ATP converges and is H-bonded to the crucial Lys33 and Asp145 amino acids [40, 41]. Consequently, all the reported EGFR inhibitors, such as 4-aminoquinazolines [42], pyrido[2,3-d]pyrimidines [43], and pyrrolo[2,3-*b*]pyridine [44, 45], have been counted as competitive inhibitors at the ATP-binding pocket, especially at the ATP-hinge region. Herein, we accomplished a molecular docking simulation of the reference drug erlotinib as a sort of 4-aminoquinazoline inhibitor [46] and the novel quinolone-based hybrids 4d–g to validate their relative affinity against the EGFR-active site. The reference erlotinib exerted a glide score of (*S* = − 2.485 kcal/mol) that could be attained through worthy H-bonding acceptor interactions of its ether oxygen with the Lys851 amino acid (1.83 A°) and the N1 position of the quinazoline ring with the Lys721 residue (2.31 A°). In addition to a precious pi-pi stacking with the Phe699 amino acid (4.35 A°). Fortunately, all the investigated quinolone-based hybrids 4d–g exhibited superior glide scoring than the reference drug erlotinib, whereas compound 4d (*S* = − 3.483 kcal/mol) utilizes its sulfonamide moiety to reveal two H-bond acceptor effects with Lys822 and the critical Gln767 amino acids, along with a prominent H-bond of the N1 position of the 2-quinolone ring with the key Lys692 (3.10 A°). Providently, comparable with the in vitro assay IC_50_ results, the sulfonamide-bearing compound **4e** that accomplished the EGFR inhibitory effect of (0.055 μM) in Table 7 achieved glide scoring of (3.226 kcal/mol) through performing two H-bond acceptor interactions with the same residues Lys822 and Gln767 that interact with compound 4d. Nevertheless, it accommodates a valuable salt bridge with the crucial residue Lys692 (3.14 A°) (Table 7, Fig. 12). Also, the novel carboxy-bearing candidates 4f (*S* = − 4.407 kcal/mol) and 4g (*S* = − 3.806 kcal/mol) performed obvious salt bridges with the Lys822 residue due to their carboxylate oxygen, while compound 4f elucidated two H-bond acceptor interactions of the same bond length (1.82 A°) with the Lys692 and Gln767 residues due to its 2-quinolone carbonyl and carboxylate carbonyl, respectively.

**Table 7:** Docking scores (kcal/mol) and the key involved residues in the interactions of the investigated compounds **4d, 4e, 4f, 4g**, and the reference drug erlotinib, accompanied with their bond length values (A^○^) inside the EGFR-active site (PDB ID: 1M17)

Compound	Glide score (kcal/ mol)	H-bond	H-bond length (A^○^)	Salt bridge	Salt bridge length (A^○^)	Pi-pi stacking	Pi-pi stacking length (A^○^)
**4d**	− 3.483	Lys692 (1)	3.10	–	–	–	–
		Gln767 (1)	2.02				
		Lys822 (1)	2.47				
**4e**	− 3.226	Gln767 (1)	2.06	Lys692 (1)	3.14	–	–
		Lys822 (1)	2.57				
**4f**	− 4.407	Lys692 (1)	1.82	Lys822 (1)	4.26	–	–
		Gln767 (1)	1.82				
**4g**	− 3.806	Lys704 (1)	2.37	Lys822 (1)	4.62	–	–
		Lys822 (1)	1.66				
Erlotinib	− 2.485	Lys851 (1)	1.83	–	–	Phe699 (1)	4.35
		Lys721 (1)	2.31

Meanwhile, compound 4g also clarified also two H-bond acceptor interactions with Lys704 (2.73 A°) and Lys822 (1.66 A°) due to the N2 of its pyrazole core and the carbonyl oxygen of its carboxylate, respectively (Table 7, Fig. 12). Wherefore, compound **4e**-bearing pyrazole could be considered a promising EGFR inhibitor through ATP-competitive inhibition at the hinge region while it accommodates valuable contacts with Gln767 where the adenine part of ATP binds, and gaining a superior computational docking score and in vitro IC_50_ results over the reference erlotinib that requires further study.

#### Molecular docking simulation of the quinolone-based hybrids 4d–g inside the BRAF^V600E^-active site

**Fig. 13:**
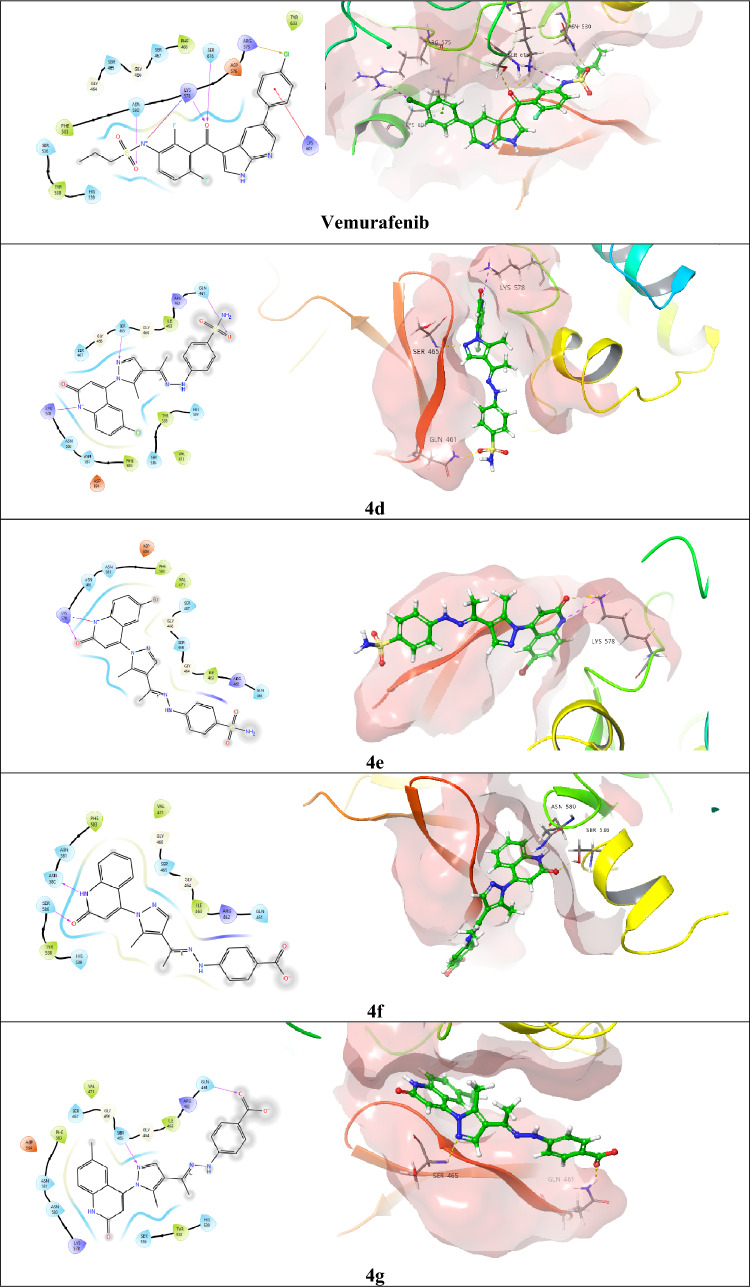
Two-dimensional and three-dimensional (green ball and sticks) surface representation of the novel investigated quinolone-based hybrids **4d–g** besides the reference compound vemurafenib in the active site of BRAF^V600E^

As the BRAF^V600E^, within the mitogen-activated protein kinase (MAPK) signaling pathway, tends to be mutated in a significant number of cancers, particularly in about 50% of melanomas [47]. About 90% of BRAF^V600E^ homo- and heterodimer mutations correlated with cancer contain a distinct mutation of the Val600 residue to Glu600 (BRAF^V600E^), which prompts an active conformation without activation loop phosphorylation [48]. Therefore, approximately all patients promote drug resistance within about 6 months of treatment by BRAF^V600E^ inhibitors that predominantly arise through reactivation of the MAPK pathway that is called transactivation [49]. In the present work, we investigated vemurafenib reference drug [50] besides the quinolone-based hybrids **4d–g** as chemically linked kinase inhibitors aiming to lock the BRAF^V600E^ dimers in an inactive conformation that cannot endure transactivation and, hence, confirm their potential against mutated instances (Table 8, Fig. 13). The reference compound vemurafenib elucidated a useful glide score of (− 3.266 kcal/mol), represented by four protruding H-bonding interactions with the Asn580, Arg575, Ser616, and Lys578 residues with bond lengths of 1.99, 2.33, 2.56, and 2.27 A°, respectively. In addition to a prominent salt bridge of sulfonamide nitrogen with the Lys578 amino acid and *pi*-cation interaction with the Lys601 besides the mutated Val600 residue. Fortunately, all the inspected quinolone-based hybrids **4d–g** revealed better glide scoring than the reference vemurafenib. While compound 4d sulfonamide oxygen created an H-bond acceptor effect with the Gln461 residue (2.13 A°), along with another H-bond acceptor effect of the N_2_ of the pyrazole moiety with the Ser465 residue (2.43 A°), and a characteristic salt bridge of the 2-quinolone nitrogen with the crucial Lys578 residue to achieve a glide score of (− 4.045 kcal/mol). Moreover, the sulfonamide compound **4e** that accomplished the most hopeful IC_50_ result among the investigated compounds (0.068 μM) displayed a glide score of (− 3.474 kcal/mol) through an obvious H-bond acceptor interaction of its 2-quinolone carbonyl oxygen with the key Lys578 and a prominent salt bridge of its 2-quinolone nitrogen with the same residue. Also, the carboxy compounds **4f** and **4g** presented superior glide scoring over the reference vemurafenib of (− 5.717 kcal/mol) and (− 4.815 kcal/mol), respectively. Whereas compound **4f** demonstrated a useful H-bond donor effect of its 2-quinolone nitrogen with the Asn580 residue (2.21 A°), the 2-carbonyl oxygen protruded H-bond acceptor effect with the Ser536 amino acid (2.15 A°). In a diverse manner, compound **4g** elucidated two H-bond acceptor effects due to its carboxylate carbonyl oxygen and N_2_ of the pyrazole core with Gln461 (1.95 A°) and Ser465 (2.53 A°), respectively. Lastly, the inspected quinolone-based hybrids **4d–g** exposed superior binding affinity to the BRAF^V600E^-active site that exceeds the reference drug vemurafenib that may explain their capability to overcome the mutated consequences of the BRAF^V600E^ and treat the resistant malignant cases that require further investigation.

**Table 8:** Docking scores (kcal/mol) and the key involved residues in the interactions of the investigated quinolone-based hybrids **4d–g** and the reference drug vemurafenib, accompanied with their bond length values (A^○^) inside the BRAF^V600E^-active site (PDB ID: 5JRQ)

Compound	Glide score (kcal/ mol)	H-bond	H-bond length (A^○^)	Salt bridge	Salt bridge length (A^○^)	Pi-cation interaction	Pi-cation bond length (A^○^)
**4d**	− 4.045	Ser465 (1)	2.43	Lys578 (1)	3.45	–	–
		Gln461 (1)	2.13				
**4e**	− 3.474	Lys578 (1)	2.66	Lys578 (1)	3.56	–	–
**4f**	− 5.717	Asn580 (1)	2.21	–	–	–	–
		Ser536 (1)	2.15				
**4g**	− 4.815	Ser465 (1)	2.53	–	–	–	–
		Gln461 (1)	461				
Vemurafenib	− 3.266	Asn580 (1)	1.99	Lys578 (1)	4.26	Lys601	6.51
		Arg575 (1)	2.33				
		Ser616 (1)	2.56				
		Lys578 (1)	2.27

#### Pharmacokinetic profile (ADME) and drug-likeness

**Fig. 14:**
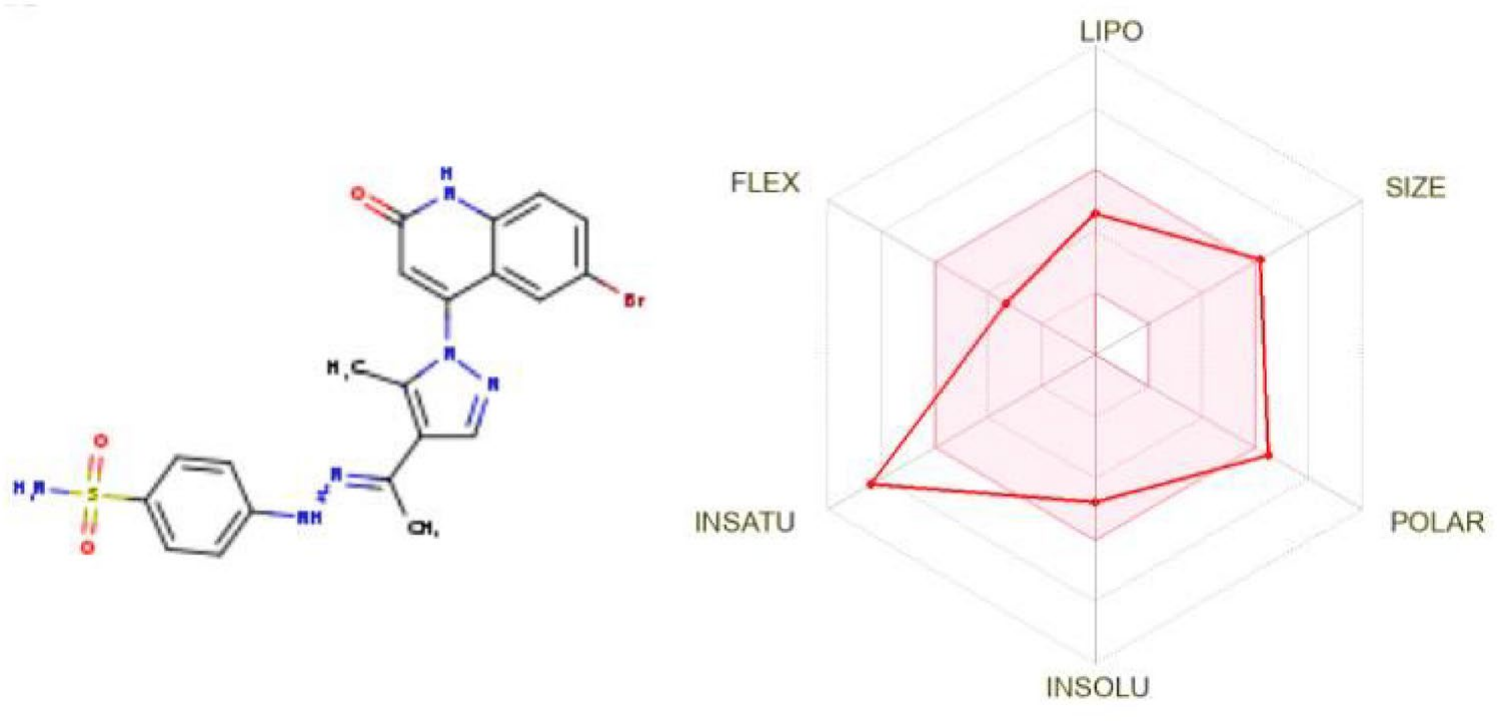
Pharmacokinetic profile (ADME) and drug-likeness of compound **4e**

The online SwissADME tool [51] was used to calculate the expected physicochemical considerations. The obtained results provide a favorable impression of the unique synthesized candidates’ feasibility profiles. It seems that none of the produced compounds violate the Lipinski criteria. Pharmacokinetic analysis revealed that compounds **4d** and **4e** have limited gastrointestinal (GI) absorption, which is regarded as a crucial factor for oral delivery. Compounds **4f** and **4g**, on the other hand, had substantial GI absorption (Table 9, Fig. 14). The Synthetic accessibility (SA) value is crucial when examining medicinal chemistry; the values acquired increase in difficulty from 1 to 10, which they cannot surpass. Thankfully, it was discovered that every produced molecule had values between 3 and 4, with no compound approaching or surpassing 10. In summary, all the compounds have suitable physicochemical parameters to be drug candidates [52].

**Table 9:** Predicted ADME parameters of the novel compounds **4d**–**4g**

Comp.	Physicochemical properties					Pharmacokinetics		Drug likeness		Medicinal chemistry
	HB A^a^	HBD^b^	TPSA^c^	Log Po/w^d^	LogS^e^	GIA	Log Kp^f^	Lipinski	Vio^g^	SA
4d	6	3	143.61	2.66	−4.46	Low	−7.43	Yes	0	3.39
**4e**	6	3	143.61	2.74	−4.73	Low	−7.65	Yes	1	3.41
4f	5	3	112.37	2.72	−4.27	High	−6.77	Yes	0	3.23
4g	5	3	112.37	3.07	−4.57	High	−6.60	Yes	0	3.36

^a^: Number of hydrogen bond acceptors. ^b^: Number of hydrogen bond donors. ^c^: Topological polar surface area. ^d^: Partition coefficient (lipophilicity). ^e^: Water Solubility. ^f^:(skin permeation). ^g^: violation

## Conclusion

A series of new compounds based on quinoline have been designed, synthesized, and tested for their ability to inhibit cell division. A panel of sixty cancer cell lines from the NCI was used to examine the newly produced compounds in vitro. Quinolone-based hybrids **4d–g** showed encouraging antiproliferative activity in the MTT experiment when compared to Staurosporine, the positive control. Compound **4e** was found to be the most effective; it may target EGFR and/or BRAF^V600E^ to function as an antiproliferative agent. Compound **4e** displayed cell cycle arrest at the G0-1 phase and caused apoptosis against BRAF^V600E^ and EGFR, two potential dual targets for anticancer therapy.

Regarding the molecular docking studies, quinolone-based hybrids **4d–g** clearly showed significant docking scores for EGFR and BRAF^V600E^ in relation to the ligands vemurafenib and erlotinib, respectively. Interestingly, compound **4e** may prove to be a promising inhibitor for both the BRAF^V600E^ and EGFR targets following structural alteration.

## Experimental

### Chemistry

General details: See Appendix A

#### General synthetic procedure for 4-(4Aacetyl-5-methyl-1H-pyrazol-1-yl)-6-substituted quinolin-2(1H)-one derivatives. (2a–e)

A mixture of 2,4-Pentanedione (5.0 mmol) was mixed with *N,N*-dimethylformamide-dimethyl acetal (DMF-DMA) (6.0 mmol) and was stirred overnight at room temperature. Next, a 4-hydrazineylquinolin-2(1*H*)-one derivative (5 mmol) was added, and the reaction mixture was heated at 70–80 °C in ethanol for 6 h. The reaction mixture was subsequently allowed to cool and refrigerated for 24 h. Then the reaction mixture was filtrated and washed with ethanol.

##### 4-(4-Acetyl-5-methyl-1H-pyrazol-1-yl)quinolin-2(1H)-one (2a)

Pale yellow powder, Yield: (93%); mp: 219–221 °C, ^1^H NMR (400 MHz, DMSO-*d*_*6*_) δ (ppm): 12.22 (1H s, 1H, NHCO), 8.38 (s, 1H), 7.60 (t, *J* = 7.7 Hz, 1H),7.44 (d, *J* = 8.2 Hz, 1H), 7.17 (t, *J* = 7.6 Hz, 1H), 6.96 (d, *J* = 7.5 Hz, 1H), 6.75 (s, 1H), 2.49 (s, 3H, –CH_3_), 2.43 (s, 3H, –CH_3_). ^13^C NMR (100 MHz, DMSO-*d*_*6*_) δ (ppm): 193.10, 161.29, 145.29, 144.03, 142.94, 139.33, 131.76, 123.99, 122.53, 120.62, 120.54, 116.36, 115.83, 28.85, 11.36. Anal. Calcd. For C_15_H_13_N_3_O_2_: C, 67.40; H, 4.90; N, 15.72. Found: C, 67.62; H, 5.06; N, 15.89.

##### 4-(4-Acetyl-5-methyl-1H-pyrazol-1-yl)-6-methylquinolin-2(1H)-one (2b)

Yield: (88%); mp: 223–225 °C, ^1^H NMR (400 MHz, DMSO-*d*_*6*_) δ (ppm): 12.15 (s, 1H, NHCO), 8.38 (s, 1H), 7.43 (d, *J* = 8.4 Hz, 1H), 7.35 (d, *J* = 8.4 Hz, 1H), 6.76 (s, 1H), 6.70 (s, 1H), 2.49 (s, 3H, –CH_3_), 2.42 (s, 3H, –CH_3_), 2.25 (s, 3H, –CH_3_^13^C NMR (100 MHz, DMSO-*d*_*6*_) δ (ppm): 193.36, 161.56, 145.51, 144.40, 143.42, 137.88, 133.50, 132.13, 123.65, 121.02, 120.94, 116.73, 116.26, 28.94, 20.88, 11.82. Anal. Calcd. For C_16_H_15_N_3_O_2_: C, 68.31; H, 5.37; N, 14.94. Found: C, 68.44; H, 5.49; N, 15.17.

##### 4-(4-Acetyl-5-methyl-1H-pyrazol-1-yl)-6-methoxyquinolin-2(1H)-one (2c)

Yellow powder, Yield: (95%); mp: 211–213 °C, ^1^H NMR (400 MHz, DMSO-*d*_*6*_) δ (ppm): 12.13 (s, 1H, NHCO), 8.39 (s, 1H), 7.40 (d, *J* = 9.0 Hz, 1H), 7.30 (d, *J* = 8.9 Hz, 1H), 6.74 (s, 1H), 6.41 (s, 1H), 3.65 (s, 3H, OCH_3_), 2.49 (s, 3H, –CH_3_), 2.44 (s, 3H, –CH_3_). ^13^C NMR (100 MHz, DMSO-*d*_*6*_) δ (ppm): 193.74, 161.34, 155.01, 145.23, 144.53, 143.50, 134.31, 121.30, 121.11, 121.01, 117.82, 117.46, 106.21, 55.91, 29.28, 11.84. Anal. Calcd. For C_16_H_15_N_3_O_3_: C, 64.64; H, 5.09; N, 14.13. Found: C, 64.80; H, 5.21; N, 14.30.

##### 4-(4Aacetyl-5-methyl-1H-pyrazol-1-yl)-6-chloroquinolin-2(1H)-one (2d)

Dark yellow powder, Yield: (91%); mp: 229–231 °C, ^1^H NMR (400 MHz, DMSO-*d*_*6*_) δ (ppm): 12.34 (s, 1H, NHCO), 8.41 (s, 1H), 7.66 (dd, *J* = 8.8, 2.4 Hz, 1H), 7.45 (d, *J* = 8.8 Hz, 1H), 7.03 (d, *J* = 2.3 Hz, 1H), 6.86 (s, 1H), 2.49 (s, 3H, –CH_3_), 2.47 (s, 3H, –CH_3_). ^13^C NMR (100 MHz, DMSO-*d*_*6*_) δ (ppm): 193.38, 161.17, 144.55, 144.15, 143.43, 138.30, 131.83, 126.62, 123.35, 121.64, 120.82, 118.04, 117.64, 28.89, 11.66. Anal. Calcd. For C_15_H_12_ClN_3_O_2_: C, 59.71; H, 4.01; Cl, 11.75; N, 13.93; Found: C, 59.89; H, 4.23; N, 14.15.

##### 4-(4-Acetyl-5-methyl-1H-pyrazol-1-yl)-6-bromoquinolin-2(1H)-one (2e)

Brown powder, Yield: (86%); mp: 240–242 °C, ^1^H NMR (400 MHz, DMSO-*d*_*6*_) δ (ppm): 12.33 (s, 1H, NHCO), 8.41 (s, 1H), 7.76 (d, *J* = 8.8 Hz, 1H), 7.39 (d, *J* = 8.8 Hz, 1H), 7.16 (s, 1H), 6.84 (s, 1H), 2.49 (s, 3H), 2.47 (s, 3H). ^13^C NMR (100 MHz, DMSO-*d*_*6*_) δ (ppm): 193.53, 161.46, 144.85, 144.30, 143.74, 139.00, 134.74, 126.68, 121.94, 121.13, 118.52, 118.39, 114.24, 29.33, 12.11. Anal. Calcd. For C_15_H_12_BrN_3_O_2_: C, 52.04; H, 3.49. N, 12.14. Found: C, 52.16; H, 3.60; N, 12.37.

#### General synthetic procedure for (E)-4-(2-(1-(1-(6-substituted-2-oxo-1,2-dihydroquinolin-4-yl)-5-methyl-1H-pyrazol-4-yl) ethylidene)hydrazineyl)benzenesulfonamide derivatives. 4a–e and (E)-4-(2-(1-(1-(6-substituted-2-oxo-1,2-dihydroquinolin-4-yl)-5-methyl-1H-pyrazol-4-yl) ethylidene)hydrazineyl)benzoic acid derivatives. 4f–j

A sulfonamide phenylhydrazine derivative **3a** or carboxylic phenylhydrazine derivative **3b** (1.0 mmol) and 3 drops of glacial acetic acid were added to a suspension of compounds **2a–e** (1.0 mmol) in ethanol (20 mL). The reaction mixture was refluxed for 10–12 h. The precipitate that formed after cooling was filtered out, washed with diethyl ether, and crystallized from 20 mL of ethanol.

##### (E)-4-(2-(1-(5-methyl-1-(2-oxo-1,2-dihydroquinolin-4-yl)-1H-pyrazol-4-yl)ethylidene) hydrazineyl)benzenesulfonamide (4a)

Pale yellow powder, yield: (76%); mp: 270–272 °C, ^1^H NMR (400 MHz, DMSO-*d*_*6*_) δ (ppm): 12.17 (s, 1H, NH), 9.59 (s, 1H), 8.09 (s, 1H), 7.68–7.53 (m, 3H), 7.44 (d, *J* = 8.2 Hz, 1H), 7.19 (m, 3H), 7.09–6.98 (m, 3H), 6.69 (s, 1H), 2.49 (s, 3H, –CH_3_), 2.32 (s, 3H, –CH_3_). ^13^C NMR (100 MHz, DMSO-*d*_*6*_) δ (ppm): 161.43, 148.78, 146.05, 140.24, 140.18, 139.32, 137.79, 133.04, 131.55, 127.25, 124.35, 122.37, 120.04, 116.74, 115.75, 111.57, 15.19, 12.59. HRMS (ESI, m/z) calcd for [C_21_H_21_N_6_O_3_S]^+^ (M + H)^+^ 437.1318, found. 437.1392.

##### (E)-4-(2-(1-(5-methyl-1-(6-methyl-2-oxo-1,2-dihydroquinolin-4-yl)-1H-pyrazol-4-yl) ethylidene)hydrazineyl)benzenesulfonamide (4b)

White powder, yield: (87%); mp: 289–291 °C, ^1^H NMR (400 MHz, DMSO-*d*_*6*_) δ (ppm): 12.11 (s, 1H, NHCO), 9.59 (s, 1H), 8.10 (s, 1H), 7.64 (d, *J* = 8.8 Hz, 2H), 7.40 (dd, *J* = 30.7, 8.5 Hz, 2H), 7.22 (d, *J* = 8.8 Hz, 2H), 7.03 (s, 2H), 6.86 (s, 1H), 6.65 (s, 1H), 2.50 (s, 3H), 2.33 (s, 3H), 2.27 (s, 3H). ^13^C NMR (100 MHz, DMSO-*d*_*6*_) δ (ppm): 161.06, 148.79, 145.82, 140.24, 137.71, 137.43, 133.03, 132.86, 131.46, 127.25, 123.58, 120.02, 119.95, 116.69, 115.74, 111.57, 20.41, 15.18, 12.61. HRMS (ESI, m/z) calcd for [C_22_H_23_N_6_O_3_S]^+^ (M + H)^+^ 451.1474, found. 451.1551.

##### (E)-4-(2-(1-(1-(6-methoxy-2-oxo-1,2-dihydroquinolin-4-yl)-5-methyl-1H-pyrazol-4-yl) ethylidene)hydrazineyl)benzenesulfonamide (4c)

White powder, yield: (77%); mp: 273–275 °C, ^1^H NMR (400 MHz, DMSO-*d*_*6*_) δ (ppm): 12.07 (s, 1H, NHCO), 9.58 (s, 1H), 8.10 (s, 1H), 7.63 (d, *J* = 8.1 Hz, 2H), 7.41–7.17 (m, 4H), 7.02 (s, 2H), 6.67 (s, 1H), 6.53 (s, 1H), 3.65 (s, 3H, –CH_3_), 2.49 (d, *J* = 5.0 Hz, 3H, –CH_3_), 2.31 (s, 3H –CH_3_). ^13^C NMR (100 MHz, DMSO-*d*_*6*_) δ (ppm): 161.42, 154.86, 149.25, 145.92, 140.76, 140.67, 138.25, 134.42, 133.51, 127.73, 120.84, 120.53, 117.83, 117.68, 112.05, 106.67, 55.89, 15.66, 13.13. HRMS (ESI, m/z) calcd for [C_22_H_231_N_6_O_4_S]^+^ (M + H)^+^ 467.1423, found. 467.1497.

##### (E)-4-(2-(1-(1-(6-chloro-2-oxo-1,2-dihydroquinolin-4-yl)-5-methyl-1H-pyrazol-4-yl) ethylidene)hydrazineyl)benzenesulfonamide(4d)

Pale yellow powder, yield: (74%); mp: 290–292 °C, ^1^H NMR (400 MHz, DMSO-*d*_*6*_) δ (ppm): 12.30 (s, 1H, NHCO), 9.60 (s, 1H), 8.13 (s, 1H), 7.65 (t, *J* = 7.9 Hz, 3H), 7.46 (d, *J* = 8.8 Hz, 1H), 7.22 (d, *J* = 8.8 Hz, 2H), 7.12 (s, 1H), 7.03 (s, 2H), 6.79 (s, 1H), 2.53 (s, 3H –CH_3_), 2.32 (s, 3H –CH_3_). ^13^C NMR (100 MHz, DMSO-*d*_*6*_) δ (ppm): 161.18, 148.75, 144.64, 140.60, 140.03, 138.22, 138.07, 133.08, 131.45, 127.25, 126.22, 123.57, 120.86, 120.29, 117.85, 117.80, 111.58, 15.20, 12.61. HRMS (ESI, m/z) calcd for [C_21_H_20_ClN_6_O_3_S]^+^ (M + H)^+^ 471.0928, found. 471.1002.

##### (E)-4-(2-(1-(1-(6-bromo-2-oxo-1,2-dihydroquinolin-4-yl)-5-methyl-1H-pyrazol-4-yl) ethylidene)hydrazineyl)benzenesulfonamide (4e)

Pale yellow powder, yield: (78%); mp: 293–295 °C, ^1^H NMR (400 MHz, DMSO-*d*_*6*_) δ (ppm): 12.29 (s, 1H, NHCO), 9.61 (s, 1H), 8.14 (s, 1H), 7.76 (dd, *J* = 8.8, 2.1 Hz, 1H), 7.64 (d, *J* = 8.8 Hz, 2H), 7.39 (d, *J* = 8.8 Hz, 1H), 7.24 (dd, *J* = 18.4, 5.5 Hz, 3H), 7.03 (s, 2H), 6.78 (s, 1H), 2.53 (s, 3H), 2.32 (s, 3H). ^13^C NMR (10 MHz, DMSO-*d*_*6*_) δ (ppm): 161.41, 148.91, 144.82, 140.78, 140.22, 138.63, 138.26, 134.30, 133.15, 127.42, 126.69, 120.88, 120.46, 118.46, 118.22, 114.21, 111.76, 15.32, 12.76. HRMS (ESI, m/z) calcd for [C_21_H_20_^79^BrN_6_O_3_S]^+^ (M + H)^+^ 515.0423, found. 515.0499.

##### (E)-4-(2-(1-(5-methyl-1-(2-oxo-1,2-dihydroquinolin-4-yl)-1H-pyrazol-4-yl)ethylidene) hydrazineyl)benzoic acid (4f)

Pale yellow powder, yield: (81%); mp: 268–270 °C, ^1^H NMR (400 MHz, DMSO-*d*_*6*_) δ (ppm): 12.16 (s, 2H, COOH, NHCO), 9.58 (s, 1H), 8.07 (s, 1H), 7.77 (d, *J* = 8.4 Hz, 1H), 7.57 (t, *J* = 7.4 Hz, 1H), 7.42 (d, *J* = 8.1 Hz, 1H), 7.15 (d, *J* = 8.3 Hz, 1H), 7.04 (d, *J* = 7.9 Hz, 1H), 6.68 (s, 1H), 2.47 (s, 1H), 2.30 (s, 1H). ^13^C NMR (100 MHz, DMSO-*d*_*6*_) δ (ppm): 167.39, 161.47, 149.81, 146.09, 140.27, 140.20, 139.32, 137.80, 131.56, 131.02, 124.36, 122.39, 120.08, 120.04, 119.90, 116.75, 115.77, 111.59, 15.26, 12.59. HRMS (ESI, m/z) calcd for [C_22_H_20_N_5_O_3_]^+^ (M + H)^+^ 402.1488, found. 402.1557.

##### (E)-4-(2-(1-(5-methyl-1-(6-methyl-2-oxo-1,2-dihydroquinolin-4-yl)-1H-pyrazol-4-yl) ethylidene)hydrazineyl)benzoic acid (4g)

Pale yellow powder, yield: (88%); mp: 255–257 °C, ^1^H NMR (400 MHz, DMSO-*d*_*6*_) δ (ppm): 12.20 (s, 1H, COOH), 12.10 (s, 1H, NHCO), 9.60 (s, 1H), 8.09 (s, 1H), 7.78 (d, *J* = 8.8 Hz, 2H), 7.42 (dd, *J* = 8.5, 1.7 Hz, 1H), 7.35 (d, *J* = 8.4 Hz, 1H), 7.17 (d, *J* = 8.8 Hz, 2H), 6.86 (s, 1H), 6.64 (s, 1H), 2.49 (s, 3H), 2.32 (s, 3H), 2.26 (s, 3H). ^13^C NMR (100 MHz, DMSO-*d*_*6*_) δ (ppm): 167.82, 161.74, 150.26, 146.29, 140.71, 140.69, 138.17, 137.89, 133.31, 131.92, 131.46, 124.05, 120.49, 120.43, 120.34, 117.15, 116.21, 112.04, 21.04, 15.72, 13.06. HRMS (ESI, m/z) calcd for [C_23_H_22_N_5_O_3_]^+^ (M + H)^+^ 416.1644, found. 416.1717.

##### (E)-4-(2-(1-(1-(6-methoxy-2-oxo-1,2-dihydroquinolin-4-yl)-5-methyl-1H-pyrazol-4-yl) ethylidene)hydrazineyl)benzoic acid (4h)

Pale yellow powder, yield: (90%); mp: 288–290 °C, ^1^H NMR (400 MHz, DMSO-*d*_*6*_) δ (ppm): 12.08 (s, 2H, COOH), 12.08 (s, 2H, NHCO), 9.59 (s, 1H), 8.10 (s, 1H), 7.78 (d, *J* = 8.8 Hz, 2H), 7.39 (d, *J* = 9.0 Hz, 1H), 7.28 (dd, *J* = 9.0, 2.7 Hz, 1H), 7.17 (d, *J* = 8.8 Hz, 2H), 6.67 (s, 1H), 6.53 (d, *J* = 2.7 Hz, 1H), 3.65 (s, 3H), 2.56–2.43 (m, 6H), 2.32 (s, 3H). ^13^C NMR (100 MHz, DMSO-*d*_*6*_) δ (ppm): 167.83, 161.43, 154.86, 150.26, 145.93, 140.76, 140.66, 138.25, 134.42, 131.47, 120.85, 120.81, 120.56, 120.35, 117.83, 117.68, 112.05, 106.67, 55.88, 40.60, 40.39, 40.18, 39.97, 39.76, 39.55, 39.34, 15.64, 13.12. HRMS (ESI, m/z) calcd for [C_23_H_22_N_5_O_4_]^+^ (M + H)^+^ 431.1594, found. 416.1666.

##### (E)-4-(2-(1-(1-(6-chloro-2-oxo-1,2-dihydroquinolin-4-yl)-5-methyl-1H-pyrazol-4-yl) ethylidene)hydrazineyl)benzoic acid (4i)

Pale yellow powder, yield: (78%); mp: 274–276 °C, ^1^H NMR (400 MHz, DMSO-*d*_*6*_) δ (ppm): 12.25 (s, 1H, COOH), 12.19 (s, 1H, s, 2H, COOH, NHCO), 9.58 (s, 1H), 8.09 (s, 1H), 7.75 (d, *J* = 7.8 Hz, 2H), 7.61 (d, *J* = 7.9 Hz, 1H), 7.42 (d, *J* = 8.5 Hz, 1H), 7.19–7.06 (m, 3H), 6.76 (s, 1H), 2.50 (s, 3H), 2.29 (s, 3H). ^13^C NMR (100 MHz, DMSO-*d*_*6*_) δ (ppm): 167.83, 161.66, 150.24, 145.12, 141.01, 140.50, 138.70, 138.53, 131.91, 131.47, 126.70, 124.06, 121.33, 120.78, 120.40, 118.32, 118.27, 112.06, 15.53, 13.09. HRMS (ESI, m/z) calcd for [C_22_H_19_^35^ClN_5_O_3_]^+^ (M + H)^+^ 436.1098, found. 416.1192.

##### (E)-4-(2-(1-(1-(6-bromo-2-oxo-1,2-dihydroquinolin-4-yl)-5-methyl-1H-pyrazol-4-yl) ethylidene)hydrazineyl)benzoic acid (4j)

Pale yellow powder, yield: (72%); mp: 281–283 °C, ^1^H NMR (400 MHz, DMSO-*d*_*6*_) δ (ppm): 12.29 (s, 2H, COOH), 12.29 (s, 2H, COOH, NHCO), 12.29 (s, 2H), 9.62 (s, 1H), 8.13 (s, 1H), 7.83–7.70 (m, 3H), 7.39 (d, *J* = 8.8 Hz, 1H), 7.27 (d, *J* = 2.1 Hz, 1H), 7.18 (d, *J* = 8.8 Hz, 2H), 6.78 (s, 1H), 2.54 (s, 3H), 2.33 (s, 3H). ^13^C NMR (100 MHz, DMSO-*d*_*6*_) δ (ppm): 167.83, 161.64, 150.23, 144.97, 141.09, 140.50, 139.01, 138.55, 134.56, 131.47, 127.04, 121.25, 120.78, 120.40, 118.77, 118.49, 114.42, 112.06, 15.65, 13.10. HRMS (ESI, m/z) calcd for [C_22_H_19_^79^BrN_3_O_3_]^+^ (M + H)^+^ 480.0593, found. 480.0669.

### Biology

#### Antiproliferative assay

Using Staurosporine as a control, the MTT assay was utilized to assess the antiproliferative effect of hybrids **4d**, **4e**, **4f**, and **4g** versus three human cancer cell lines: the leukemia (MOLT-4) cell line, the lung cancer (HOP-92) cell line, and the breast cancer (T47D) cell line. The median inhibitory concentration (IC_50_) and GI_50_ (average IC_50_) against the three cancer cell lines were calculated. See Appendix B.

#### EGFR inhibitory activity

The most potent Antiproliferative derivatives, **4d–g**, were tested for inhibitory effect against EGFR as a possible molecular target. Assessed using a 3-(4,5-dimethylthiazole-2-yl)-2,5-diphenyltetrazol (MTT) test. For more details, see Appendix B.

#### BRAFV600E inhibitory assay

Using Vemurafenib as the control medication, Compounds **4d**, **4e**, **4f**, and **4g** were further assessed for their inhibitory activity against BRAF^V600E^. Refer to Appendix B for further information.

### Docking study

Docking analyses of the most stable poses of 2-quinolone candidates incorporating pyrazole core **4d**, **4e**, **4f,** and **4g** along with the selected reference ligands: erlotinib and vemurafenib were accomplished against EGFR- and BRAF-active sites using Maestro software of Schrodinger 2022.4. The nominated biotargets are (1) EGFR (PDB ID: 1M17) [53] and (2) BRAF (PDB ID:5JRQ) [54] that were regained from RCSB protein data bank (PDB). The most stable conformers of the appointed 2-quinolones compounds and the biotargets reference ligands were built using ChemBiodraw 18.0 PerkinElmer software and cleaned up for bond alignment then imported with Schrodinger software in a 3D demonstration mode. The energy minimization has occurred using OPLS3e (Optimized Potentials for Liquid Simulations) [55] force field in Ligprep (Version 2022-4, Schrodinger) [56]. This minimization aids to assign bond orders, the hydrogens addition to the selected ligands, then the generated output files that denote the best poses of the ligands, were further utilized for docking analyses. Furthermore, the indicated targets were displayed for preparation using Protein preparation wizard (Version 2022-4, Schrodinger) [56] as a main tool for protein preparation. The hydrogens have been added to the proteins, the charges, and the produced Het states using Epik at pH 7.0 ± 2.0 were allocated. The targets have been exposed to Pre-process, protein modification, and refinement by the applicable chain selection and the water molecules have been removed. Lastly, the targets have been minimized using OPLS3 force field. Besides, the receptor grids were constructed by considering the co-crystal ligands (X-ray pose of the ligand in the protein). The centroid of the ligands has been selected to establish a grid box around it and VanderWaal radius of the target atoms was scaled to 1.00 Å with a partial atomic charge of 0.25. The molecular docking has been run using performed glide rigid docking protocol [57] by means of Maestro software of Schrodinger. All the docking estimates have been done using Extra Precision (XP) mode, where a scaling factor of 0.8 and a partial atomic charge < 0.15 were employed to the atoms of proteins. Glide docking scoring has been devoted for verifying the best-docked poses from the output, and the interactions of these docked poses have been further investigated using XP visualizer. After the last step of docking with the co-crystal ligand in XP mode, root-mean-square deviation (RMSD) was ensured to authorize the target, and the RMSD values lie within the range of 0.46 Å.

## Supplementary Information

Below is the link to the electronic supplementary material.

Supplementary file1 (DOCX 28806 KB)

Supplementary file2 (DOCX 32920 KB)

## Data Availability

Data will be made available on request. The authors declare that the data supporting the findings of this study are available within the paper and its Supplementary Information files. Should any raw data files be needed in another format they are available from the corresponding author upon reasonable request.
