# Supplementary material for: An innovative approach to development of new pyrazolylquinolin-2-one hybrids as dual EGFR and BRAFV600E inhibitors
Source: Mol Divers. 2025 Mar 8;29(6):6379–400. doi: 10.1007/s11030-025-11127-4 (PMC12638379; doi:10.1007/s11030-025-11127-4)
Supplement: Supplementary file 1 — Supplementary file1 (DOCX 28806 KB) [file 11030_2025_11127_MOESM1_ESM.docx]

**An innovative approach to development of new pyrazolylquinoline-2-one hybrids as dual EGFR and BRAF^V600E^ inhibitors**

Mohamed M. Hawwas^a^, Ahmed S. Mancy^b^, Mohamed Ramadan^a^, Tarek S. Ibrahim^c,d^, Ashraf H Bayoumi^e^, Mohamed Alswah^e^

*^a^ Department of Pharmaceutical Organic Chemistry, Faculty of Pharmacy, Al-Azhar University, Assiut, Egypt.*

*^b^ Department of Pharmacology and Experimental Neuroscience, University of Nebraska Medical Center, Omaha, NE 68198, USA.*

*^c^ Department of Pharmaceutical Chemistry, Faculty of Pharmacy, King Abdulaziz University, Jeddah, 21589, Saudi Arabia.*

*^d^ Department of Pharmaceutical Organic Chemistry, Faculty of Pharmacy, Zagazig University, Zagazig 44519, Egypt.*

*^e^ Department of Pharmaceutical Organic Chemistry, Faculty of Pharmacy, Al-Azhar University, Cairo, Egypt.*

*^e^ Department of Pharmaceutical Organic Chemistry, Faculty of Pharmacy, Al-Azhar University, Cairo, Egypt.*

^1^H NMR (400 MHz, DMSO*d_6_*) for compound 2a

^13^C NMR (100 MHz, DMSO *d_6_*) for compound 2a

^1^H NMR (400 MHz, DMSO*d_6_*) for compound 2b

^13^C NMR (100 MHz, DMSO *d_6_*) for compound 2b

^1^H NMR (400 MHz, DMSO*d_6_*) for compound 2c

^13^C NMR (100 MHz, DMSO *d_6_*) for compound 2c

^1^H NMR (400 MHz, DMSO*d_6_*) for compound 2d

^13^C NMR (100 MHz, DMSO *d_6_*) for compound 2d

^1^H NMR (400 MHz, DMSO*d_6_*) for compound 2e

^13^C NMR (100 MHz, DMSO *d_6_*) for compound 2e

^1^H NMR (400 MHz, DMSO*d_6_*) for compound 4a

^13^C NMR (100 MHz, DMSO *d_6_*) for compound 4a

^1^H NMR (400 MHz, DMSO*d_6_*) for compound 4b

^13^C NMR (100 MHz, DMSO *d_6_*) for compound 4b

^1^H NMR (400 MHz, DMSO*d_6_*) for compound 4c

^13^C NMR (100 MHz, DMSO *d_6_*) for compound 4c

^1^H NMR (400 MHz, DMSO*d_6_*) for compound 4d

^13^C NMR (100 MHz, DMSO *d_6_*) for compound 4d

^1^H NMR (400 MHz, DMSO*d_6_*) for compound 4e

^13^C NMR (100 MHz, DMSO *d_6_*) for compound 4e

^1^H NMR (400 MHz, DMSO*d_6_*) for compound 4f

^13^C NMR (100 MHz, DMSO *d_6_*) for compound 4f

^1^H NMR (400 MHz, DMSO*d_6_*) for compound 4g

^13^C NMR (100 MHz, DMSO *d_6_*) for compound 4g

^1^H NMR (400 MHz, DMSO*d_6_*) for compound 4h

^13^C NMR (100 MHz, DMSO *d_6_*) for compound 4h

^1^H NMR (400 MHz, DMSO*d_6_*) for compound 4i

^13^C NMR (100 MHz, DMSO *d_6_*) for compound 4i

^1^H NMR (400 MHz, DMSO*d_6_*) for compound 4j

^13^C NMR (100 MHz, DMSO *d_6_*) for compound 4j

HRMS for compound 4a


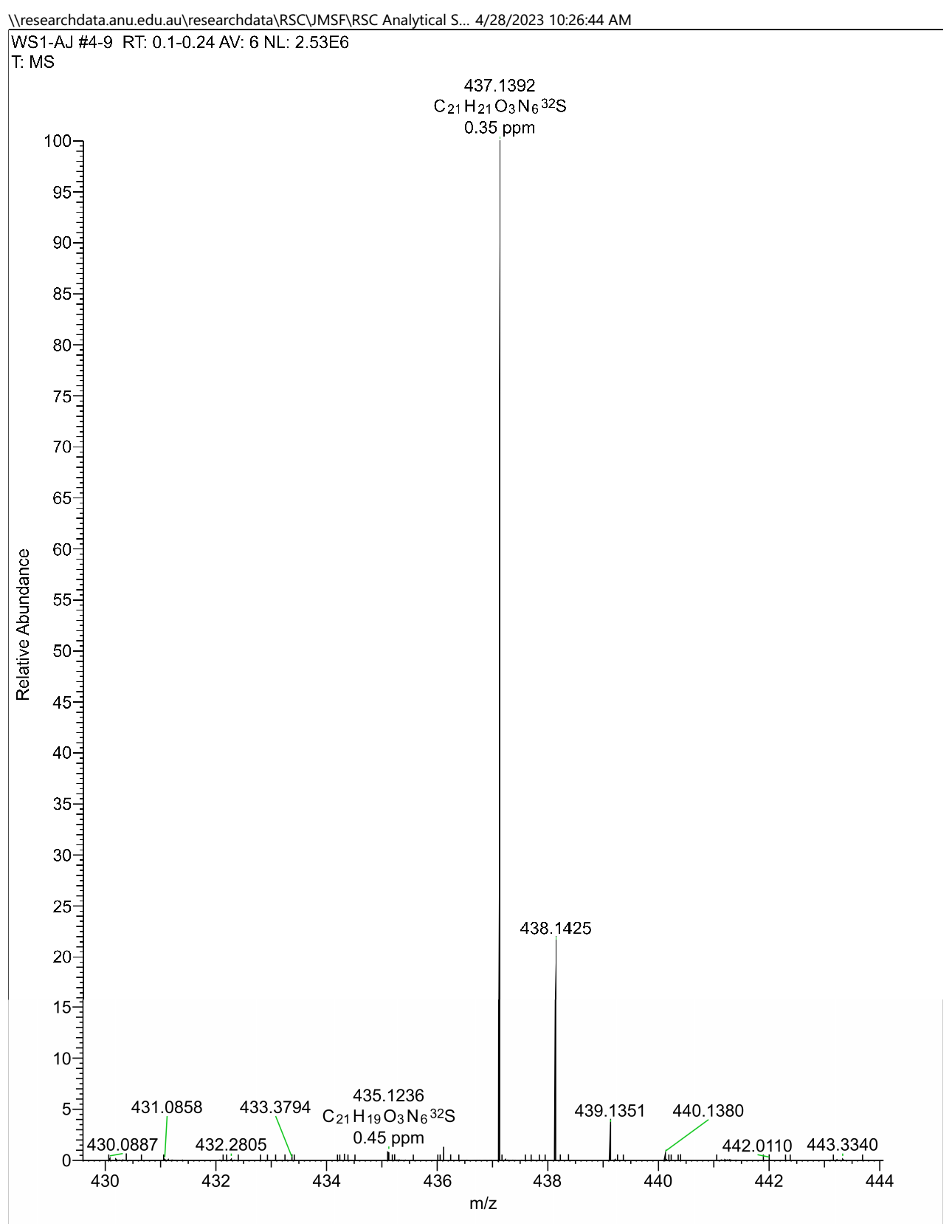


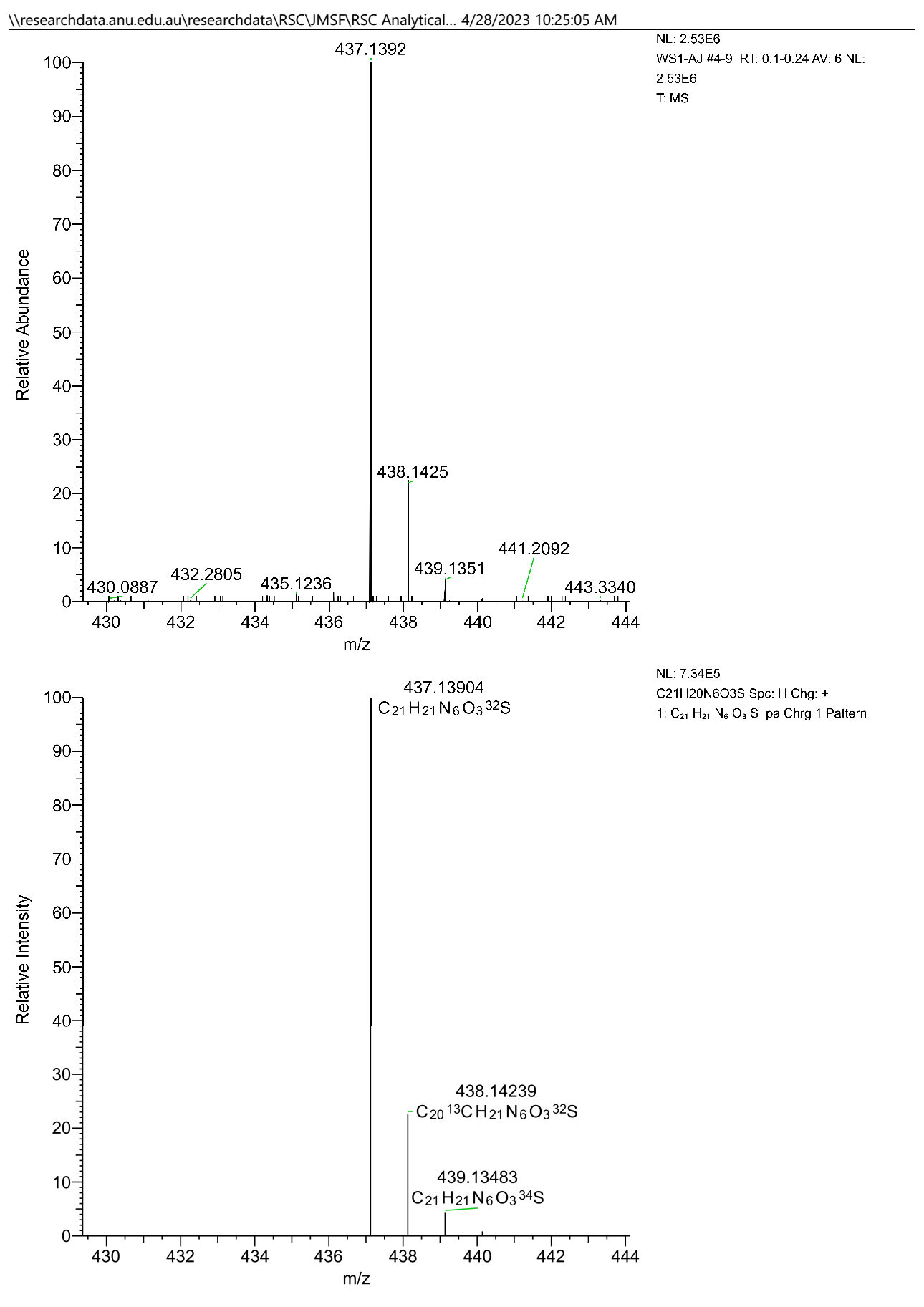


HRMS for compound 4b


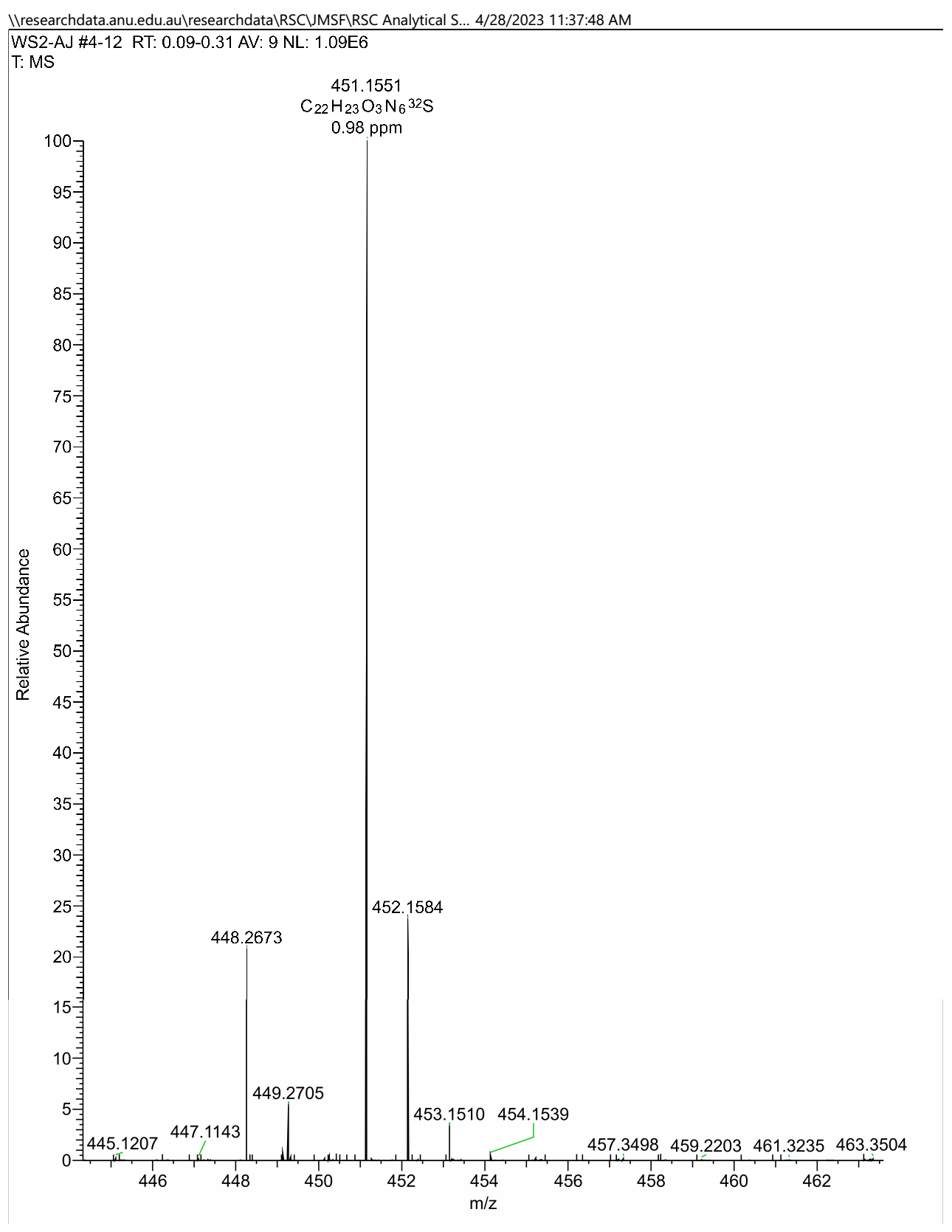


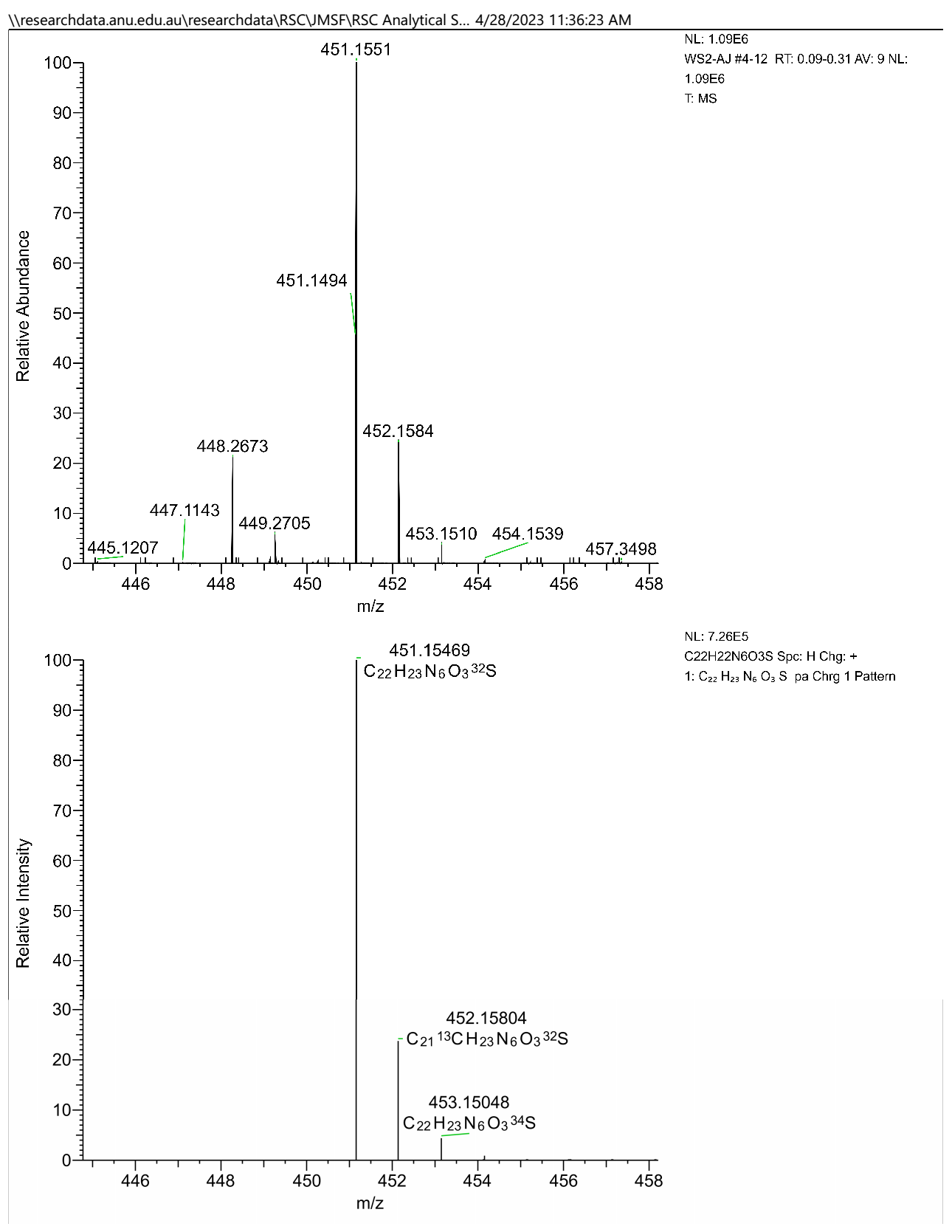


HRMS for compound 4c


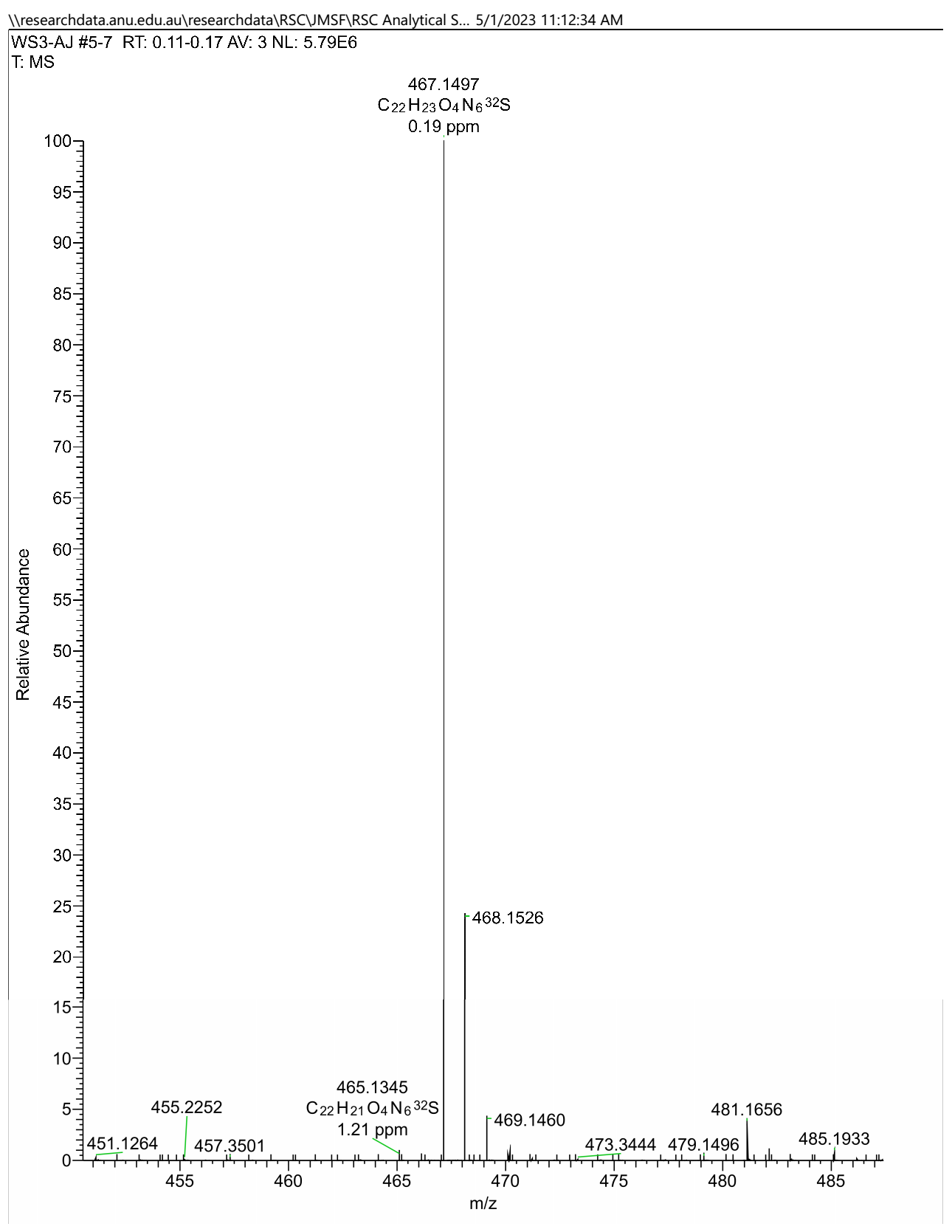


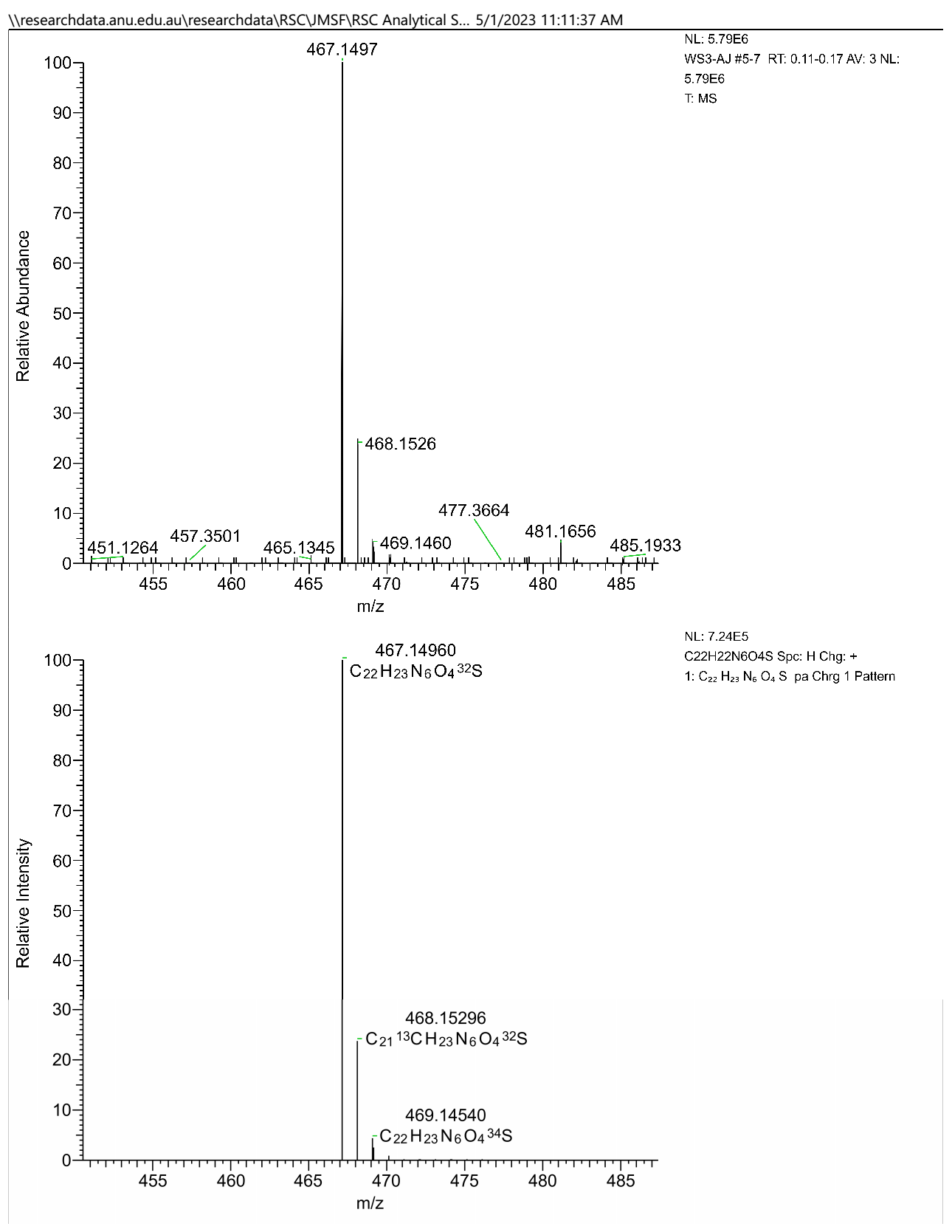


HRMS for compound 4d


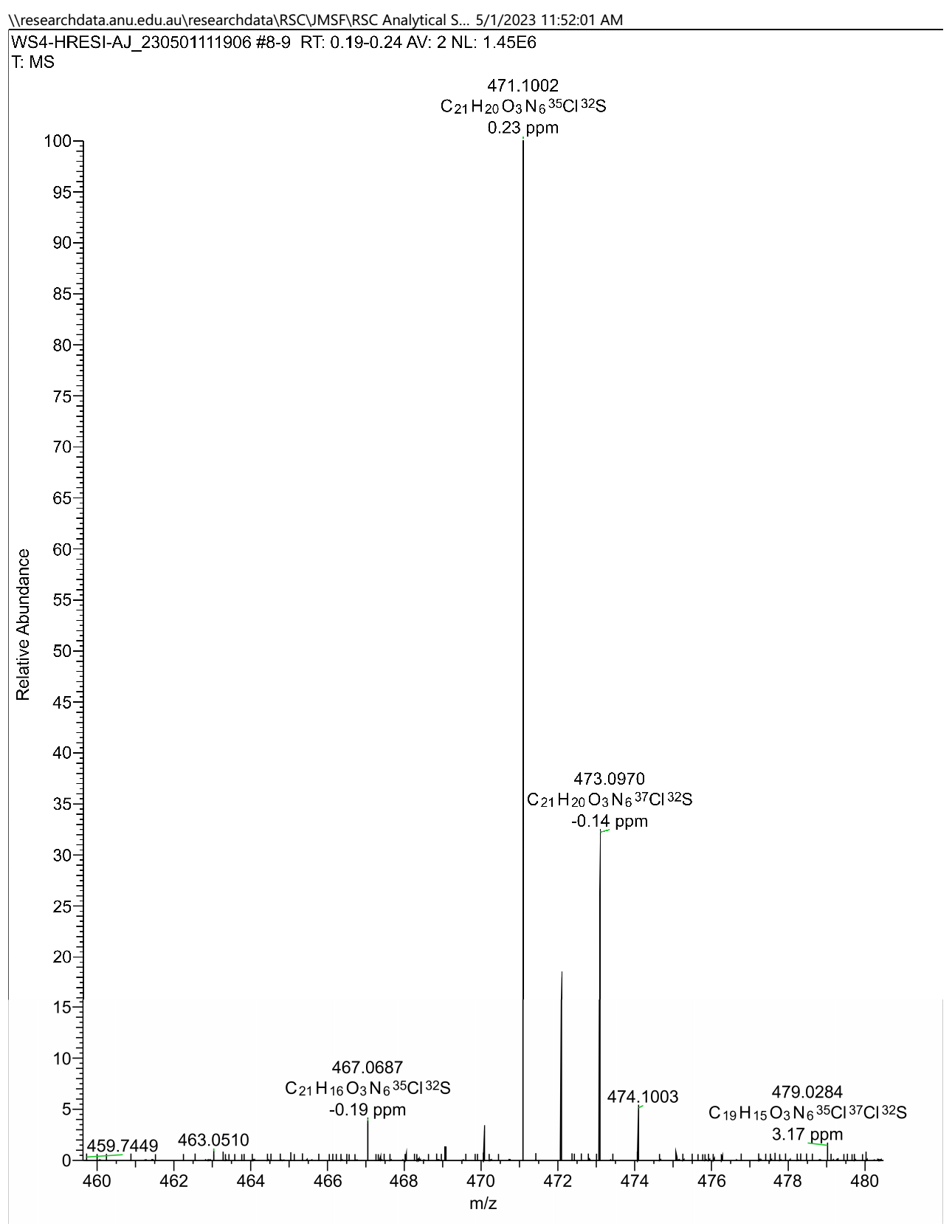


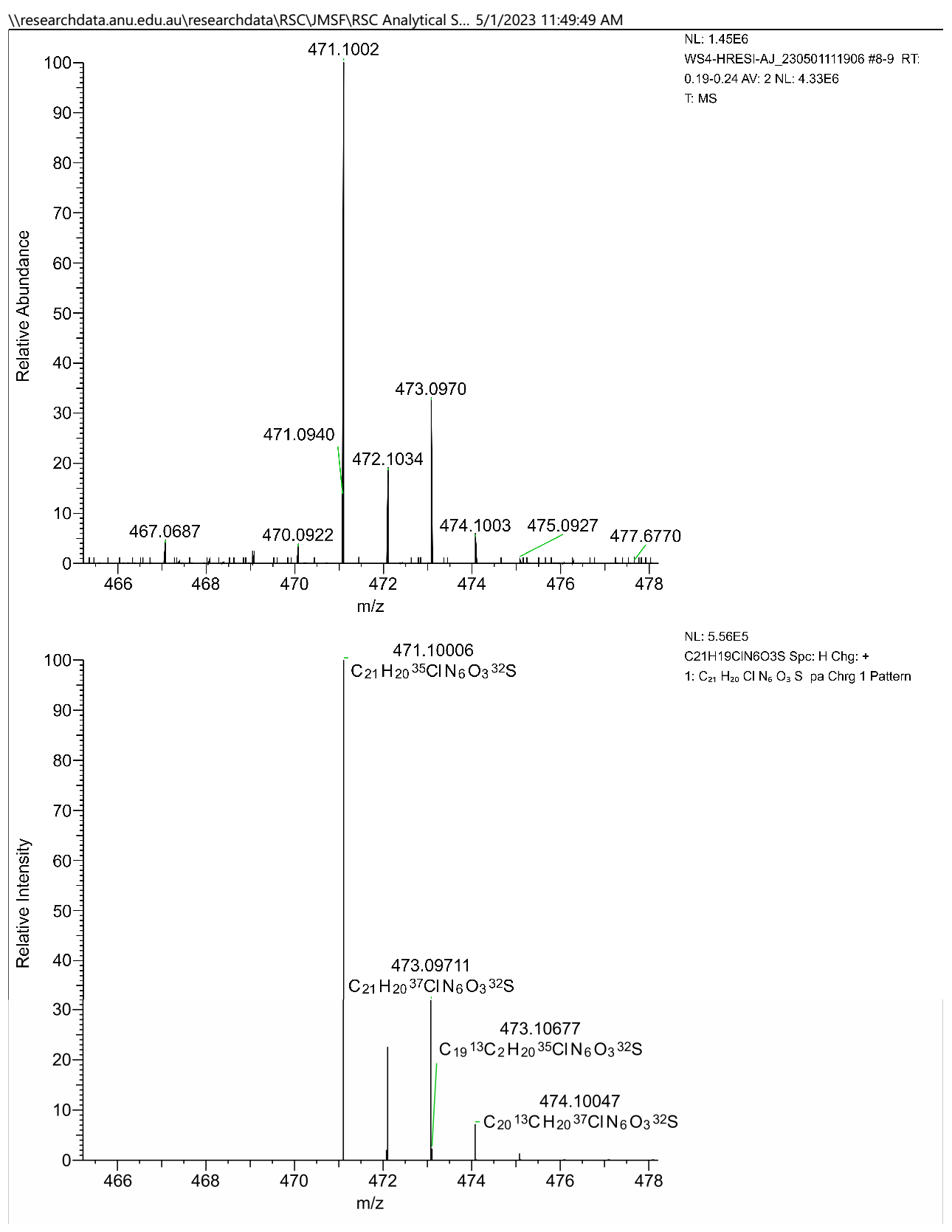


HRMS for compound 4e


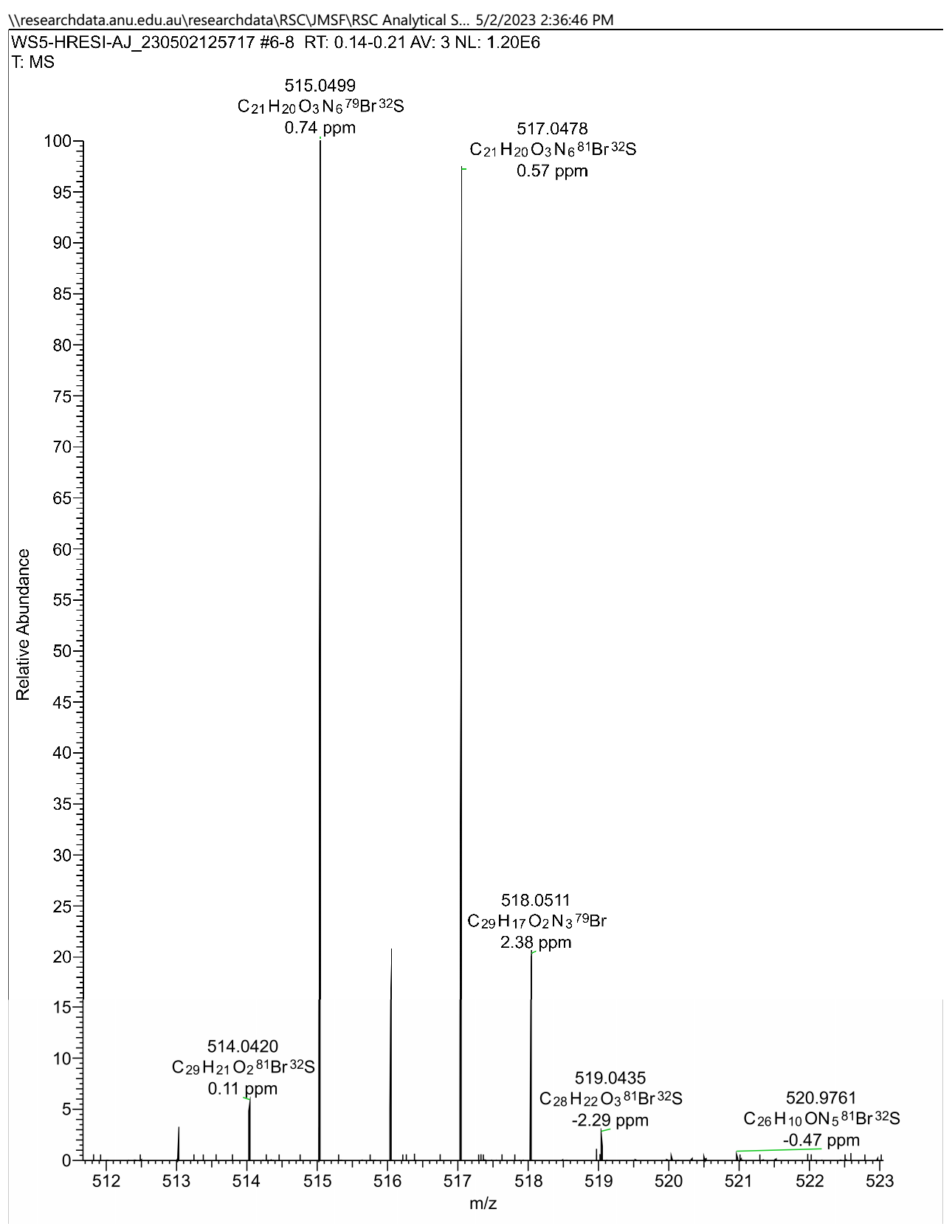


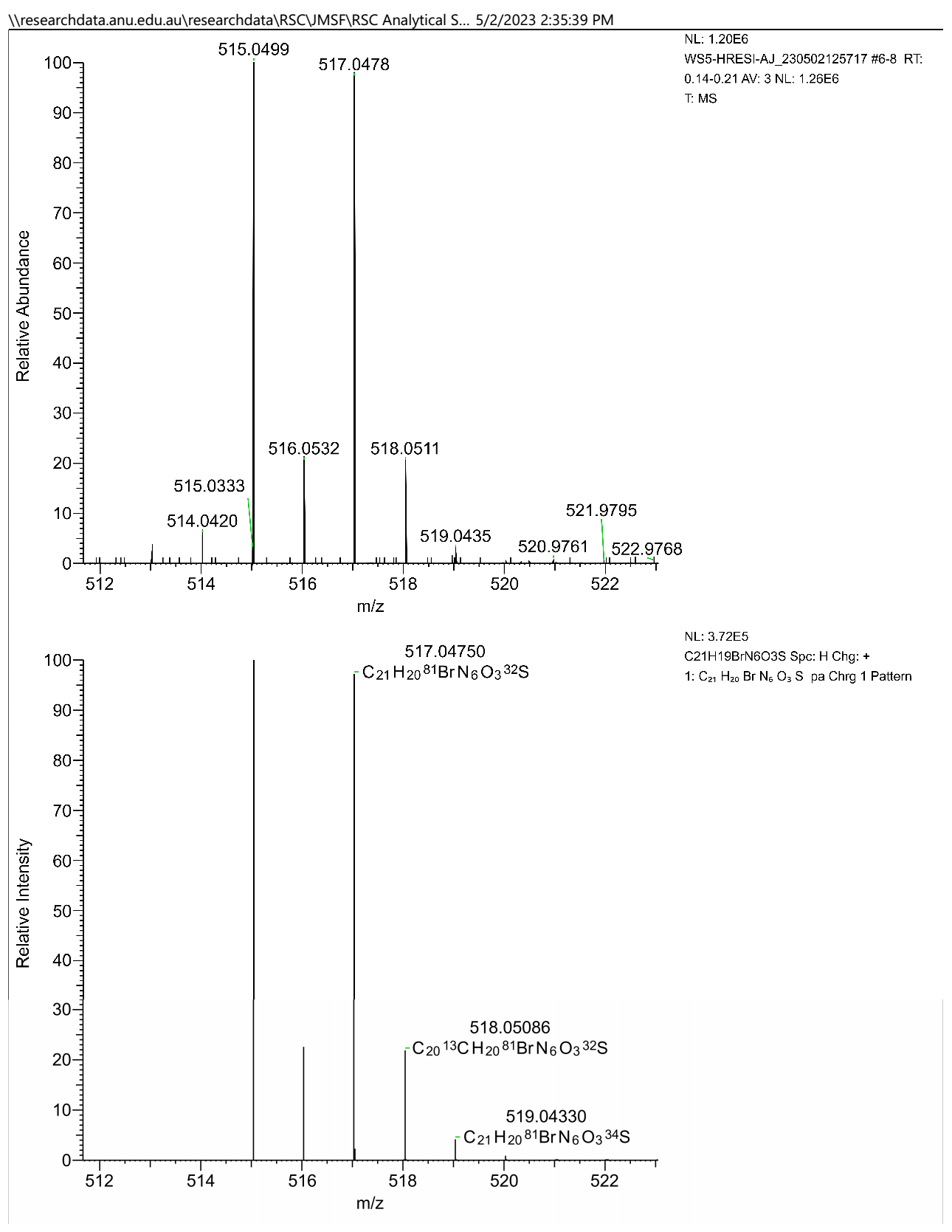


HRMS for compound 4f


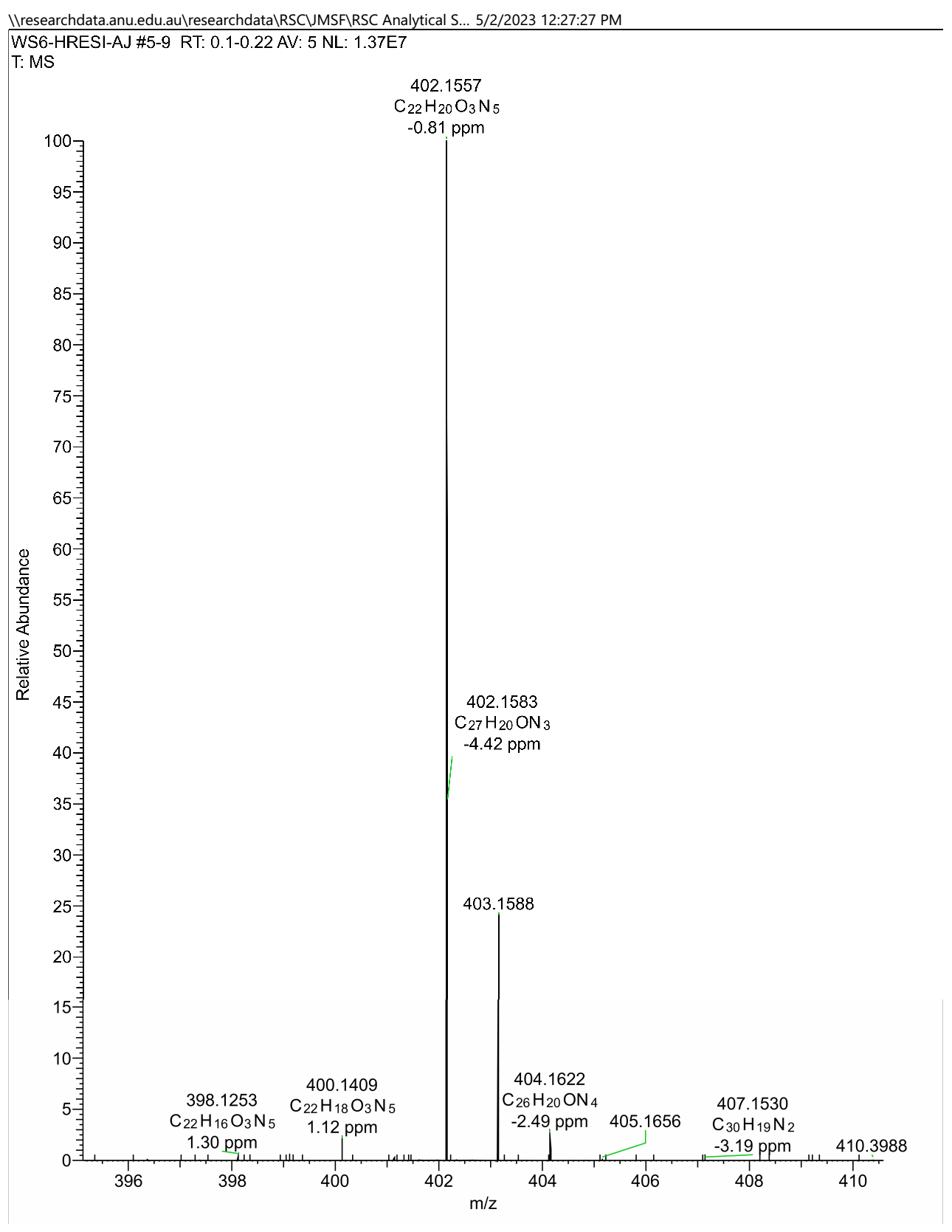


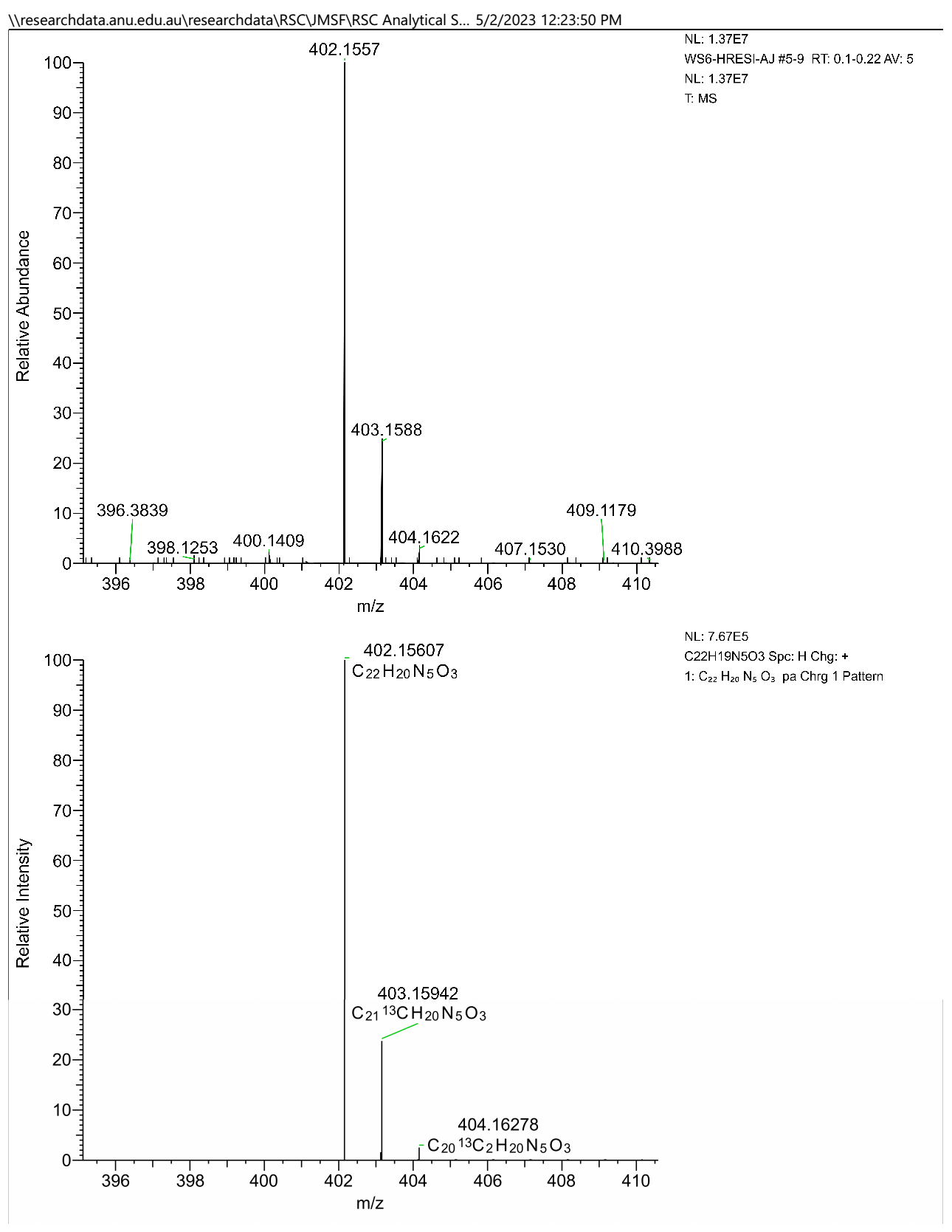


HRMS for compound 4g


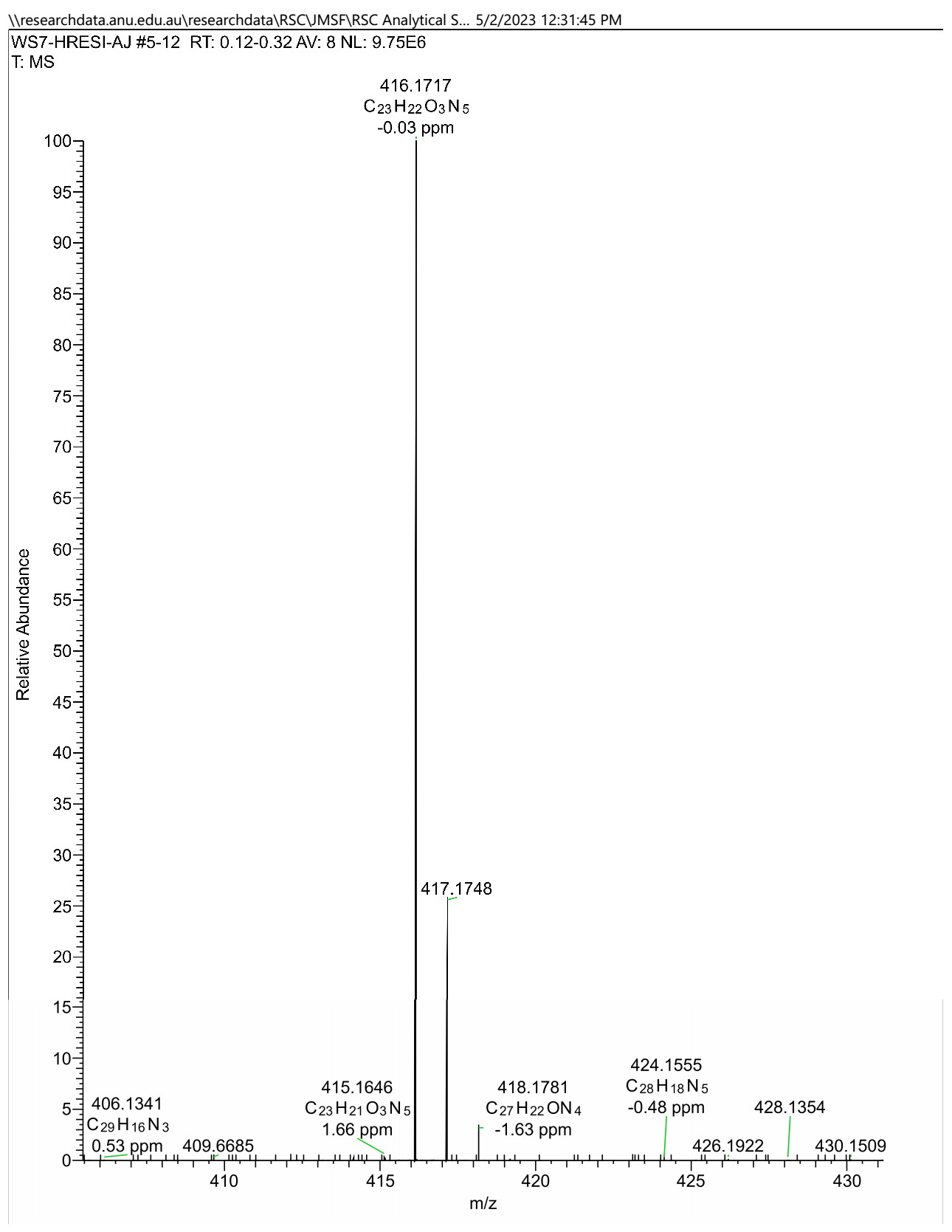


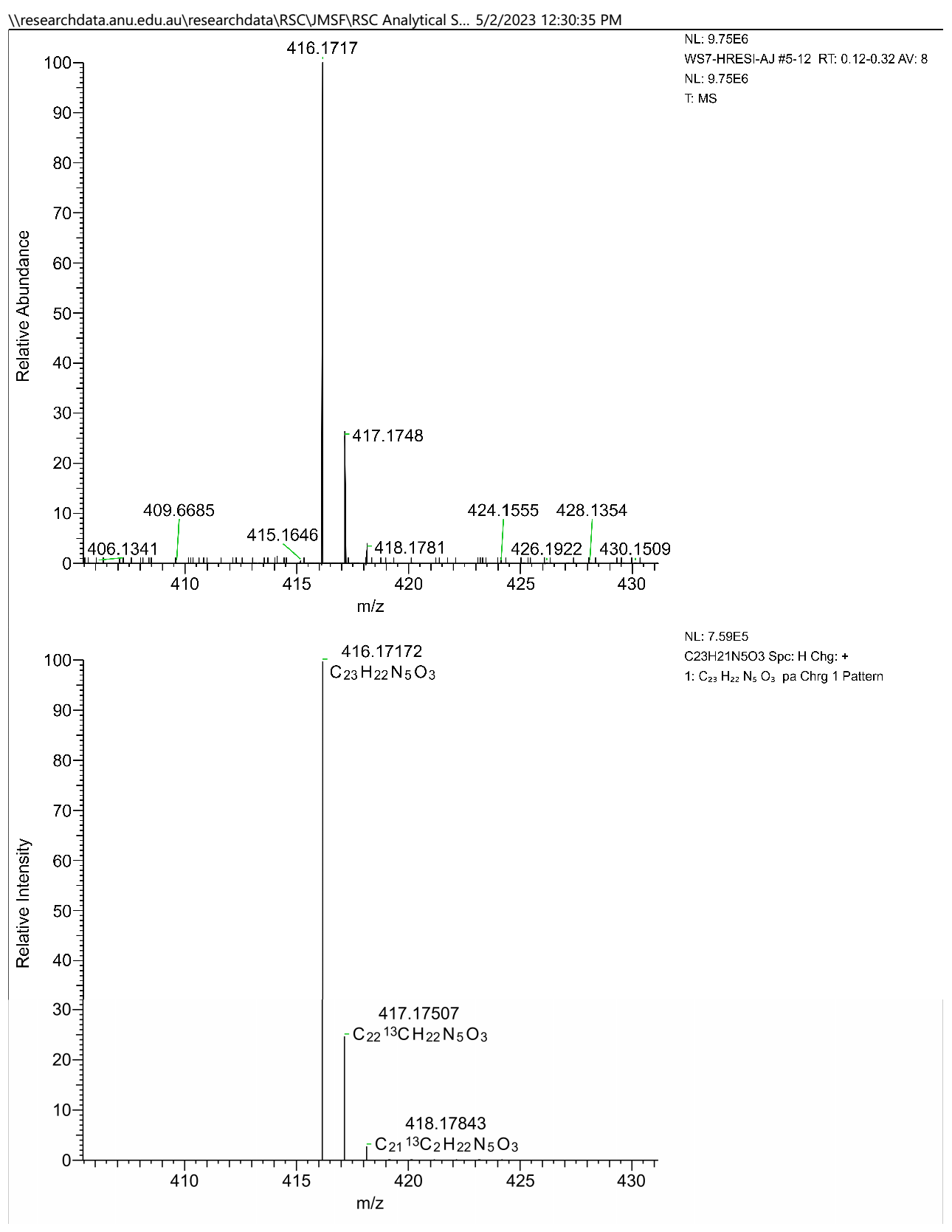


HRMS for compound 4h


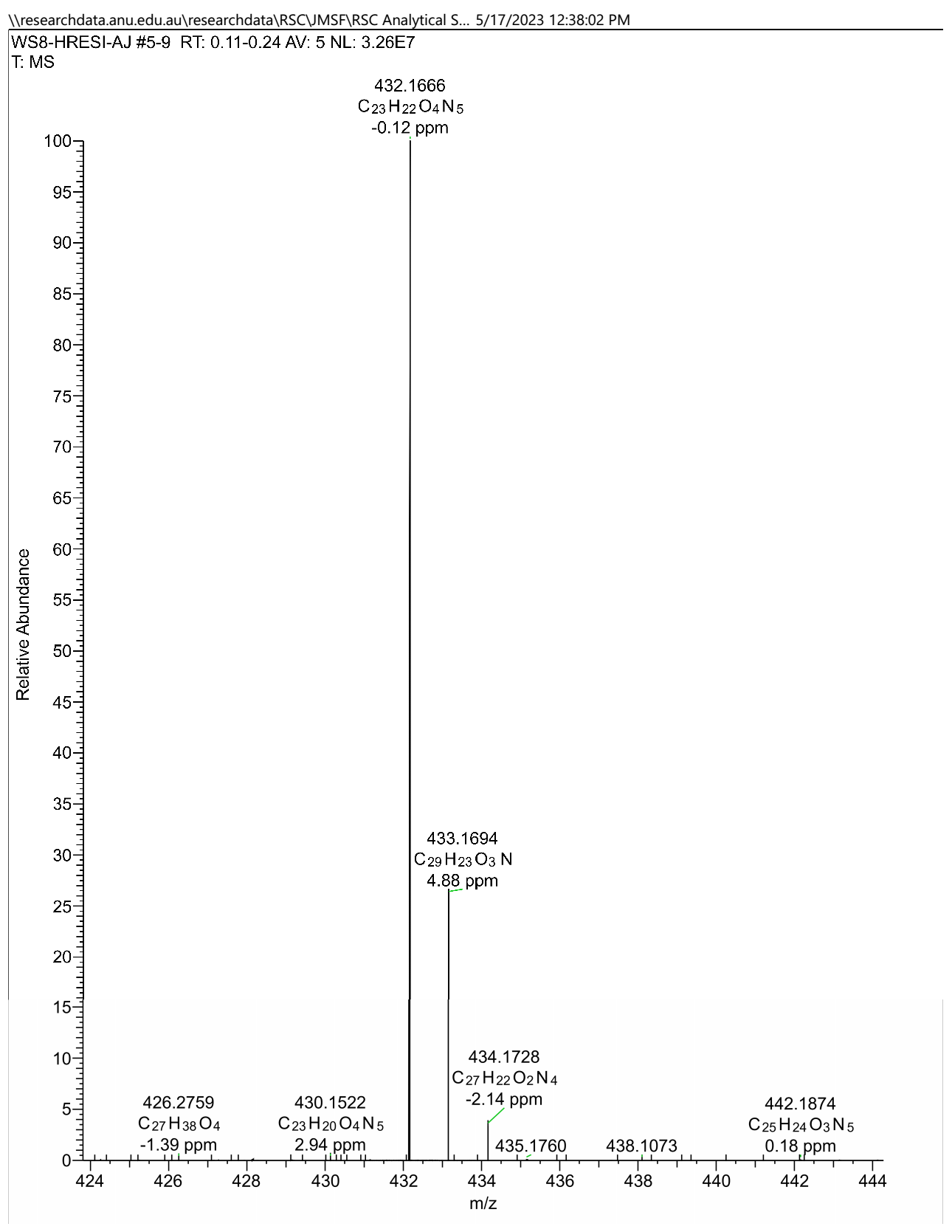


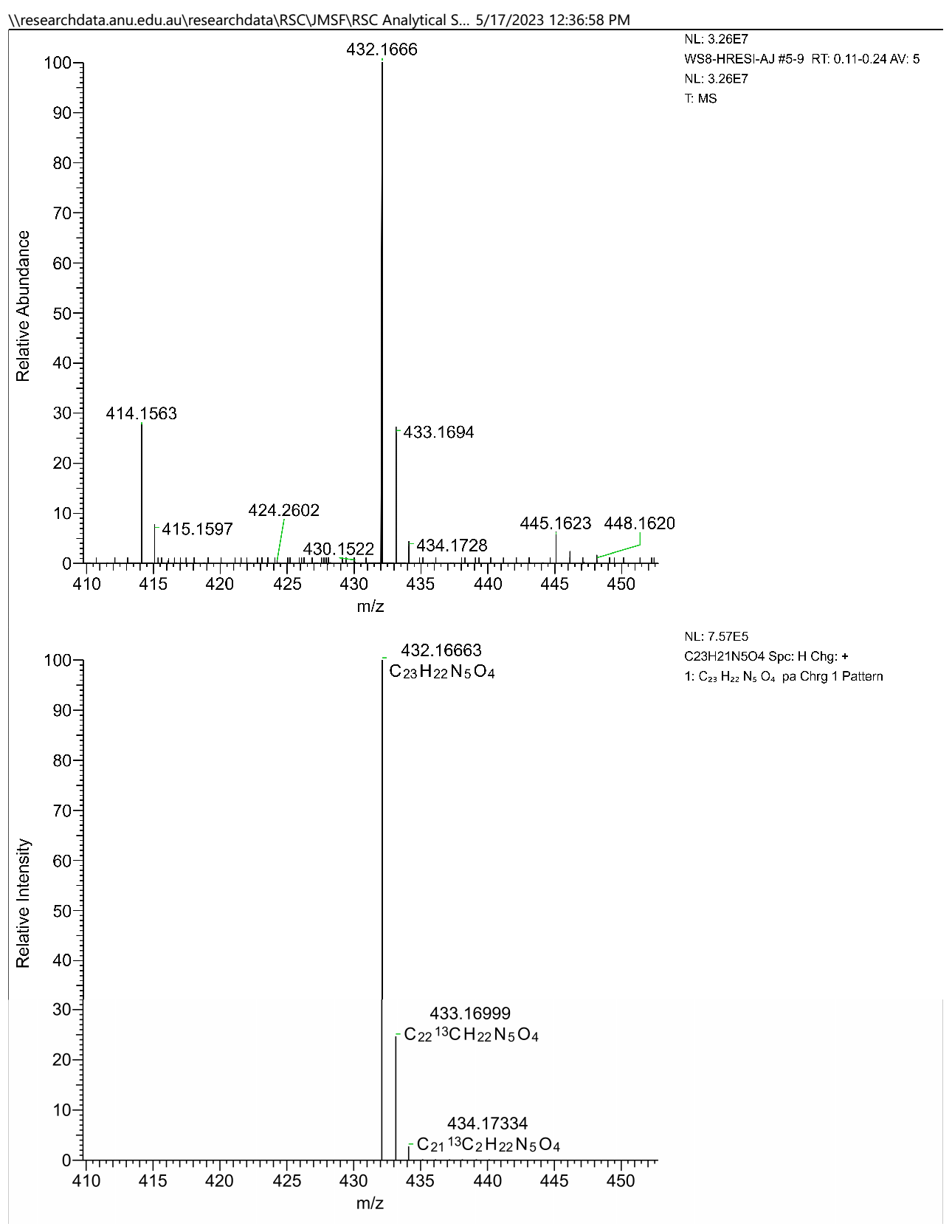


HRMS for compound 4i


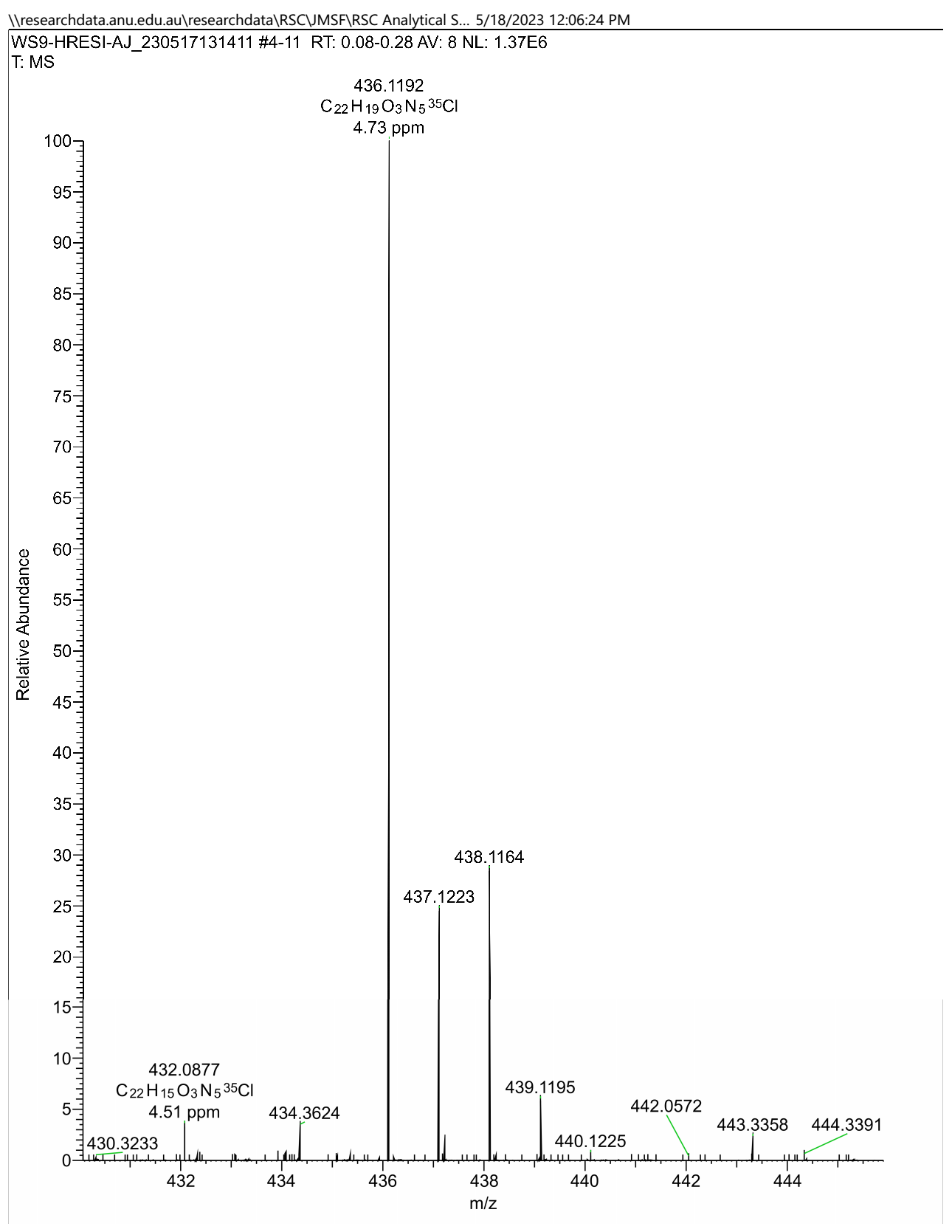


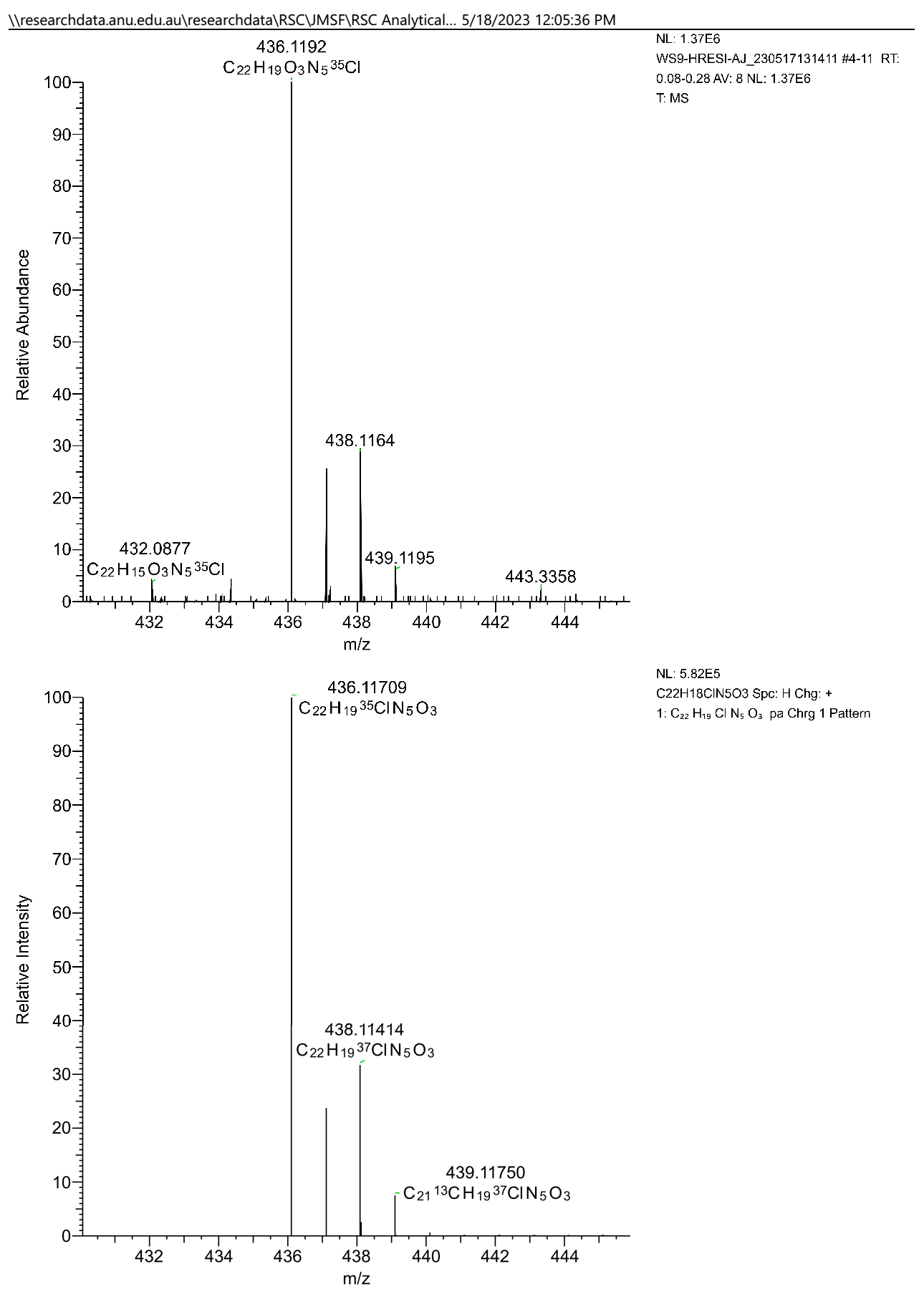


HRMS for compound 4j


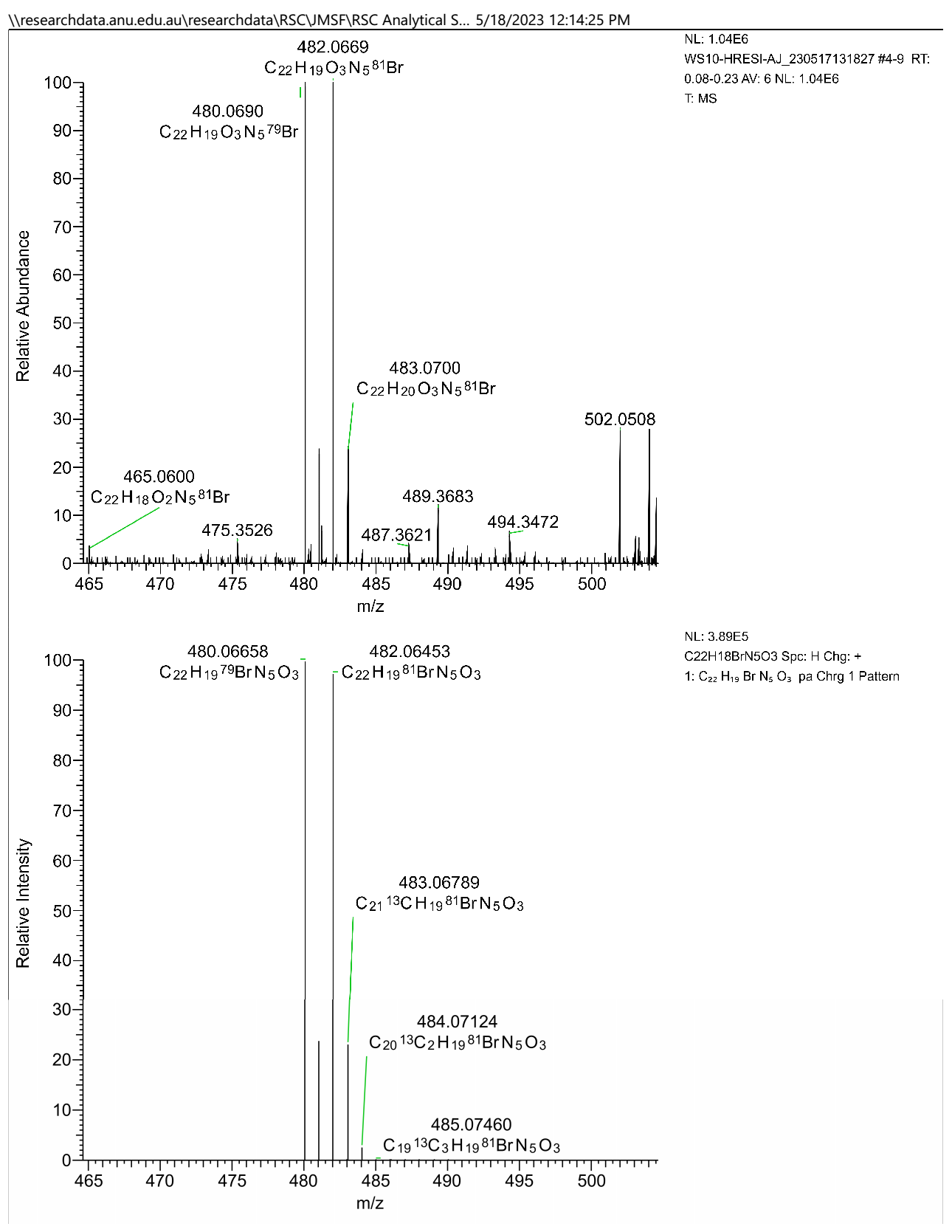


|  |  |  |
| --- | --- | --- |
| **Ws1** | **4a** |  |
| **Ws2** | **4b** |  |
| **Ws3** | **4c** |  |
| **Ws4** | **4d** |  |
| **Ws5** | **4e** |  |
| **Ws6** | **4f** |  |
| **Ws7** | **4g** |  |
| **Ws8** | **4h** |  |
| **Ws9** | **4i** |  |
| **Ws10** | **4j** |  |

**MTT assay**

| Ser | **cytotoxicity**  **IC50**  **uM** | | | | SD | | |
| --- | --- | --- | --- | --- | --- | --- | --- |
|  | code | **T-47D** | **HOP-92** | **MOLT-4** | | WI38 |  |
| 1 | **Ws4** | **13.85**±0.48 | **36.52**±1.46 | **7.24**±0.29 | | **62.46**±2.49 |  |
| 2 | **WS5** | **8.62**±0.34 | **4.982**±0.2 | **8.023**±0.31 | | **29.62**±1.18 |  |
| 3 | **ws6** | **28.56**±0.99 | **24.93**±1**.9** | **16.31**±0.65 | | **42.14**±1.68 |  |
| 4 | **Ws7** | **9.71**±0.34 | **14.72**±0.66 | **6.17**±0.25 | | **37.57**±1.5 |  |
| *** | **Staurosporine** | **4.94**±0.19 | **3.172**±0.19 | **5.856**±0.23 | | **17.54**±1.5 |  |

| researcher |  | assay |  |  | Date |  |  | cells |  |  |
| --- | --- | --- | --- | --- | --- | --- | --- | --- | --- | --- |
| Dr.Mohamed Hawas | | MTT |  |  | 18/10/2023 | |  | **T-47D** | **HOP-92** | **MOLT-4** |

|  | **Blank** | **CC** | **Sample No. Ws4/HOP-92** | | | | | **Sample No. ws6/HOP-92** | | | | |
| --- | --- | --- | --- | --- | --- | --- | --- | --- | --- | --- | --- | --- |
|  | **1** | **2** | **3** | **4** | **5** | **6** | **7** | **8** | **9** | **10** | **11** | **12** |
| A | B | C | 100uM | 25uM | 6.3uM | 1.6uM | 0.4uM | 100uM | 25uM | 6.3uM | 1.6uM | 0.4uM |
| B | B | C | 100uM | 25uM | 6.3uM | 1.6uM | 0.4uM | 100uM | 25uM | 6.3uM | 1.6uM | 0.4uM |
| C | B | C | 100uM | 25uM | 6.3uM | 1.6uM | 0.4uM | 100uM | 25uM | 6.3uM | 1.6uM | 0.4uM |

| ROBONIK P2000 Eia reader | | | |  |  |  |  |  |  |  |  |  |
| --- | --- | --- | --- | --- | --- | --- | --- | --- | --- | --- | --- | --- |
| Wave length: 450 nm | | | |  |  |  |  |  |  |  |  |  |
| Reference: 630 nm | | | |  |  |  |  |  |  |  |  |  |
|  | **1** | **2** | **3** | **4** | **5** | **6** | **7** | **8** | **9** | **10** | **11** | **12** |
|  |  |  |  |  |  |  |  |  |  |  |  |  |
| A | 0.001 | 0.529 | 0.213 | 0.269 | 0.328 | 0.386 | 0.427 | 0.182 | 0.261 | 0.328 | 0.372 | 0.422 |
| B | 0.003 | 0.494 | 0.196 | 0.254 | 0.314 | 0.371 | 0.434 | 0.177 | 0.254 | 0.311 | 0.359 | 0.413 |
| C | 0.001 | 0.485 | 0.227 | 0.263 | 0.331 | 0.388 | 0.424 | 0.174 | 0.266 | 0.331 | 0.391 | 0.422 |
| mean | 0.002 | 0.503 | 0.212 | 0.262 | 0.3243 | 0.3817 | 0.4283 | 0.1777 | 0.26033 | 0.3233 | 0.374 | 0.419 |
| % viability |  |  | 42.1751 | 52.122 | 64.523 | 75.928 | 85.212 | 35.345 | 51.7905 | 64.324 | 74.4 | 83.36 |
| Ws4/HOP-92 | | 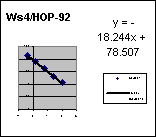 |  |  |  |  |  | ws6/HOP-92 | | 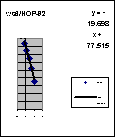 |  |  |
| 2 | 42.18 |  |  |  |  |  |  | 2 | 35.3448 |  |  |  |
| 1.4 | 52.12 |  |  |  |  |  |  | 1.3979 | 51.7905 |  |  |  |
| 0.8 | 64.52 |  |  |  |  |  |  | 0.7959 | 64.3236 |  |  |  |
| 0.19 | 75.93 |  |  |  |  |  |  | 0.1931 | 74.4032 |  |  |  |
| -0.41 | 85.21 |  |  |  |  |  |  | -0.4089 | 83.3554 |  |  |  |
|  |  |  |  |  |  |  |  |  |  |  |  |  |
| IC50= |  |  |  |  |  |  |  | IC50= |  |  |  |  |

| ROBONIK P2000 eia reader | | | |  |  |  |  |  |  |  |  |  |
| --- | --- | --- | --- | --- | --- | --- | --- | --- | --- | --- | --- | --- |
| Wave length: | | 450 nm |  |  |  |  |  |  |  |  |  |  |
| Reference: 630 nm | | | |  |  |  |  |  |  |  |  |  |
|  | **1** | **2** | **3** | **4** | **5** | **6** | **7** | **8** | **9** | **10** | **11** | **12** |
|  |  |  |  |  |  |  |  |  |  |  |  |  |
| A | 0.001 | 0.611 | 0.154 | 0.202 | 0.276 | 0.359 | 0.418 | 0.121 | 0.144 | 0.262 | 0.344 | 0.414 |
| B | 0.001 | 0.603 | 0.161 | 0.208 | 0.285 | 0.364 | 0.441 | 0.104 | 0.132 | 0.259 | 0.392 | 0.428 |
| C | 0.001 | 0.598 | 0.165 | 0.214 | 0.306 | 0.371 | 0.435 | 0.086 | 0.171 | 0.261 | 0.373 | 0.415 |
| mean | 0.001 | 0.604 | 0.16 | 0.208 | 0.289 | 0.365 | 0.431 | 0.1037 | 0.149 | 0.2607 | 0.3697 | 0.419 |
| % viability |  |  | 26.49 | 34.44 | 47.85 | 60.38 | 71.41 | 17.163 | 24.67 | 43.157 | 61.203 | 69.371 |
| Ws5/HOP-92 | | 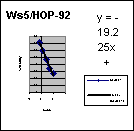 | | | |  |  | STA/HOP-92 | | 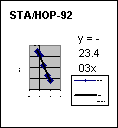 | | |
| 2 | 26.49 |  |  |  |  |  |  | 2 | 17.16 |  |  |  |
| 1.398 | 34.437 |  |  |  |  |  |  | 1.3979 | 24.67 |  |  |  |
| 0.796 | 47.848 |  |  |  |  |  |  | 0.7959 | 43.16 |  |  |  |
| 0.193 | 60.375 |  |  |  |  |  |  | 0.1931 | 61.2 |  |  |  |
| -0.41 | 71.413 |  |  |  |  |  |  | -0.409 | 69.37 |  |  |  |
|  |  |  |  |  |  |  |  |  |  |  |  |  |
| IC50= |  |  |  |  |  |  |  | IC50= |  |  |  |  |
|  |  |  |  |  |  |  |  |  |  |  |  |  |

|  | **Blank** | **CC** | **Sample No. Ws7/HOP-92** | | | | |
| --- | --- | --- | --- | --- | --- | --- | --- |
|  | **1** | **2** | **3** | **4** | **5** | **6** | **7** |
| A | B | C | 100uM | 25uM | 6.3uM | 1.6uM | 0.4uM |
| B | B | C | 100uM | 25uM | 6.3uM | 1.6uM | 0.4uM |
| C | B | C | 100uM | 25uM | 6.3uM | 1.6uM | 0.4uM |
| ROBONIK P2000 Eia reader | | | |  |  |  |  |
| Wave length: 450 nm | | | |  |  |  |  |
| Reference: 630 nm | | | |  |  |  |  |
|  | **1** | **2** | **3** | **4** | **5** | **6** | **7** |
|  |  |  |  |  |  |  |  |
| A | 0.001 | 0.563 | 0.188 | 0.262 | 0.311 | 0.371 | 0.424 |
| B | 0.001 | 0.558 | 0.194 | 0.278 | 0.307 | 0.385 | 0.437 |
| C | 0.001 | 0.549 | 0.181 | 0.262 | 0.326 | 0.363 | 0.441 |
| mean | 0.001 | 0.557 | 0.18767 | 0.2673 | 0.3147 | 0.373 | 0.434 |
| % viability |  |  | 33.7126 | 48.024 | 56.527 | 67.006 | 77.964 |
| Ws7/HOP-92 | |  |  | 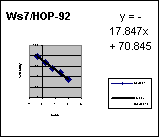 |  |  |  |
| 2 | 33.71 |  |  |  |  |  |  |
| 1.4 | 48.02 |  |  |  |  |  |  |

|  |  |  |  |  |  |  |  |  |  |  |  |  |
| --- | --- | --- | --- | --- | --- | --- | --- | --- | --- | --- | --- | --- |
|  |  |  |  |  |  |  |  |  |  |  |  |  |
|  | **Blank** | **CC** | **Sample No. Ws4/T-47D** | | | | | **Sample No. ws6/T-47D** | | | | |
|  | **1** | **2** | **3** | **4** | **5** | **6** | **7** | **8** | **9** | **10** | **11** | **12** |
| A | B | C | 100uM | 25uM | 6.3uM | 1.6uM | 0.4uM | 100uM | 25uM | 6.3uM | 1.6uM | 0.4uM |
| B | B | C | 100uM | 25uM | 6.3uM | 1.6uM | 0.4uM | 100uM | 25uM | 6.3uM | 1.6uM | 0.4uM |
| C | B | C | 100uM | 25uM | 6.3uM | 1.6uM | 0.4uM | 100uM | 25uM | 6.3uM | 1.6uM | 0.4uM |
| ROBONIK P2000 Eia reader | | | |  |  |  |  |  |  |  |  |  |
| Wave length: 450 nm | | | |  |  |  |  |  |  |  |  |  |
| Reference: 630 nm | | | |  |  |  |  |  |  |  |  |  |
|  | **1** | **2** | **3** | **4** | **5** | **6** | **7** | **8** | **9** | **10** | **11** | **12** |
|  |  |  |  |  |  |  |  |  |  |  |  |  |
| A | 0.001 | 0.549 | 0.192 | 0.246 | 0.311 | 0.364 | 0.425 | 0.228 | 0.284 | 0.342 | 0.392 | 0.428 |
| B | 0.003 | 0.532 | 0.178 | 0.259 | 0.327 | 0.371 | 0.411 | 0.219 | 0.276 | 0.337 | 0.404 | 0.431 |
| C | 0.001 | 0.555 | 0.179 | 0.244 | 0.292 | 0.379 | 0.427 | 0.225 | 0.259 | 0.325 | 0.384 | 0.427 |
| mean | 0.002 | 0.545 | 0.183 | 0.2497 | 0.31 | 0.3713 | 0.421 | 0.224 | 0.273 | 0.3347 | 0.393 | 0.429 |
| % viability |  |  | 33.5575 | 45.782 | 56.846 | 68.093 | 77.2 | 41.076 | 50.0611 | 61.369 | 72.13 | 78.61 |
| Ws4/T-47D | | 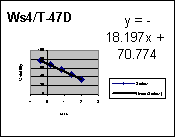 |  |  |  |  |  | ws6/T-47D | | 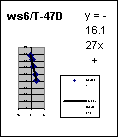 |  |  |
| 2 | 33.56 |  |  |  |  |  |  | 2 | 41.0758 |  |  |  |
| 1.4 | 45.78 |  |  |  |  |  |  | 1.3979 | 50.0611 |  |  |  |
| 0.8 | 56.85 |  |  |  |  |  |  | 0.7959 | 61.3692 |  |  |  |
| 0.19 | 68.09 |  |  |  |  |  |  | 0.1931 | 72.1271 |  |  |  |

|  |  |  |  |  |  |  |  |  | |  |  | |  |  |
| --- | --- | --- | --- | --- | --- | --- | --- | --- | --- | --- | --- | --- | --- | --- |
|  | **Blank** | **CC** | **Sample No. Ws4/MOLT-4** | | | | | **Sample No. ws6/MOLT-4** | | | | | | |
|  | **1** | **2** | **3** | **4** | **5** | **6** | **7** | **8** | | **9** | **10** | | **11** | **12** |
| A | B | C | 100uM | 25uM | 6.3uM | 1.6uM | 0.4uM | 100uM | | 25uM | 6.3uM | | 1.6uM | 0.4uM |
| B | B | C | 100uM | 25uM | 6.3uM | 1.6uM | 0.4uM | 100uM | | 25uM | 6.3uM | | 1.6uM | 0.4uM |
| C | B | C | 100uM | 25uM | 6.3uM | 1.6uM | 0.4uM | 100uM | | 25uM | 6.3uM | | 1.6uM | 0.4uM |
| ROBONIK P2000 Eia reader | | | |  |  |  |  |  | |  |  | |  |  |
| Wave length: 450 nm | | | |  |  |  |  |  | |  |  | |  |  |
| Reference: 630 nm | | | |  |  |  |  |  | |  |  | |  |  |
|  | **1** | **2** | **3** | **4** | **5** | **6** | **7** | **8** | | **9** | **10** | | **11** | **12** |
|  |  |  |  |  |  |  |  |  | |  |  | |  |  |
| A | 0.001 | 0.569 | 0.159 | 0.231 | 0.284 | 0.331 | 0.404 | 0.221 | | 0.267 | 0.323 | | 0.331 | 0.385 |
| B | 0.002 | 0.547 | 0.167 | 0.226 | 0.291 | 0.364 | 0.382 | 0.216 | | 0.265 | 0.325 | | 0.329 | 0.393 |
| C | 0.001 | 0.551 | 0.178 | 0.225 | 0.286 | 0.351 | 0.379 | 0.215 | | 0.269 | 0.328 | | 0.333 | 0.394 |
| mean | 0.001 | 0.556 | 0.168 | 0.2273 | 0.287 | 0.3487 | 0.3883 | 0.2173 | | 0.267 | 0.3253 | | 0.331 | 0.391 |
| % viability |  |  | 30.234 | 40.912 | 51.65 | 62.747 | 69.886 | 39.112 | | 48.0504 | 58.548 | | 59.56 | 70.31 |
| Ws4/MOLT-4 | | 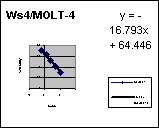 | | | |  |  | ws6/MOLT-4 | | | 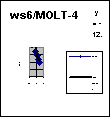 | |  |  |
| 2 | 30.23 |  |  |  |  |  |  | 2 | | 39.1122 |  | |  |  |
| 1.4 | 40.91 |  |  |  |  |  |  | 1.3979 | | 48.0504 |  | |  |  |
| 0.8 | 51.65 |  |  |  |  |  |  | 0.7959 | | 58.5483 |  | |  |  |
| 0.19 | 62.75 |  |  |  |  |  |  | 0.1931 | | 59.5561 |  | |  |  |
| -0.41 | 69.89 |  |  |  |  |  |  | -0.4089 | | 70.3059 |  | |  |  |
|  |  |  |  |  |  |  |  |  | |  |  | |  |  |
| IC50= |  |  |  |  |  |  |  | IC50= | |  |  | |  |  |
|  |  |  |  |  |  |  |  |  | |  |  | |  |  |
|  |  |  |  |  |  |  |  |  | |  |  | |  |  |
|  | **Blank** | **CC** | **Sample No. Ws7/MOLT-4** | | | | | **Sample No. STA/MOLT-4** | | | | | | |
|  | **1** | **2** | **3** | **4** | **5** | **6** | **7** | **8** | | **9** | **10** | | **11** | **12** |
| A | B | C | 100uM | 25uM | 6.3uM | 1.6uM | 0.4uM | 100uM | | 25uM | 6.3uM | | 1.6uM | 0.4uM |
| B | B | C | 100uM | 25uM | 6.3uM | 1.6uM | 0.4uM | 100uM | | 25uM | 6.3uM | | 1.6uM | 0.4uM |
| C | B | C | 100uM | 25uM | 6.3uM | 1.6uM | 0.4uM | 100uM | | 25uM | 6.3uM | | 1.6uM | 0.4uM |
| ROBONIK P2000 Eia reader | | | |  |  |  |  |  | |  |  | |  |  |
| Wave length: 450 nm | | | |  |  |  |  |  | |  |  | |  |  |
| Reference: 630 nm | | | |  |  |  |  |  | |  |  | |  |  |
|  | **1** | **2** | **3** | **4** | **5** | **6** | **7** |  |  |  |  |  |  |  |
|  |  |  |  |  |  |  |  |  |  |  |  |  |  |  |
| A | 0.003 | 0.611 | 0.213 | 0.257 | 0.294 | 0.333 | 0.414 |  |  |  |  |  |  |  |
| B | 0.001 | 0.628 | 0.217 | 0.259 | 0.296 | 0.338 | 0.425 |  |  |  |  |  |  |  |
| C | 0.001 | 0.597 | 0.219 | 0.262 | 0.295 | 0.336 | 0.426 |  |  |  |  |  |  |  |
| mean | 0.002 | 0.612 | 0.21633 | 0.2593 | 0.295 | 0.3357 | 0.4217 |  |  |  |  |  |  |  |
| % viability |  |  | 35.3486 | 42.375 | 48.203 | 54.847 | 68.9 |  |  |  |  |  |  |  |
| Ws7/MOLT-4 | | 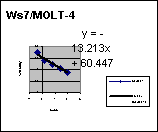 | | | |  |  |  |  | | |  |  |  |
| 2 | 35.35 |  |  |  |  |  |  |  |  |  |  |  |  |  |
| 1.4 | 42.37 |  |  |  |  |  |  |  |  | | |  |  |  |
| 0.8 | 48.2 |  |  |  |  |  |  |  |  | | |  |  |  |
| 0.19 | 54.85 |  |  |  |  |  |  |  |  | | |  |  |  |
| -0.41 | 68.9 |  |  |  |  |  |  |  |  | | |  |  |  |
|  |  |  |  |  |  |  |  |  | |  |  | |  |  |
| IC50= |  |  |  |  |  |  |  | IC50= | |  |  | |  |  |
|  |  |  |  |  |  |  |  |  | |  |  | |  |  |
|  |  |  |  |  |  |  |  |  | |  |  | |  |  |

|  | **Blank** | | | **CC** | | **Sample No. Ws5/MOLT-4** | | | | | | | | **Sample No. STA/MOLT-4** | | | | | | | | | |  |
| --- | --- | --- | --- | --- | --- | --- | --- | --- | --- | --- | --- | --- | --- | --- | --- | --- | --- | --- | --- | --- | --- | --- | --- | --- |
|  | **1** | | | **2** | | **3** | **4** | **5** | | **6** | | **7** | | **8** | | **9** | | **10** | | **11** | | **12** | |  |
| A | B | | | C | | 100ug | 25ug | 6.3ug | | 1.6ug | | 0.4ug | | 100ug | | 25ug | | 6.3ug | | 1.6ug | | 0.4ug | |  |
| B | B | | | C | | 100ug | 25ug | 6.3ug | | 1.6ug | | 0.4ug | | 100ug | | 25ug | | 6.3ug | | 1.6ug | | 0.4ug | |  |
| C | B | | | C | | 100ug | 25ug | 6.3ug | | 1.6ug | | 0.4ug | | 100ug | | 25ug | | 6.3ug | | 1.6ug | | 0.4ug | |  |
| ROBONIK P2000 eia reader | | | | | | |  |  | |  | |  | |  | |  | |  | |  | |  | |  |
| Wave length: | | | | 450 nm | |  |  |  | |  | |  | |  | |  | |  | |  | |  | |  |
| Reference: 630 nm | | | | | | |  |  | |  | |  | |  | |  | |  | |  | |  | |  |
|  | **1** | | | **2** | | **3** | **4** | **5** | | **6** | | **7** | | **8** | | **9** | | **10** | | **11** | | **12** | |  |
|  |  | | |  | |  |  |  | |  | |  | |  | |  | |  | |  | |  | |  |
| A | 0.001 | | | 0.578 | | 0.222 | 0.262 | 0.298 | | 0.339 | | 0.384 | | 0.137 | | 0.195 | | 0.294 | | 0.372 | | 0.425 | |  |
| B | 0.002 | | | 0.581 | | 0.194 | 0.255 | 0.312 | | 0.347 | | 0.389 | | 0.164 | | 0.215 | | 0.306 | | 0.355 | | 0.418 | |  |
| C | 0.001 | | | 0.599 | | 0.208 | 0.259 | 0.319 | | 0.341 | | 0.385 | | 0.129 | | 0.227 | | 0.311 | | 0.364 | | 0.433 | |  |
| mean | 0.0013 | | | 0.586 | | 0.208 | 0.259 | 0.31 | | 0.342 | | 0.386 | | 0.1433 | | 0.212 | | 0.3037 | | 0.3637 | | 0.4253 | |  |
| % viability |  | | |  | | 35.495 | 44.14 | 52.84 | | 58.42 | | 65.87 | | 24.46 | | 36.23 | | 51.82 | | 62.059 | | 72.582 | |  |
| Ws5/MOLT-4 | | | | 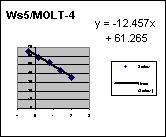 | |  |  |  | |  | |  | | STA/MOLT-4 | | | | 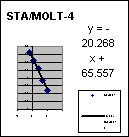 | |  | |  | |  |
| 2 | 35.495 | | |  | |  |  |  | |  | |  | | 2 | | 24.46 | |  | |  | |  | |  |
| 1.398 | 44.141 | | |  | |  |  |  | |  | |  | | 1.3979 | | 36.23 | |  | |  | |  | |  |
| 0.796 | 52.844 | | |  | |  |  |  | |  | |  | | 0.7959 | | 51.82 | |  | |  | |  | |  |
| 0.193 | 58.419 | | |  | |  |  |  | |  | |  | | 0.1931 | | 62.06 | |  | |  | |  | |  |
| -0.41 | 65.87 | | |  | |  |  |  | |  | |  | | -0.409 | | 72.58 | |  | |  | |  | |  |
|  |  | | |  | |  |  |  | |  | |  | |  | |  | |  | |  | |  | |  |
| IC50= |  | | |  | |  |  |  | |  | |  | | IC50= | |  | |  | |  | |  | |  |
|  | | **Blank** | **CC** | | **Sample No. Ws4/WI38** | | | | | | | | | | **Sample No. ws6/WI38** | | | | | | | | | |
|  | | **1** | **2** | | **3** | | **4** | | **5** | | **6** | | **7** | | **8** | | **9** | | **10** | | **11** | | **12** | |
| A | | B | C | | 100uM | | 25uM | | 6.3uM | | 1.6uM | | 0.4uM | | 100uM | | 25uM | | 6.3uM | | 1.6uM | | 0.4uM | |
| B | | B | C | | 100uM | | 25uM | | 6.3uM | | 1.6uM | | 0.4uM | | 100uM | | 25uM | | 6.3uM | | 1.6uM | | 0.4uM | |
| C | | B | C | | 100uM | | 25uM | | 6.3uM | | 1.6uM | | 0.4uM | | 100uM | | 25uM | | 6.3uM | | 1.6uM | | 0.4uM | |
| ROBONIK P2000 Eia reader | | | | | | |  | |  | |  | |  | |  | |  | |  | |  | |  | |
| Wave length: 450 nm | | | | | | |  | |  | |  | |  | |  | |  | |  | |  | |  | |
| Reference: 630 nm | | | | | | |  | |  | |  | |  | |  | |  | |  | |  | |  | |
|  | | **1** | **2** | | **3** | | **4** | | **5** | | **6** | | **7** | | **8** | | **9** | | **10** | | **11** | | **12** | |
|  | |  |  | |  | |  | |  | |  | |  | |  | |  | |  | |  | |  | |
| A | | 0.001 | 0.549 | | 0.264 | | 0.313 | | 0.354 | | 0.408 | | 0.449 | | 0.234 | | 0.287 | | 0.364 | | 0.418 | | 0.465 | |
| B | | 0.002 | 0.551 | | 0.259 | | 0.324 | | 0.359 | | 0.412 | | 0.457 | | 0.225 | | 0.322 | | 0.379 | | 0.405 | | 0.444 | |
| C | | 0.001 | 0.583 | | 0.265 | | 0.309 | | 0.356 | | 0.414 | | 0.455 | | 0.241 | | 0.315 | | 0.357 | | 0.431 | | 0.437 | |
| mean | | 0.001 | 0.561 | | 0.26267 | | 0.3153 | | 0.3563 | | 0.4113 | | 0.4537 | | 0.2333 | | 0.308 | | 0.3667 | | 0.418 | | 0.449 | |
| % viability | |  |  | | 46.8212 | | 56.209 | | 63.518 | | 73.321 | | 80.867 | | 41.592 | | 54.902 | | 65.359 | | 74.51 | | 79.98 | |
| Ws4/WI38 | | | 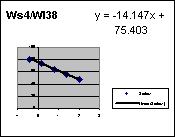 | |  | |  | |  | |  | |  | | ws6/WI38 | | | | 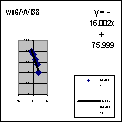 | |  | |  | |
| 2 | | 46.82 |  | |  | |  | |  | |  | |  | | 2 | | 41.5924 | |  | |  | |  | |
| 1.4 | | 56.21 |  | |  | |  | |  | |  | |  | | 1.3979 | | 54.902 | |  | |  | |  | |
| 0.8 | | 63.52 |  | |  | |  | |  | |  | |  | | 0.7959 | | 65.3595 | |  | |  | |  | |
| 0.19 | | 73.32 |  | |  | |  | |  | |  | |  | | 0.1931 | | 74.5098 | |  | |  | |  | |
| -0.41 | | 80.87 |  | |  | |  | |  | |  | |  | | -0.4089 | | 79.9762 | |  | |  | |  | |
|  | |  |  | |  | |  | |  | |  | |  | |  | |  | |  | |  | |  | |
| IC50= | |  |  | |  | |  | |  | |  | |  | | IC50= | |  | |  | |  | |  | |
|  | |  |  | |  | |  | |  | |  | |  | |  | |  | |  | |  | |  | |
|  | |  |  | |  | |  | |  | |  | |  | |  | |  | |  | |  | |  | |
|  | | **Blank** | **CC** | | **Sample No. Ws7/WI38** | | | | | | | | | | **Sample No. Ws5/WI38** | | | | | | | | | |
|  | | **1** | **2** | | **3** | | **4** | | **5** | | **6** | | **7** | | **8** | | **9** | | **10** | | **11** | | **12** | |
| A | | B | C | | 100uM | | 25uM | | 6.3uM | | 1.6uM | | 0.4uM | | 100uM | | 25uM | | 6.3uM | | 1.6uM | | 0.4uM | |
| B | | B | C | | 100uM | | 25uM | | 6.3uM | | 1.6uM | | 0.4uM | | 100uM | | 25uM | | 6.3uM | | 1.6uM | | 0.4uM | |
| C | | B | C | | 100uM | | 25uM | | 6.3uM | | 1.6uM | | 0.4uM | | 100uM | | 25uM | | 6.3uM | | 1.6uM | | 0.4uM | |
| ROBONIK P2000 Eia reader | | | | | | |  | |  | |  | |  | |  | |  | |  | |  | |  | |
| Wave length: 450 nm | | | | | | |  | |  | |  | |  | |  | |  | |  | |  | |  | |
| Reference: 630 nm | | | | | | |  | |  | |  | |  | |  | |  | |  | |  | |  | |
|  | | **1** | **2** | | **3** | | **4** | | **5** | | **6** | | **7** | | **8** | | **9** | | **10** | | **11** | | **12** | |
|  | |  |  | |  | |  | |  | |  | |  | |  | |  | |  | |  | |  | |
| A | | 0.003 | 0.538 | | 0.236 | | 0.287 | | 0.341 | | 0.388 | | 0.436 | | 0.231 | | 0.263 | | 0.322 | | 0.392 | | 0.434 | |
| B | | 0.001 | 0.543 | | 0.233 | | 0.272 | | 0.334 | | 0.373 | | 0.431 | | 0.228 | | 0.265 | | 0.319 | | 0.367 | | 0.447 | |
| C | | 0.001 | 0.529 | | 0.241 | | 0.271 | | 0.336 | | 0.369 | | 0.428 | | 0.219 | | 0.284 | | 0.341 | | 0.381 | | 0.458 | |
| mean | | 0.002 | 0.537 | | 0.23667 | | 0.2767 | | 0.337 | | 0.3767 | | 0.4317 | | 0.226 | | 0.27067 | | 0.3273 | | 0.38 | | 0.446 | |
| % viability | |  |  | | 44.0994 | | 51.553 | | 62.795 | | 70.186 | | 80.435 | | 42.112 | | 50.4348 | | 60.994 | | 70.81 | | 83.17 | |
| Ws7/WI38 | | | 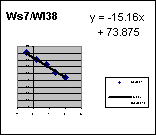 | | | | | | | |  | |  | | Ws5/WI38 | | | | 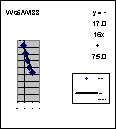  \|  \| \| --- \| | |  | |  | |
| 2 | | 44.1 |  |  |  |  |  |  |  |  |  | |  | | 2 | | 42.1118 | |  | |  | |  | |
| 1.4 | | 51.55 |  |  |  |  |  |  |  |  |  | |  | | 1.3979 | | 50.4348 | |  | |  | |  | |
| 0.8 | | 62.8 |  |  |  |  |  |  |  |  |  | |  | | 0.7959 | | 60.9938 | |  | |  | |  | |
| 0.19 | | 70.19 |  |  |  |  |  |  |  |  |  | |  | | 0.1931 | | 70.8075 | |  | |  | |  | |
| -0.41 | | 80.43 |  |  |  |  |  |  |  |  |  | |  | | -0.4089 | | 83.1677 | |  | |  | |  | |
|  | |  |  |  |  |  |  |  |  |  |  | |  | |  | |  | |  | |  | |  | |
| IC50= | |  |  |  |  |  |  |  |  |  |  | |  | | IC50= | |  | |  | |  | |  | |
|  | |  |  | |  | |  | |  | |  | |  | |  | |  | |  | |  | |  | |
|  | |  |  | |  | |  | |  | |  | |  | |  | |  | |  | |  | |  | |
|  | | **Blank** | **CC** | | **Sample No. STA/WI38** | | | | | | | | | | **Sample No.** | | | | | | | | | |
|  | | **1** | **2** | | **3** | | **4** | | **5** | | **6** | | **7** | | **8** | | **9** | | **10** | | **11** | | **12** | |
| A | | B | C | | 100uM | | 25uM | | 6.3uM | | 1.6uM | | 0.4uM | |  | |  | |  | |  | |  | |
| B | | B | C | | 100uM | | 25uM | | 6.3uM | | 1.6uM | | 0.4uM | |  | |  | |  | |  | |  | |
| C | | B | C | | 100uM | | 25uM | | 6.3uM | | 1.6uM | | 0.4uM | |  | |  | |  | |  | |  | |
| ROBONIK P2000 Eia reader | | | | | | |  | |  | |  | |  | |  | |  | |  | |  | |  | |
| Wave length: 450 nm | | | | | | |  | |  | |  | |  | |  | |  | |  | |  | |  | |
| Reference: 630 nm | | | | | | |  | |  | |  | |  | |  | |  | |  | |  | |  | |
|  | | **1** | **2** | | **3** | | **4** | | **5** | | **6** | | **7** | | **8** | | **9** | | **10** | | **11** | | **12** | |
|  | |  |  | |  | |  | |  | |  | |  | |  | |  | |  | |  | |  | |
| A | | 0.001 | 0.477 | | 0.181 | | 0.226 | | 0.264 | | 0.319 | | 0.388 | |  | |  | |  | |  | |  | |
| B | | 0.003 | 0.483 | | 0.177 | | 0.221 | | 0.271 | | 0.319 | | 0.404 | |  | |  | |  | |  | |  | |
| C | | 0.001 | 0.464 | | 0.175 | | 0.219 | | 0.265 | | 0.3317 | | 0.412 | |  | |  | |  | |  | |  | |
| mean | | 0.002 | 0.475 | | 0.17767 | | 0.222 | | 0.2667 | | 0.3232 | | 0.4013 | | 0 | | 0 | | 0 | | 0 | | 0 | |
| % | |  | ` | | 37.4298 | | 46.77 | | 56.18 | | 68.097 | | 84.551 | | 0 | | 0 | | 0 | | 0 | | 0 | |
| STA/WI38 | |  |  | | 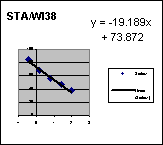 | | | | | | | |  | |  | |  | | 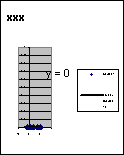 | |  | |  | |
| log conc. | | % viability |  | |  |  |  |  |  |  |  |  |  | | log conc. | | % viability | |  | |  | |  | |
| 2 | | 37.43 |  | |  |  |  |  |  |  |  |  |  | | 2 | | 0 | |  | |  | |  | |
| 1.4 | | 46.77 |  | |  |  |  |  |  |  |  |  |  | | 1.3979 | | 0 | |  | |  | |  | |
| 0.8 | | 56.18 |  | |  |  |  |  |  |  |  |  |  | | 0.7959 | | 0 | |  | |  | |  | |
| 0.19 | | 68.1 |  | |  |  |  |  |  |  |  |  |  | | 0.1931 | | 0 | |  | |  | |  | |
| -0.41 | | 84.55 |  | |  |  |  |  |  |  |  |  |  | | -0.4089 | | 0 | |  | |  | |  | |
|  | |  |  | |  |  |  |  |  |  |  |  |  | |  | |  | |  | |  | |  | |
| IC50= | |  |  | |  |  |  |  |  |  |  |  |  | | IC50= | |  | |  | |  | |  | |
|  | |  |  | |  | |  | |  | |  | |  | |  | |  | |  | |  | |  | |
|  | |  |  | |  | |  | |  | |  | |  | |  | |  | |  | |  | |  | |

|  |  |  |  |  |  |  |  |  |  | |  | |  | |  |
| --- | --- | --- | --- | --- | --- | --- | --- | --- | --- | --- | --- | --- | --- | --- | --- |
|  | **Blank** | **CC** | **Sample No. Ws7/T-47D** | | | | |  |  |  |  |  |  |  |  |
|  | **1** | **2** | **3** | **4** | **5** | **6** | **7** |  |  |  |  |  |  |  |  |
| A | B | C | 100uM | 25uM | 6.3uM | 1.6uM | 0.4uM |  |  |  |  |  |  |  |  |
| B | B | C | 100uM | 25uM | 6.3uM | 1.6uM | 0.4uM |  |  |  |  |  |  |  |  |
| C | B | C | 100uM | 25uM | 6.3uM | 1.6uM | 0.4uM |  |  |  |  |  |  |  |  |
| ROBONIK P2000 Eia reader | | | |  |  |  |  |  |  | |  | |  | |  |
| Wave length: 450 nm | | | |  |  |  |  |  |  | |  | |  | |  |
| Reference: 630 nm | | | |  |  |  |  |  |  | |  | |  | |  |
|  | **1** | **2** | **3** | **4** | **5** | **6** | **7** |  |  |  |  |  |  |  |  |
|  |  |  |  |  |  |  |  |  |  |  |  |  |  |  |  |
| A | 0.001 | 0.575 | 0.185 | 0.256 | 0.308 | 0.345 | 0.388 |  |  |  |  |  |  |  |  |
| B | 0.001 | 0.564 | 0.179 | 0.249 | 0.311 | 0.349 | 0.386 |  |  |  |  |  |  |  |  |
| C | 0.001 | 0.559 | 0.213 | 0.252 | 0.315 | 0.353 | 0.389 |  |  |  |  |  |  |  |  |
| mean | 0.001 | 0.566 | 0.19233 | 0.2523 | 0.3113 | 0.349 | 0.3877 |  |  |  |  |  |  |  |  |
| % viability |  |  | 33.9812 | 44.582 | 55.006 | 61.661 | 68.492 |  |  |  |  |  |  |  |  |
| Ws7/T-47D | | 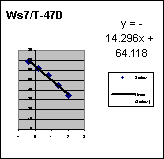 |  |  |  |  |  |  | |  | |  | |  |  |
| log conc. | % viability |  |  |  |  |  |  |  | |  | |  | |  |  |
| 2 | 33.98 |  |  |  |  |  |  |  | |  | |  | |  |  |
| 1.4 | 44.58 |  |  |  |  |  |  |  | |  | |  | |  |  |
| 0.8 | 55.01 |  |  |  |  |  |  |  | |  | |  | |  |  |
| 0.19 | 61.66 |  |  |  |  |  |  |  | |  | |  | |  |  |
| -0.41 | 68.49 |  |  |  |  |  |  |  | |  | |  | |  |  |
|  |  |  |  |  |  |  |  |  |  | |  | |  | |  |

|  | **Blank** | **CC** | **Sample No. Ws5/T47D** | | | | | **Sample No. STA/T47D** | | | | |
| --- | --- | --- | --- | --- | --- | --- | --- | --- | --- | --- | --- | --- |
|  | **1** | **2** | **3** | **4** | **5** | **6** | **7** | **8** | **9** | **10** | **11** | **12** |
| A | B | C | 100uM | 25uM | 6.3uM | 1.6ug | 0.4uM | 100uM | 25uM | 6.3uM | 1.6ug | 0.4uM |
| B | B | C | 100uM | 25uM | 6.3uM | 1.6ug | 0.4uM | 100uM | 25uM | 6.3uM | 1.6ug | 0.4uM |
| C | B | C | 100uM | 25uM | 6.3uM | 1.6ug | 0.4uM | 100uM | 25uM | 6.3uM | 1.6ug | 0.4uM |
| ROBONIK P2000 eia reader | | | |  |  |  |  |  |  |  |  |  |
| Wave length: 450 nm | | | |  |  |  |  |  |  |  |  |  |
| Reference: 630 nm | | | |  |  |  |  |  |  |  |  |  |
|  | **1** | **2** | **3** | **4** | **5** | **6** | **7** | **8** | **9** | **10** | **11** | **12** |
|  |  |  |  |  |  |  |  |  |  |  |  |  |
| A | 0.001 | 0.543 | 0.176 | 0.231 | 0.281 | 0.332 | 0.405 | 0.159 | 0.191 | 0.252 | 0.333 | 0.377 |
| B | 0.003 | 0.528 | 0.163 | 0.222 | 0.277 | 0.328 | 0.393 | 0.167 | 0.182 | 0.246 | 0.316 | 0.365 |
| C | 0.001 | 0.531 | 0.175 | 0.221 | 0.276 | 0.331 | 0.387 | 0.149 | 0.188 | 0.237 | 0.337 | 0.364 |
| mean | 0.0017 | 0.534 | 0.1713 | 0.225 | 0.278 | 0.33 | 0.395 | 0.1583 | 0.187 | 0.245 | 0.3287 | 0.3687 |
| % viability |  |  | 32.085 | 42.07 | 52.06 | 61.86 | 73.97 | 29.65 | 35.02 | 45.88 | 61.548 | 69.039 |
| Ws5/T47D | | 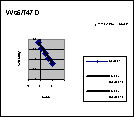 | | | |  |  | STA/T47D | | 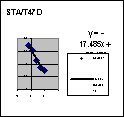 | | |
| 2 | 32.085 |  |  |  |  |  |  | 2 | 29.65 |  |  |  |
| 1.398 | 42.072 |  |  |  |  |  |  | 1.3979 | 35.02 |  |  |  |
| 0.796 | 52.06 |  |  |  |  |  |  | 0.7959 | 45.88 |  |  |  |
| 0.193 | 61.86 |  |  |  |  |  |  | 0.1931 | 61.55 |  |  |  |
| -0.41 | 73.97 |  |  |  |  |  |  | -0.409 | 69.04 |  |  |  |
|  |  |  |  |  |  |  |  |  |  |  |  |  |
| IC50= |  |  |  |  |  |  |  | IC50= |  |  |  |  |
|  |  |  |  |  |  |  |  |  |  |  |  |  |
|  |  |  |  |  |  |  |  |  |  |  |  |  |
|  | **Blank** | **CC** | **Sample No. Ws5/MDA-MB-468** | | | | | **Sample No. STA/MDA-MB-468** | | | | |
|  | **1** | **2** | **3** | **4** | **5** | **6** | **7** | **8** | **9** | **10** | **11** | **12** |
| A | B | C | 100uM | 25uM | 6.3uM | 1.6ug | 0.4uM | 100uM | 25uM | 6.3uM | 1.6ug | 0.4uM |
| B | B | C | 100uM | 25uM | 6.3uM | 1.6ug | 0.4uM | 100uM | 25uM | 6.3uM | 1.6ug | 0.4uM |
| C | B | C | 100uM | 25uM | 6.3uM | 1.6ug | 0.4uM | 100uM | 25uM | 6.3uM | 1.6ug | 0.4uM |

**EGFR inhibitor screening**

**Researcher** : Dr.Mohamed Hawas email: [mohhawwas80@gmail.com](mailto:mohhawwas80@gmail.com)  **mob.** 01151345909

Assay : EGFR inhibitor screening [mohamedhassan.pharm.ast@azhr.edu.eg](mailto:mohamedhassan.pharm.ast@azhr.edu.eg)

**Samples** : 04 compounds

Cell lines : ---

**Ref**. : ----

Date : 03-12-2023

**Reader** : Tecan Spark

Kit used : ---

**Solvent** : DMSO

**Lab Report**

| **ser** | **Compound** | | | **EGFR** | **SD±** |
| --- | --- | --- | --- | --- | --- |
|  | **code** | **MW** | **conc.**  **uM** | **IC50**  **uM** |  |
| 1 | **WS4** | **470** | --- | **0.156** | 0.006 |
| 2 | **WS5** | **515** | --- | **0.055** | 0.002 |
| 3 | **WS6** | **401** | --- | **0.64** | 0.023 |
| 4 | **WS7** | **415** | --- | **0.194** | 0.007 |
| 5 | **Erlotinib** | **393.44** | --- | **0.06** | 0.002 |

**Detailed results**

| **EGFR** |  |  |  |  |  |  |  |  |  |  |  |  |
| --- | --- | --- | --- | --- | --- | --- | --- | --- | --- | --- | --- | --- |
| code | IC50 | conc | log | %inh | T2 | T1 | ∆T | RFU2 | RFU1 | ∆RFU | slope | K.Activity |
| Ws4 |  | 100 | 2 | 93.7 | 30 | 0 | 30 | 6.26 | 0 | 6.26 | 3.333 | 7.512 |
| 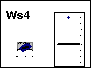 |  | 10 | 1 | 86.9 | 30 | 0 | 30 | 13.09 | 0 | 13.09 | 3.333 | 15.708 |
|  |  | 1 | 0 | 72.7 | 30 | 0 | 30 | 27.33 | 0 | 27.33 | 3.333 | 32.796 |
|  |  | 0.1 | -1 | 40.6 | 30 | 0 | 30 | 59.41 | 0 | 59.41 | 3.333 | 71.292 |
|  |  | 0.01 | -2 | 27.8 | 30 | 0 | 30 | 72.15 | 0 | 72.15 | 3.333 | 86.58 |
| EC |  |  |  | 0 | 30 | 0 | 30 | 100 | 0 | 100 | 3.333 | 120 |
|  |  |  |  |  |  |  |  |  |  |  |  |  |
| code | IC50 | conc | log | %inh | T2 | T1 | ∆T | RFU2 | RFU1 | ∆RFU | slope | K.Activity |
| Ws5 |  | 100 | 2 | 94.2 | 30 | 0 | 30 | 5.84 | 0 | 5.84 | 3.333 | 7.008 |
| 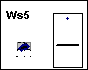 |  | 10 | 1 | 88.1 | 30 | 0 | 30 | 11.92 | 0 | 11.92 | 3.333 | 14.304 |
|  |  | 1 | 0 | 75.8 | 30 | 0 | 30 | 24.19 | 0 | 24.19 | 3.333 | 29.028 |
|  |  | 0.1 | -1 | 50.6 | 30 | 0 | 30 | 49.35 | 0 | 49.35 | 3.333 | 59.22 |
|  |  | 0.01 | -2 | 37 | 30 | 0 | 30 | 62.99 | 0 | 62.99 | 3.333 | 75.588 |
| EC |  |  |  | 0 | 30 | 0 | 30 | 100 | 0 | 100 | 3.333 | 120 |
|  |  |  |  |  |  |  |  |  |  |  |  |  |
| code | IC50 | conc | log | %inh | T2 | T1 | ∆T | RFU2 | RFU1 | ∆RFU | slope | K.Activity |
| Ws6 |  | 100 | 2 | 92.1 | 30 | 0 | 30 | 7.86 | 0 | 7.86 | 3.333 | 9.432 |
| 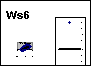 |  | 10 | 1 | 80.6 | 30 | 0 | 30 | 19.41 | 0 | 19.41 | 3.333 | 23.292 |
|  |  | 1 | 0 | 61.8 | 30 | 0 | 30 | 38.22 | 0 | 38.22 | 3.333 | 45.864 |
|  |  | 0.1 | -1 | 26 | 30 | 0 | 30 | 74.03 | 0 | 74.03 | 3.333 | 88.836 |
|  |  | 0.01 | -2 | 10.6 | 30 | 0 | 30 | 89.39 | 0 | 89.39 | 3.333 | 107.27 |
| EC |  |  |  | 0 | 30 | 0 | 30 | 100 | 0 | 100 | 3.333 | 120 |
|  |  |  |  |  |  |  |  |  |  |  |  |  |
| code | IC50 | conc | log | %inh | T2 | T1 | ∆T | RFU2 | RFU1 | ∆RFU | slope | K.Activity |
| Ws7 |  | 100 | 2 | 94.5 | 30 | 0 | 30 | 5.52 | 0 | 5.52 | 3.333 | 6.624 |
| 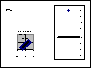 |  | 10 | 1 | 86.3 | 30 | 0 | 30 | 13.71 | 0 | 13.71 | 3.333 | 16.452 |
|  |  | 1 | 0 | 71.4 | 30 | 0 | 30 | 28.55 | 0 | 28.55 | 3.333 | 34.26 |
|  |  | 0.1 | -1 | 44.2 | 30 | 0 | 30 | 55.79 | 0 | 55.79 | 3.333 | 66.948 |
|  |  | 0.01 | -2 | 21 | 30 | 0 | 30 | 79.04 | 0 | 79.04 | 3.333 | 94.848 |
| EC |  |  |  | 0 | 30 | 0 | 30 | 100 | 0 | 100 | 3.333 | 120 |
|  |  |  |  |  |  |  |  |  |  |  |  |  |
| code | IC50 | conc | log | %inh | T2 | T1 | ∆T | RFU2 | RFU1 | ∆RFU | slope | K.Activity |
| Erlotenib |  | 100 | 2 | 95.7 | 30 | 0 | 30 | 4.29 | 0 | 4.29 | 3.333 | 5.148 |
| 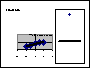 |  | 10 | 1 | 88.9 | 30 | 0 | 30 | 11.08 | 0 | 11.08 | 3.333 | 13.296 |
|  |  | 1 | 0 | 76.4 | 30 | 0 | 30 | 23.55 | 0 | 23.55 | 3.333 | 28.26 |
|  |  | 0.1 | -1 | 51.8 | 30 | 0 | 30 | 48.19 | 0 | 48.19 | 3.333 | 57.828 |
|  |  | 0.01 | -2 | 34.5 | 30 | 0 | 30 | 65.46 | 0 | 65.46 | 3.333 | 78.552 |
| EC |  |  |  | 0 | 30 | 0 | 30 | 100 | 0 | 100 | 3.333 | 120 |
|  |  |  |  |  |  |  |  |  |  |  |  |  |

| 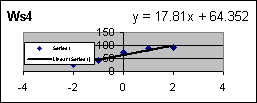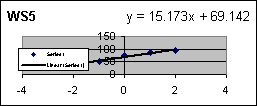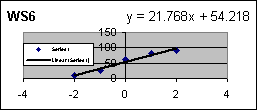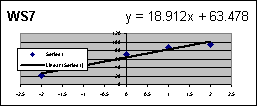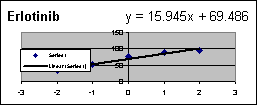 |  |  |  |
| --- | --- | --- | --- |
|  |  |  |  |
|  |  |  |  |
|  |  |  |  |
|  |  |  |  |
|  |  |  |  |
|  |  |  |  |
|  |  |  |  |
|  |  |  |  |
|  |  |  |  |
|  |  |  |  |
|  |  |  |  |
|  |  |  |  |

**B-Raf (V600E)Kinase Assay**

**Researcher** : Dr.Mohamed Hawas email: [mohhawwas80@gmail.com](mailto:mohhawwas80@gmail.com)  **mob.** 01151345909

Assay : B-Raf (V600E)Kinase Assay [mohamedhassan.pharm.ast@azhr.edu.eg](mailto:mohamedhassan.pharm.ast@azhr.edu.eg)

Samples : 04 samples .

Reference : ---

Cell lines : ---

Kit used : ---

Solvent : DMSO

Assay samples :

**Lab Report**

| ser | compound | | | **B-Raf(V600E)** | SD  ± |
| --- | --- | --- | --- | --- | --- |
|  | code | M.W | conc | IC50  uM |  |
| **1** | **WS4** | **470** | --- | **0.249** | 0.01 |
| **2** | **WS5** | **515** | --- | **0.068** | 0.003 |
| **3** | **WS6** | **401** | --- | **0.410** | 0.016 |
| **4** | **WS7** | **415** | --- | **0.194** | 0.008 |
| ******* | **Vemurafenib** | 489.92 | --- | **0.035** | 0.001 |

**Detailed results:**

| **B-raf (V600E)** | | | | | | | | | | | |  |
| --- | --- | --- | --- | --- | --- | --- | --- | --- | --- | --- | --- | --- |
| code | IC50 | conc | log | %inh | T2 | T1 | ∆T | RFU2 | RFU1 | ∆RFU | slope | K.Activity |
| WS4 |  | 100 | 2 | 93 | 30 | 0 | 30 | 7.29 | 0 | 7.29 | 3.3333 | 8.74809 |
|  |  | 10 | 1 | 84 | 30 | 0 | 30 | 16.03 | 0 | 16.03 | 3.3333 | 19.2362 |
| 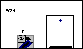 |  | 1 | 0 | 64 | 30 | 0 | 30 | 35.88 | 0 | 35.88 | 3.3333 | 43.0564 |
|  |  | 0.1 | -1 | 41 | 30 | 0 | 30 | 59.37 | 0 | 59.37 | 3.3333 | 71.2447 |
|  |  | 0.01 | -2 | 23 | 30 | 0 | 30 | 76.55 | 0 | 76.55 | 3.3333 | 91.8609 |
| EC |  |  |  | 0 | 30 | 0 | 30 | 100 | 0 | 100 | 3.3333333 | 120 |
|  |  |  |  |  |  |  |  |  |  |  |  |  |
| code | IC50 | conc | log | %inh | T2 | T1 | ∆T | RFU2 | RFU1 | ∆RFU | slope | K.Activity |
| WS5 |  | 100 | 2 | 94 | 30 | 0 | 30 | 5.72 | 0 | 5.72 | 3.3333 | 6.86407 |
| 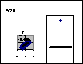 |  | 10 | 1 | 88 | 30 | 0 | 30 | 12.41 | 0 | 12.41 | 3.3333 | 14.8921 |
|  |  | 1 | 0 | 71 | 30 | 0 | 30 | 29.13 | 0 | 29.13 | 3.3333 | 34.9563 |
|  |  | 0.1 | -1 | 52 | 30 | 0 | 30 | 47.68 | 0 | 47.68 | 3.3333 | 57.2166 |
|  |  | 0.01 | -2 | 35 | 30 | 0 | 30 | 65.33 | 0 | 65.33 | 3.3333 | 78.3968 |
| EC |  |  |  | 0 | 30 | 0 | 30 | 100 | 0 | 100 | 3.3333333 | 120 |
|  |  |  |  |  |  |  |  |  |  |  |  |  |
| code | IC50 | conc | log | %inh | T2 | T1 | ∆T | RFU2 | RFU1 | ∆RFU | slope | K.Activity |
| WS6 |  | 100 | 2 | 91 | 30 | 0 | 30 | 9.03 | 0 | 9.03 | 3.3333 | 10.8361 |
|  |  | 10 | 1 | 81 | 30 | 0 | 30 | 19.35 | 0 | 19.35 | 3.3333 | 23.2202 |
| 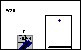 |  | 1 | 0 | 63 | 30 | 0 | 30 | 37.22 | 0 | 37.22 | 3.3333 | 44.6644 |
|  |  | 0.1 | -1 | 35 | 30 | 0 | 30 | 64.93 | 0 | 64.93 | 3.3333 | 77.9168 |
|  |  | 0.01 | -2 | 18 | 30 | 0 | 30 | 82.31 | 0 | 82.31 | 3.3333 | 98.773 |
| EC |  |  |  | 0 | 30 | 0 | 30 | 100 | 0 | 100 | 3.3333333 | 120 |
|  |  |  |  |  |  |  |  |  |  |  |  |  |
| code | IC50 | conc | log | %inh | T2 | T1 | ∆T | RFU2 | RFU1 | ∆RFU | slope | K.Activity |
| WS7 |  | 100 | 2 | 94 | 30 | 0 | 30 | 6.12 | 0 | 6.12 | 3.3333 | 7.34407 |
| 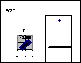 |  | 10 | 1 | 86 | 30 | 0 | 30 | 14.39 | 0 | 14.39 | 3.3333 | 17.2682 |
|  |  | 1 | 0 | 70 | 30 | 0 | 30 | 29.59 | 0 | 29.59 | 3.3333 | 35.5084 |
|  |  | 0.1 | -1 | 42 | 30 | 0 | 30 | 58.24 | 0 | 58.24 | 3.3333 | 69.8887 |
|  |  | 0.01 | -2 | 24 | 30 | 0 | 30 | 76.19 | 0 | 76.19 | 3.3333 | 91.4289 |
| EC |  |  |  | 0 | 30 | 0 | 30 | 100 | 0 | 100 | 3.3333333 | 120 |
|  |  |  |  |  |  |  |  |  |  |  |  |  |
| code | IC50 | conc | log | %inh | T2 | T1 | ∆T | RFU2 | RFU1 | ∆RFU | slope | K.Activity |
| Vemurafenib |  | 100 | 2 | 95 | 30 | 0 | 30 | 5.41 | 0 | 5.41 | 3.3333 | 6.49206 |
| 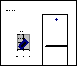 |  | 10 | 1 | 88 | 30 | 0 | 30 | 11.63 | 0 | 11.63 | 3.3333 | 13.9561 |
|  |  | 1 | 0 | 75 | 30 | 0 | 30 | 25.02 | 0 | 25.02 | 3.3333 | 30.0243 |
|  |  | 0.1 | -1 | 61 | 30 | 0 | 30 | 38.69 | 0 | 38.69 | 3.3333 | 46.4285 |
|  |  | 0.01 | -2 | 36 | 30 | 0 | 30 | 64.43 | 0 | 64.43 | 3.3333 | 77.3168 |
| EC |  |  |  | 0 | 30 | 0 | 30 | 100 | 0 | 100 | 3.3333333 | 120 |
|  |  |  |  |  |  |  |  |  |  |  |  |  |

| 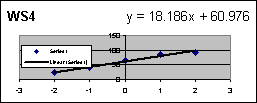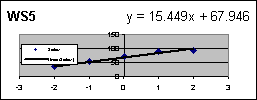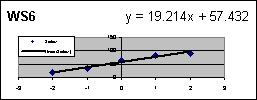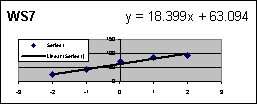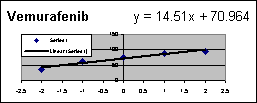 |  |  |  |
| --- | --- | --- | --- |
|  |  |  |  |
|  |  |  |  |
|  |  |  |  |
|  |  |  |  |
|  |  |  |  |
|  |  |  |  |

**Cell Cycle Analysis**

**Researcher** : Dr.Mohamed Hawas email: [mohhawwas80@gmail.com](mailto:mohhawwas80@gmail.com) mob. 01151345909

Assay : Cell Cycle Analysis [mohamedhassan.pharm.ast@azhr.edu.eg](mailto:mohamedhassan.pharm.ast@azhr.edu.eg)

Samples : 02 samples

cell line : ---

Ref. : ---

Date : 00-00-2022

Reader : BD FACSCalibur

Kit used : ab139418_Propidium Iodide Flow Cytometry Kit/BD

Solvent : DMSO

Assay samples : Cell culture

**Lab Report**

| **ser** | **Sample** | | **DNA content** | | | |  |
| --- | --- | --- | --- | --- | --- | --- | --- |
|  | **code** | **IC50**  **uM** | **%G0-G1** | **%S** | **%G2/M** | **Comment** |  |
| 1 | **Ws5/HOP92** | --- | 54.11 | 31.29 | 14.6 | cell growth arrest@ G1 |  |
| 2 | **Cont. HOP92** | --- | 48.26 | 35.66 | 16.08 | --- |  |

| **s** | **code** | **conc** | **Apoptosis** | | | **Necrosis** |
| --- | --- | --- | --- | --- | --- | --- |
|  |  |  | Total | Early | Late |  |
| 1 | **Ws5/HOP92** | --- | **37.59** | **22.91** | **11.28** | **3.4** |
| 2 | **Cont. HOP92** | --- | **2.14** | **0.69** | **0.15** | **1.3** |

**Detailed results**


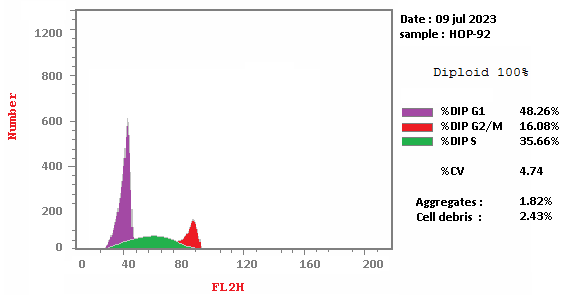


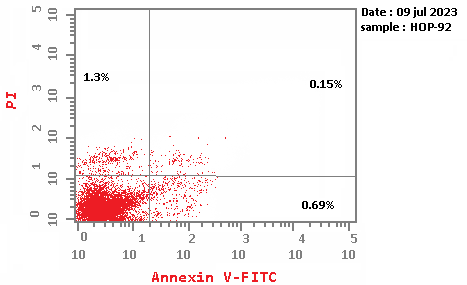


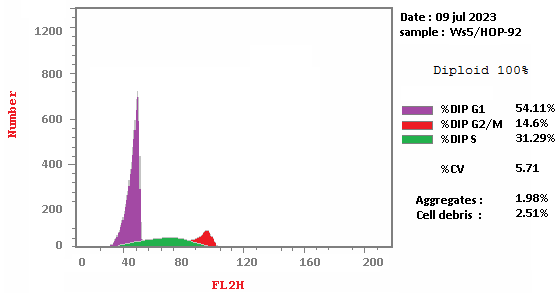


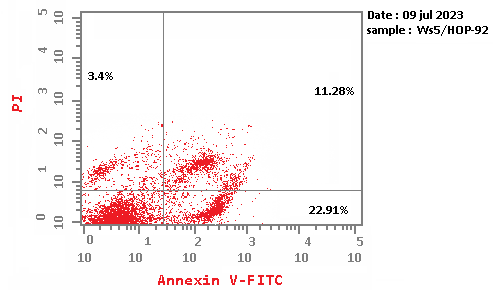


**Researcher** : Dr.Mohamed Hawas email: [mohhawwas80@gmail.com](mailto:mohhawwas80@gmail.com) mob. 01151345909

Assay : Cell Cycle Analysis [mohamedhassan.pharm.ast@azhr.edu.eg](mailto:mohamedhassan.pharm.ast@azhr.edu.eg)

**Assay** : RT-PCR

**Samples** : 02 Samples

**Cell** **lines** : ----

**Ref**. : ----

**Date** : 12-06-2023

**Reader** : Rotorgene RT- PCR system

Kit used : Qiagen RNA extraction/BioRad syber green PCR MMX

**Solvent** : DMSO

**Assay samples** : Cell Lysate

**Lab Report**

| **Ser** | **Sample** | | | **RT-PCR**  **Fold Change** | | | **SD** |
| --- | --- | --- | --- | --- | --- | --- | --- |
|  | **code** | **cells** | **IC50** | **FLD** | | |  |
|  |  |  |  | **Casp3** | **Casp9** | **Bcl2** |  |
| **1** | **Ws5/HOP-92** | **---** | **---** | **4.716** | **3.082** | **0.299** |  |
| **2** | **Staurosporine/HOP-92** | **---** | **---** | **6.906** | **5.514** | **0.326** |  |
| **3** | **Cont.HOP-92** | **---** | **---** | **1** | **1** | **1** |  |

Detailed results


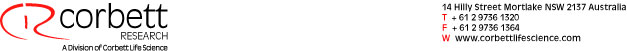


**Quantitation Report**

Experiment Information

| Run Name | Run 2023-07-09 (1) |
| --- | --- |
| Run Start | 2023-07-09 01:46:17 PM |
| Run Finish | 2023-07-09 05:15:04 PM |
| Operator | ERA |
| Notes | --- |
| Run On Software Version | Rotor-Gene 1.7.87 |
| Run Signature | The Run Signature is valid. |
| Gain Green | 10. |
| Gain Yellow | 9.33 |

Quantitation data

| 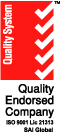 | This report generated by Rotor-Gene 6000 Series Software 1.7 (Build 87) Copyright 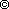2000-2006 Corbett Research, a Division of Corbett Life Science. All rights reserved. ISO 9001:2000 (Reg. No. QEC21313)  Primers  primers  Casp3 : F 5’- GGAAGCGAATCAATGGACTCTGG -3’,  Casp3 : R 5'- GCATCGACATCTGTACCAGACC -3'.  Casp9 : F 5’- GTTTGAGGACCTTCGACCAGCT -3’,  Casp9 : R 5'- CAACGTACCAGGAGCCACTCTT -3'.  Bcl2 : F 5’- ATCGCCCTGTGGATGACTGAGT -3’,  Bcl2 : R 5'- GCCAGGAGAAATCAAACAGAGGC -3'.  GAPDH : F 5’- GTCTCCTCTGACTTCAACAGCG-3’  GAPDH : R 5’- ACCACCCTGTTGCTGTAGCCAA-3’ |
| --- | --- |

|  |  |  |  |  |  |  |  |  |  |  |
| --- | --- | --- | --- | --- | --- | --- | --- | --- | --- | --- |
| **Sample** | | | **Gene Expression** | | | | | | | |
|  |  |  |  |  |  |  |  |  |  |  |
|  |  |  | Control cells | | | Test cells | | | | FLD |
|  |  |  |  |  |  |  |  |  |  |  |
| **Ser** | **code** | **Conc** | **GAPDH** | **Casp3** | ΔCTC | **GAPDH** | **Casp3** | ΔCTE | ΔΔ CT | 2^ ΔΔCT |
|  |  |  | HC | TC | TC-HC | HE | TE | TE-HE | ΔCTE-ΔCTC | E=1.869 |
| **1** | **Ws5/HOP-92** |  | **22.06** | **34.18** | 12.12 | **21.93** | **31.57** | 9.64 | **-2.48** | 4.7162 |
| **2** | **Staurosporine/HOP-92** |  | **22.06** | **34.18** | 12.12 | **22.25** | **31.28** | 9.03 | **-3.09** | 6.9067 |
| **3** | **Cont.HOP-92** |  | **22.06** | **34.18** | 12.12 | **22.06** | **34.18** | 12.12 | **0** | 1 |
|  |  |  |  |  |  |  |  |  |  |  |
| **Ser** | **code** | **Conc** | **GAPDH** | **Casp9** | ΔCTC | **GAPDH** | **Casp9** | ΔCTE | ΔΔ CT | 2^ ΔΔCT |
|  |  |  | HC | TC | TC-HC | HE | TE | TE-HE | ΔCTE-ΔCTC | E=1.869 |
| **1** | **Ws5/HOP-92** |  | **22.06** | **32.69** | 10.63 | **21.93** | **30.76** | 8.83 | **-1.8** | 3.0825 |
| **2** | **Staurosporine/HOP-92** |  | **22.06** | **32.69** | 10.63 | **22.25** | **30.15** | 7.9 | **-2.73** | 5.5143 |
| **3** | **Cont.HOP-92** |  | **22.06** | **32.69** | 10.63 | **22.06** | **32.69** | 10.63 | **0** | 1 |
|  |  |  |  |  |  |  |  |  |  |  |
| **Ser** | **code** | **Conc** | **GAPDH** | **bcl2** | ΔCTC | **GAPDH** | **bcl2** | ΔCTE | ΔΔ CT | 2^ ΔΔCT |
|  |  |  | HC | TC | TC-HC | HE | TE | TE-HE | ΔCTE-ΔCTC | E=1.869 |
| **1** | **Ws5/HOP-92** |  | **22.06** | **26.81** | 4.75 | **21.93** | **28.61** | 6.68 | **1.93** | 0.2991 |
| **2** | **Staurosporine/HOP-92** |  | **22.06** | **26.81** | 4.75 | **22.25** | **28.79** | 6.54 | **1.79** | 0.3265 |
| **3** | **Cont.HOP-92** |  | **22.06** | **26.81** | 4.75 | **22.06** | **26.81** | 4.75 | **0** | 1 |
|  |  |  |  |  |  |  |  |  |  |  |
