# Supplementary material for: An innovative approach to development of new pyrazolylquinolin-2-one hybrids as dual EGFR and BRAFV600E inhibitors
Source: Mol Divers. 2025 Mar 8;29(6):6379–400. doi: 10.1007/s11030-025-11127-4 (PMC12638379; doi:10.1007/s11030-025-11127-4)
Supplement: Supplementary file 2 — Supplementary file2 (DOCX 32920 KB) [file 11030_2025_11127_MOESM2_ESM.docx]

**Supporting Information**

**An innovative approach to development of new pyrazolylquinoline-2-one hybrids as dual EGFR and BRAF^V600E^ inhibitors**

Mohamed M. Hawwas^a^, Ahmed S. Mancy^b,c^, Mohamed Ramadan^a^, Tarek S. Ibrahim^d,e^, Ashraf H Bayoumi^f^, Mohamed Alswah^f^

*^a^ Department of Pharmaceutical Organic Chemistry, Faculty of Pharmacy, Al-Azhar University, Assiut, Egypt.*

*^b^ Department of Pharmacology and Experimental Neuroscience, University of Nebraska Medical Center, Omaha, NE 68198, USA.*

*^c^ Department of Pharmaceutical Sciences, College of Pharmacy, University of Nebraska Medical Center, Omaha, Nebraska, USA*

*^d^ Department of Pharmaceutical Chemistry, Faculty of Pharmacy, King Abdulaziz University, Jeddah, 21589, Saudi Arabia.*

*^e^ Department of Pharmaceutical Organic Chemistry, Faculty of Pharmacy, Zagazig University, Zagazig 44519, Egypt.*

*^f^ Department of Pharmaceutical Organic Chemistry, Faculty of Pharmacy, Al-Azhar University, Cairo, Egypt.*

**4. Experimental**

**4.1. Chemistry**

All commercial reagents and solvents are available and purchased from generic chemical companies and used as they were without purification. Melting points were determined on a Thomas-Hoover capillary apparatus and were uncorrected. ^1^H NMR and ^13^C NMR spectra were measured on a Bruker Avance III 400 MHz (BrukerBioSpin AG, Fallanden, Switzerland) for ^1^H and 100 MHz for ^13^C, Faculty of Pharmacy, Zagazig University, Egypt, DMSO–d_6_ as solvent, TMS as the internal standard, where *J* (coupling constant) values are estimated in Hertz (Hz) and chemical shifts were recorded in ppm on δ scale. Elemental analyses were determined by the Regional Center for Mycology and Biotechnology (RCMB), Al Azhar University, Egypt, utilizing FLASH 2000 CHNS/O analyzer, Thermo Scientific. HRMS-ESI were recorded at Research School of Chemistry, the Australian National University at Canberra, Australia**.**

^1^H NMR (400 MHz, DMSO*d_6_*) for compound 2a

^13^C NMR (100 MHz, DMSO *d_6_*) for compound 2a

^1^H NMR (400 MHz, DMSO*d_6_*) for compound 2b

^13^C NMR (100 MHz, DMSO *d_6_*) for compound 2b

^1^H NMR (400 MHz, DMSO*d_6_*) for compound 2c

^13^C NMR (100 MHz, DMSO *d_6_*) for compound 2c

^1^H NMR (400 MHz, DMSO*d_6_*) for compound 2d

^13^C NMR (100 MHz, DMSO *d_6_*) for compound 2d

^1^H NMR (400 MHz, DMSO*d_6_*) for compound 2e

^13^C NMR (100 MHz, DMSO *d_6_*) for compound 2e

^1^H NMR (400 MHz, DMSO*d_6_*) for compound 4a

^13^C NMR (100 MHz, DMSO *d_6_*) for compound 4a

^1^H NMR (400 MHz, DMSO*d_6_*) for compound 4b

^13^C NMR (100 MHz, DMSO *d_6_*) for compound 4b

^1^H NMR (400 MHz, DMSO*d_6_*) for compound 4c

^13^C NMR (100 MHz, DMSO *d_6_*) for compound 4c

^1^H NMR (400 MHz, DMSO*d_6_*) for compound 4d

^13^C NMR (100 MHz, DMSO *d_6_*) for compound 4d

^1^H NMR (400 MHz, DMSO*d_6_*) for compound 4e

^13^C NMR (100 MHz, DMSO *d_6_*) for compound 4e

^1^H NMR (400 MHz, DMSO*d_6_*) for compound 4f

^13^C NMR (100 MHz, DMSO *d_6_*) for compound 4f

^1^H NMR (400 MHz, DMSO*d_6_*) for compound 4g

^13^C NMR (100 MHz, DMSO *d_6_*) for compound 4g

^1^H NMR (400 MHz, DMSO*d_6_*) for compound 4h

^13^C NMR (100 MHz, DMSO *d_6_*) for compound 4h

^1^H NMR (400 MHz, DMSO*d_6_*) for compound 4i

^13^C NMR (100 MHz, DMSO *d_6_*) for compound 4i

^1^H NMR (400 MHz, DMSO*d_6_*) for compound 4j

^13^C NMR (100 MHz, DMSO *d_6_*) for compound 4j

HRMS for compound 4a


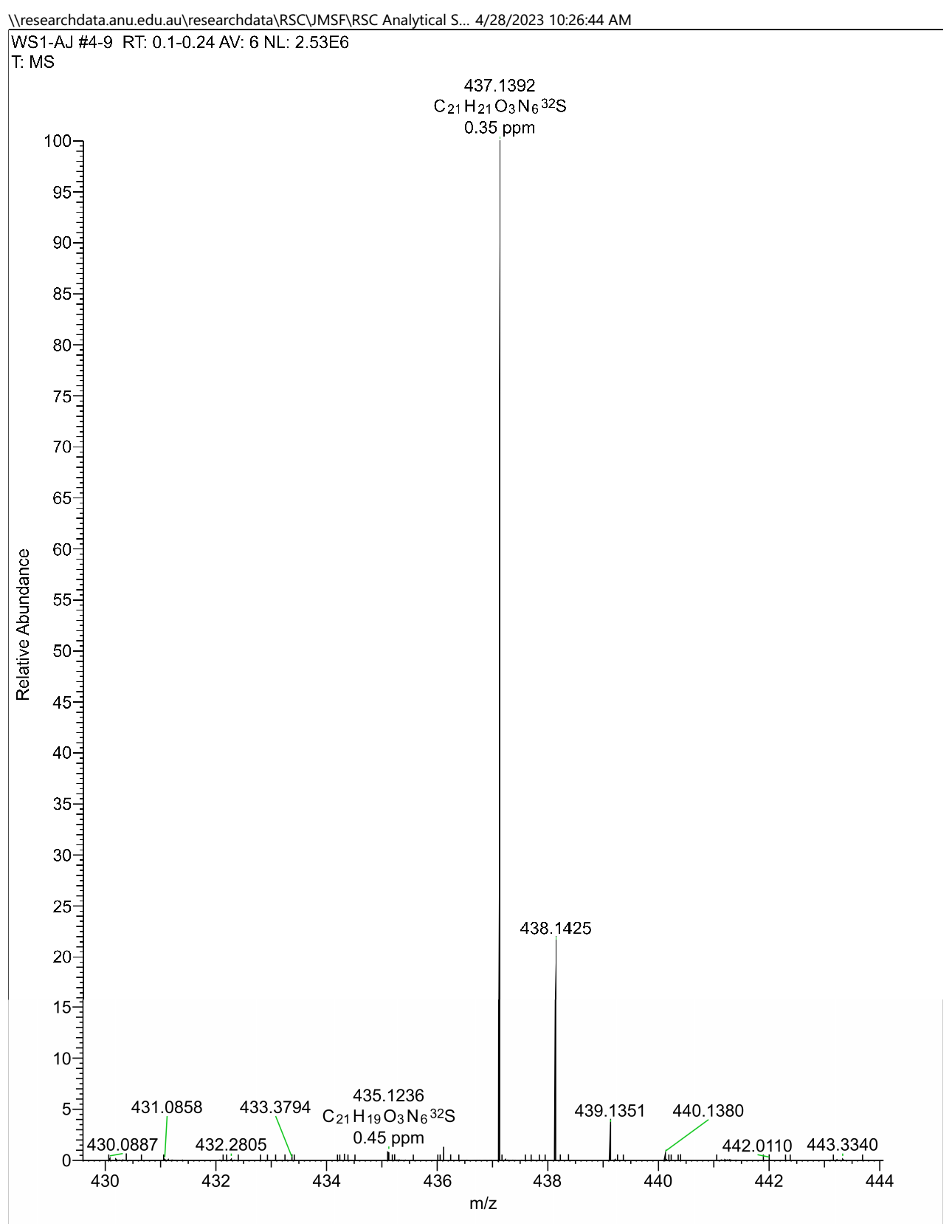


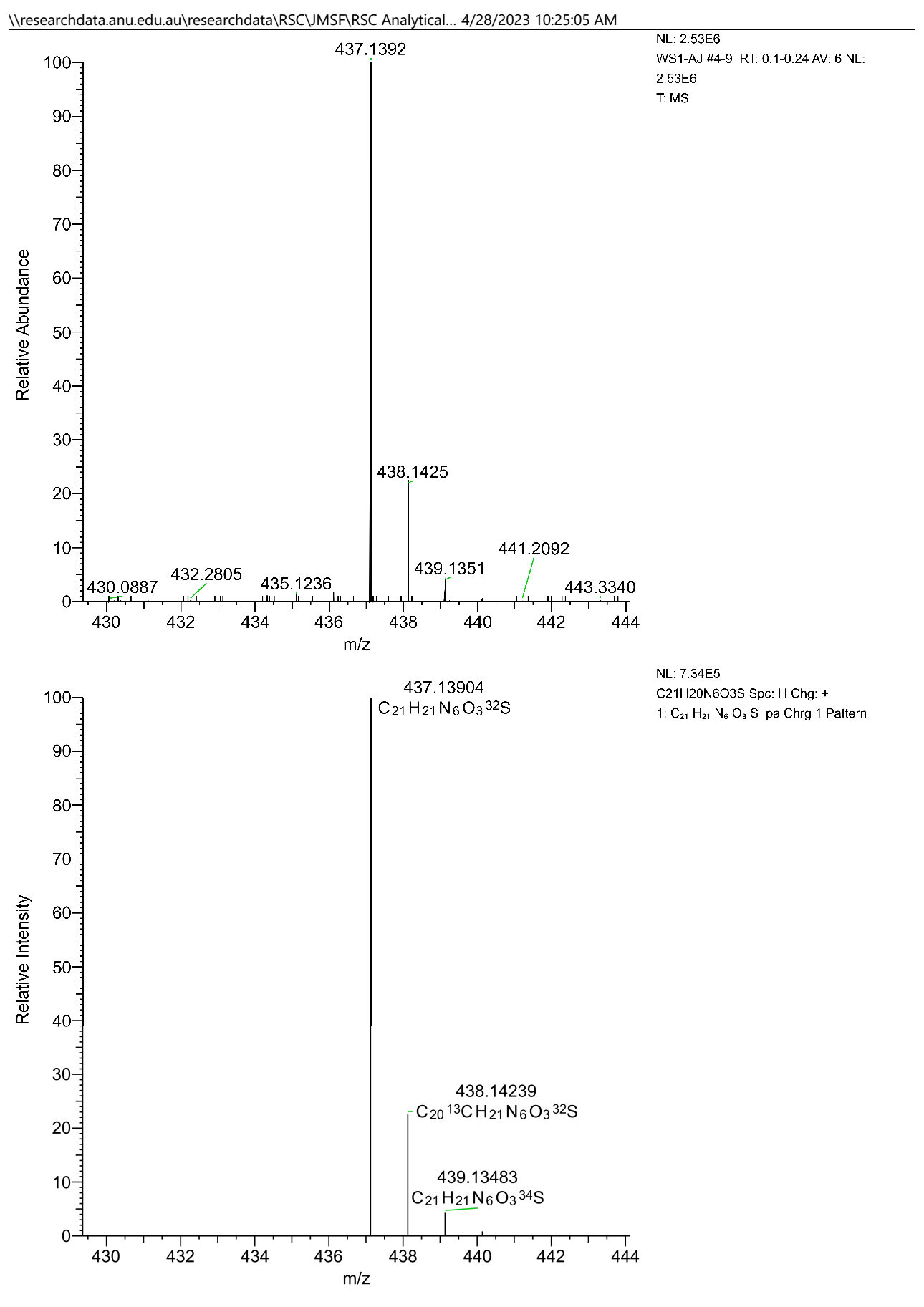


HRMS for compound 4b


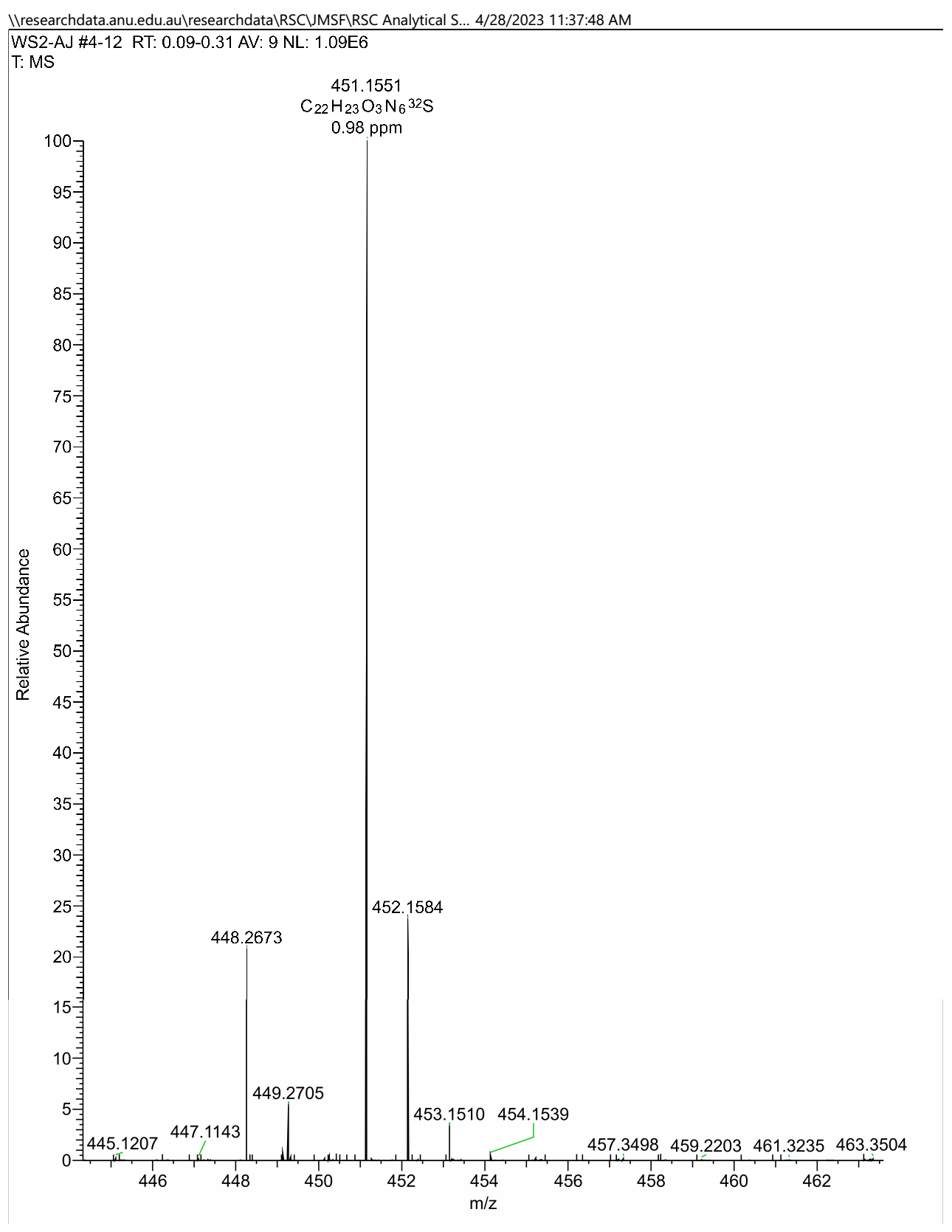


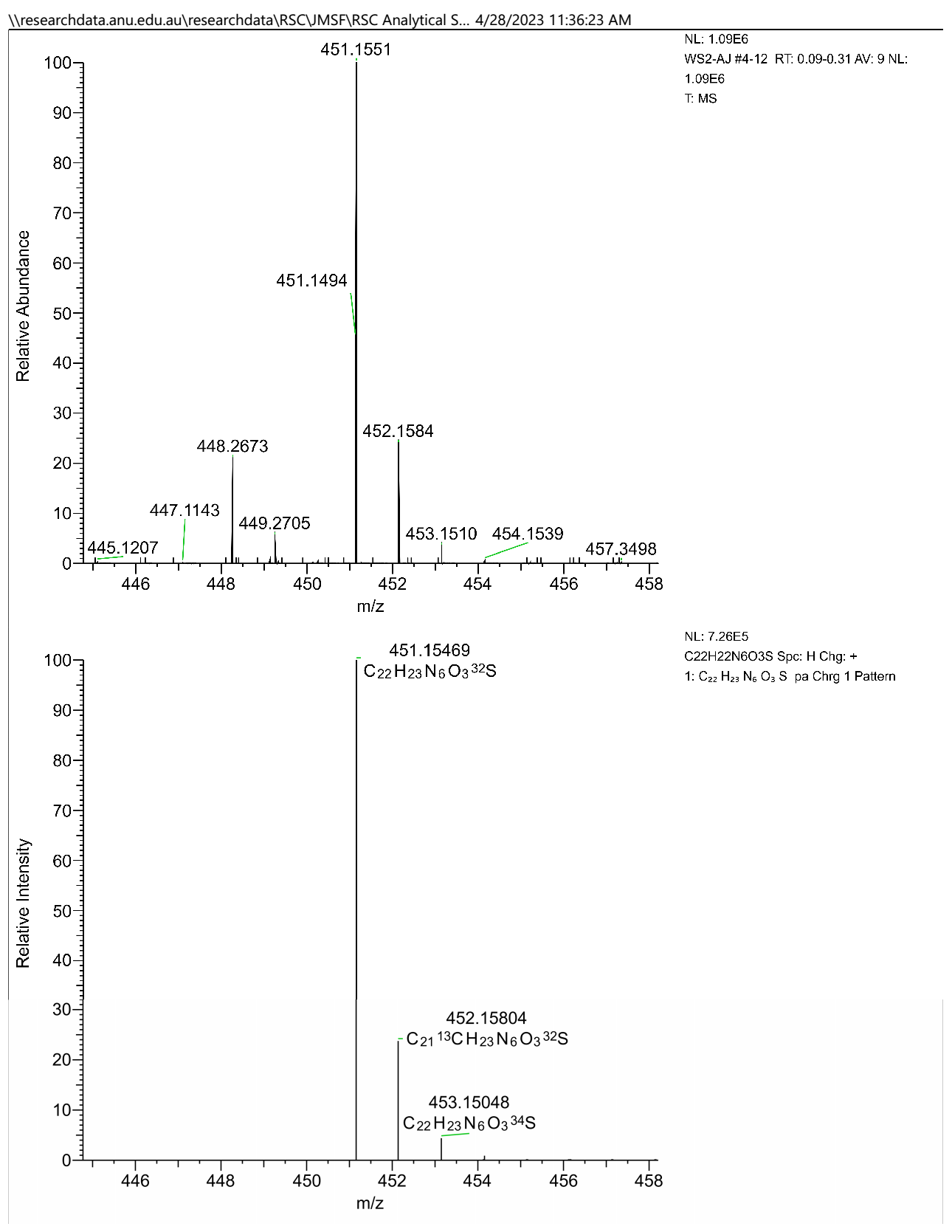


HRMS for compound 4c


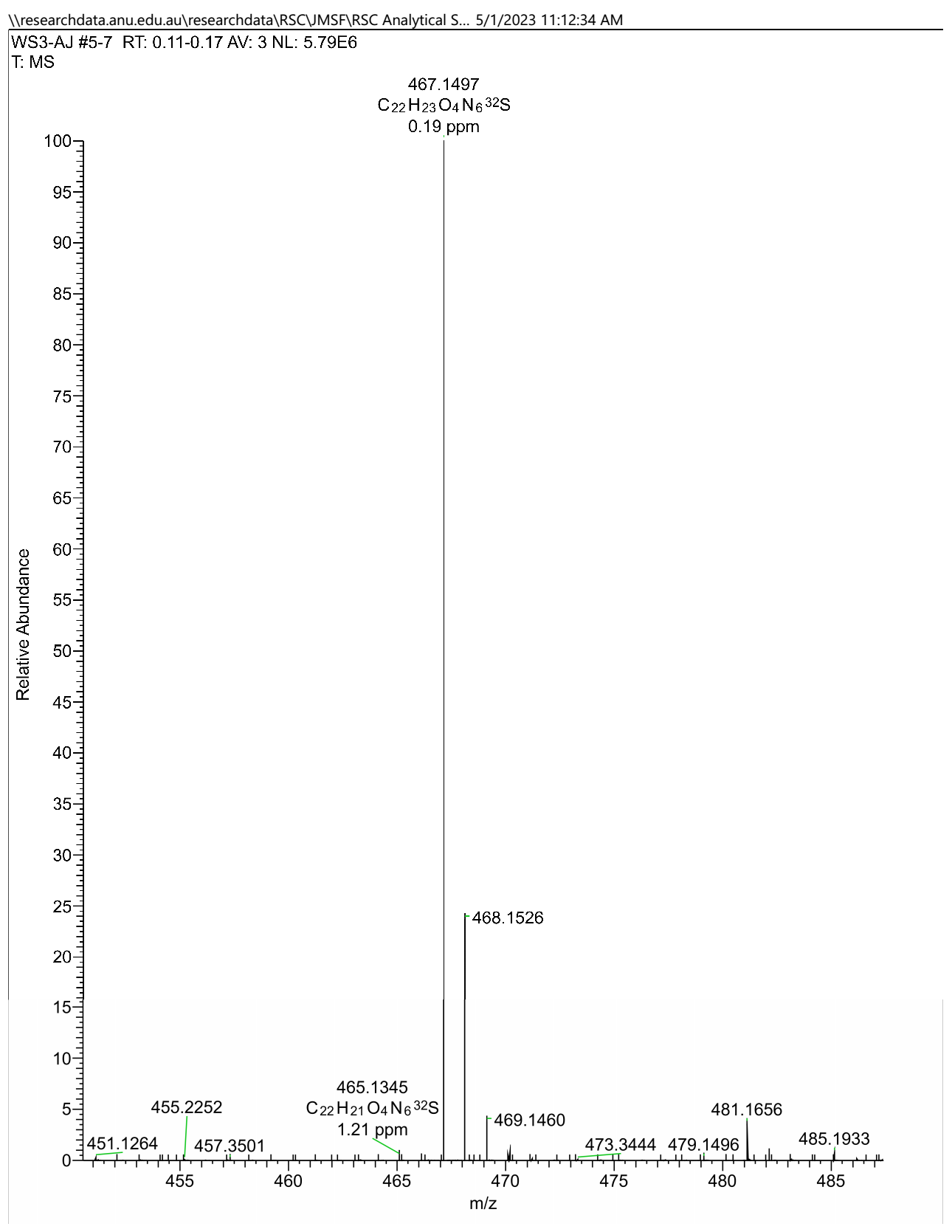


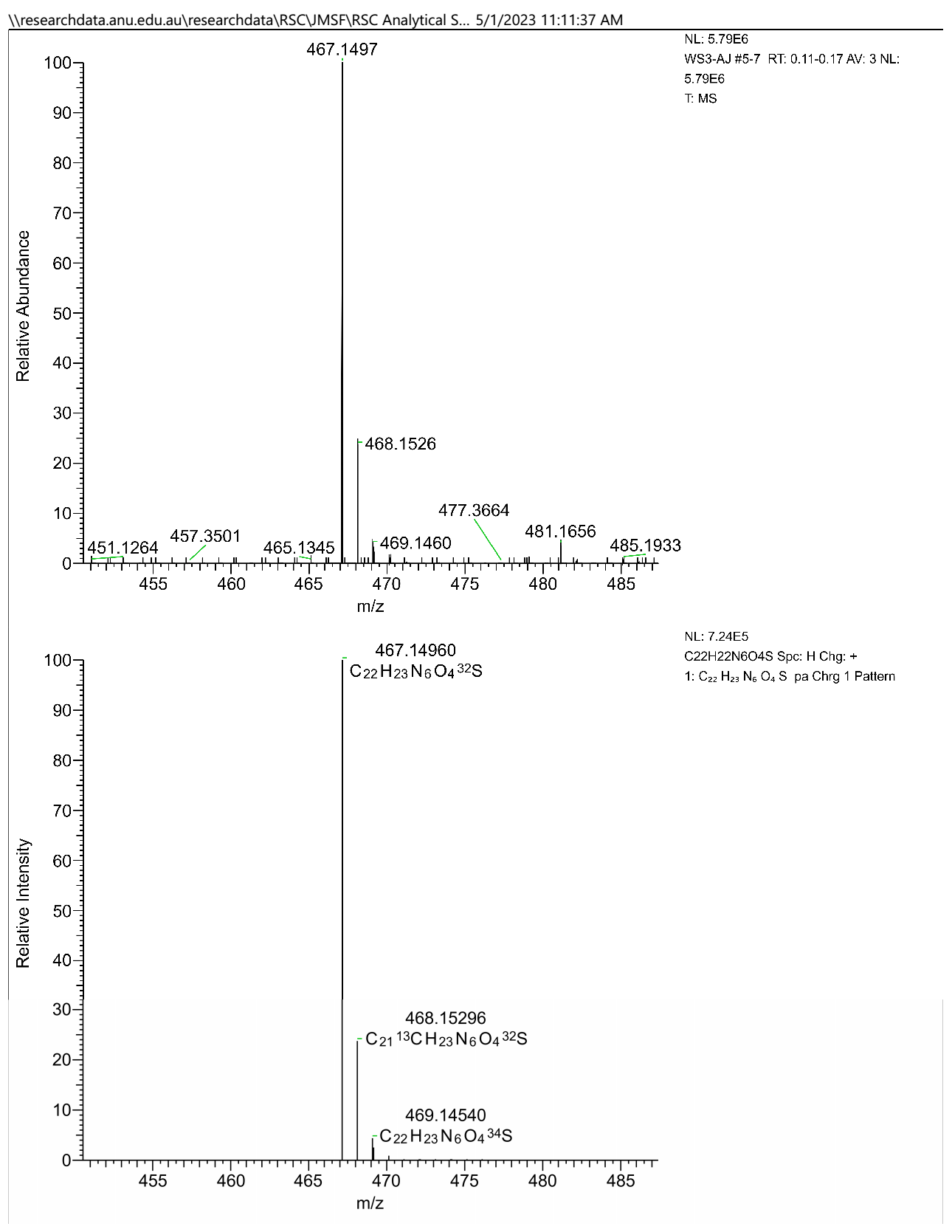


HRMS for compound 4d


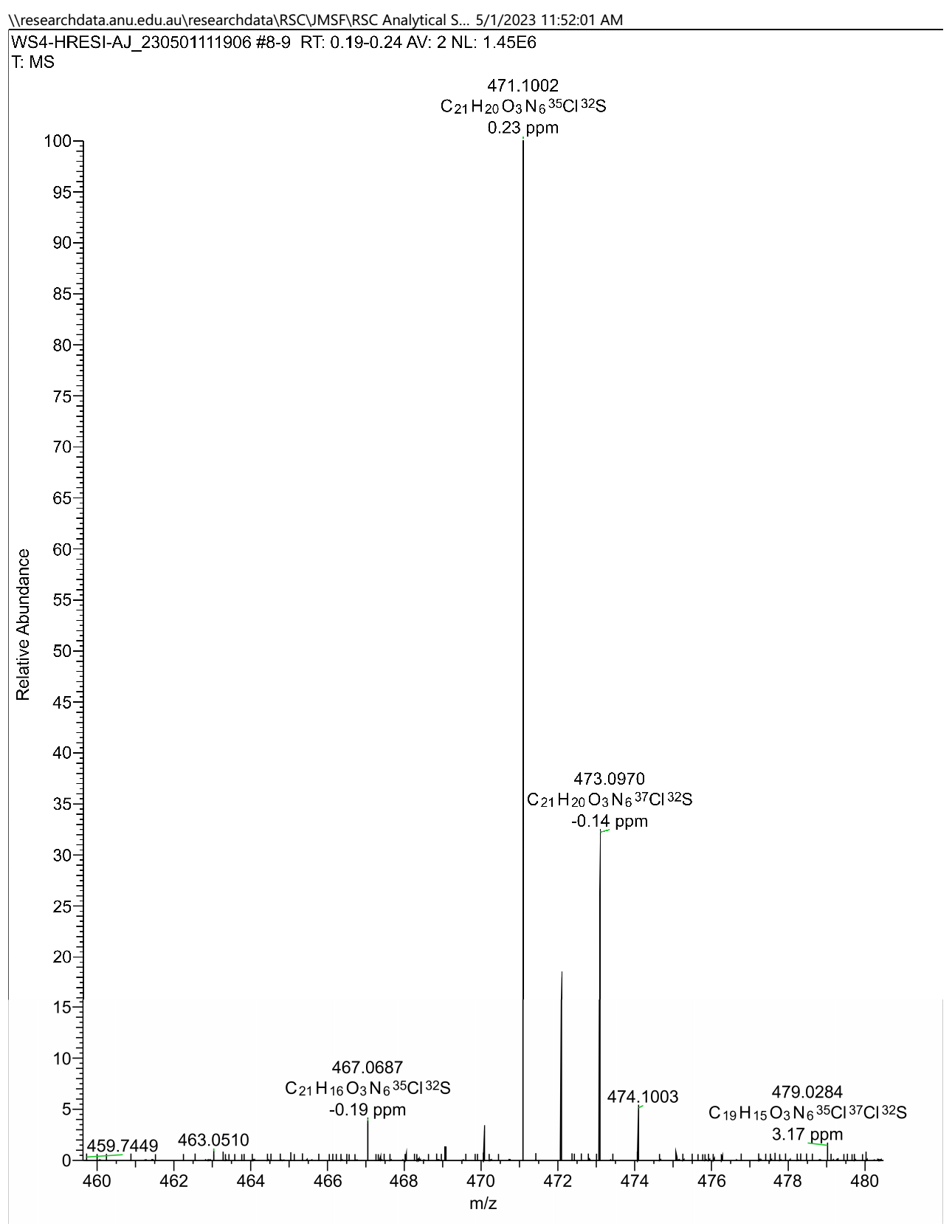


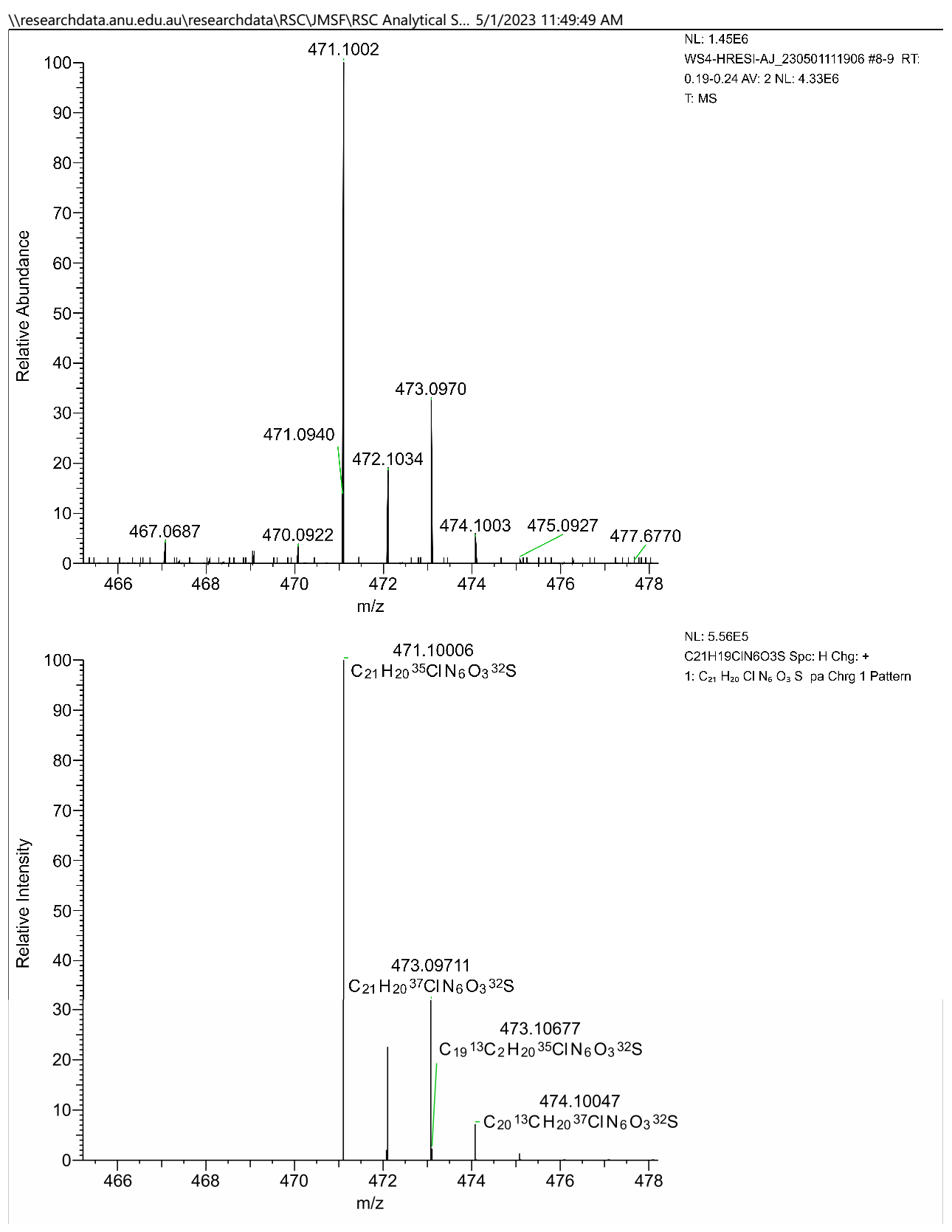


HRMS for compound 4e


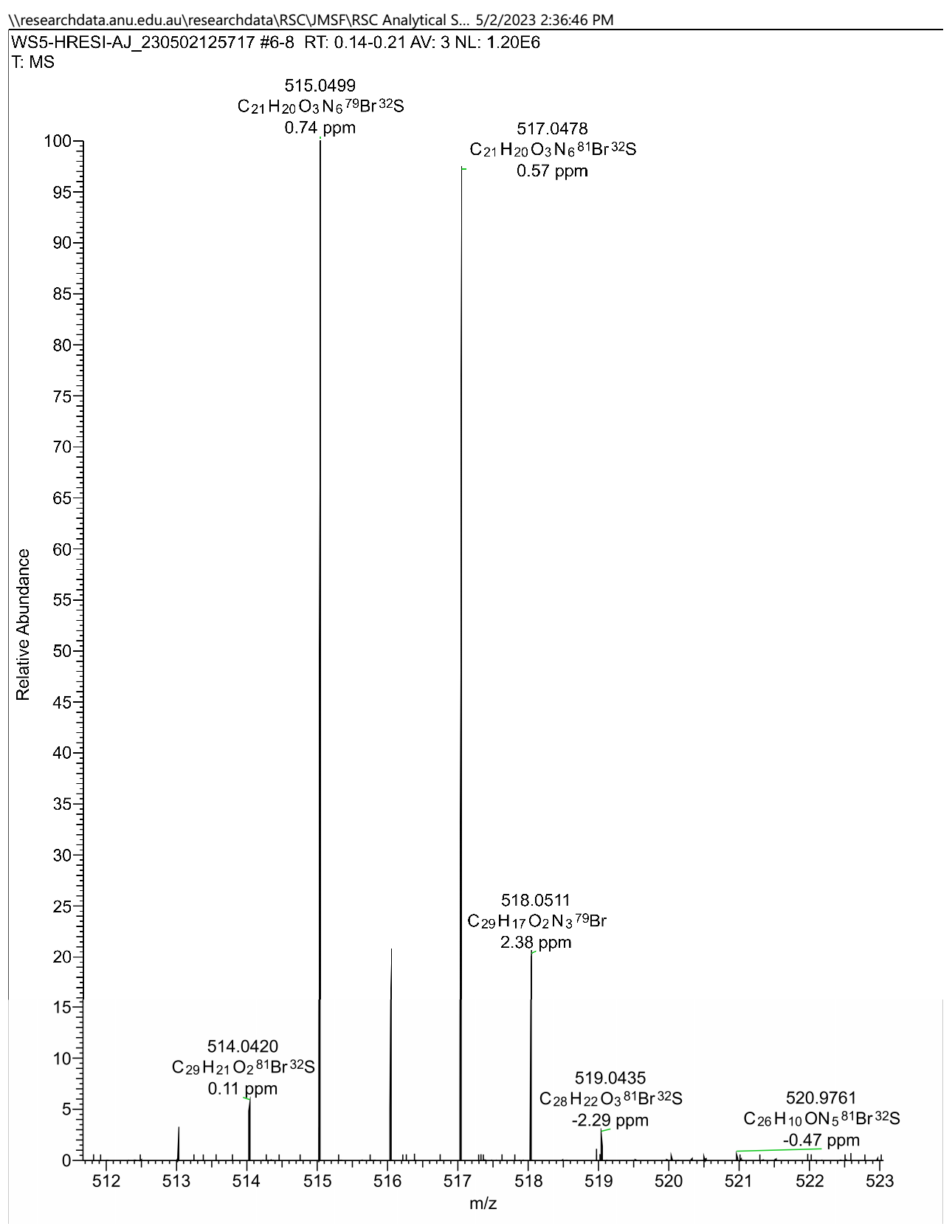


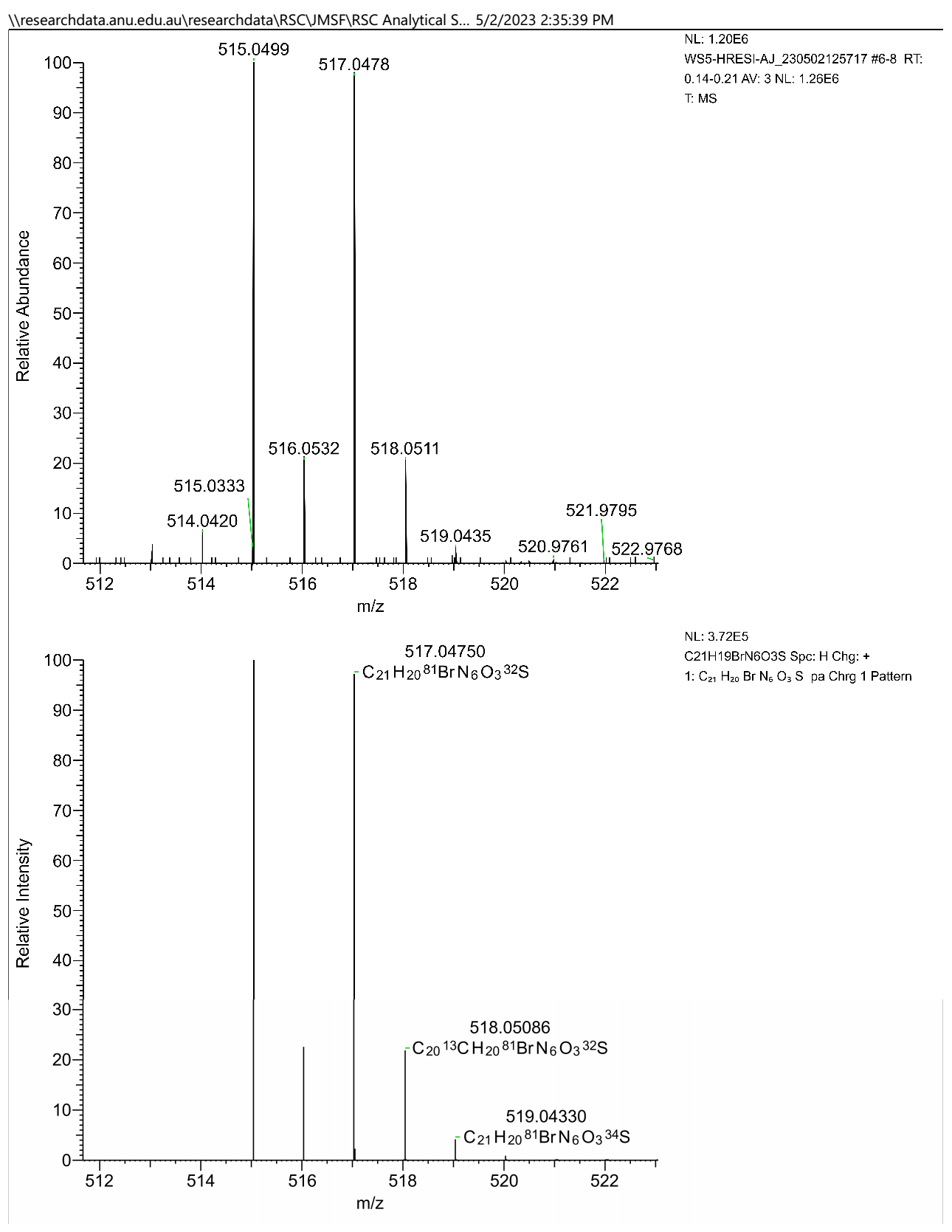


HRMS for compound 4f


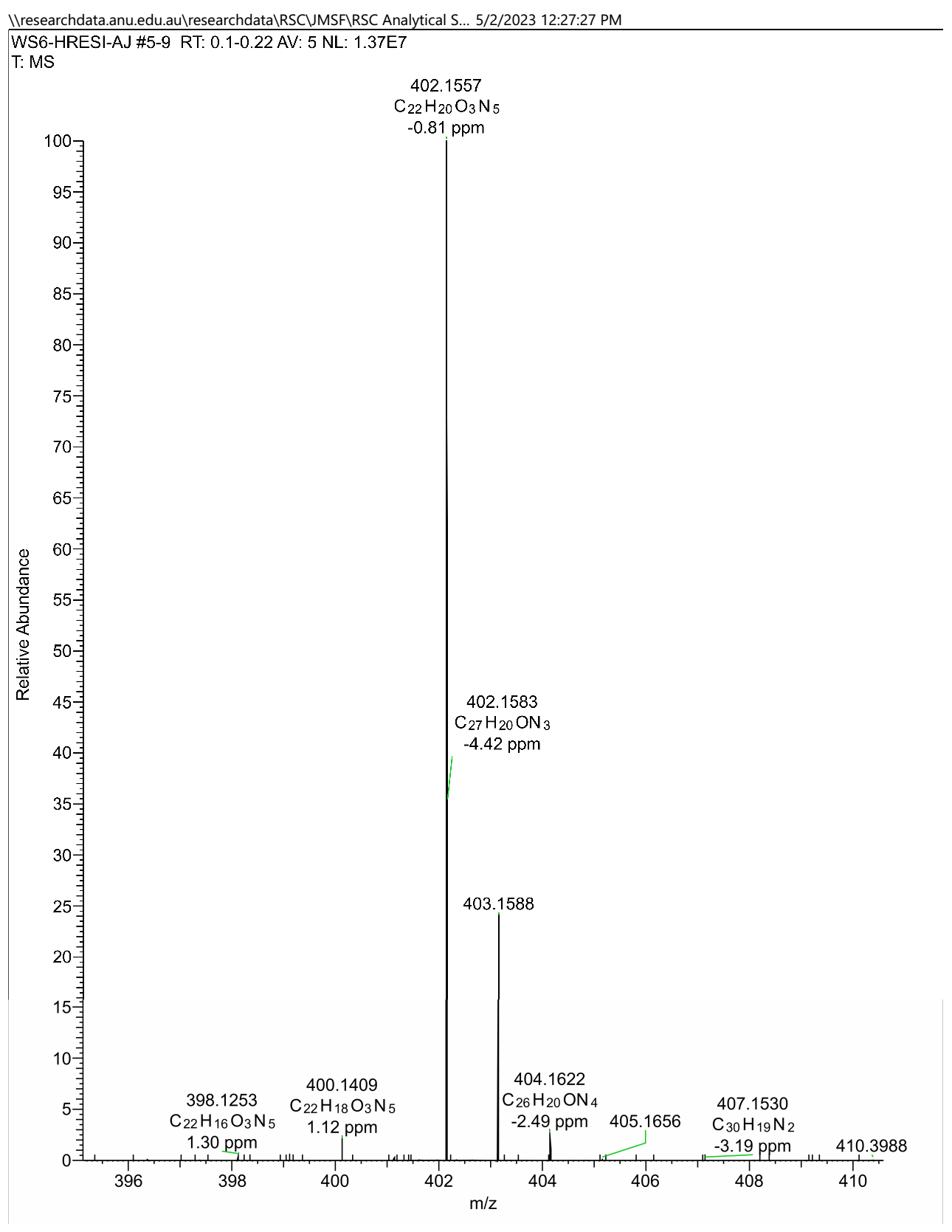


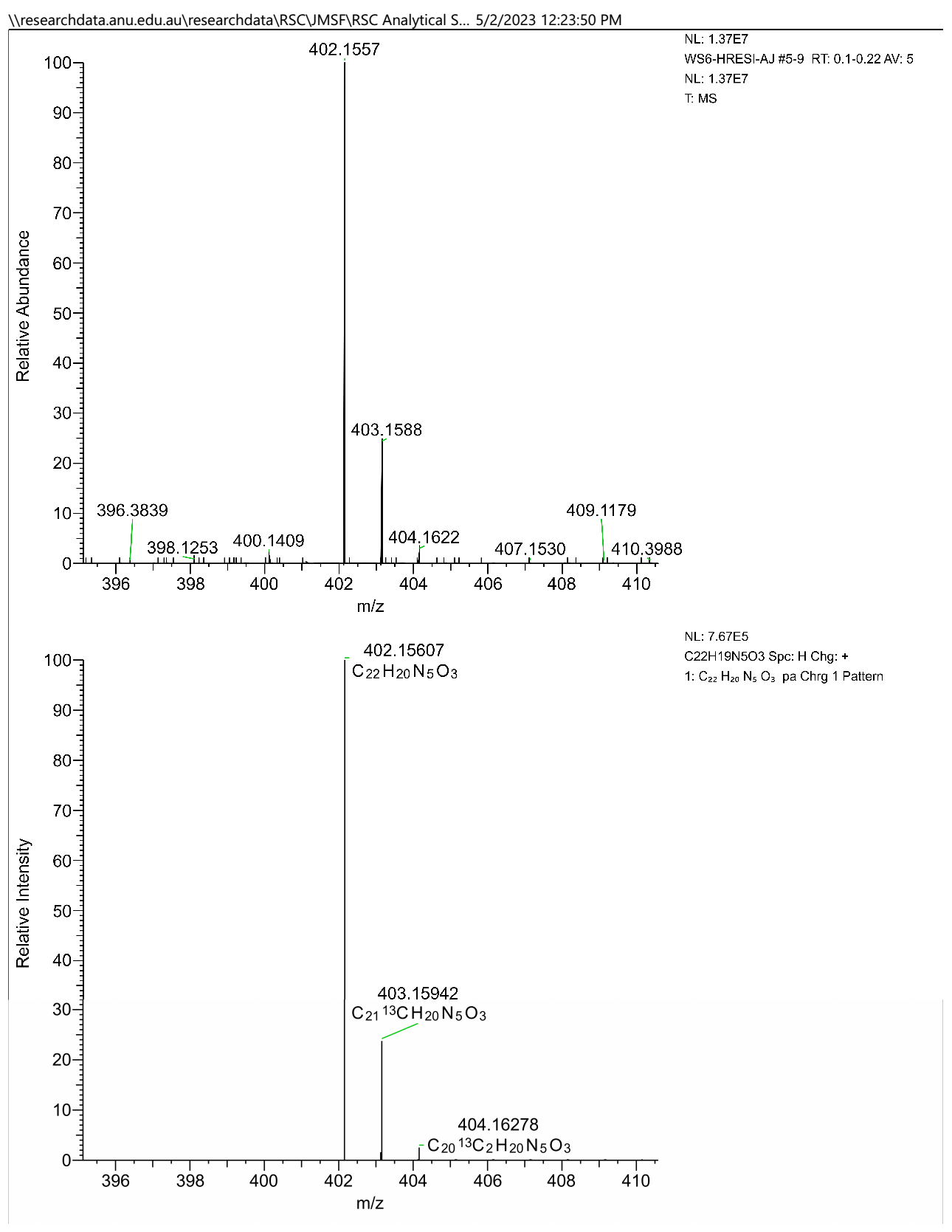


HRMS for compound 4g


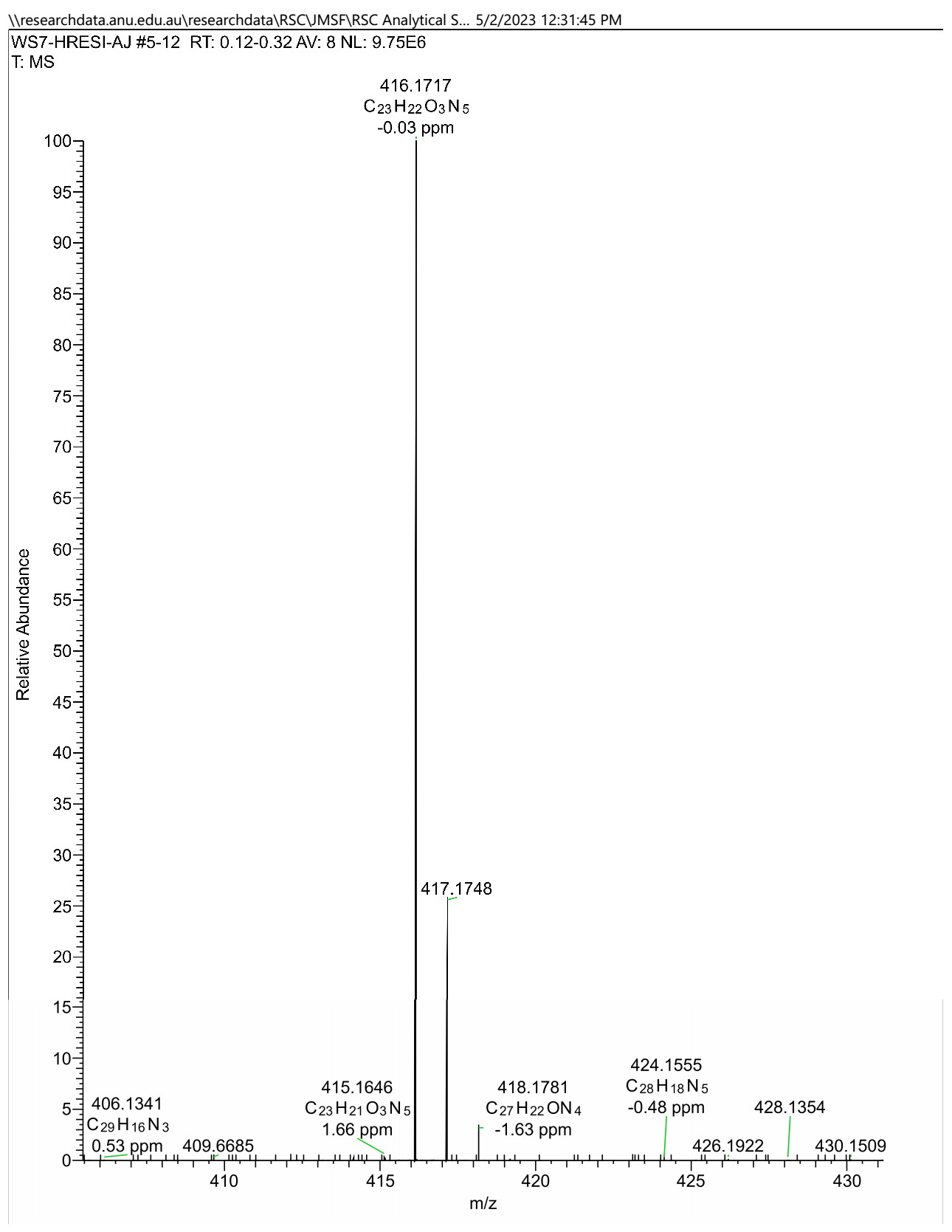


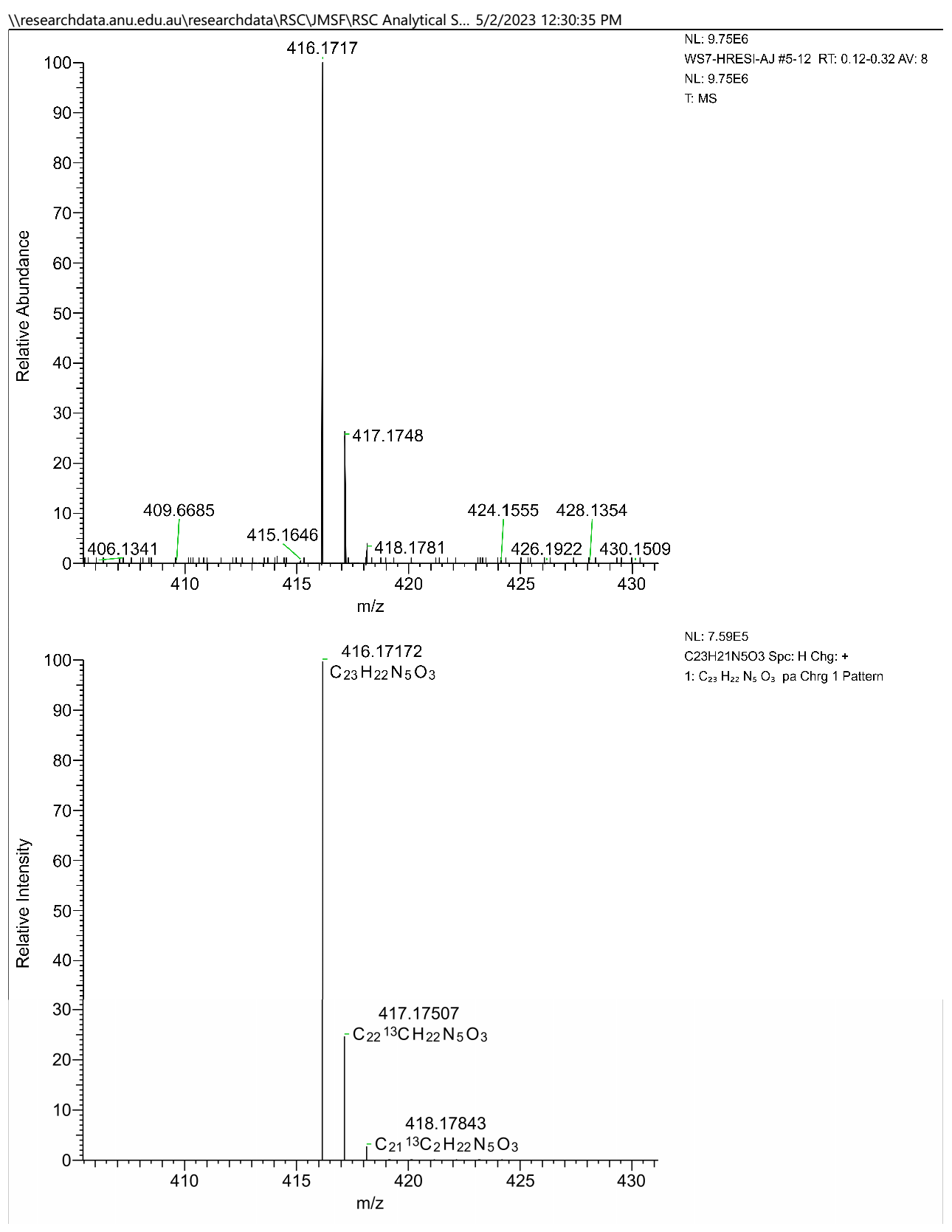


HRMS for compound 4h


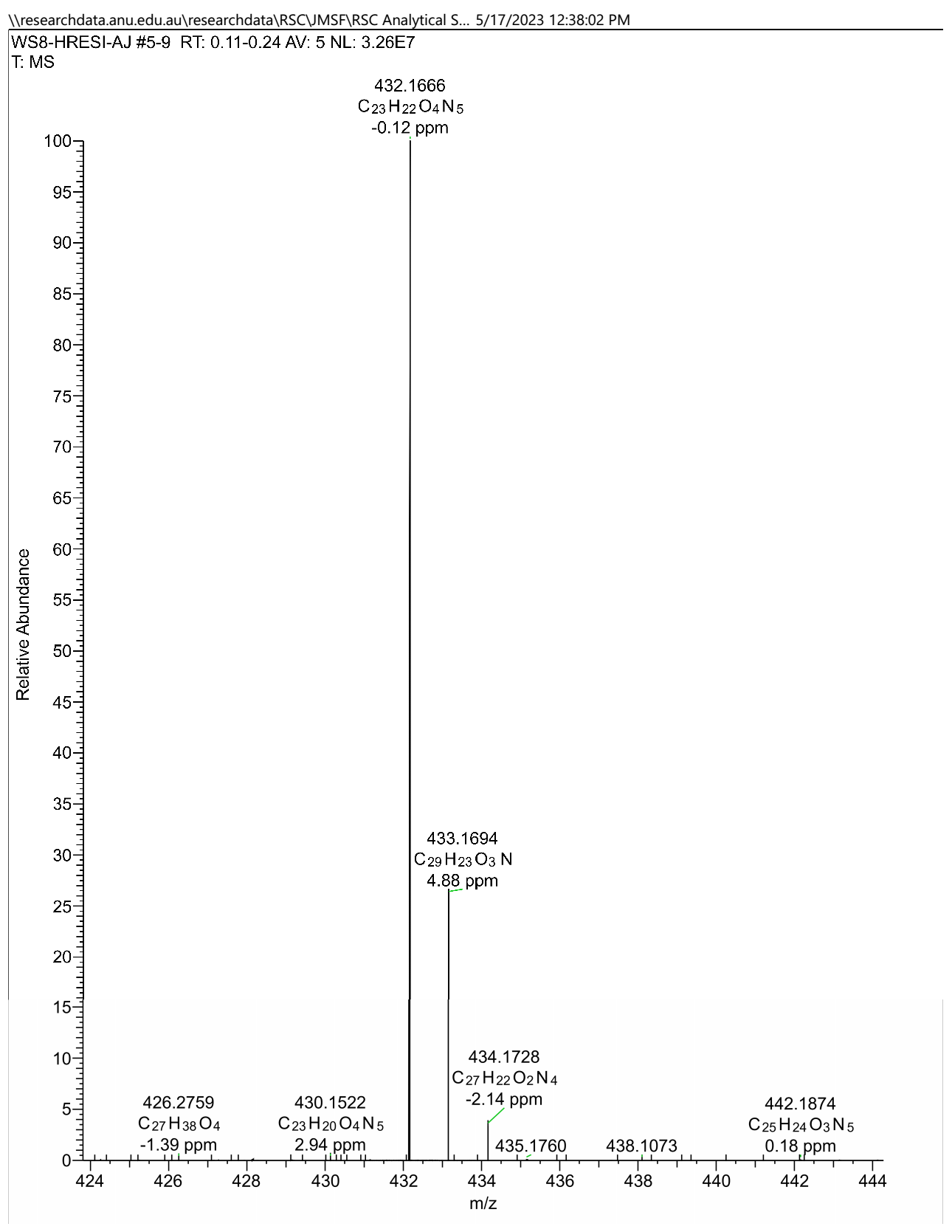


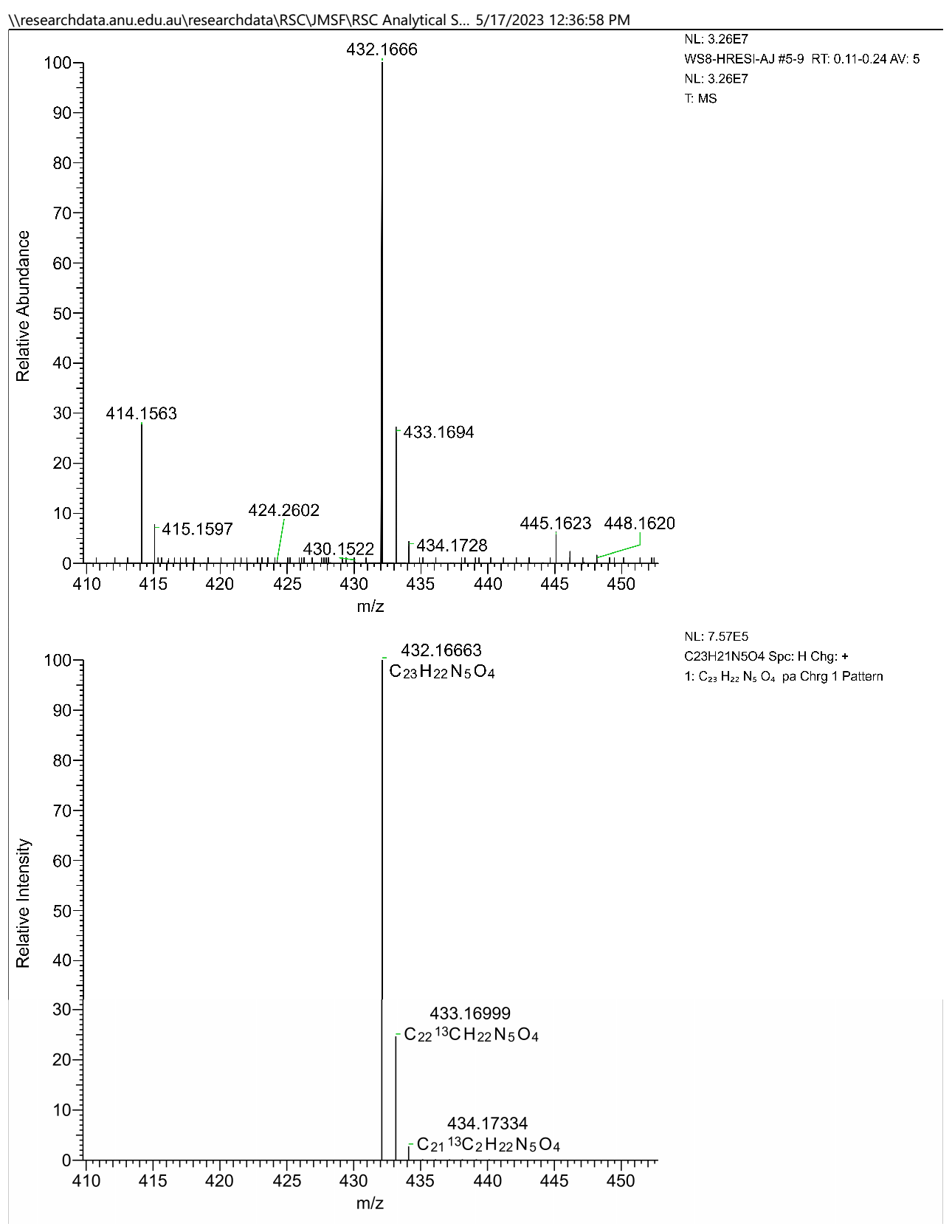


HRMS for compound 4i


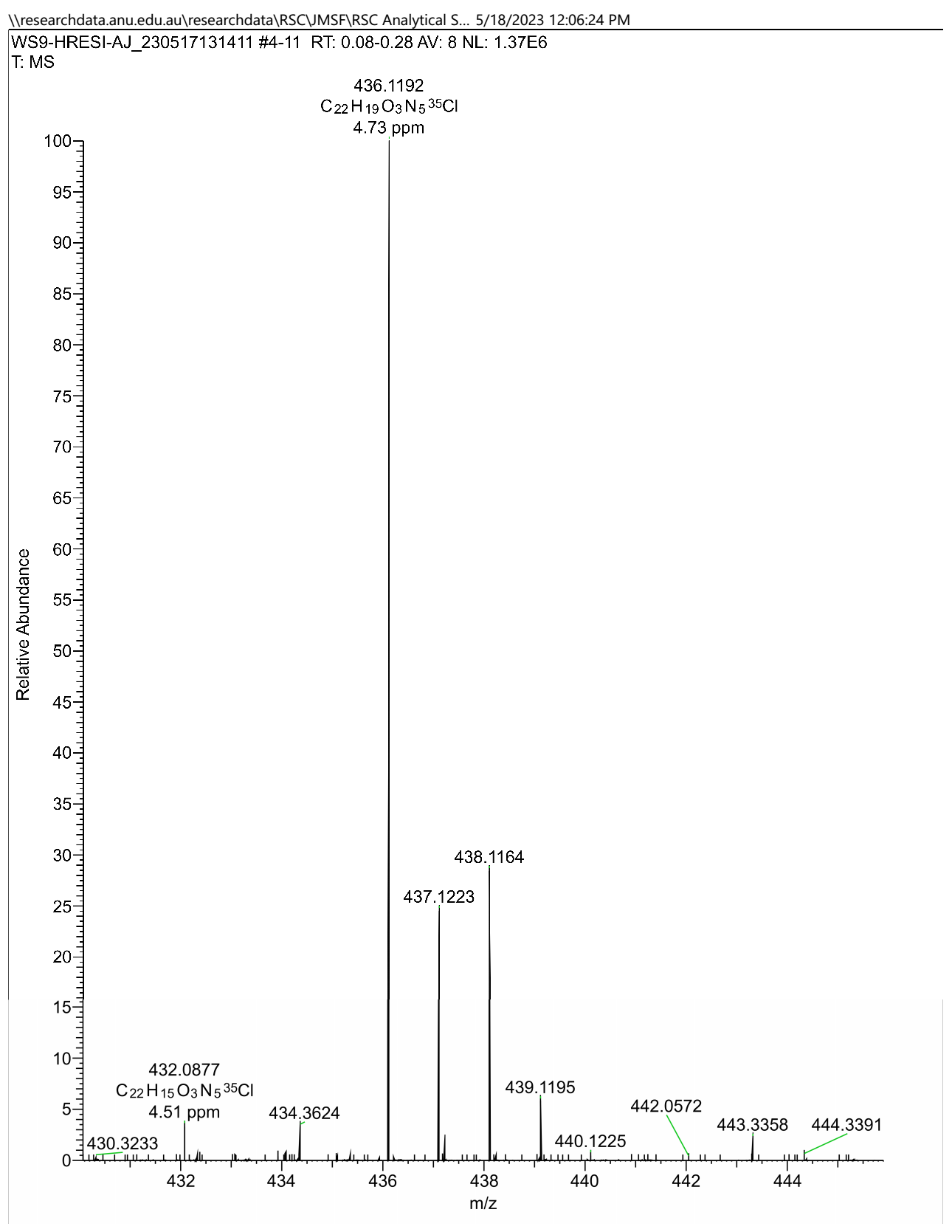


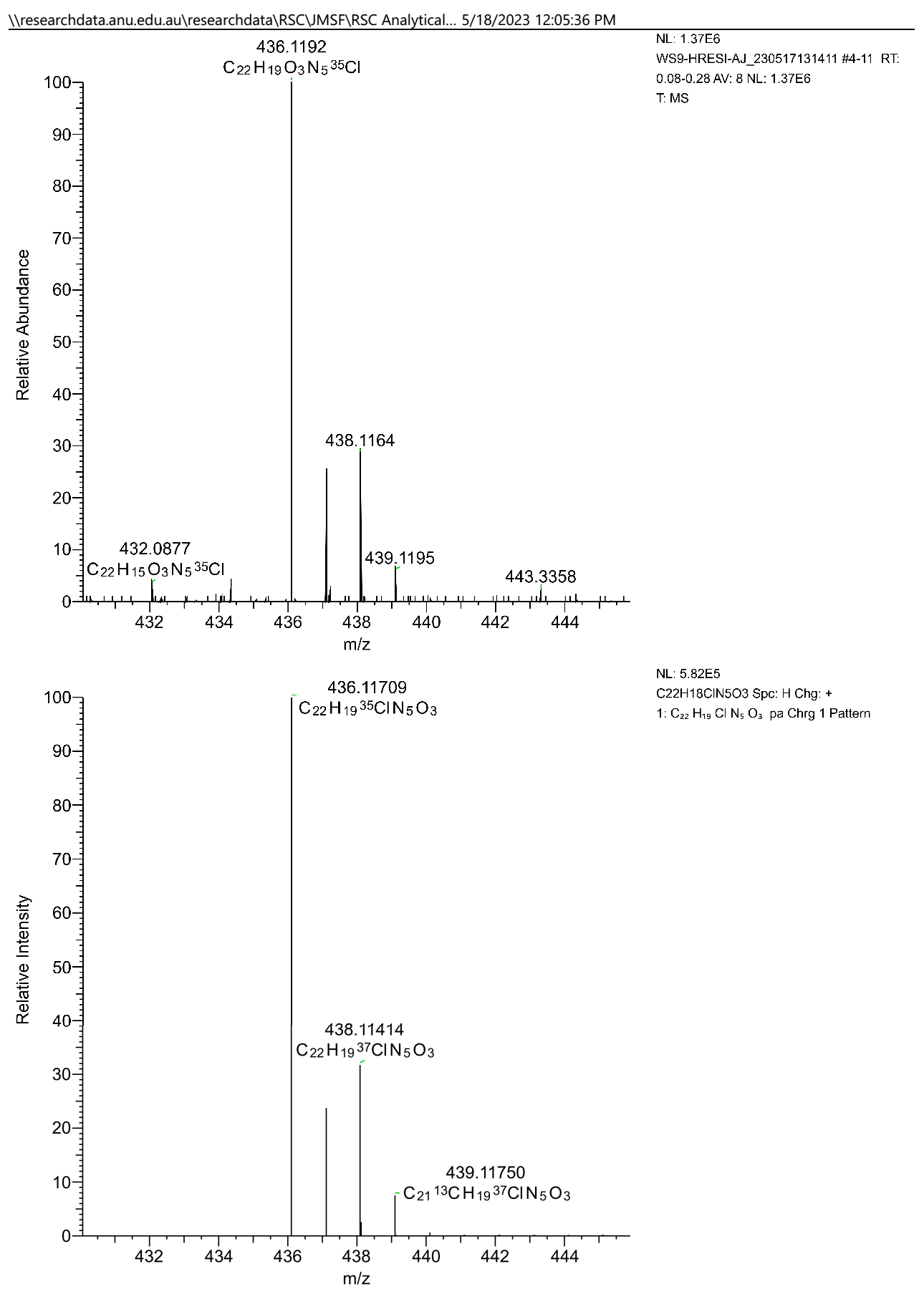


HRMS for compound 4j


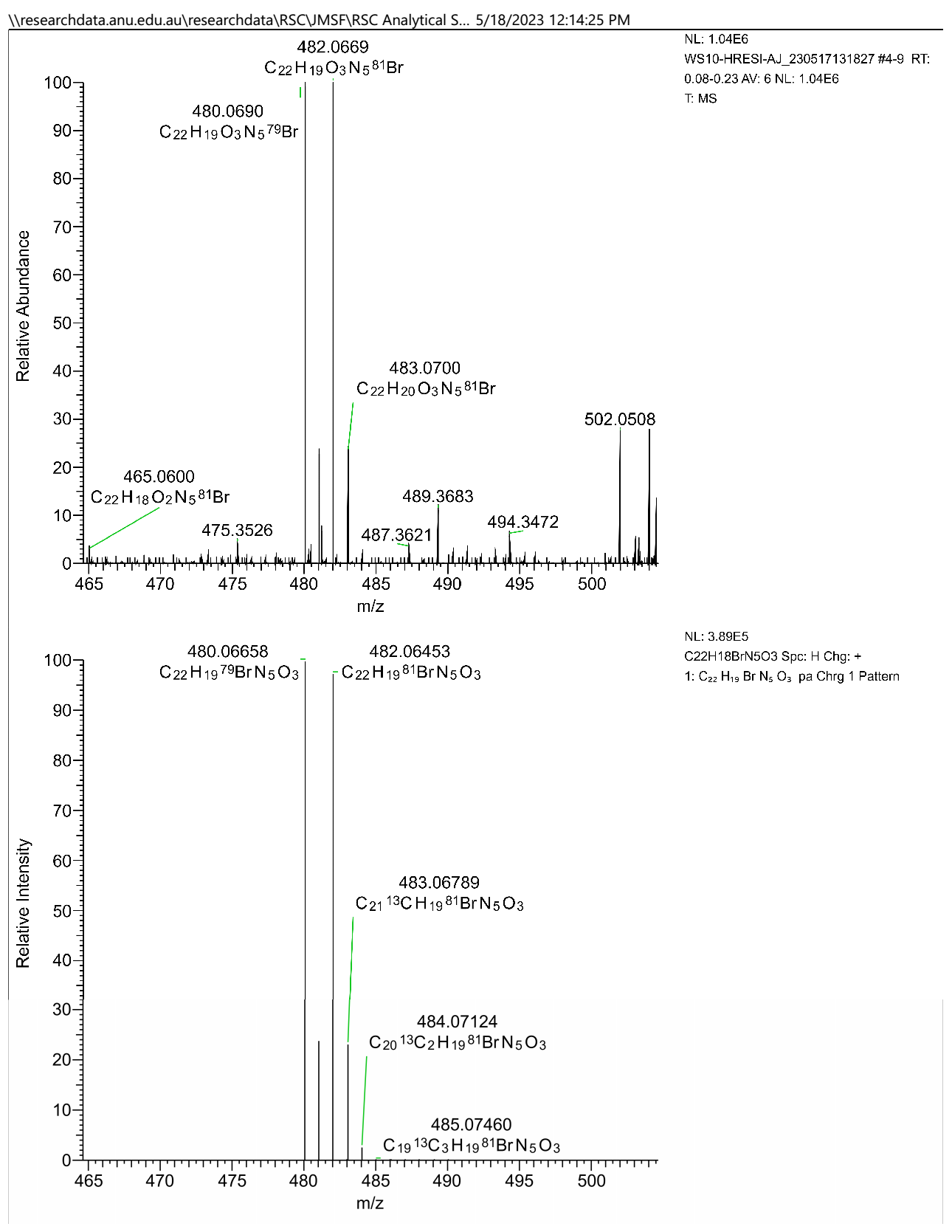


|  |  |  |
| --- | --- | --- |
| **Ws1** | **4a** |  |
| **Ws2** | **4b** |  |
| **Ws3** | **4c** |  |
| **Ws4** | **4d** |  |
| **Ws5** | **4e** |  |
| **Ws6** | **4f** |  |
| **Ws7** | **4g** |  |
| **Ws8** | **4h** |  |
| **Ws9** | **4i** |  |
| **Ws10** | **4j** |  |

| **** | **4f** | **** | **4a** |
| --- | --- | --- | --- |
| **** | **4g** | **** | **4b** |
| **** | **4h** | **** | **4C** |
| **** | **4i** | **** | **4d** |
| **** | **4j** | **** | **4e** |

**4.2. Biological evaluation**

**Biological Evaluation**

## 2.2. Biology

### 2.2.1. National Cancer Institute (NCI) screening

Compounds **2a-e and 4a-j** were selected by the National Cancer Institute (NCI) according to the protocol of the Compound Evaluation Branch of the National Cancer Institute, Bethesda, USA, for *in vitro* anticancer screening which refers in turn to apoptotic activity. The methodology of the NCI anticancer screening has been described in detail elsewhere (http://www.dtp.nci.nih.gov).

The results revealed that all compounds exhibit very weak to no apoptotic activity against all NCI cell lines as shown in above table. Moreover, the growth percentage of many tested cell lines was increased more than 100% . The results indicated that **4d-g** have promising activity on HOP-92 lung, MOLT-4 leukemia, and T-47D breast cell lines. Interestingly, compound **4e** showed remarkable activity over most NCI cancer cell lines.

**4.2.1. Cytotoxic activity using MTT Assay and evaluation of IC_50_**

**4.2.1.1. MTT assay**

MTT assay was performed to investigate the effect of the synthesized compounds on mammary epithelial cells (MCF-10A) [33, 34]. The cells were propagated in medium consisting of Ham's F-12 medium/ Dulbecco's modified Eagle's medium (DMEM) (1:1) supplemented with 10% foetal calf serum, 2 mM glutamine, insulin (10 μg/mL), hydrocortisone (500 ng/mL) and epidermal growth factor (20 ng/mL). Trypsin ethylene diamine tetra acetic acid (EDTA) was used to passage the cells after every 2-3 days. 96-well flat-bottomed cell culture plates were used to seed the cells at a density of 104 cells mL-1. The medium was aspirated from all the wells of culture plates after 24 hr followed by the addition of synthesized compounds (in 200 μL medium to yield a final concentration of 0.1% (v/v) dimethylsulfoxide) into individual wells of the plates. Four wells were designated to a single compound. The plates were allowed to incubate at 37°C for 96 h. Afterwards, the medium was aspirated and 3-[4,5-dimethylthiazol-2-yl]-2,5-diphenyltetrazolium bromide (MTT) (0.4 mg/mL) in medium was added to each well and subsequently incubated for 3 h. The medium was aspirated and 150 μL dimethyl sulfoxide (DMSO) was added to each well. The plates were vortexed followed by the measurement of absorbance at 540 nm on a microplate reader. The results were presented as inhibition (%) of proliferation in contrast to controls comprising 0.1% DMSO.

**4.2.1.2. Assay for antiproliferative effect**

**4.2.2. MTT assay**

Cells were cultured using DMEM (Invitrogen/Life Technologies) supplemented with 10% FBS (Hyclone,), 10 ug/ml of insulin (Sigma), and 1% penicillin-streptomycin. Plate cells (cells density 1.2 – 1.8 × 10,000 cells/well) in a volume of 100µl complete growth medium + 100 ul of the tested compound per well in a 96-well plate for 24 hours before the MTT assay [32]. In atypical experiment, one hundred micro liters of serial 10-fold diluted sterile tested compounds were added to final concentrations of 0.01e100 mM using culture media as negative control. After 24 h of culture incubation and supernatants discarded. HOP-92 lung, MOLT-4 leukemia, and T-47D breast cell lines were trypsinized and washed with Ca2‏/Mg2‏ free PBS (pH 7.2). we removed cultures from incubator into laminar flow hood or other sterile work area. Cells in the log phase of growth should be employed and final cell number should not exceed 106 cells/cm^2^. Each test should include a blank containing complete medium without cells. Reconstitute each vial of MTT [M-5655] to be used with 3 ml of medium or balanced salt solution without phenol red and serum. Add reconstituted MTT in an amount equal to 10% of the culture medium volume. Return cultures to incubator for 2-4 hours depending on cell type and maximum cell density. After the incubation period, remove cultures from incubator and dissolve the resulting formazan crystals by adding an amount of MTT Solubilization Solution [M-8910] equal to the original culture medium volume. Spectrophotometrically absorbance was measured at a wavelength of 570 nm. Measure the background absorbance of multiwell plates at 690 nm and subtract from the 450 nm measurement. Results from all experiments were recorded and the percentage of viable cells was calculated.

**4.2.3. EGFR inhibitory assay**

The activity of compounds **4d,4e,4f and 4g** using erlotinib as reference against EGFR was investigated using (BPS EGFR Kinase Assay Kit Catalog # 40321 bpsbioscience U.S) [33].

Baculoviral expression vectors including pBlueBacHis2B and pFASTBacHTc were used separately to clone 1.6 kb cDNA coding for EGFR cytoplasmic domain (EGFR-CD, amino acids 645–1186). 5ʹ upstream to the EGFR sequence comprised a sequence that encoded (His)6. Sf-9 cells were infected for 72h for protein expression. The pellets of Sf-9 cells were solubilized in a buffer containing sodium vanadate (100 μM), aprotinin (10 μg/mL), triton (1%), HEPES buffer (50mM), ammonium molybdate (10 μM), benzamidineHCl (16 μg/mL), NaCl (10 mM), leupeptin (10 μg/mL) and pepstatin (10 μg/mL) at 0°C for 20 min at pH 7.4, followed by centrifugation for 20 min. To eliminate the nonspecifically bound material, a Ni-NTA superflow packed column was used to pass through and wash the crude extract supernatant first with 10 mM and then with 100 mM imidazole. Histidine-linked proteins were first eluted with 250 and then with 500 mM imidazole subsequent to dialysis against NaCl (50 mM), HEPES (20 mM), glycerol (10%) and 1 μg/mL each of aprotinin, leupeptin and pepstatin for 120 min. The purification was performed either at 4 °C or on ice. To record autophosphorylation level, EGFR kinase assay was carried out on the basis of DELFIA/Time-Resolved Fluorometry. The compounds were first dissolved in DMSO absolute, subsequent to dilution to appropriate concentration using HEPES (25 mM) at pH 7.4. Each compound (10 μL) was incubated with recombinant enzyme (10 μL, 5 ng for EGFR, 1:80 dilution in 100 mM HEPES) for 10 min at 25^o^C, subsequent to the addition of 5X buffer (10 μL, containing 2 mM MnCl2, 100 μM Na3VO4, 20 mM HEPES and 1 mM DTT) and ATP-MgCl2 (20 μL, containing 0.1 mM ATP and 50 mM MgCl2) and incubation for 1h. The negative and positive controls were included in each plate by the incubation of enzyme either with or without ATP-MgCl2. The liquid was removed after incubation and the plates were washed thrice using wash buffer. Europium-tagged antiphosphotyrosine antibody (75 μL, 400 ng) was added to each well followed by incubation of 1h and then washing of the plates using buffer. The enhancement solution was added to each well and the signal was recorded at excitation and emission wavelengths of 340 at 615 nm. The autophosphorylation percentage inhibition by compounds was calculated using the following equation:

Using the curves of percentage inhibition of eight concentrations of each compound, IC50 was calculated. Majority of signals detected by antiphosphotyrosine antibody were from EGFR because the enzyme preparation contained low impurities.

**4.2.4. Cell cycle apoptosis and detection**

**4.2.4.1. Annexin V-FITC apoptosis assay**

Cell apoptosis were analyzed by the Annexin V-FITC Apoptosis Detection Kit (BioVision Research Products, USA). Thereafter, 1-5 x 105 cells was collected by centrifugation and resuspend in 500 μl of 1X Binding Buffer. Added 5 μl of Annexin V-FITC and 5 μl of propidium iodide (PI 50mg/ml, optional.). The cells incubated at room temperature for 5 min in the dark;Analyzed by Annexin V-FITC binding flow cytometric method (Ex = 488 nm; Em = 530 nm) usingFITC signal detector (usually FL1) and PI staining by the phycoerythrin emissionsignal detector (usually FL2).For adherent cells, we gently trypsinized and washed cells once with serum-containing mediabefore incubation with Annexin V-FITC (A.3-5).

**4.2.4.2. Activation of Caspases**

**Caspase-3 activation assay**

Allow all reagents to reach room temperature before use. Gently mix all liquidreagents prior to use. Determine the number of 8-well strips needed for the assay. Insert these in the frame(s) for current use. Add 100 μl of the *Standard Diluent Buffer* to the zero standard wells. Well(s) reserved for chromogen blank should be left empty. Add 100 μl of standards and controls or diluted samples to the appropriate microtiter wells. The sample dilution chosen should be optimized for each experimental system. Tap gently on side of plate to mix. Cover wells with *plate cover* and incubate for 2 hours at room temperature. Thoroughly aspirate or decant solution from wells and discard the liquid, Wash wells 4 times. Pipette 100 μl of *Caspase-3 (Active) Detection Antibod*y solution into each well except the chromogen blank(s). Tap gently on the side of the plate to mix. Cover plate with *plate cover* and incubate for 1 hour at room temperature. Thoroughly aspirate or decant solution from wells and discard the liquid, Wash wells 4 times. Add 100 μl Anti-Rabbit IgG HRP Working Solution to each well except the chromogen blank(s). Prepare the working dilution as described in Preparing IgG HRP. Cover wells with the *plate cover* and incubate for 30 minutes at room temperature. Thoroughly aspirate or decant solution from wells and discard the liquid. Wash wells 4 times. Add 100 μl of *Stabilized Chromogen*to each well. The liquid in the wells will begin to turn blue. Incubate for 30 minutes at room temperature and in the dark. The incubation time for chromogen substrate is often determined by the microtiter platereader used. Many plate readers have the capacity to record a maximum optical density (O.D.) of 2.0. The O.D. values should be monitored and the substrate reaction stopped before the O.D. of the positive wells exceeds the limits of the instrument. The O.D. values at 450 nm can only be read after the *Stop Solution* has been added to each well. If using a reader that records only to 2.0 O.D., stopping the assay after 20 to 25 minutes is suggested. Add 100 μl of *Stop Solution* to each well. Tap side of plate gently to mix. The solution in the wells should change from blue to yellow. Read the absorbance of each well at 450 nm having blanked the plate reader against a chromogen blank composed of 100 μl each of *StabilizedChromogen*and *Stop Solution*. Read the plate within 2 hours after adding the *Stop Solution*. Use a curve fitting software to generate the standard curve. A four-parameter algorithm provides the best standard curve fit. Read the concentrations for unknown samples and controls from the standard curve. Multiply value(s) obtained for sample(s) by the appropriate dilution factor to correct for the dilution in step 3. Samples producing signals greater than that of the highest standard should be diluted in *StandardDiluent Buffer* and reanalyzed.

**Caspase 9 activation assay**

Cells were obtained from American Type Culture Collection, cells were grown in RPMI 1640 containing 10% fetal bovine serum at 37°C, stimulated with the compounds to be tested for 9, and lysed with Cell Extraction Buffer. This lysate was diluted in Standard Diluent Buffer over the range of the assay and measured for human active caspase-8 or 9 content. (*cells are Plated in a density of 1.2 – 1.8 × 10,000 cells/well in a volume of 100µl complete growth medium + 100 ul of the tested compound per well in a 96-well plate for 24 hours before the enzyme assay*). The absorbance of each microwell was read on a spectro-photometer at 450 nm. A standard curve is prepared from 7 human Caspases standard dilutions and human Caspase-8 or 9 concentration determined.

**4.2.4.3. Effects Bcl-2 proteins**

**Bcl-2 inhibition assay**

Mix all reagents thoroughly without foaming before use. Wash the microwells twice with approximately 300 μL Wash Buffer per well with thorough aspiration of microwell contents between washes. Take caution not to scratch the surface of the microwells. After the last wash, empty the wells and tap microwell strips on absorbent pad or paper towel to remove excess Wash Buffer. Use the microwell strips immediately after washing or place upside down on a wet absorbent paper for not longer than 15 minutes. Do not allow wells to dry. Add 100 μL of Sample Diluent in duplicate to all standard wells and to the blank wells. Prepare standard (1:2 dilution) in duplicate ranging from 32 ng/mL to 0.5 ng/mL. Add 100 μL of Sample Diluent, in duplicate, to the blank wells. Add 80 μL of Sample Diluent, in duplicate, to the sample wells. Add 20 μL of each Sample, in duplicate, to the designated wells. Add 50 μL of diluted biotin-conjugate to all wells, including the blank wells. Cover with a plate cover and incubate at room temperature, on a microplate shaker at 100 rpm if available, for 2 hours. Remove plate cover and empty the wells. Wash microwell strips 3 times as described in step 2. Add 100 μL of diluted Streptavidin-HRP to all wells, including the blank wells. Cover with a plate cover and incubate at room temperature, on a microplate shaker at 100 rpm if available, for 1 hour. Remove plate cover and empty the wells. Wash microwell strips 3 times as described in step 2. Proceed to the next step. Pipette 100 μl of mixed TMB Substrate Solution to all wells, including the blanks. Incubate the microwell strips at room temperature (18° to 25°C) for about 15 minutes, if available on a rotator set at 100 rpm. Avoid direct exposure to intense light. The point, at which the substrate reaction is stopped, is often determined by the ELISA reader. Many ELISA readers record absorbance only up to 2.0 O.D. Therefore, the color development within individual microwells must be watched by the person running the assay and the substrate reaction stopped before positive wells are no longer properly detectable. Stop the enzyme reaction by quickly pipetting 100 μL of Stop Solution into each well, including the blank wells. It is important that the Stop Solution is spread quickly and uniformly throughout the microwells to completely inactivate the enzyme. Results must be read immediately after the Stop Solution is added or within one hour if the microwell strips are stored at 2 - 8°C in the dark. Read absorbance of each microwell on a spectrophotometer using 450 nm as the primary wave length.

**Table: Growth inhibition percenttages (GI %) for compounds 2a-e in vitro subpanel tumor cell lines at 10 μM concentration . ( - = GI< 5% )**

| **Subpanel cancer cell Lines** | | **2a** | **2b** | **2c** | **2d** | **2e** |
| --- | --- | --- | --- | --- | --- | --- |
| **Leukemia** | **CCRF-CEM** | **11.33** | **-** | **5.28** | **8.99** | **8.16** |
|  | **HL-60(TB)** | **6.26** | **18.99** | **12.49** | **6.19** | **12.81** |
|  | **K-562** | **9.72** | **-** | **-** | **5.5** | **-** |
|  | **MOLT-4** | **7.63** | **-** | **-** | **8.77** | **-** |
|  | **RPMI-8226** | **26.63** | **-** | **11.73** | **9.16** | **9.34** |
|  | **SR** | **5.8** | **-** | **8.98** | **-** | **-** |
| **Non-Small Cell Lung Cancer** | **A549/ATCC** | **5.98** | **-** | **-** | **5.11** | **-** |
|  | **EKVX** | **54.36** | **-** | **6.27** | **13.9** | **6.46** |
|  | **HOP-62** | **22.87** | **6.93** | **18.83** | **9.35** | **13.63** |
|  | **HOP-92** | **25.13** | **-** | **31.61** | **13.42** | **20.37** |
|  | **NCI-H226** | **32.38** | **-** | **7.15** | **16.64** | **10.65** |
|  | **NCI-H23** | **10.27** | **-** | **-** | **5.37** | **-** |
|  | **NCI-H322M** | **16.06** | **-** | **7.11** | **12.16** | **-** |
|  | **NCI-H460** | **-** | **-** | **-** | **-** | **-** |
|  | **NCI-H522** | **10.28** | **6.79** | **8.32** | **5.29** | **7.32** |
| **Colon Cancer** | **COLO 205** | **-** | **-** | **-** | **-** | **-** |
|  | **HCC-2998** | **-** | **-** | **-** | **-** | **-** |
|  | **HCT-116** | **-** | **-** | **7.78** | **-** | **-** |
|  | **HCT-15** | **-** | **-** | **-** | **-** | **-** |
|  | **HT29** | **-** | **-** | **-** | **-** | **7.95** |
|  | **KM12** | **-** | **-** | **-** | **-** | **-** |
|  | **SW-620** | **-** | **-** | **-** | **-** | **-** |
| **CNS Cancer** | **SF-268** | **-** | **-** | **-** | **-** | **-** |
|  | **SF-295** | **6.24** | **-** | **-** | **5.21** | **6.55** |
|  | **SF-539** | **12.97** | **-** | **-** | **14.45** | **7.46** |
|  | **SNB-19** | **11.36** | **-** | **-** | **7.35** | **5.52** |
|  | **SNB-75** | **-** | **-** | **11.82** | **-** | **15.56** |
|  | **U251** | **8.86** | **5.31** | **5.38** | **-** | **-** |
| **Melanoma** | **LOX IMVI** | **15.6** | **5.38** | **11.28** | **11.21** | **9.22** |
|  | **MALME-3M** | **11.3** | **-** | **-** | **13.27** | **14.01** |
|  | **M14** | **-** | **-** | **-** | **-** | **-** |
|  | **MDA-MB-435** | **-** | **-** | **-** | **-** | **-** |
|  | **SK-MEL-2** | **-** | **-** | **-** | **-** | **-** |
|  | **SK-MEL-28** | **-** | **-** | **-** | **-** | **-** |
|  | **SK-MEL-5** | **11.91** | **-** | **-** | **5.89** | **-** |
|  | **UACC-257** | **-** | **-** | **-** | **-** | **-** |
|  | **UACC-62** | **19.52** | **-** | **-** | **15.74** | **-** |
| **Ovarian Cancer** | **IGROV1** | **37.94** | **-** | **-** | **7.41** | **-** |
|  | **OVCAR-3** | **-** | **-** | **-** | **-** | **-** |
|  | **OVCAR-4** | **-** | **-** | **-** | **-** | **-** |
|  | **OVCAR-5** | **-** | **-** | **-** | **-** | **-** |
|  | **OVCAR-8** | **-** | **-** | **-** | **-** | **-** |
|  | **NCI/ADR-RES** | **8.35** | **-** | **-** | **5.32** | **-** |
|  | **SK-OV-3** | **19.96** | **-** | **7.83** | **6.14** | **-** |
| **Renal Cancer** | **786-0** | **-** | **-** | **-** |  | **-** |
|  | **A498** | **-** | **-** | **-** | **-** | **-** |
|  | **ACHN** | **7.82** | **-** | **-** | **7.6** | **-** |
|  | **CAKI-1** | **21.48** | **8.89** | **15.55** | **15.1** | **16.47** |
|  | **RXF 393** | **-** | **-** | **-** | **-** | **-** |
|  | **SN12C** | **8.07** | **-** | **-** | **15.23** | **6.46** |
|  | **TK-10** | **-** | **-** | **-** | **-** | **-** |
|  | **UO-31** | **38.33** | **19.01** | **34.36** | **34.98** | **37.44** |
| **Prostate Cancer** | **PC-3** | **15.24** | **4.29** | **13.36** | **11.04** | **13.35** |
|  | **DU-145** | **-** | **-** | **-** | **-** | **-** |
| **Breast Cancer** | **MCF7** | **17.31** |  | **-** | **6.43** | **-** |
|  | **MDA-MB 231/ATCC** | **26.93** | **-** | **7** | **1043** | **-** |
|  | **HS 578T** | **8.26** | **-** | **-** | **-** | **6.91** |
|  | **BT-549** | **6.06** | **-** | **-** | **-** | **-** |
|  | **T-47D** | **16.92** | **6.75** | **9.18** | **-** | **12.21** |
|  | **MDA-MB-468** | **-** | **-** | **-** | **-** | **-** |

**Table: Growth inhibition percenttages (GI %) for compounds 4a-j in vitro subpanel tumor cell lines at 10 μM concentration . ( - = GI< 5% )**

| **Subpanel cancer cell Lines** | **% Growth Inhibition (GI %)^a^** | | | | | | | | | |
| --- | --- | --- | --- | --- | --- | --- | --- | --- | --- | --- |
|  | **4a** | **4b** | **4c** | **4d** | **4e** | **4f** | **4g** | **4h** | **4i** | **4j** |
| **Leukemia** | | | | | | | | | | |
| **CCRF-CEM** | **7.82** | **15.96** | **-** | **52.2** | **91.77** | **-** | **6.98** | **-** | **-** | **-** |
| **HL-60(TB)** | **-** |  | **-** | **-** | **15.25** | **-** | **-** | **-** | **5.88** | **7.02** |
| **K-562** | **-** | **9.72** | **-** | **29.68** | **64.96** | **-** | **-** | **-** | **7.48** | **-** |
| **MOLT-4** | **-** | **16.13** | **-** | **34.27** | **81.48** | **-** |  | **-** | **-** | **-** |
| **RPMI-8226** | **11.16** | **-** | **-** | **15.74** | **25.73** | **-** | **14.92** | **-** | **-** | **-** |
| **SR** | **12.74** | **40.26** | **12.99** | **71.59** | **95.89** | **12.23** | **11.39** | **-** | **-** | **7.14** |
| **Non-small cell lung cancer** | | | | | | | | | | |
| **A549/ATCC** | **-** | **-** | **-** | **-** | **-** | **-** | **-** | **-** | **-** | **-** |
| **EKVX** |  | **-** |  | **10.09** | **27.9** | **-** | **7.01** | **-** | **-** | **6.05** |
| **HOP-62** | **7.49** | **9.34** |  | **24.61** | **36.6** | **8.55** | **6.86** | **-** | **-** | **5.55** |
| **HOP-92** | **-** | **-** | **-** | **-** | **102.47** | **79.85** | **93.87** | **38.45** | **36.73** | **-** |
| **NCI-H226** | **14.68** | **15.93** | **8.4** | **17.59** | **37.66** | **10.88** | **9.91** | **-** | **-** | **5.42** |
| **NCI-H23** | **-** | **-** | **6** | **16.94** | **30.12** | **7.19** | **-** | **-** | **-** | **-** |
| **NCI-H322M** | **6.78** | **-** |  | **8.41** | **22.33** | **-** | **-** | **-** | **-** | **-** |
| **NCI-H460** | **-** | **-** | **-** | **6.52** | **22.06** | **-** | **-** | **-** | **-** | **-** |
| **NCI-H522** | **18.89** | **27.9** | **11.83** | **44.81** | **94.93** | **13.89** | **10.61** | **-** | **-** | **-** |
| **Colon cancer** | | | | | | | | | | |
| **COLO 205** | **-** | **-** | **-** | **-** | **14.48** | **-** |  | **-** | **-** | **-** |
| **HCC-2998** | **-** | **-** | **-** | **-** | **26.59** | **-** | **-** | **-** | **-** | **-** |
| **HCT-116** | **-** | **-** | **-** | **19.07** | **41.54** | **-** |  | **-** | **-** | **-** |
| **HCT-15** | **-** | **-** |  | **-** | **8.8** | **-** |  | **-** | **-** | **-** |
| **HT29** | **-** | **-** | **-** | **-** | **-** | **-** | **-** | **-** | **-** | **-** |
| **KM12** | **-** |  | **-** | **16.67** | **46.34** | **-** |  | **-** | **-** | **-** |
| **SW-620** | **-** | **-** | **-** | **7.13** | **24.34** | **-** |  | **-** | **-** | **-** |
| **CNS Cancer** | | | | | | | | | | |
| **SF-268** | **14** | **13.29** | **-** | **28.71** | **42.68** | **-** |  | **-** | **-** | **-** |
| **SF-295** | **5.93** | **11.05** | **10.47** | **9.48** | **8.09** | **-** |  | **-** | **-** | **-** |
| **SF-539** | **8.96** | **16.89** | **5.07** | **33.03** | **58.41** | **-** | **-** | **-** | **-** | **7.41** |
| **SNB-19** | **9.13** | **12.64** | **5.89** | **24.88** | **53.98** | **-** |  | **-** | **-** | **-** |
| **SNB-75** | **10.07** | **12.24** | **-** | **35.44** | **78.02** | **12.95** | **8.08** | **-** | **5.56** | **17.18** |
| **U251** | **7.57** | **13** | **-** | **26.33** | **62.09** | **-** | **-** | **-** | **-** | **-** |
| **Melanoma** | | | | | | |  | | | |
| **LOX IMVI** | **5.22** | **12.96** |  | **29.02** | **61.77** | **10.85** | **5.11** | **-** | **-** | **-** |
| **MALM-3M** | **30.08** | **16.36** | **10.55** | **44.47** | **78.31** | **9.22** | **16.68** | **-** | **19.4** | **14.42** |
| **M14** |  | **-** | **-** | **20.69** | **26.95** | **8.95** |  | **-** | **-** | **-** |
| **MDA-MB-435** | **-** | **8.01** | **-** | **27.28** | **95.61** | **-** | **-** | **-** | **-** | **-** |
| **SK-MEL-2** | **11.99** | **7.35** | **-** | **27.17** | **80.05** | **-** | **-** | **-** | **-** | **-** |
| **SK-MEL-28** | **6.43** | **-** | **-** | **24.72** | **65.26** | **-** | **-** | **-** | **-** | **-** |
| **SK-MEL-5** | **8.23** | **9.87** | **-** | **21.78** | **45.01** | **-** |  | **-** | **-** | **-** |
| **UACC-257** | **-** | **-** | **-** | **4.52** | **33.71** | **-** | **-** | **-** | **-** | **-** |
| **UACC-62** | **8.54** | **19.16** | **5.15** | **41.81** | **88.64** | **6.43** | **8.46** | **-** | **6.04** | **6.6** |
| **Ovarian cancer** | | | | | | | | | | |
| **IGROV1** | **-** | **-** | **-** | **14.64** | **43.05** | **-** | **-** | **-** | **-** | **-** |
| **OVCAR-3** |  |  | **-** | **23.4** | **43.75** | **-** |  | **-** | **-** | **-** |
| **OVCAR-4** | **11.41** | **11.41** | **-** | **24.19** | **32.26** | **7.33** | **5.43** | **-** | **-** | **-** |
| **OVCAR-5** | **-** | **-** | **-** | **11.53** | **18.58** | **-** |  | **-** | **-** | **-** |
| **OVCAR-8** | **-** |  | **-** | **6.15** | **19.48** | **-** | **-** | **-** | **-** | **-** |
| **NCI/ADR-RES** | **-** | **-** | **--** | **5.85** | **9.87** | **-** | **-** | **-** | **-** | **-** |
| **SK-OV-3** | **-** | **-** | **-** | **24.15** | **30.84** | **-** | **5.67** | **-** | **5.11** | **4.08** |
| **Renal cancer** | | | | | | | | | | |
| **786-0** | **11.64** |  | **-** | **14.45** | **14.06** | **-** | **6.84** | **-** | **-** | **-** |
| **A498** |  | **5.79** | **7.1** | **-** | **-** | **-** | **-** | **-** | **-** | **-** |
| **ACHN** | **-** | **-** | **-** | **14.13** | **19.01** | **5.83** | **-** | **-** | **-** | **-** |
| **CAKI-1** | **5.51** | **5.35** | **-** | **21.46** | **23.27** | **16.09** | **10.32** | **-** | **10.76** | **14.7** |
| **RXF 393** | **-** | **9.76** | **6.12** | **-** | **31.36** | **11.06** | **6.27** | **-** | **-** | **-** |
| **SN12C** | **-** | **5.72** | **8.13** | **26.99** | **38.8** | **-** | **7.44** | **-** | **-** | **-** |
| **TK-10** | **-** | **-** | **-** | **-** | **-** | **-** | **-** | **-** | **-** | **-** |
| **UO-31** | **22.62** | **11.71** | **12.92** | **37.72** | **45.23** | **45.2** | **32.11** | **22.55** | **29.06** | **39.26** |
| **Prostate cancer** | | | | | | | | | | |
| **PC-3** | **8.46** | **9.56** | **-** | **18.48** | **41.65** | **12.38** | **11.24** | **-** | **-** | **-** |
| **DU-145** | **-** | **-** | **-** | **10.93** | **22.12** | **-** | **-** | **-** | **-** | **-** |
| **Breast cancer** | | | | | | | | | | |
| **MCF7** | **13.94** | **16.1** | **6.69** | **36.34** | **76.85** | **5.99** | **9.62** | **5.52** | **8.38** | **-** |
| **MDA-MB-231/ATCC** | **17.7** | **18.81** | **8.98** | **51.7** | **68.55** | **12.53** | **8.94** | **4.98** | **12.87** | **17** |
| **HS 578T** | **19.45** | **14.43** |  | **36.26** | **71.63** | **12.29** | **7.48** | **-** | **6.25** | **11.74** |
| **BT-549** | **34.27** | **-** | **-** | **56.27** | **62.28** | **-** | **11.63** | **-** | **-** | **-** |
| **T-47D** | **24.69** | **27.29** | **12.08** | **54.01** | **82.53** | **-** |  | **-** | **6.23** | **7.76** |
| **MDA-MB-468** | **24.78** | **41.67** | **7.22** | **47.22** | **110.84** | **-** | **16.84** | **-** | **-** | **-** |


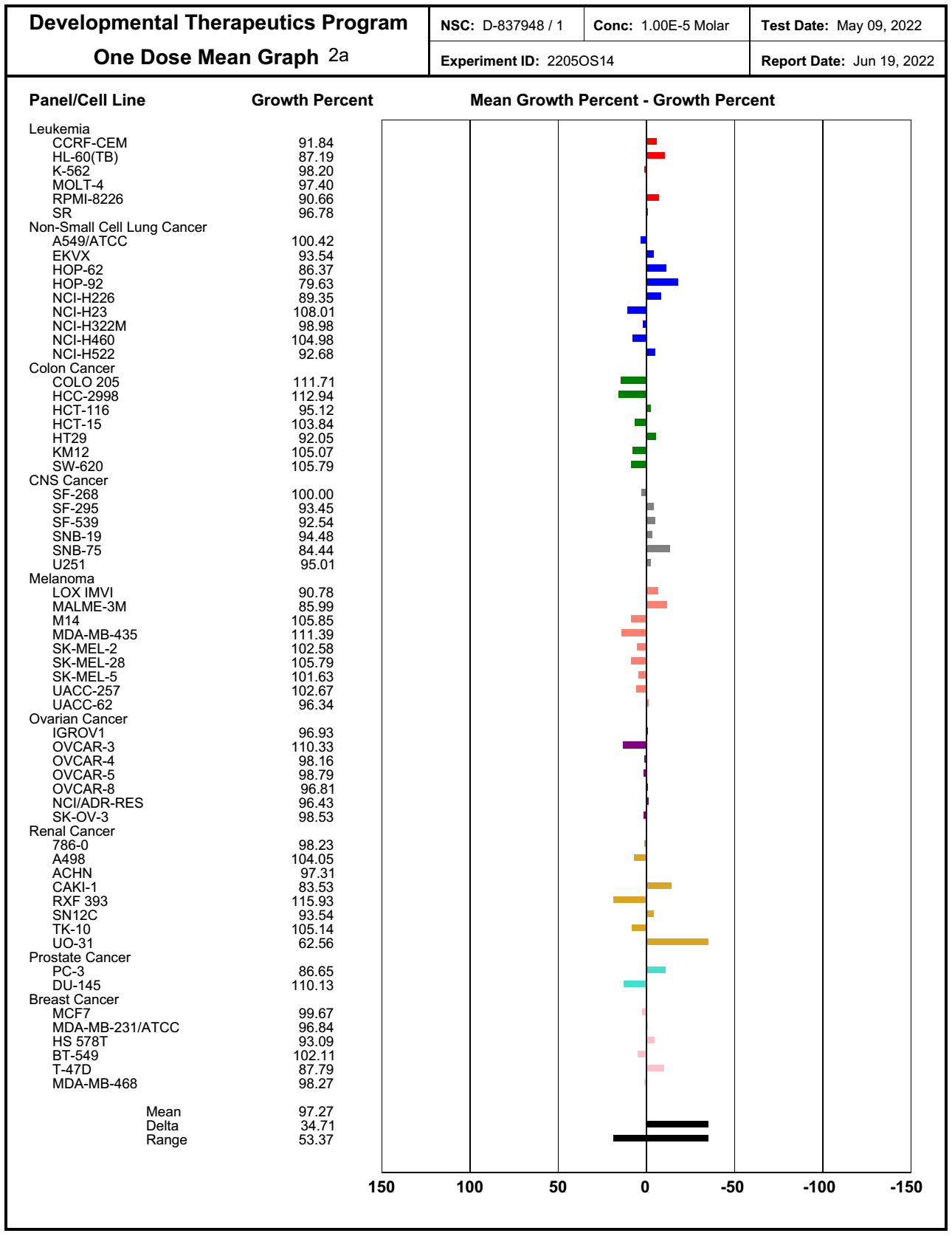


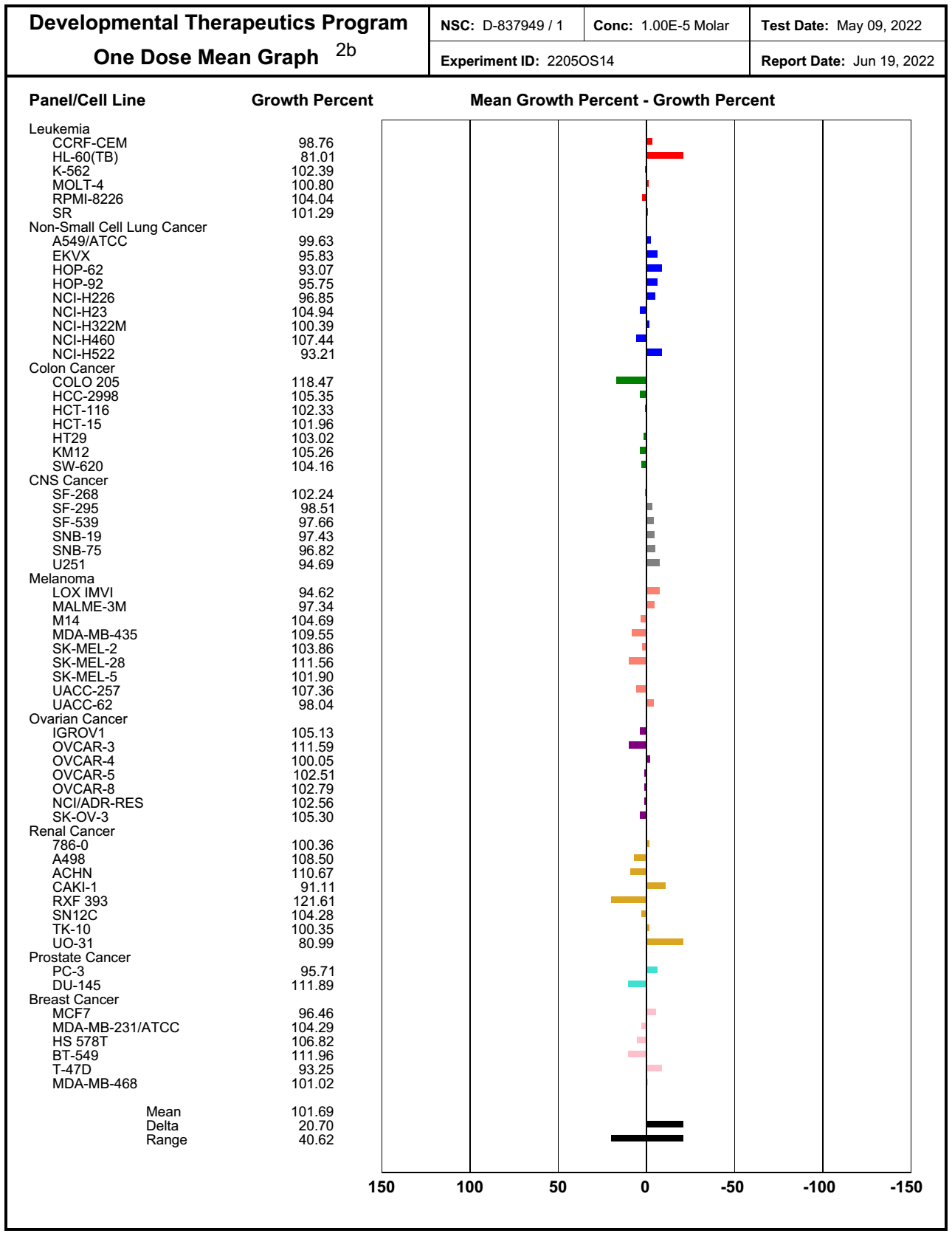


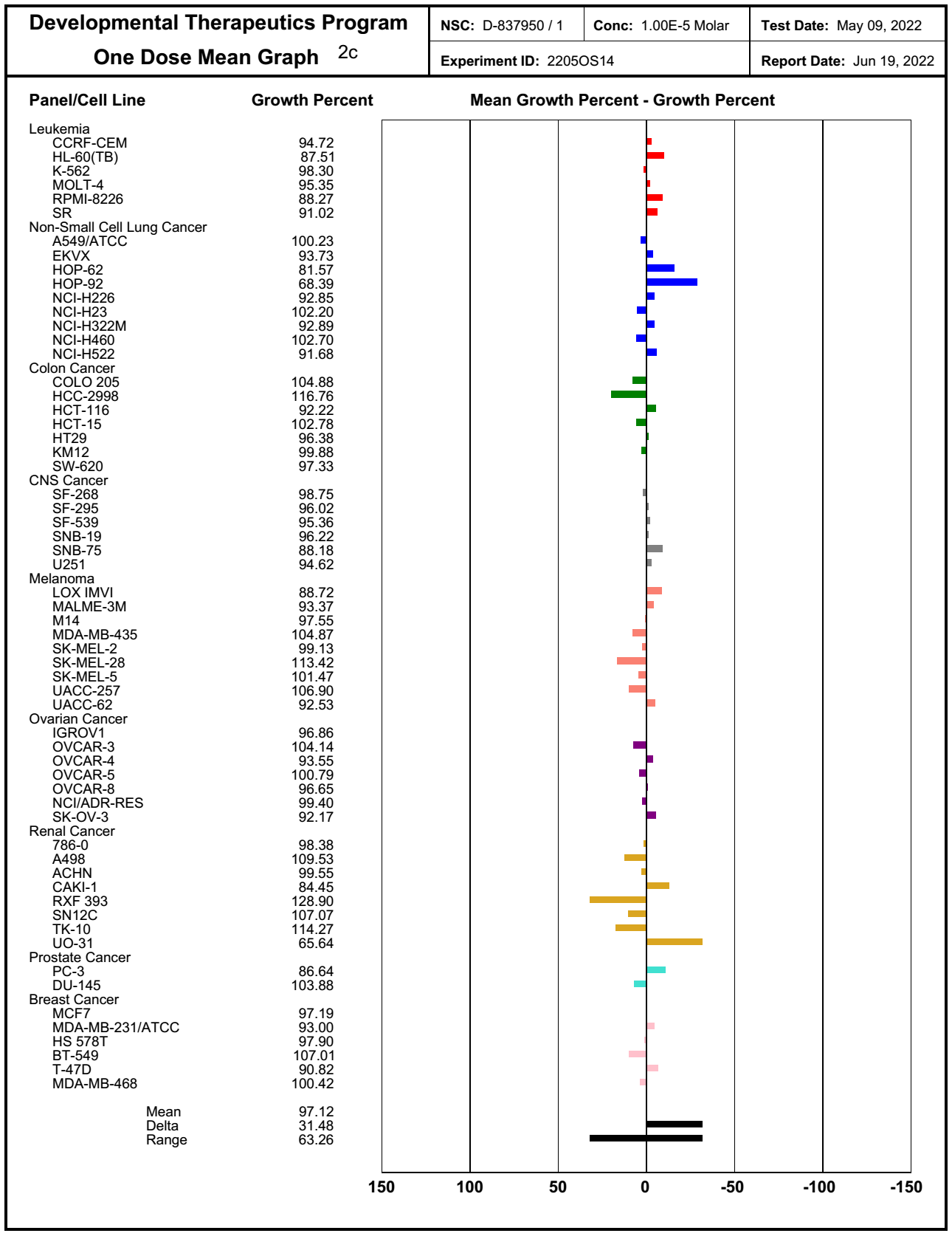


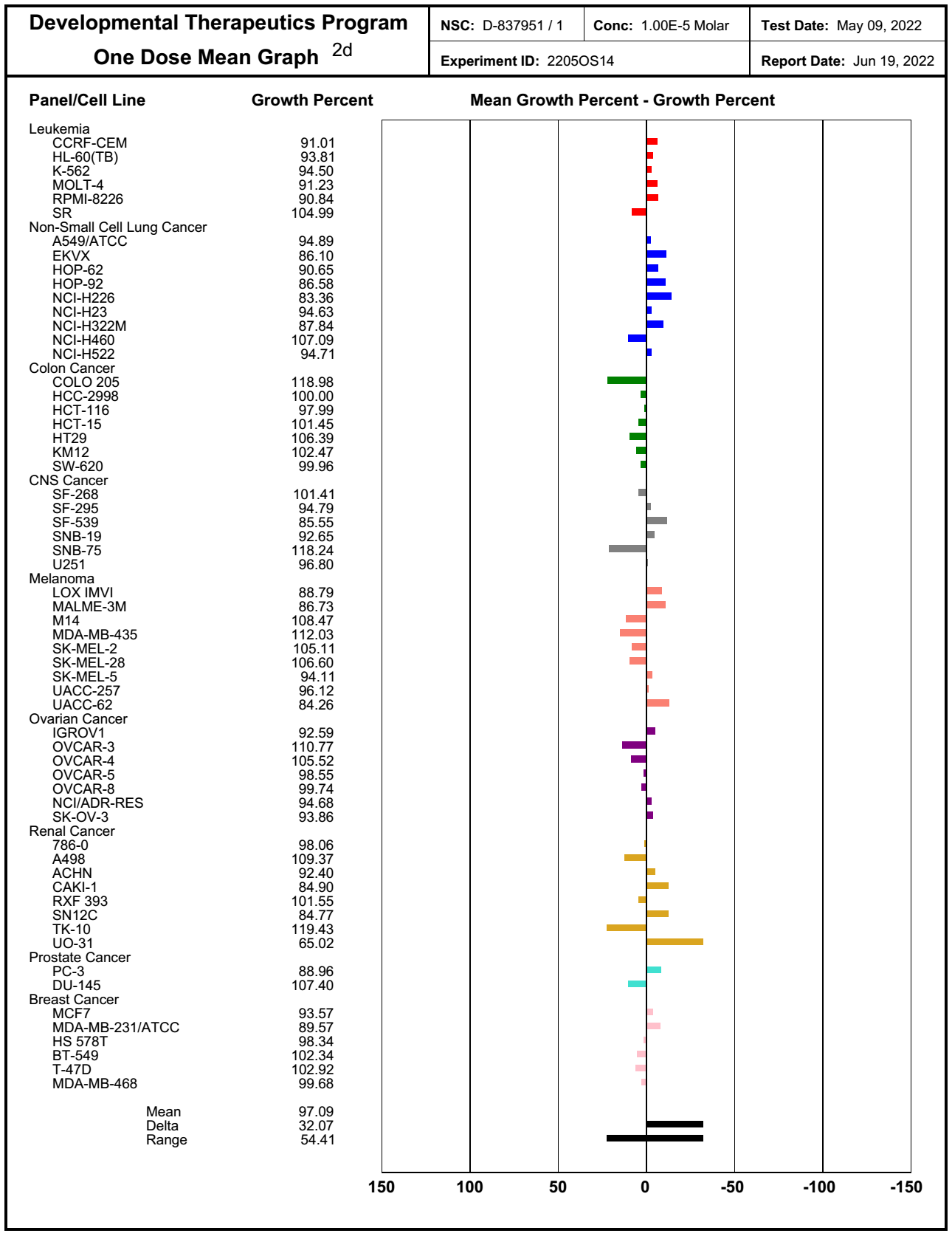


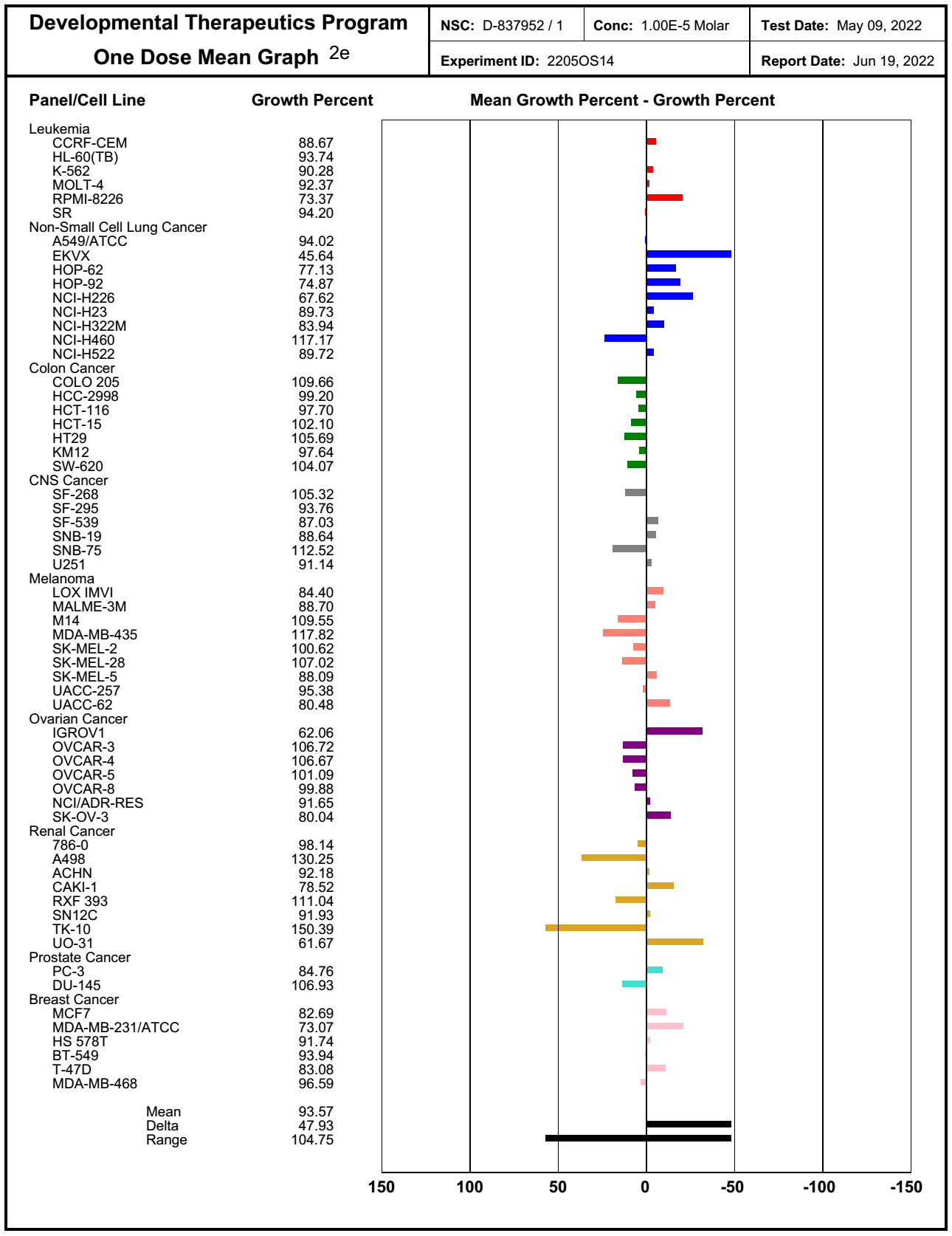


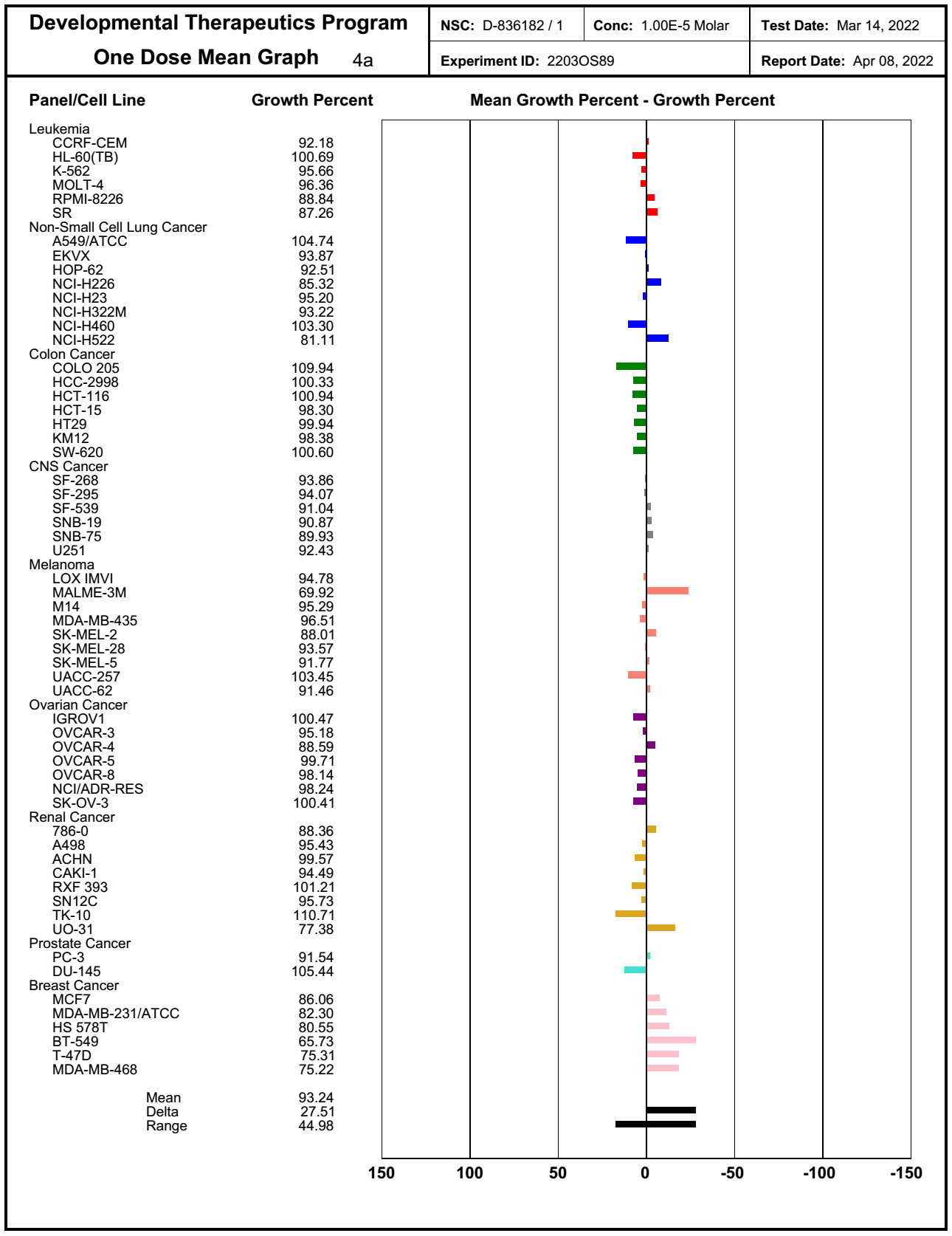


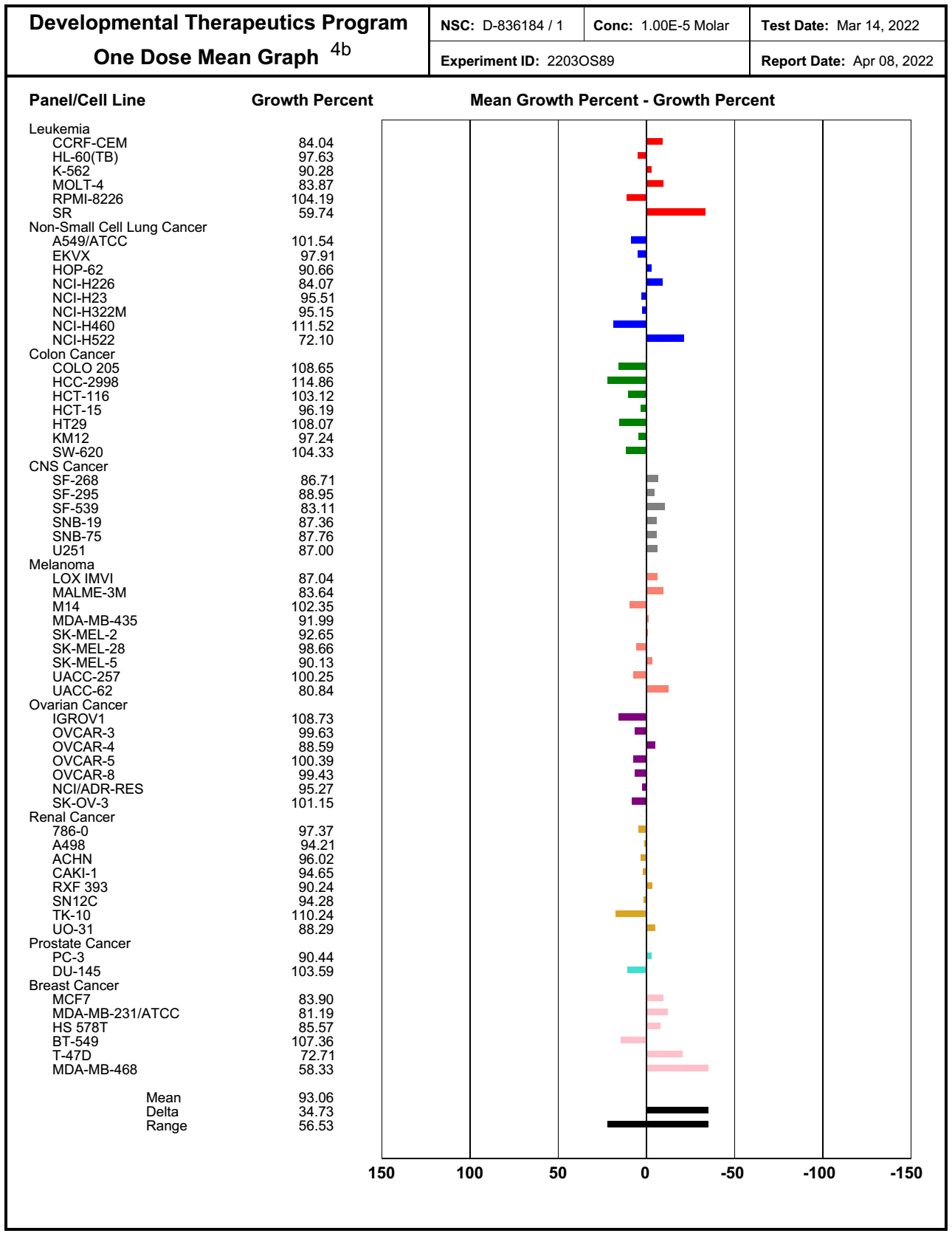


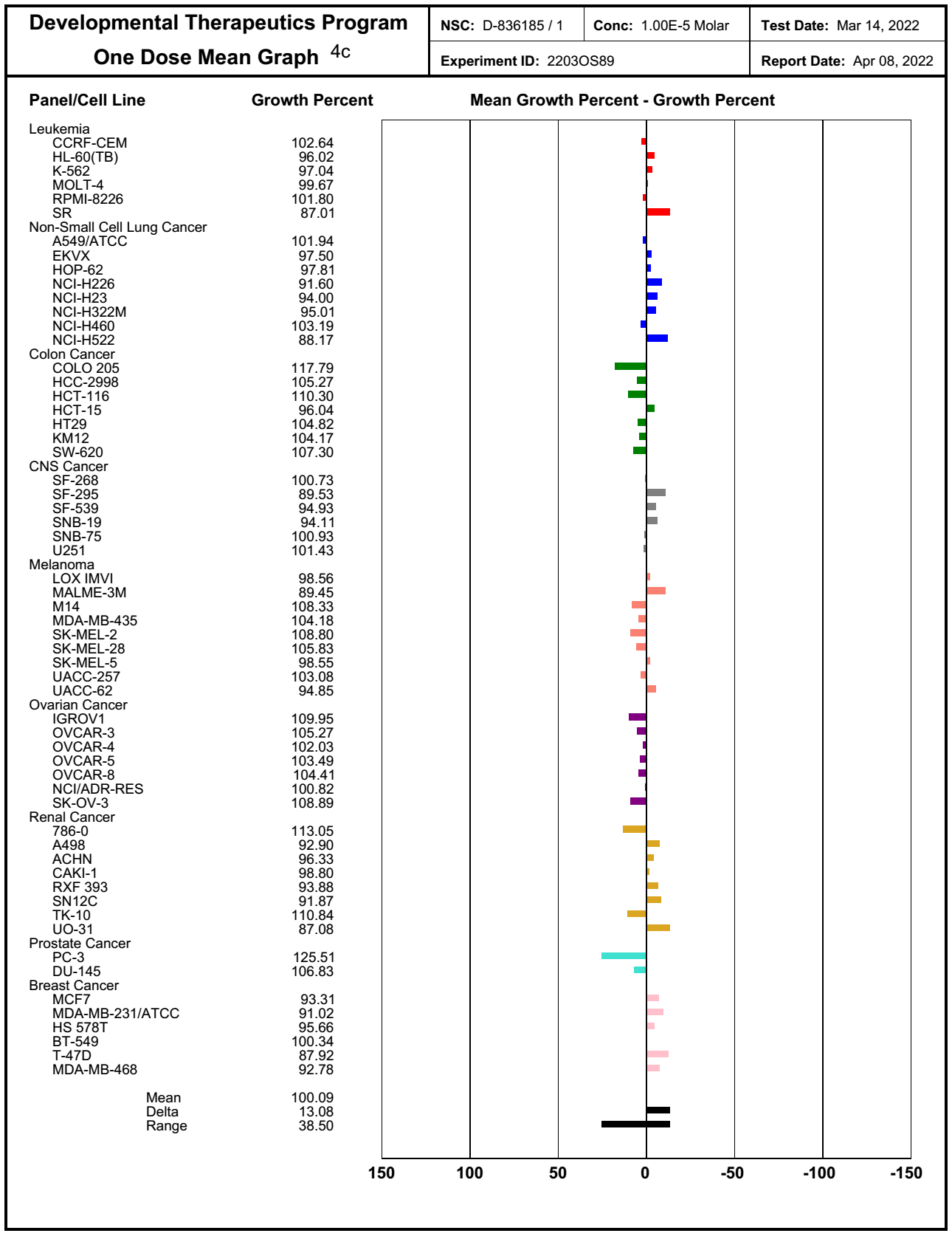


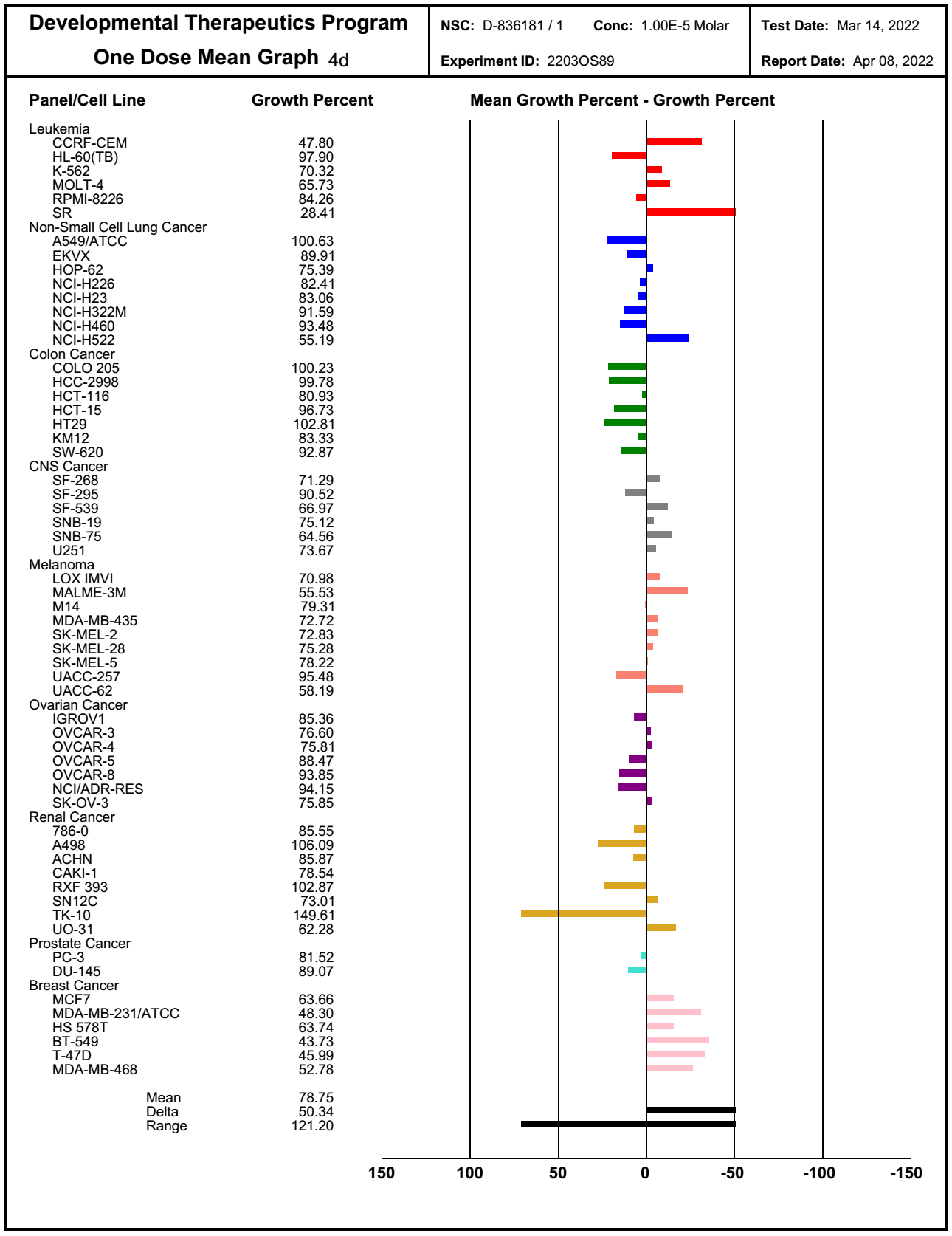


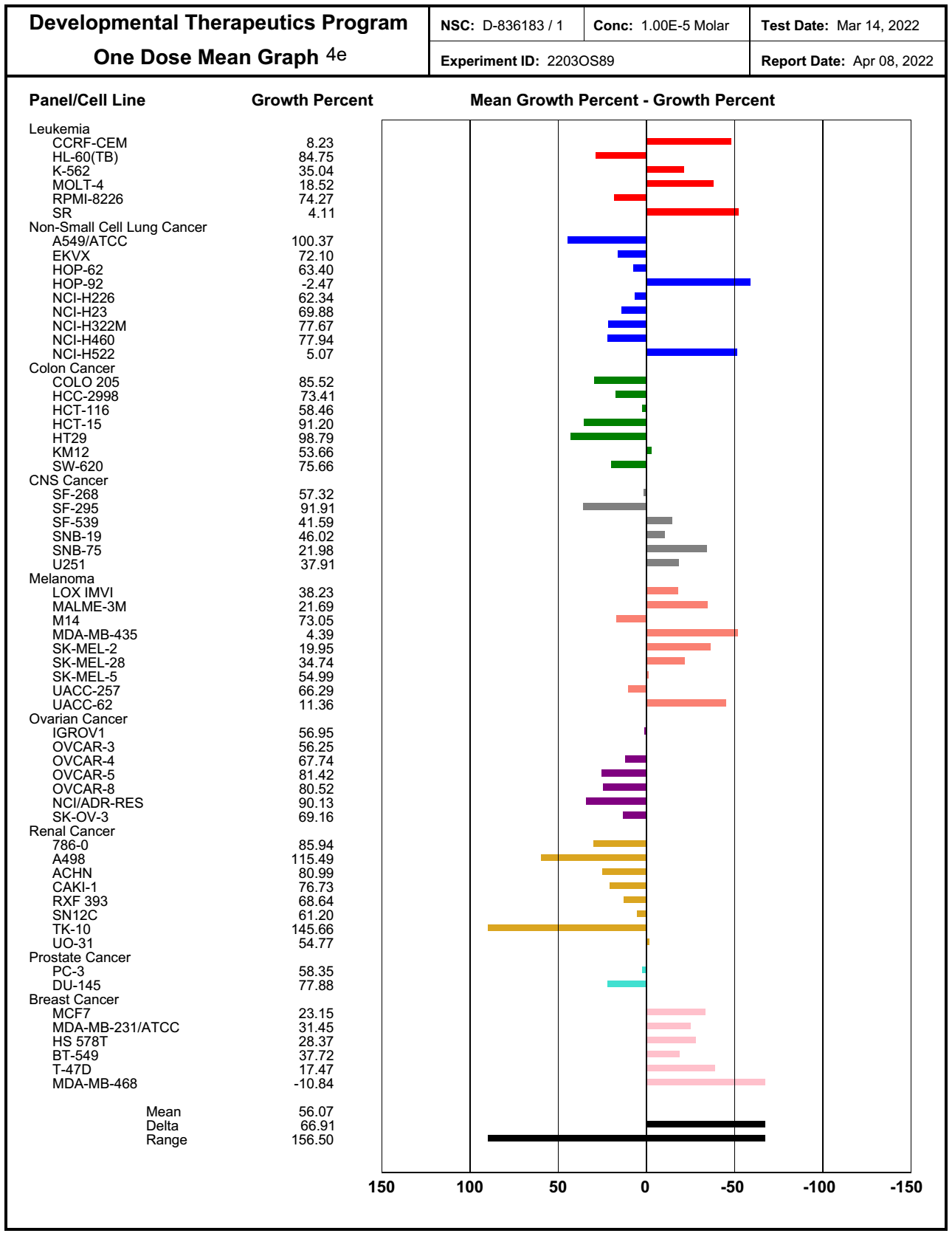


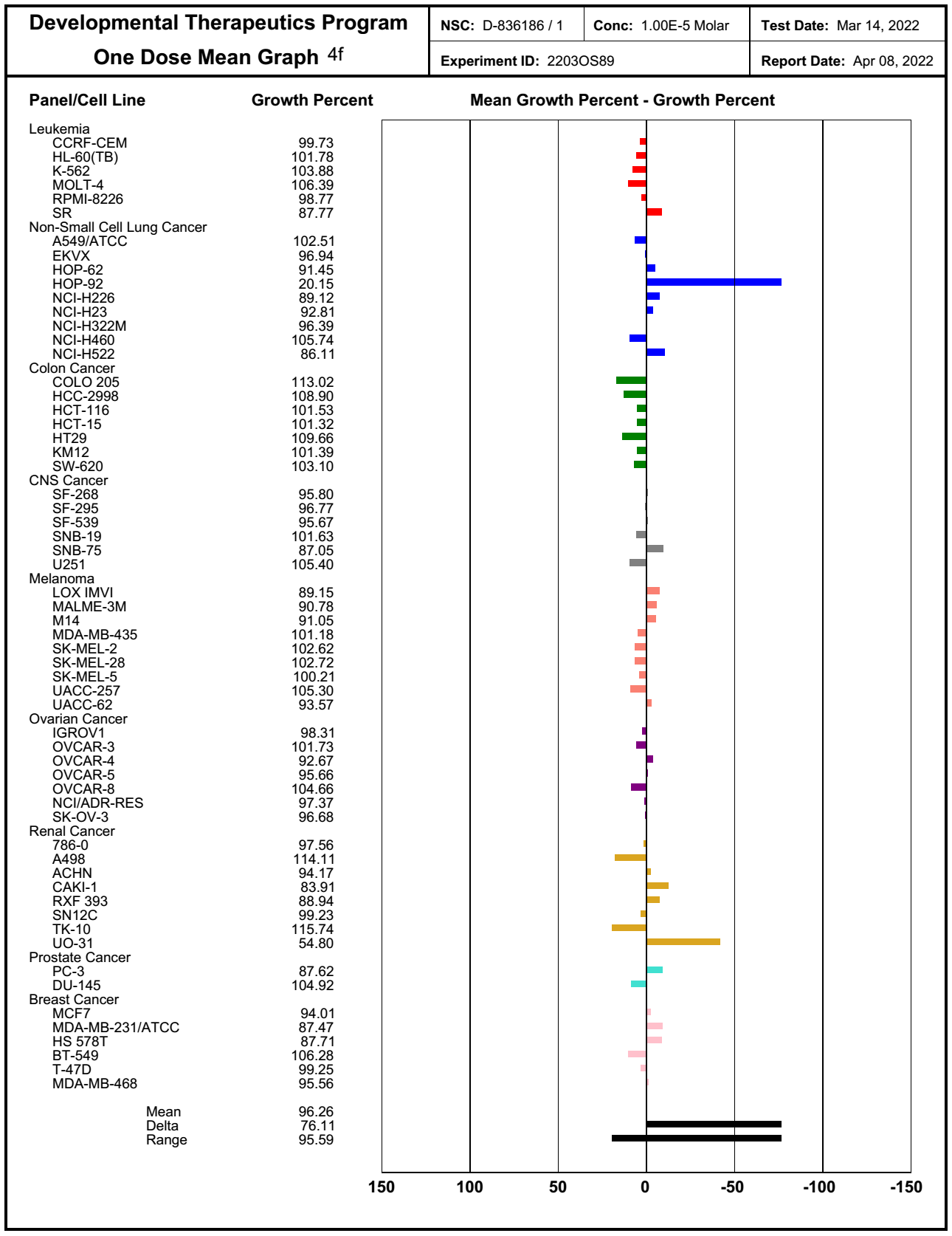


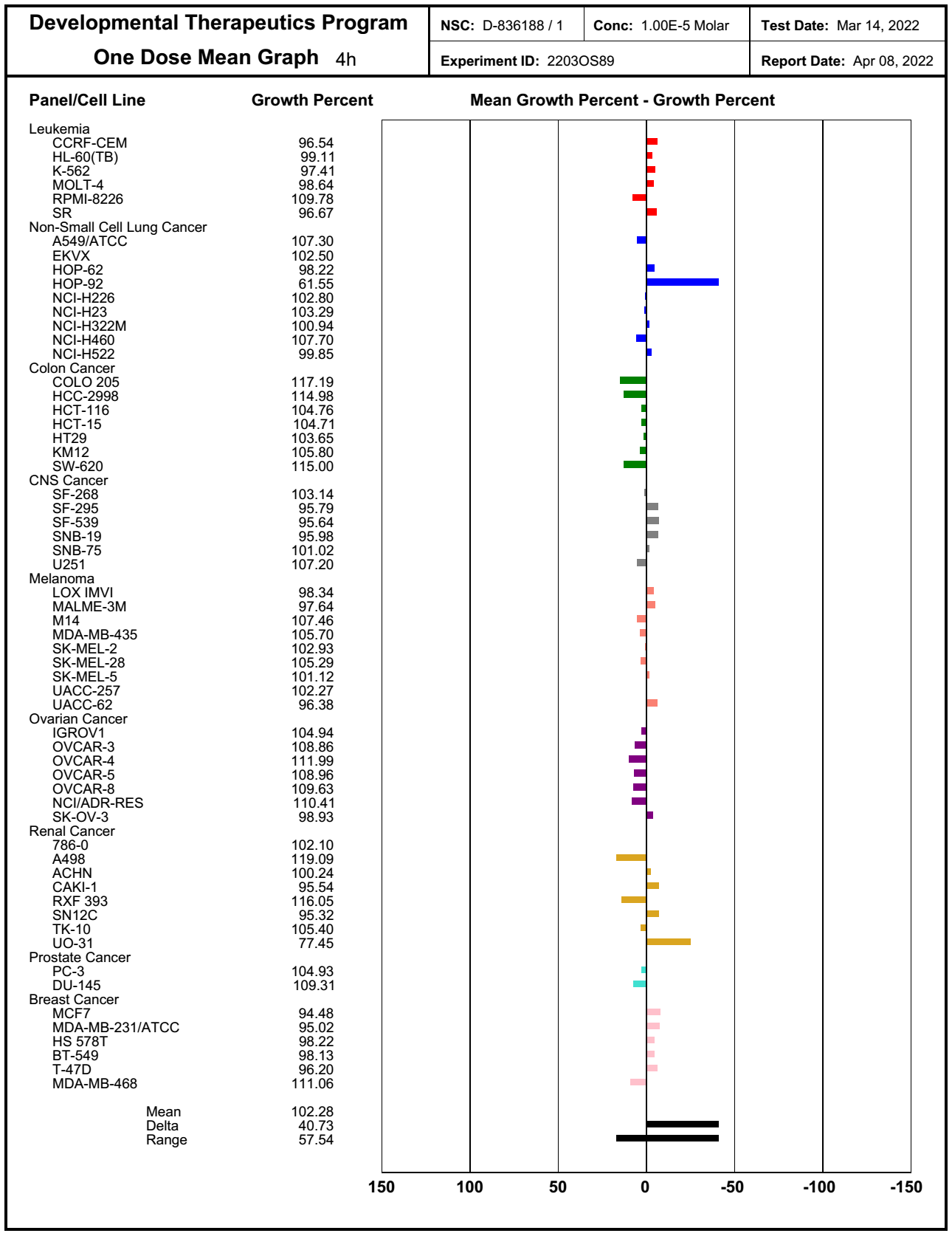


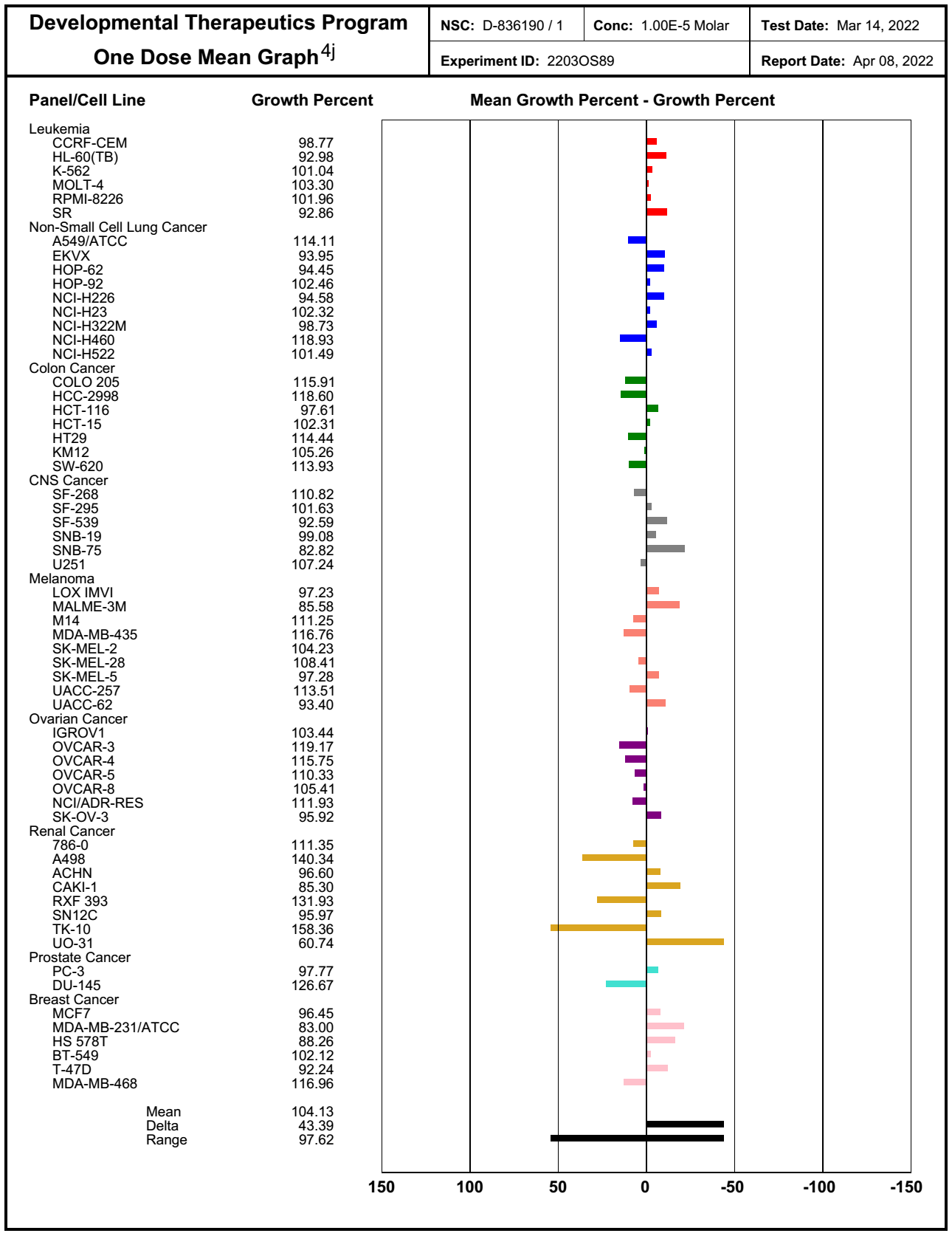

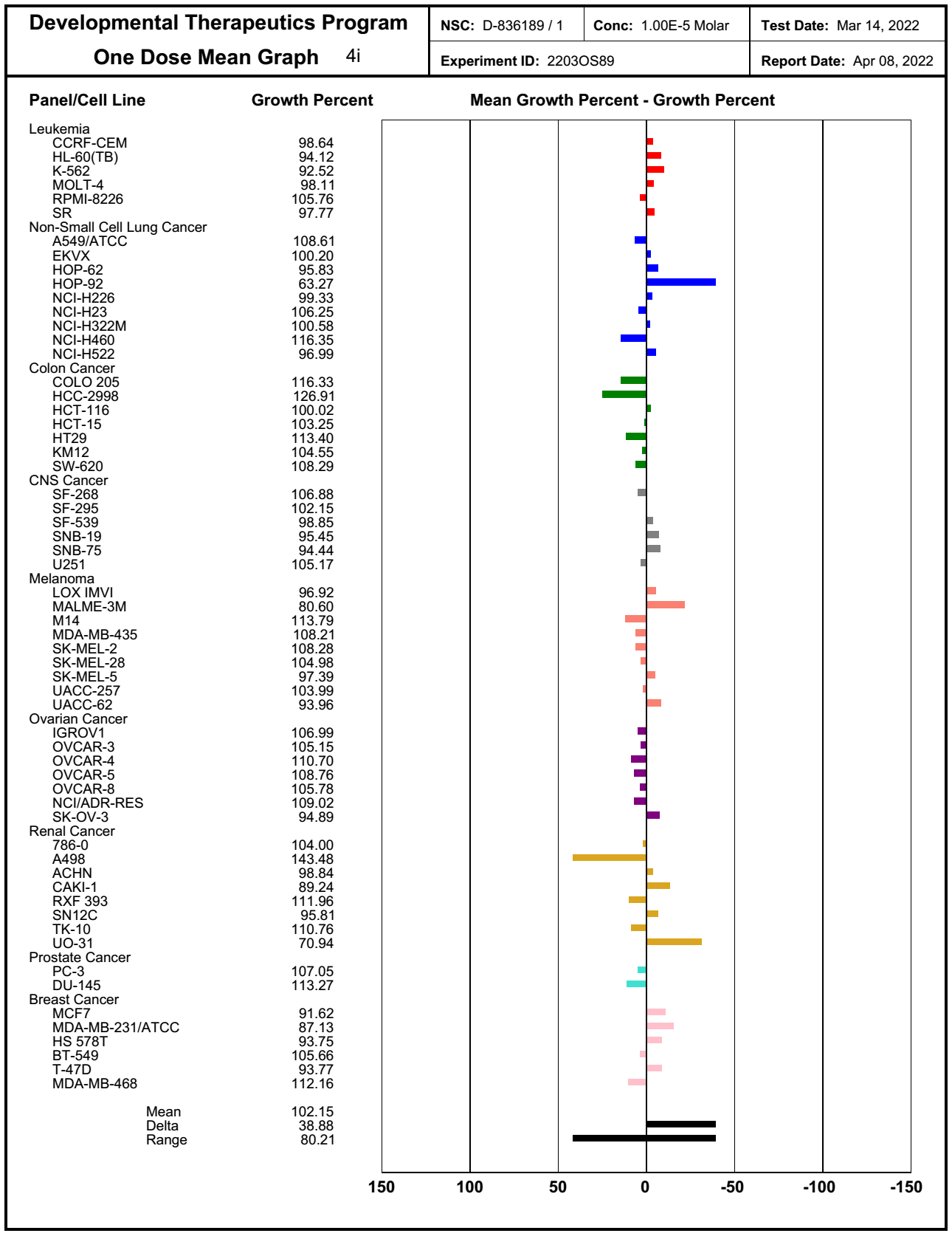


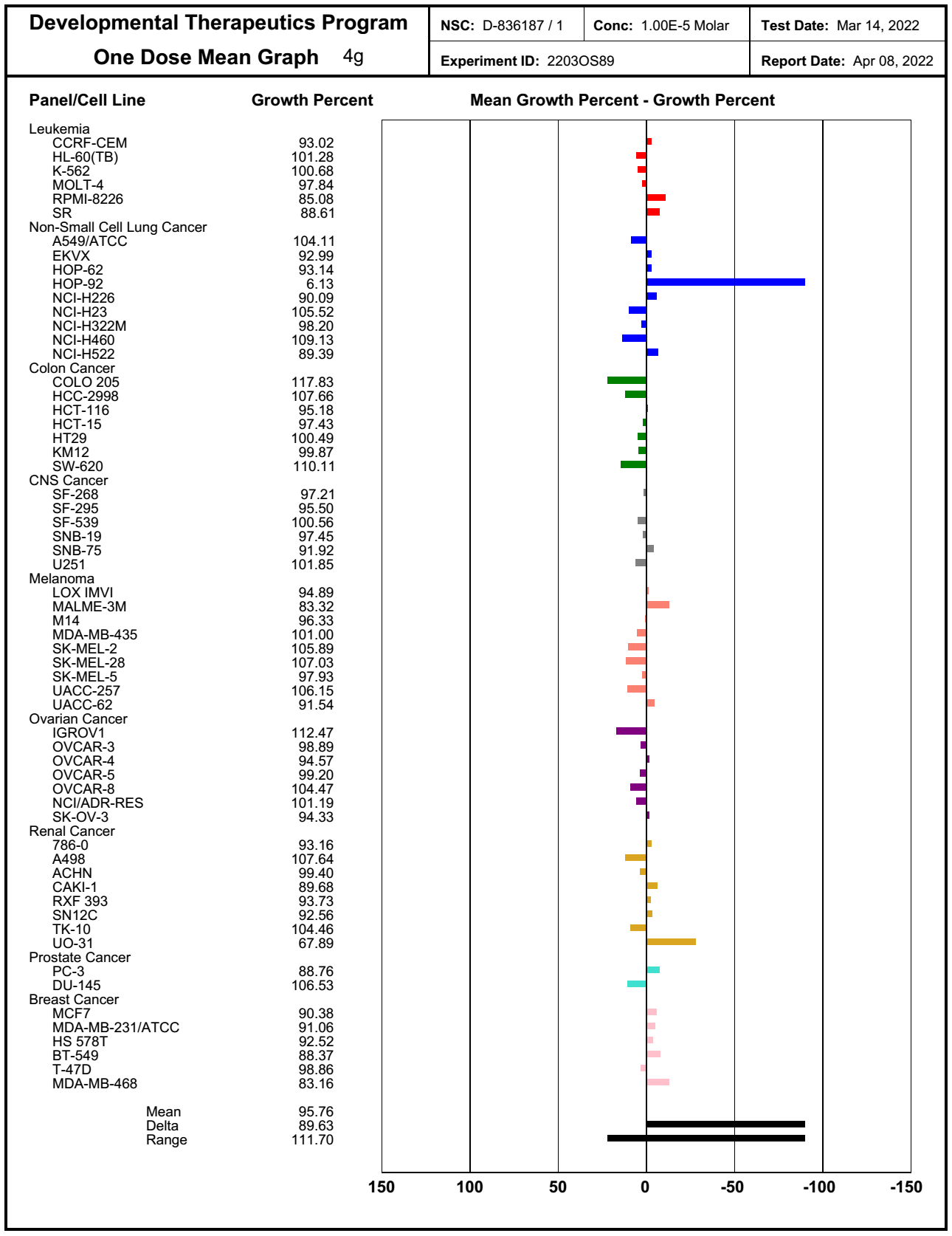


**MTT assay**

| Ser | **cytotoxicity**  **IC50**  **uM** | | | | SD | | |
| --- | --- | --- | --- | --- | --- | --- | --- |
|  | code | **T-47D** | **HOP-92** | **MOLT-4** | | WI38 |  |
| 1 | **Ws4** | **13.85**±0.48 | **36.52**±1.46 | **7.24**±0.29 | | **62.46**±2.49 |  |
| 2 | **WS5** | **8.62**±0.34 | **4.982**±0.2 | **8.023**±0.31 | | **29.62**±1.18 |  |
| 3 | **ws6** | **28.56**±0.99 | **24.93**±1**.9** | **16.31**±0.65 | | **42.14**±1.68 |  |
| 4 | **Ws7** | **9.71**±0.34 | **14.72**±0.66 | **6.17**±0.25 | | **37.57**±1.5 |  |
| *** | **Staurosporine** | **4.94**±0.19 | **3.172**±0.19 | **5.856**±0.23 | | **17.54**±1.5 |  |

| researcher |  | assay |  |  | Date |  |  | cells |  |  |
| --- | --- | --- | --- | --- | --- | --- | --- | --- | --- | --- |
| Dr.Mohamed Hawas | | MTT |  |  | 18/10/2023 | |  | **T-47D** | **HOP-92** | **MOLT-4** |

|  | **Blank** | **CC** | **Sample No. Ws4/HOP-92** | | | | | **Sample No. ws6/HOP-92** | | | | |
| --- | --- | --- | --- | --- | --- | --- | --- | --- | --- | --- | --- | --- |
|  | **1** | **2** | **3** | **4** | **5** | **6** | **7** | **8** | **9** | **10** | **11** | **12** |
| A | B | C | 100uM | 25uM | 6.3uM | 1.6uM | 0.4uM | 100uM | 25uM | 6.3uM | 1.6uM | 0.4uM |
| B | B | C | 100uM | 25uM | 6.3uM | 1.6uM | 0.4uM | 100uM | 25uM | 6.3uM | 1.6uM | 0.4uM |
| C | B | C | 100uM | 25uM | 6.3uM | 1.6uM | 0.4uM | 100uM | 25uM | 6.3uM | 1.6uM | 0.4uM |

| ROBONIK P2000 Eia reader | | | |  |  |  |  |  |  |  |  |  |
| --- | --- | --- | --- | --- | --- | --- | --- | --- | --- | --- | --- | --- |
| Wave length: 450 nm | | | |  |  |  |  |  |  |  |  |  |
| Reference: 630 nm | | | |  |  |  |  |  |  |  |  |  |
|  | **1** | **2** | **3** | **4** | **5** | **6** | **7** | **8** | **9** | **10** | **11** | **12** |
|  |  |  |  |  |  |  |  |  |  |  |  |  |
| A | 0.001 | 0.529 | 0.213 | 0.269 | 0.328 | 0.386 | 0.427 | 0.182 | 0.261 | 0.328 | 0.372 | 0.422 |
| B | 0.003 | 0.494 | 0.196 | 0.254 | 0.314 | 0.371 | 0.434 | 0.177 | 0.254 | 0.311 | 0.359 | 0.413 |
| C | 0.001 | 0.485 | 0.227 | 0.263 | 0.331 | 0.388 | 0.424 | 0.174 | 0.266 | 0.331 | 0.391 | 0.422 |
| mean | 0.002 | 0.503 | 0.212 | 0.262 | 0.3243 | 0.3817 | 0.4283 | 0.1777 | 0.26033 | 0.3233 | 0.374 | 0.419 |
| % viability |  |  | 42.1751 | 52.122 | 64.523 | 75.928 | 85.212 | 35.345 | 51.7905 | 64.324 | 74.4 | 83.36 |
| Ws4/HOP-92 | | 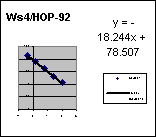 |  |  |  |  |  | ws6/HOP-92 | | 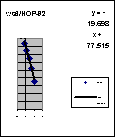 |  |  |
| 2 | 42.18 |  |  |  |  |  |  | 2 | 35.3448 |  |  |  |
| 1.4 | 52.12 |  |  |  |  |  |  | 1.3979 | 51.7905 |  |  |  |
| 0.8 | 64.52 |  |  |  |  |  |  | 0.7959 | 64.3236 |  |  |  |
| 0.19 | 75.93 |  |  |  |  |  |  | 0.1931 | 74.4032 |  |  |  |
| -0.41 | 85.21 |  |  |  |  |  |  | -0.4089 | 83.3554 |  |  |  |
|  |  |  |  |  |  |  |  |  |  |  |  |  |
| IC50= |  |  |  |  |  |  |  | IC50= |  |  |  |  |

| ROBONIK P2000 eia reader | | | |  |  |  |  |  |  |  |  |  |
| --- | --- | --- | --- | --- | --- | --- | --- | --- | --- | --- | --- | --- |
| Wave length: | | 450 nm |  |  |  |  |  |  |  |  |  |  |
| Reference: 630 nm | | | |  |  |  |  |  |  |  |  |  |
|  | **1** | **2** | **3** | **4** | **5** | **6** | **7** | **8** | **9** | **10** | **11** | **12** |
|  |  |  |  |  |  |  |  |  |  |  |  |  |
| A | 0.001 | 0.611 | 0.154 | 0.202 | 0.276 | 0.359 | 0.418 | 0.121 | 0.144 | 0.262 | 0.344 | 0.414 |
| B | 0.001 | 0.603 | 0.161 | 0.208 | 0.285 | 0.364 | 0.441 | 0.104 | 0.132 | 0.259 | 0.392 | 0.428 |
| C | 0.001 | 0.598 | 0.165 | 0.214 | 0.306 | 0.371 | 0.435 | 0.086 | 0.171 | 0.261 | 0.373 | 0.415 |
| mean | 0.001 | 0.604 | 0.16 | 0.208 | 0.289 | 0.365 | 0.431 | 0.1037 | 0.149 | 0.2607 | 0.3697 | 0.419 |
| % viability |  |  | 26.49 | 34.44 | 47.85 | 60.38 | 71.41 | 17.163 | 24.67 | 43.157 | 61.203 | 69.371 |
| Ws5/HOP-92 | | 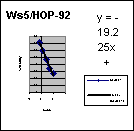 | | | |  |  | STA/HOP-92 | | 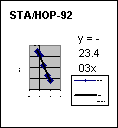 | | |
| 2 | 26.49 |  |  |  |  |  |  | 2 | 17.16 |  |  |  |
| 1.398 | 34.437 |  |  |  |  |  |  | 1.3979 | 24.67 |  |  |  |
| 0.796 | 47.848 |  |  |  |  |  |  | 0.7959 | 43.16 |  |  |  |
| 0.193 | 60.375 |  |  |  |  |  |  | 0.1931 | 61.2 |  |  |  |
| -0.41 | 71.413 |  |  |  |  |  |  | -0.409 | 69.37 |  |  |  |
|  |  |  |  |  |  |  |  |  |  |  |  |  |
| IC50= |  |  |  |  |  |  |  | IC50= |  |  |  |  |
|  |  |  |  |  |  |  |  |  |  |  |  |  |

|  | **Blank** | **CC** | **Sample No. Ws7/HOP-92** | | | | |
| --- | --- | --- | --- | --- | --- | --- | --- |
|  | **1** | **2** | **3** | **4** | **5** | **6** | **7** |
| A | B | C | 100uM | 25uM | 6.3uM | 1.6uM | 0.4uM |
| B | B | C | 100uM | 25uM | 6.3uM | 1.6uM | 0.4uM |
| C | B | C | 100uM | 25uM | 6.3uM | 1.6uM | 0.4uM |
| ROBONIK P2000 Eia reader | | | |  |  |  |  |
| Wave length: 450 nm | | | |  |  |  |  |
| Reference: 630 nm | | | |  |  |  |  |
|  | **1** | **2** | **3** | **4** | **5** | **6** | **7** |
|  |  |  |  |  |  |  |  |
| A | 0.001 | 0.563 | 0.188 | 0.262 | 0.311 | 0.371 | 0.424 |
| B | 0.001 | 0.558 | 0.194 | 0.278 | 0.307 | 0.385 | 0.437 |
| C | 0.001 | 0.549 | 0.181 | 0.262 | 0.326 | 0.363 | 0.441 |
| mean | 0.001 | 0.557 | 0.18767 | 0.2673 | 0.3147 | 0.373 | 0.434 |
| % viability |  |  | 33.7126 | 48.024 | 56.527 | 67.006 | 77.964 |
| Ws7/HOP-92 | |  |  | 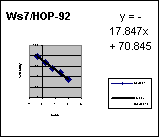 |  |  |  |
| 2 | 33.71 |  |  |  |  |  |  |
| 1.4 | 48.02 |  |  |  |  |  |  |

|  |  |  |  |  |  |  |  |  |  |  |  |  |
| --- | --- | --- | --- | --- | --- | --- | --- | --- | --- | --- | --- | --- |
|  |  |  |  |  |  |  |  |  |  |  |  |  |
|  | **Blank** | **CC** | **Sample No. Ws4/T-47D** | | | | | **Sample No. ws6/T-47D** | | | | |
|  | **1** | **2** | **3** | **4** | **5** | **6** | **7** | **8** | **9** | **10** | **11** | **12** |
| A | B | C | 100uM | 25uM | 6.3uM | 1.6uM | 0.4uM | 100uM | 25uM | 6.3uM | 1.6uM | 0.4uM |
| B | B | C | 100uM | 25uM | 6.3uM | 1.6uM | 0.4uM | 100uM | 25uM | 6.3uM | 1.6uM | 0.4uM |
| C | B | C | 100uM | 25uM | 6.3uM | 1.6uM | 0.4uM | 100uM | 25uM | 6.3uM | 1.6uM | 0.4uM |
| ROBONIK P2000 Eia reader | | | |  |  |  |  |  |  |  |  |  |
| Wave length: 450 nm | | | |  |  |  |  |  |  |  |  |  |
| Reference: 630 nm | | | |  |  |  |  |  |  |  |  |  |
|  | **1** | **2** | **3** | **4** | **5** | **6** | **7** | **8** | **9** | **10** | **11** | **12** |
|  |  |  |  |  |  |  |  |  |  |  |  |  |
| A | 0.001 | 0.549 | 0.192 | 0.246 | 0.311 | 0.364 | 0.425 | 0.228 | 0.284 | 0.342 | 0.392 | 0.428 |
| B | 0.003 | 0.532 | 0.178 | 0.259 | 0.327 | 0.371 | 0.411 | 0.219 | 0.276 | 0.337 | 0.404 | 0.431 |
| C | 0.001 | 0.555 | 0.179 | 0.244 | 0.292 | 0.379 | 0.427 | 0.225 | 0.259 | 0.325 | 0.384 | 0.427 |
| mean | 0.002 | 0.545 | 0.183 | 0.2497 | 0.31 | 0.3713 | 0.421 | 0.224 | 0.273 | 0.3347 | 0.393 | 0.429 |
| % viability |  |  | 33.5575 | 45.782 | 56.846 | 68.093 | 77.2 | 41.076 | 50.0611 | 61.369 | 72.13 | 78.61 |
| Ws4/T-47D | | 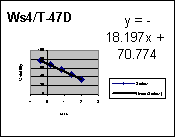 |  |  |  |  |  | ws6/T-47D | | 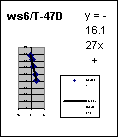 |  |  |
| 2 | 33.56 |  |  |  |  |  |  | 2 | 41.0758 |  |  |  |
| 1.4 | 45.78 |  |  |  |  |  |  | 1.3979 | 50.0611 |  |  |  |
| 0.8 | 56.85 |  |  |  |  |  |  | 0.7959 | 61.3692 |  |  |  |
| 0.19 | 68.09 |  |  |  |  |  |  | 0.1931 | 72.1271 |  |  |  |

|  |  |  |  |  |  |  |  |  | |  |  | |  |  |
| --- | --- | --- | --- | --- | --- | --- | --- | --- | --- | --- | --- | --- | --- | --- |
|  | **Blank** | **CC** | **Sample No. Ws4/MOLT-4** | | | | | **Sample No. ws6/MOLT-4** | | | | | | |
|  | **1** | **2** | **3** | **4** | **5** | **6** | **7** | **8** | | **9** | **10** | | **11** | **12** |
| A | B | C | 100uM | 25uM | 6.3uM | 1.6uM | 0.4uM | 100uM | | 25uM | 6.3uM | | 1.6uM | 0.4uM |
| B | B | C | 100uM | 25uM | 6.3uM | 1.6uM | 0.4uM | 100uM | | 25uM | 6.3uM | | 1.6uM | 0.4uM |
| C | B | C | 100uM | 25uM | 6.3uM | 1.6uM | 0.4uM | 100uM | | 25uM | 6.3uM | | 1.6uM | 0.4uM |
| ROBONIK P2000 Eia reader | | | |  |  |  |  |  | |  |  | |  |  |
| Wave length: 450 nm | | | |  |  |  |  |  | |  |  | |  |  |
| Reference: 630 nm | | | |  |  |  |  |  | |  |  | |  |  |
|  | **1** | **2** | **3** | **4** | **5** | **6** | **7** | **8** | | **9** | **10** | | **11** | **12** |
|  |  |  |  |  |  |  |  |  | |  |  | |  |  |
| A | 0.001 | 0.569 | 0.159 | 0.231 | 0.284 | 0.331 | 0.404 | 0.221 | | 0.267 | 0.323 | | 0.331 | 0.385 |
| B | 0.002 | 0.547 | 0.167 | 0.226 | 0.291 | 0.364 | 0.382 | 0.216 | | 0.265 | 0.325 | | 0.329 | 0.393 |
| C | 0.001 | 0.551 | 0.178 | 0.225 | 0.286 | 0.351 | 0.379 | 0.215 | | 0.269 | 0.328 | | 0.333 | 0.394 |
| mean | 0.001 | 0.556 | 0.168 | 0.2273 | 0.287 | 0.3487 | 0.3883 | 0.2173 | | 0.267 | 0.3253 | | 0.331 | 0.391 |
| % viability |  |  | 30.234 | 40.912 | 51.65 | 62.747 | 69.886 | 39.112 | | 48.0504 | 58.548 | | 59.56 | 70.31 |
| Ws4/MOLT-4 | | 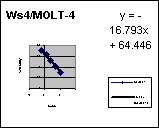 | | | |  |  | ws6/MOLT-4 | | | 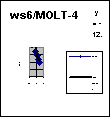 | |  |  |
| 2 | 30.23 |  |  |  |  |  |  | 2 | | 39.1122 |  | |  |  |
| 1.4 | 40.91 |  |  |  |  |  |  | 1.3979 | | 48.0504 |  | |  |  |
| 0.8 | 51.65 |  |  |  |  |  |  | 0.7959 | | 58.5483 |  | |  |  |
| 0.19 | 62.75 |  |  |  |  |  |  | 0.1931 | | 59.5561 |  | |  |  |
| -0.41 | 69.89 |  |  |  |  |  |  | -0.4089 | | 70.3059 |  | |  |  |
|  |  |  |  |  |  |  |  |  | |  |  | |  |  |
| IC50= |  |  |  |  |  |  |  | IC50= | |  |  | |  |  |
|  |  |  |  |  |  |  |  |  | |  |  | |  |  |
|  |  |  |  |  |  |  |  |  | |  |  | |  |  |
|  | **Blank** | **CC** | **Sample No. Ws7/MOLT-4** | | | | | **Sample No. STA/MOLT-4** | | | | | | |
|  | **1** | **2** | **3** | **4** | **5** | **6** | **7** | **8** | | **9** | **10** | | **11** | **12** |
| A | B | C | 100uM | 25uM | 6.3uM | 1.6uM | 0.4uM | 100uM | | 25uM | 6.3uM | | 1.6uM | 0.4uM |
| B | B | C | 100uM | 25uM | 6.3uM | 1.6uM | 0.4uM | 100uM | | 25uM | 6.3uM | | 1.6uM | 0.4uM |
| C | B | C | 100uM | 25uM | 6.3uM | 1.6uM | 0.4uM | 100uM | | 25uM | 6.3uM | | 1.6uM | 0.4uM |
| ROBONIK P2000 Eia reader | | | |  |  |  |  |  | |  |  | |  |  |
| Wave length: 450 nm | | | |  |  |  |  |  | |  |  | |  |  |
| Reference: 630 nm | | | |  |  |  |  |  | |  |  | |  |  |
|  | **1** | **2** | **3** | **4** | **5** | **6** | **7** |  |  |  |  |  |  |  |
|  |  |  |  |  |  |  |  |  |  |  |  |  |  |  |
| A | 0.003 | 0.611 | 0.213 | 0.257 | 0.294 | 0.333 | 0.414 |  |  |  |  |  |  |  |
| B | 0.001 | 0.628 | 0.217 | 0.259 | 0.296 | 0.338 | 0.425 |  |  |  |  |  |  |  |
| C | 0.001 | 0.597 | 0.219 | 0.262 | 0.295 | 0.336 | 0.426 |  |  |  |  |  |  |  |
| mean | 0.002 | 0.612 | 0.21633 | 0.2593 | 0.295 | 0.3357 | 0.4217 |  |  |  |  |  |  |  |
| % viability |  |  | 35.3486 | 42.375 | 48.203 | 54.847 | 68.9 |  |  |  |  |  |  |  |
| Ws7/MOLT-4 | | 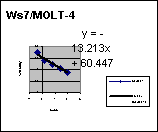 | | | |  |  |  |  | | |  |  |  |
| 2 | 35.35 |  |  |  |  |  |  |  |  |  |  |  |  |  |
| 1.4 | 42.37 |  |  |  |  |  |  |  |  | | |  |  |  |
| 0.8 | 48.2 |  |  |  |  |  |  |  |  | | |  |  |  |
| 0.19 | 54.85 |  |  |  |  |  |  |  |  | | |  |  |  |
| -0.41 | 68.9 |  |  |  |  |  |  |  |  | | |  |  |  |
|  |  |  |  |  |  |  |  |  | |  |  | |  |  |
| IC50= |  |  |  |  |  |  |  | IC50= | |  |  | |  |  |
|  |  |  |  |  |  |  |  |  | |  |  | |  |  |
|  |  |  |  |  |  |  |  |  | |  |  | |  |  |

|  | **Blank** | | | **CC** | | **Sample No. Ws5/MOLT-4** | | | | | | | | **Sample No. STA/MOLT-4** | | | | | | | | | |  |
| --- | --- | --- | --- | --- | --- | --- | --- | --- | --- | --- | --- | --- | --- | --- | --- | --- | --- | --- | --- | --- | --- | --- | --- | --- |
|  | **1** | | | **2** | | **3** | **4** | **5** | | **6** | | **7** | | **8** | | **9** | | **10** | | **11** | | **12** | |  |
| A | B | | | C | | 100ug | 25ug | 6.3ug | | 1.6ug | | 0.4ug | | 100ug | | 25ug | | 6.3ug | | 1.6ug | | 0.4ug | |  |
| B | B | | | C | | 100ug | 25ug | 6.3ug | | 1.6ug | | 0.4ug | | 100ug | | 25ug | | 6.3ug | | 1.6ug | | 0.4ug | |  |
| C | B | | | C | | 100ug | 25ug | 6.3ug | | 1.6ug | | 0.4ug | | 100ug | | 25ug | | 6.3ug | | 1.6ug | | 0.4ug | |  |
| ROBONIK P2000 eia reader | | | | | | |  |  | |  | |  | |  | |  | |  | |  | |  | |  |
| Wave length: | | | | 450 nm | |  |  |  | |  | |  | |  | |  | |  | |  | |  | |  |
| Reference: 630 nm | | | | | | |  |  | |  | |  | |  | |  | |  | |  | |  | |  |
|  | **1** | | | **2** | | **3** | **4** | **5** | | **6** | | **7** | | **8** | | **9** | | **10** | | **11** | | **12** | |  |
|  |  | | |  | |  |  |  | |  | |  | |  | |  | |  | |  | |  | |  |
| A | 0.001 | | | 0.578 | | 0.222 | 0.262 | 0.298 | | 0.339 | | 0.384 | | 0.137 | | 0.195 | | 0.294 | | 0.372 | | 0.425 | |  |
| B | 0.002 | | | 0.581 | | 0.194 | 0.255 | 0.312 | | 0.347 | | 0.389 | | 0.164 | | 0.215 | | 0.306 | | 0.355 | | 0.418 | |  |
| C | 0.001 | | | 0.599 | | 0.208 | 0.259 | 0.319 | | 0.341 | | 0.385 | | 0.129 | | 0.227 | | 0.311 | | 0.364 | | 0.433 | |  |
| mean | 0.0013 | | | 0.586 | | 0.208 | 0.259 | 0.31 | | 0.342 | | 0.386 | | 0.1433 | | 0.212 | | 0.3037 | | 0.3637 | | 0.4253 | |  |
| % viability |  | | |  | | 35.495 | 44.14 | 52.84 | | 58.42 | | 65.87 | | 24.46 | | 36.23 | | 51.82 | | 62.059 | | 72.582 | |  |
| Ws5/MOLT-4 | | | | 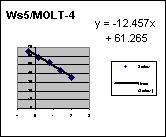 | |  |  |  | |  | |  | | STA/MOLT-4 | | | | 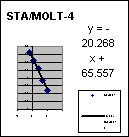 | |  | |  | |  |
| 2 | 35.495 | | |  | |  |  |  | |  | |  | | 2 | | 24.46 | |  | |  | |  | |  |
| 1.398 | 44.141 | | |  | |  |  |  | |  | |  | | 1.3979 | | 36.23 | |  | |  | |  | |  |
| 0.796 | 52.844 | | |  | |  |  |  | |  | |  | | 0.7959 | | 51.82 | |  | |  | |  | |  |
| 0.193 | 58.419 | | |  | |  |  |  | |  | |  | | 0.1931 | | 62.06 | |  | |  | |  | |  |
| -0.41 | 65.87 | | |  | |  |  |  | |  | |  | | -0.409 | | 72.58 | |  | |  | |  | |  |
|  |  | | |  | |  |  |  | |  | |  | |  | |  | |  | |  | |  | |  |
| IC50= |  | | |  | |  |  |  | |  | |  | | IC50= | |  | |  | |  | |  | |  |
|  | | **Blank** | **CC** | | **Sample No. Ws4/WI38** | | | | | | | | | | **Sample No. ws6/WI38** | | | | | | | | | |
|  | | **1** | **2** | | **3** | | **4** | | **5** | | **6** | | **7** | | **8** | | **9** | | **10** | | **11** | | **12** | |
| A | | B | C | | 100uM | | 25uM | | 6.3uM | | 1.6uM | | 0.4uM | | 100uM | | 25uM | | 6.3uM | | 1.6uM | | 0.4uM | |
| B | | B | C | | 100uM | | 25uM | | 6.3uM | | 1.6uM | | 0.4uM | | 100uM | | 25uM | | 6.3uM | | 1.6uM | | 0.4uM | |
| C | | B | C | | 100uM | | 25uM | | 6.3uM | | 1.6uM | | 0.4uM | | 100uM | | 25uM | | 6.3uM | | 1.6uM | | 0.4uM | |
| ROBONIK P2000 Eia reader | | | | | | |  | |  | |  | |  | |  | |  | |  | |  | |  | |
| Wave length: 450 nm | | | | | | |  | |  | |  | |  | |  | |  | |  | |  | |  | |
| Reference: 630 nm | | | | | | |  | |  | |  | |  | |  | |  | |  | |  | |  | |
|  | | **1** | **2** | | **3** | | **4** | | **5** | | **6** | | **7** | | **8** | | **9** | | **10** | | **11** | | **12** | |
|  | |  |  | |  | |  | |  | |  | |  | |  | |  | |  | |  | |  | |
| A | | 0.001 | 0.549 | | 0.264 | | 0.313 | | 0.354 | | 0.408 | | 0.449 | | 0.234 | | 0.287 | | 0.364 | | 0.418 | | 0.465 | |
| B | | 0.002 | 0.551 | | 0.259 | | 0.324 | | 0.359 | | 0.412 | | 0.457 | | 0.225 | | 0.322 | | 0.379 | | 0.405 | | 0.444 | |
| C | | 0.001 | 0.583 | | 0.265 | | 0.309 | | 0.356 | | 0.414 | | 0.455 | | 0.241 | | 0.315 | | 0.357 | | 0.431 | | 0.437 | |
| mean | | 0.001 | 0.561 | | 0.26267 | | 0.3153 | | 0.3563 | | 0.4113 | | 0.4537 | | 0.2333 | | 0.308 | | 0.3667 | | 0.418 | | 0.449 | |
| % viability | |  |  | | 46.8212 | | 56.209 | | 63.518 | | 73.321 | | 80.867 | | 41.592 | | 54.902 | | 65.359 | | 74.51 | | 79.98 | |
| Ws4/WI38 | | | 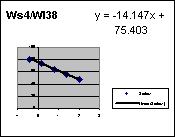 | |  | |  | |  | |  | |  | | ws6/WI38 | | | | 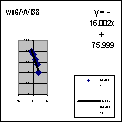 | |  | |  | |
| 2 | | 46.82 |  | |  | |  | |  | |  | |  | | 2 | | 41.5924 | |  | |  | |  | |
| 1.4 | | 56.21 |  | |  | |  | |  | |  | |  | | 1.3979 | | 54.902 | |  | |  | |  | |
| 0.8 | | 63.52 |  | |  | |  | |  | |  | |  | | 0.7959 | | 65.3595 | |  | |  | |  | |
| 0.19 | | 73.32 |  | |  | |  | |  | |  | |  | | 0.1931 | | 74.5098 | |  | |  | |  | |
| -0.41 | | 80.87 |  | |  | |  | |  | |  | |  | | -0.4089 | | 79.9762 | |  | |  | |  | |
|  | |  |  | |  | |  | |  | |  | |  | |  | |  | |  | |  | |  | |
| IC50= | |  |  | |  | |  | |  | |  | |  | | IC50= | |  | |  | |  | |  | |
|  | |  |  | |  | |  | |  | |  | |  | |  | |  | |  | |  | |  | |
|  | |  |  | |  | |  | |  | |  | |  | |  | |  | |  | |  | |  | |
|  | | **Blank** | **CC** | | **Sample No. Ws7/WI38** | | | | | | | | | | **Sample No. Ws5/WI38** | | | | | | | | | |
|  | | **1** | **2** | | **3** | | **4** | | **5** | | **6** | | **7** | | **8** | | **9** | | **10** | | **11** | | **12** | |
| A | | B | C | | 100uM | | 25uM | | 6.3uM | | 1.6uM | | 0.4uM | | 100uM | | 25uM | | 6.3uM | | 1.6uM | | 0.4uM | |
| B | | B | C | | 100uM | | 25uM | | 6.3uM | | 1.6uM | | 0.4uM | | 100uM | | 25uM | | 6.3uM | | 1.6uM | | 0.4uM | |
| C | | B | C | | 100uM | | 25uM | | 6.3uM | | 1.6uM | | 0.4uM | | 100uM | | 25uM | | 6.3uM | | 1.6uM | | 0.4uM | |
| ROBONIK P2000 Eia reader | | | | | | |  | |  | |  | |  | |  | |  | |  | |  | |  | |
| Wave length: 450 nm | | | | | | |  | |  | |  | |  | |  | |  | |  | |  | |  | |
| Reference: 630 nm | | | | | | |  | |  | |  | |  | |  | |  | |  | |  | |  | |
|  | | **1** | **2** | | **3** | | **4** | | **5** | | **6** | | **7** | | **8** | | **9** | | **10** | | **11** | | **12** | |
|  | |  |  | |  | |  | |  | |  | |  | |  | |  | |  | |  | |  | |
| A | | 0.003 | 0.538 | | 0.236 | | 0.287 | | 0.341 | | 0.388 | | 0.436 | | 0.231 | | 0.263 | | 0.322 | | 0.392 | | 0.434 | |
| B | | 0.001 | 0.543 | | 0.233 | | 0.272 | | 0.334 | | 0.373 | | 0.431 | | 0.228 | | 0.265 | | 0.319 | | 0.367 | | 0.447 | |
| C | | 0.001 | 0.529 | | 0.241 | | 0.271 | | 0.336 | | 0.369 | | 0.428 | | 0.219 | | 0.284 | | 0.341 | | 0.381 | | 0.458 | |
| mean | | 0.002 | 0.537 | | 0.23667 | | 0.2767 | | 0.337 | | 0.3767 | | 0.4317 | | 0.226 | | 0.27067 | | 0.3273 | | 0.38 | | 0.446 | |
| % viability | |  |  | | 44.0994 | | 51.553 | | 62.795 | | 70.186 | | 80.435 | | 42.112 | | 50.4348 | | 60.994 | | 70.81 | | 83.17 | |
| Ws7/WI38 | | | 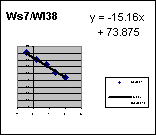 | | | | | | | |  | |  | | Ws5/WI38 | | | | 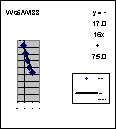   \|  \| \| --- \| | |  | |  | |
| 2 | | 44.1 |  |  |  |  |  |  |  |  |  | |  | | 2 | | 42.1118 | |  | |  | |  | |
| 1.4 | | 51.55 |  |  |  |  |  |  |  |  |  | |  | | 1.3979 | | 50.4348 | |  | |  | |  | |
| 0.8 | | 62.8 |  |  |  |  |  |  |  |  |  | |  | | 0.7959 | | 60.9938 | |  | |  | |  | |
| 0.19 | | 70.19 |  |  |  |  |  |  |  |  |  | |  | | 0.1931 | | 70.8075 | |  | |  | |  | |
| -0.41 | | 80.43 |  |  |  |  |  |  |  |  |  | |  | | -0.4089 | | 83.1677 | |  | |  | |  | |
|  | |  |  |  |  |  |  |  |  |  |  | |  | |  | |  | |  | |  | |  | |
| IC50= | |  |  |  |  |  |  |  |  |  |  | |  | | IC50= | |  | |  | |  | |  | |
|  | |  |  | |  | |  | |  | |  | |  | |  | |  | |  | |  | |  | |
|  | |  |  | |  | |  | |  | |  | |  | |  | |  | |  | |  | |  | |
|  | | **Blank** | **CC** | | **Sample No. STA/WI38** | | | | | | | | | | **Sample No.** | | | | | | | | | |
|  | | **1** | **2** | | **3** | | **4** | | **5** | | **6** | | **7** | | **8** | | **9** | | **10** | | **11** | | **12** | |
| A | | B | C | | 100uM | | 25uM | | 6.3uM | | 1.6uM | | 0.4uM | |  | |  | |  | |  | |  | |
| B | | B | C | | 100uM | | 25uM | | 6.3uM | | 1.6uM | | 0.4uM | |  | |  | |  | |  | |  | |
| C | | B | C | | 100uM | | 25uM | | 6.3uM | | 1.6uM | | 0.4uM | |  | |  | |  | |  | |  | |
| ROBONIK P2000 Eia reader | | | | | | |  | |  | |  | |  | |  | |  | |  | |  | |  | |
| Wave length: 450 nm | | | | | | |  | |  | |  | |  | |  | |  | |  | |  | |  | |
| Reference: 630 nm | | | | | | |  | |  | |  | |  | |  | |  | |  | |  | |  | |
|  | | **1** | **2** | | **3** | | **4** | | **5** | | **6** | | **7** | | **8** | | **9** | | **10** | | **11** | | **12** | |
|  | |  |  | |  | |  | |  | |  | |  | |  | |  | |  | |  | |  | |
| A | | 0.001 | 0.477 | | 0.181 | | 0.226 | | 0.264 | | 0.319 | | 0.388 | |  | |  | |  | |  | |  | |
| B | | 0.003 | 0.483 | | 0.177 | | 0.221 | | 0.271 | | 0.319 | | 0.404 | |  | |  | |  | |  | |  | |
| C | | 0.001 | 0.464 | | 0.175 | | 0.219 | | 0.265 | | 0.3317 | | 0.412 | |  | |  | |  | |  | |  | |
| mean | | 0.002 | 0.475 | | 0.17767 | | 0.222 | | 0.2667 | | 0.3232 | | 0.4013 | | 0 | | 0 | | 0 | | 0 | | 0 | |
| % | |  | ` | | 37.4298 | | 46.77 | | 56.18 | | 68.097 | | 84.551 | | 0 | | 0 | | 0 | | 0 | | 0 | |
| STA/WI38 | |  |  | | 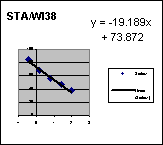 | | | | | | | |  | |  | |  | | 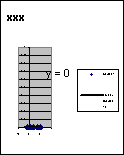 | |  | |  | |
| log conc. | | % viability |  | |  |  |  |  |  |  |  |  |  | | log conc. | | % viability | |  | |  | |  | |
| 2 | | 37.43 |  | |  |  |  |  |  |  |  |  |  | | 2 | | 0 | |  | |  | |  | |
| 1.4 | | 46.77 |  | |  |  |  |  |  |  |  |  |  | | 1.3979 | | 0 | |  | |  | |  | |
| 0.8 | | 56.18 |  | |  |  |  |  |  |  |  |  |  | | 0.7959 | | 0 | |  | |  | |  | |
| 0.19 | | 68.1 |  | |  |  |  |  |  |  |  |  |  | | 0.1931 | | 0 | |  | |  | |  | |
| -0.41 | | 84.55 |  | |  |  |  |  |  |  |  |  |  | | -0.4089 | | 0 | |  | |  | |  | |
|  | |  |  | |  |  |  |  |  |  |  |  |  | |  | |  | |  | |  | |  | |
| IC50= | |  |  | |  |  |  |  |  |  |  |  |  | | IC50= | |  | |  | |  | |  | |
|  | |  |  | |  | |  | |  | |  | |  | |  | |  | |  | |  | |  | |
|  | |  |  | |  | |  | |  | |  | |  | |  | |  | |  | |  | |  | |

|  |  |  |  |  |  |  |  |  |  | |  | |  | |  |
| --- | --- | --- | --- | --- | --- | --- | --- | --- | --- | --- | --- | --- | --- | --- | --- |
|  | **Blank** | **CC** | **Sample No. Ws7/T-47D** | | | | |  |  |  |  |  |  |  |  |
|  | **1** | **2** | **3** | **4** | **5** | **6** | **7** |  |  |  |  |  |  |  |  |
| A | B | C | 100uM | 25uM | 6.3uM | 1.6uM | 0.4uM |  |  |  |  |  |  |  |  |
| B | B | C | 100uM | 25uM | 6.3uM | 1.6uM | 0.4uM |  |  |  |  |  |  |  |  |
| C | B | C | 100uM | 25uM | 6.3uM | 1.6uM | 0.4uM |  |  |  |  |  |  |  |  |
| ROBONIK P2000 Eia reader | | | |  |  |  |  |  |  | |  | |  | |  |
| Wave length: 450 nm | | | |  |  |  |  |  |  | |  | |  | |  |
| Reference: 630 nm | | | |  |  |  |  |  |  | |  | |  | |  |
|  | **1** | **2** | **3** | **4** | **5** | **6** | **7** |  |  |  |  |  |  |  |  |
|  |  |  |  |  |  |  |  |  |  |  |  |  |  |  |  |
| A | 0.001 | 0.575 | 0.185 | 0.256 | 0.308 | 0.345 | 0.388 |  |  |  |  |  |  |  |  |
| B | 0.001 | 0.564 | 0.179 | 0.249 | 0.311 | 0.349 | 0.386 |  |  |  |  |  |  |  |  |
| C | 0.001 | 0.559 | 0.213 | 0.252 | 0.315 | 0.353 | 0.389 |  |  |  |  |  |  |  |  |
| mean | 0.001 | 0.566 | 0.19233 | 0.2523 | 0.3113 | 0.349 | 0.3877 |  |  |  |  |  |  |  |  |
| % viability |  |  | 33.9812 | 44.582 | 55.006 | 61.661 | 68.492 |  |  |  |  |  |  |  |  |
| Ws7/T-47D | | 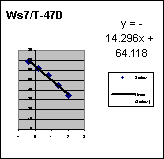 |  |  |  |  |  |  | |  | |  | |  |  |
| log conc. | % viability |  |  |  |  |  |  |  | |  | |  | |  |  |
| 2 | 33.98 |  |  |  |  |  |  |  | |  | |  | |  |  |
| 1.4 | 44.58 |  |  |  |  |  |  |  | |  | |  | |  |  |
| 0.8 | 55.01 |  |  |  |  |  |  |  | |  | |  | |  |  |
| 0.19 | 61.66 |  |  |  |  |  |  |  | |  | |  | |  |  |
| -0.41 | 68.49 |  |  |  |  |  |  |  | |  | |  | |  |  |
|  |  |  |  |  |  |  |  |  |  | |  | |  | |  |

|  | **Blank** | **CC** | **Sample No. Ws5/T47D** | | | | | **Sample No. STA/T47D** | | | | |
| --- | --- | --- | --- | --- | --- | --- | --- | --- | --- | --- | --- | --- |
|  | **1** | **2** | **3** | **4** | **5** | **6** | **7** | **8** | **9** | **10** | **11** | **12** |
| A | B | C | 100uM | 25uM | 6.3uM | 1.6ug | 0.4uM | 100uM | 25uM | 6.3uM | 1.6ug | 0.4uM |
| B | B | C | 100uM | 25uM | 6.3uM | 1.6ug | 0.4uM | 100uM | 25uM | 6.3uM | 1.6ug | 0.4uM |
| C | B | C | 100uM | 25uM | 6.3uM | 1.6ug | 0.4uM | 100uM | 25uM | 6.3uM | 1.6ug | 0.4uM |
| ROBONIK P2000 eia reader | | | |  |  |  |  |  |  |  |  |  |
| Wave length: 450 nm | | | |  |  |  |  |  |  |  |  |  |
| Reference: 630 nm | | | |  |  |  |  |  |  |  |  |  |
|  | **1** | **2** | **3** | **4** | **5** | **6** | **7** | **8** | **9** | **10** | **11** | **12** |
|  |  |  |  |  |  |  |  |  |  |  |  |  |
| A | 0.001 | 0.543 | 0.176 | 0.231 | 0.281 | 0.332 | 0.405 | 0.159 | 0.191 | 0.252 | 0.333 | 0.377 |
| B | 0.003 | 0.528 | 0.163 | 0.222 | 0.277 | 0.328 | 0.393 | 0.167 | 0.182 | 0.246 | 0.316 | 0.365 |
| C | 0.001 | 0.531 | 0.175 | 0.221 | 0.276 | 0.331 | 0.387 | 0.149 | 0.188 | 0.237 | 0.337 | 0.364 |
| mean | 0.0017 | 0.534 | 0.1713 | 0.225 | 0.278 | 0.33 | 0.395 | 0.1583 | 0.187 | 0.245 | 0.3287 | 0.3687 |
| % viability |  |  | 32.085 | 42.07 | 52.06 | 61.86 | 73.97 | 29.65 | 35.02 | 45.88 | 61.548 | 69.039 |
| Ws5/T47D | | 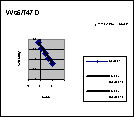 | | | |  |  | STA/T47D | | 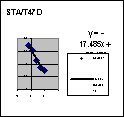 | | |
| 2 | 32.085 |  |  |  |  |  |  | 2 | 29.65 |  |  |  |
| 1.398 | 42.072 |  |  |  |  |  |  | 1.3979 | 35.02 |  |  |  |
| 0.796 | 52.06 |  |  |  |  |  |  | 0.7959 | 45.88 |  |  |  |
| 0.193 | 61.86 |  |  |  |  |  |  | 0.1931 | 61.55 |  |  |  |
| -0.41 | 73.97 |  |  |  |  |  |  | -0.409 | 69.04 |  |  |  |
|  |  |  |  |  |  |  |  |  |  |  |  |  |
| IC50= |  |  |  |  |  |  |  | IC50= |  |  |  |  |
|  |  |  |  |  |  |  |  |  |  |  |  |  |
|  |  |  |  |  |  |  |  |  |  |  |  |  |
|  | **Blank** | **CC** | **Sample No. Ws5/MDA-MB-468** | | | | | **Sample No. STA/MDA-MB-468** | | | | |
|  | **1** | **2** | **3** | **4** | **5** | **6** | **7** | **8** | **9** | **10** | **11** | **12** |
| A | B | C | 100uM | 25uM | 6.3uM | 1.6ug | 0.4uM | 100uM | 25uM | 6.3uM | 1.6ug | 0.4uM |
| B | B | C | 100uM | 25uM | 6.3uM | 1.6ug | 0.4uM | 100uM | 25uM | 6.3uM | 1.6ug | 0.4uM |
| C | B | C | 100uM | 25uM | 6.3uM | 1.6ug | 0.4uM | 100uM | 25uM | 6.3uM | 1.6ug | 0.4uM |

**EGFR inhibitor screening**

**Researcher** : Dr.Mohamed Hawas email: [mohhawwas80@gmail.com](mailto:mohhawwas80@gmail.com)  **mob.** 01151345909

Assay : EGFR inhibitor screening [mohamedhassan.pharm.ast@azhr.edu.eg](mailto:mohamedhassan.pharm.ast@azhr.edu.eg)

**Samples** : 04 compounds

Cell lines : ---

**Ref**. : ----

Date : 03-12-2023

**Reader** : Tecan Spark

Kit used : ---

**Solvent** : DMSO

**Lab Report**

| **ser** | **Compound** | | | **EGFR** | **SD±** |
| --- | --- | --- | --- | --- | --- |
|  | **code** | **MW** | **conc.**  **uM** | **IC50**  **uM** |  |
| 1 | **WS4** | **470** | --- | **0.156** | 0.006 |
| 2 | **WS5** | **515** | --- | **0.055** | 0.002 |
| 3 | **WS6** | **401** | --- | **0.64** | 0.023 |
| 4 | **WS7** | **415** | --- | **0.194** | 0.007 |
| 5 | **Erlotinib** | **393.44** | --- | **0.06** | 0.002 |

**Detailed results**

| **EGFR** |  |  |  |  |  |  |  |  |  |  |  |  |
| --- | --- | --- | --- | --- | --- | --- | --- | --- | --- | --- | --- | --- |
| code | IC50 | conc | log | %inh | T2 | T1 | ∆T | RFU2 | RFU1 | ∆RFU | slope | K.Activity |
| Ws4 |  | 100 | 2 | 93.7 | 30 | 0 | 30 | 6.26 | 0 | 6.26 | 3.333 | 7.512 |
| 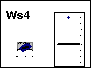 |  | 10 | 1 | 86.9 | 30 | 0 | 30 | 13.09 | 0 | 13.09 | 3.333 | 15.708 |
|  |  | 1 | 0 | 72.7 | 30 | 0 | 30 | 27.33 | 0 | 27.33 | 3.333 | 32.796 |
|  |  | 0.1 | -1 | 40.6 | 30 | 0 | 30 | 59.41 | 0 | 59.41 | 3.333 | 71.292 |
|  |  | 0.01 | -2 | 27.8 | 30 | 0 | 30 | 72.15 | 0 | 72.15 | 3.333 | 86.58 |
| EC |  |  |  | 0 | 30 | 0 | 30 | 100 | 0 | 100 | 3.333 | 120 |
|  |  |  |  |  |  |  |  |  |  |  |  |  |
| code | IC50 | conc | log | %inh | T2 | T1 | ∆T | RFU2 | RFU1 | ∆RFU | slope | K.Activity |
| Ws5 |  | 100 | 2 | 94.2 | 30 | 0 | 30 | 5.84 | 0 | 5.84 | 3.333 | 7.008 |
| 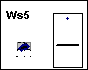 |  | 10 | 1 | 88.1 | 30 | 0 | 30 | 11.92 | 0 | 11.92 | 3.333 | 14.304 |
|  |  | 1 | 0 | 75.8 | 30 | 0 | 30 | 24.19 | 0 | 24.19 | 3.333 | 29.028 |
|  |  | 0.1 | -1 | 50.6 | 30 | 0 | 30 | 49.35 | 0 | 49.35 | 3.333 | 59.22 |
|  |  | 0.01 | -2 | 37 | 30 | 0 | 30 | 62.99 | 0 | 62.99 | 3.333 | 75.588 |
| EC |  |  |  | 0 | 30 | 0 | 30 | 100 | 0 | 100 | 3.333 | 120 |
|  |  |  |  |  |  |  |  |  |  |  |  |  |
| code | IC50 | conc | log | %inh | T2 | T1 | ∆T | RFU2 | RFU1 | ∆RFU | slope | K.Activity |
| Ws6 |  | 100 | 2 | 92.1 | 30 | 0 | 30 | 7.86 | 0 | 7.86 | 3.333 | 9.432 |
| 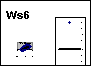 |  | 10 | 1 | 80.6 | 30 | 0 | 30 | 19.41 | 0 | 19.41 | 3.333 | 23.292 |
|  |  | 1 | 0 | 61.8 | 30 | 0 | 30 | 38.22 | 0 | 38.22 | 3.333 | 45.864 |
|  |  | 0.1 | -1 | 26 | 30 | 0 | 30 | 74.03 | 0 | 74.03 | 3.333 | 88.836 |
|  |  | 0.01 | -2 | 10.6 | 30 | 0 | 30 | 89.39 | 0 | 89.39 | 3.333 | 107.27 |
| EC |  |  |  | 0 | 30 | 0 | 30 | 100 | 0 | 100 | 3.333 | 120 |
|  |  |  |  |  |  |  |  |  |  |  |  |  |
| code | IC50 | conc | log | %inh | T2 | T1 | ∆T | RFU2 | RFU1 | ∆RFU | slope | K.Activity |
| Ws7 |  | 100 | 2 | 94.5 | 30 | 0 | 30 | 5.52 | 0 | 5.52 | 3.333 | 6.624 |
| 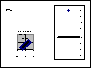 |  | 10 | 1 | 86.3 | 30 | 0 | 30 | 13.71 | 0 | 13.71 | 3.333 | 16.452 |
|  |  | 1 | 0 | 71.4 | 30 | 0 | 30 | 28.55 | 0 | 28.55 | 3.333 | 34.26 |
|  |  | 0.1 | -1 | 44.2 | 30 | 0 | 30 | 55.79 | 0 | 55.79 | 3.333 | 66.948 |
|  |  | 0.01 | -2 | 21 | 30 | 0 | 30 | 79.04 | 0 | 79.04 | 3.333 | 94.848 |
| EC |  |  |  | 0 | 30 | 0 | 30 | 100 | 0 | 100 | 3.333 | 120 |
|  |  |  |  |  |  |  |  |  |  |  |  |  |
| code | IC50 | conc | log | %inh | T2 | T1 | ∆T | RFU2 | RFU1 | ∆RFU | slope | K.Activity |
| Erlotenib |  | 100 | 2 | 95.7 | 30 | 0 | 30 | 4.29 | 0 | 4.29 | 3.333 | 5.148 |
| 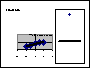 |  | 10 | 1 | 88.9 | 30 | 0 | 30 | 11.08 | 0 | 11.08 | 3.333 | 13.296 |
|  |  | 1 | 0 | 76.4 | 30 | 0 | 30 | 23.55 | 0 | 23.55 | 3.333 | 28.26 |
|  |  | 0.1 | -1 | 51.8 | 30 | 0 | 30 | 48.19 | 0 | 48.19 | 3.333 | 57.828 |
|  |  | 0.01 | -2 | 34.5 | 30 | 0 | 30 | 65.46 | 0 | 65.46 | 3.333 | 78.552 |
| EC |  |  |  | 0 | 30 | 0 | 30 | 100 | 0 | 100 | 3.333 | 120 |
|  |  |  |  |  |  |  |  |  |  |  |  |  |

| 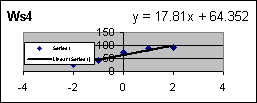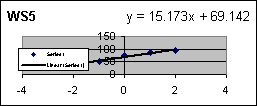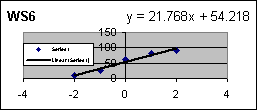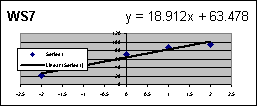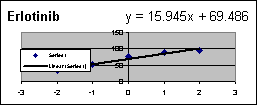 |  |  |  |
| --- | --- | --- | --- |
|  |  |  |  |
|  |  |  |  |
|  |  |  |  |
|  |  |  |  |
|  |  |  |  |
|  |  |  |  |
|  |  |  |  |
|  |  |  |  |
|  |  |  |  |
|  |  |  |  |
|  |  |  |  |
|  |  |  |  |

**B-Raf (V600E)Kinase Assay**

**Researcher** : Dr.Mohamed Hawas email: [mohhawwas80@gmail.com](mailto:mohhawwas80@gmail.com)  **mob.** 01151345909

Assay : B-Raf (V600E)Kinase Assay [mohamedhassan.pharm.ast@azhr.edu.eg](mailto:mohamedhassan.pharm.ast@azhr.edu.eg)

Samples : 04 samples .

Reference : ---

Cell lines : ---

Kit used : ---

Solvent : DMSO

Assay samples :

**Lab Report**

| ser | compound | | | **B-Raf(V600E)** | SD  ± |
| --- | --- | --- | --- | --- | --- |
|  | code | M.W | conc | IC50  uM |  |
| **1** | **WS4** | **470** | --- | **0.249** | 0.01 |
| **2** | **WS5** | **515** | --- | **0.068** | 0.003 |
| **3** | **WS6** | **401** | --- | **0.410** | 0.016 |
| **4** | **WS7** | **415** | --- | **0.194** | 0.008 |
| ******* | **Vemurafenib** | 489.92 | --- | **0.035** | 0.001 |

**Detailed results:**

| **B-raf (V600E)** | | | | | | | | | | | |  |
| --- | --- | --- | --- | --- | --- | --- | --- | --- | --- | --- | --- | --- |
| code | IC50 | conc | log | %inh | T2 | T1 | ∆T | RFU2 | RFU1 | ∆RFU | slope | K.Activity |
| WS4 |  | 100 | 2 | 93 | 30 | 0 | 30 | 7.29 | 0 | 7.29 | 3.3333 | 8.74809 |
|  |  | 10 | 1 | 84 | 30 | 0 | 30 | 16.03 | 0 | 16.03 | 3.3333 | 19.2362 |
| 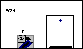 |  | 1 | 0 | 64 | 30 | 0 | 30 | 35.88 | 0 | 35.88 | 3.3333 | 43.0564 |
|  |  | 0.1 | -1 | 41 | 30 | 0 | 30 | 59.37 | 0 | 59.37 | 3.3333 | 71.2447 |
|  |  | 0.01 | -2 | 23 | 30 | 0 | 30 | 76.55 | 0 | 76.55 | 3.3333 | 91.8609 |
| EC |  |  |  | 0 | 30 | 0 | 30 | 100 | 0 | 100 | 3.3333333 | 120 |
|  |  |  |  |  |  |  |  |  |  |  |  |  |
| code | IC50 | conc | log | %inh | T2 | T1 | ∆T | RFU2 | RFU1 | ∆RFU | slope | K.Activity |
| WS5 |  | 100 | 2 | 94 | 30 | 0 | 30 | 5.72 | 0 | 5.72 | 3.3333 | 6.86407 |
| 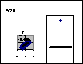 |  | 10 | 1 | 88 | 30 | 0 | 30 | 12.41 | 0 | 12.41 | 3.3333 | 14.8921 |
|  |  | 1 | 0 | 71 | 30 | 0 | 30 | 29.13 | 0 | 29.13 | 3.3333 | 34.9563 |
|  |  | 0.1 | -1 | 52 | 30 | 0 | 30 | 47.68 | 0 | 47.68 | 3.3333 | 57.2166 |
|  |  | 0.01 | -2 | 35 | 30 | 0 | 30 | 65.33 | 0 | 65.33 | 3.3333 | 78.3968 |
| EC |  |  |  | 0 | 30 | 0 | 30 | 100 | 0 | 100 | 3.3333333 | 120 |
|  |  |  |  |  |  |  |  |  |  |  |  |  |
| code | IC50 | conc | log | %inh | T2 | T1 | ∆T | RFU2 | RFU1 | ∆RFU | slope | K.Activity |
| WS6 |  | 100 | 2 | 91 | 30 | 0 | 30 | 9.03 | 0 | 9.03 | 3.3333 | 10.8361 |
|  |  | 10 | 1 | 81 | 30 | 0 | 30 | 19.35 | 0 | 19.35 | 3.3333 | 23.2202 |
| 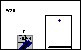 |  | 1 | 0 | 63 | 30 | 0 | 30 | 37.22 | 0 | 37.22 | 3.3333 | 44.6644 |
|  |  | 0.1 | -1 | 35 | 30 | 0 | 30 | 64.93 | 0 | 64.93 | 3.3333 | 77.9168 |
|  |  | 0.01 | -2 | 18 | 30 | 0 | 30 | 82.31 | 0 | 82.31 | 3.3333 | 98.773 |
| EC |  |  |  | 0 | 30 | 0 | 30 | 100 | 0 | 100 | 3.3333333 | 120 |
|  |  |  |  |  |  |  |  |  |  |  |  |  |
| code | IC50 | conc | log | %inh | T2 | T1 | ∆T | RFU2 | RFU1 | ∆RFU | slope | K.Activity |
| WS7 |  | 100 | 2 | 94 | 30 | 0 | 30 | 6.12 | 0 | 6.12 | 3.3333 | 7.34407 |
| 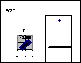 |  | 10 | 1 | 86 | 30 | 0 | 30 | 14.39 | 0 | 14.39 | 3.3333 | 17.2682 |
|  |  | 1 | 0 | 70 | 30 | 0 | 30 | 29.59 | 0 | 29.59 | 3.3333 | 35.5084 |
|  |  | 0.1 | -1 | 42 | 30 | 0 | 30 | 58.24 | 0 | 58.24 | 3.3333 | 69.8887 |
|  |  | 0.01 | -2 | 24 | 30 | 0 | 30 | 76.19 | 0 | 76.19 | 3.3333 | 91.4289 |
| EC |  |  |  | 0 | 30 | 0 | 30 | 100 | 0 | 100 | 3.3333333 | 120 |
|  |  |  |  |  |  |  |  |  |  |  |  |  |
| code | IC50 | conc | log | %inh | T2 | T1 | ∆T | RFU2 | RFU1 | ∆RFU | slope | K.Activity |
| Vemurafenib |  | 100 | 2 | 95 | 30 | 0 | 30 | 5.41 | 0 | 5.41 | 3.3333 | 6.49206 |
| 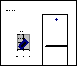 |  | 10 | 1 | 88 | 30 | 0 | 30 | 11.63 | 0 | 11.63 | 3.3333 | 13.9561 |
|  |  | 1 | 0 | 75 | 30 | 0 | 30 | 25.02 | 0 | 25.02 | 3.3333 | 30.0243 |
|  |  | 0.1 | -1 | 61 | 30 | 0 | 30 | 38.69 | 0 | 38.69 | 3.3333 | 46.4285 |
|  |  | 0.01 | -2 | 36 | 30 | 0 | 30 | 64.43 | 0 | 64.43 | 3.3333 | 77.3168 |
| EC |  |  |  | 0 | 30 | 0 | 30 | 100 | 0 | 100 | 3.3333333 | 120 |
|  |  |  |  |  |  |  |  |  |  |  |  |  |

| 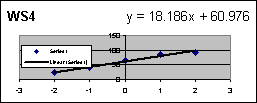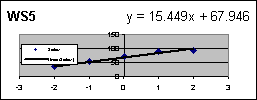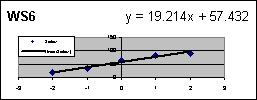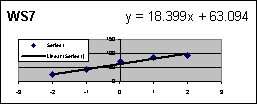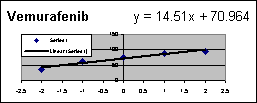 |  |  |  |
| --- | --- | --- | --- |
|  |  |  |  |
|  |  |  |  |
|  |  |  |  |
|  |  |  |  |
|  |  |  |  |
|  |  |  |  |

**Cell Cycle Analysis**

**Researcher** : Dr.Mohamed Hawas email: [mohhawwas80@gmail.com](mailto:mohhawwas80@gmail.com) mob. 01151345909

Assay : Cell Cycle Analysis [mohamedhassan.pharm.ast@azhr.edu.eg](mailto:mohamedhassan.pharm.ast@azhr.edu.eg)

Samples : 02 samples

cell line : ---

Ref. : ---

Date : 00-00-2022

Reader : BD FACSCalibur

Kit used : ab139418_Propidium Iodide Flow Cytometry Kit/BD

Solvent : DMSO

Assay samples : Cell culture

**Lab Report**

| **ser** | **Sample** | | **DNA content** | | | |  |
| --- | --- | --- | --- | --- | --- | --- | --- |
|  | **code** | **IC50**  **uM** | **%G0-G1** | **%S** | **%G2/M** | **Comment** |  |
| 1 | **Ws5/HOP92** | --- | 54.11 | 31.29 | 14.6 | cell growth arrest@ G1 |  |
| 2 | **Cont. HOP92** | --- | 48.26 | 35.66 | 16.08 | --- |  |

| **s** | **code** | **conc** | **Apoptosis** | | | **Necrosis** |
| --- | --- | --- | --- | --- | --- | --- |
|  |  |  | Total | Early | Late |  |
| 1 | **Ws5/HOP92** | --- | **37.59** | **22.91** | **11.28** | **3.4** |
| 2 | **Cont. HOP92** | --- | **2.14** | **0.69** | **0.15** | **1.3** |

**Detailed results**


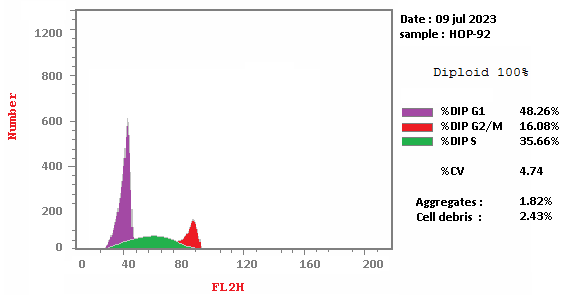


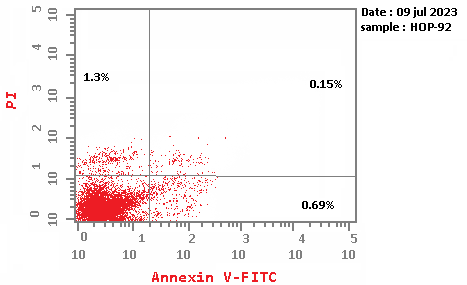


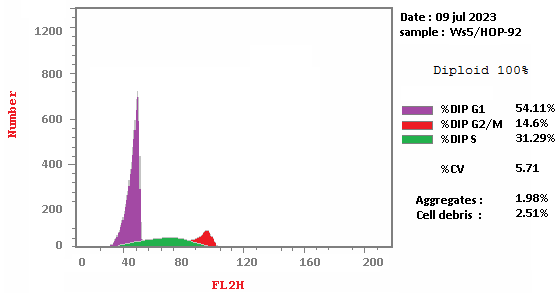


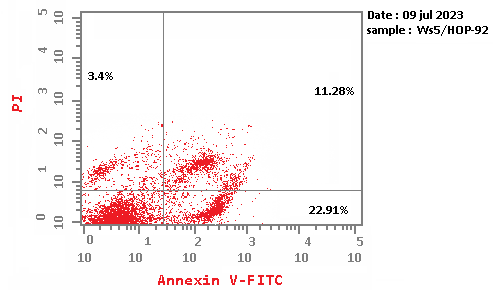


**Researcher** : Dr.Mohamed Hawas email: [mohhawwas80@gmail.com](mailto:mohhawwas80@gmail.com) mob. 01151345909

Assay : Cell Cycle Analysis [mohamedhassan.pharm.ast@azhr.edu.eg](mailto:mohamedhassan.pharm.ast@azhr.edu.eg)

**Assay** : RT-PCR

**Samples** : 02 Samples

**Cell** **lines** : ----

**Ref**. : ----

**Date** : 12-06-2023

**Reader** : Rotorgene RT- PCR system

Kit used : Qiagen RNA extraction/BioRad syber green PCR MMX

**Solvent** : DMSO

**Assay samples** : Cell Lysate

**Lab Report**

| **Ser** | **Sample** | | | **RT-PCR**  **Fold Change** | | | **SD** |
| --- | --- | --- | --- | --- | --- | --- | --- |
|  | **code** | **cells** | **IC50** | **FLD** | | |  |
|  |  |  |  | **Casp3** | **Casp9** | **Bcl2** |  |
| **1** | **Ws5/HOP-92** | **---** | **---** | **4.716** | **3.082** | **0.299** |  |
| **2** | **Staurosporine/HOP-92** | **---** | **---** | **6.906** | **5.514** | **0.326** |  |
| **3** | **Cont.HOP-92** | **---** | **---** | **1** | **1** | **1** |  |

Detailed results


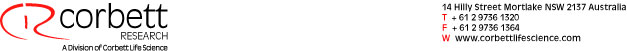


**Quantitation Report**

Experiment Information

| Run Name | Run 2023-07-09 (1) |
| --- | --- |
| Run Start | 2023-07-09 01:46:17 PM |
| Run Finish | 2023-07-09 05:15:04 PM |
| Operator | ERA |
| Notes | --- |
| Run On Software Version | Rotor-Gene 1.7.87 |
| Run Signature | The Run Signature is valid. |
| Gain Green | 10. |
| Gain Yellow | 9.33 |

Quantitation data

| 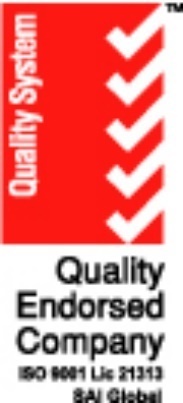 | This report generated by Rotor-Gene 6000 Series Software 1.7 (Build 87) Copyright 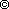2000-2006 Corbett Research, a Division of Corbett Life Science. All rights reserved. ISO 9001:2000 (Reg. No. QEC21313)  Primers  primers  Casp3 : F 5’- GGAAGCGAATCAATGGACTCTGG -3’,  Casp3 : R 5'- GCATCGACATCTGTACCAGACC -3'.  Casp9 : F 5’- GTTTGAGGACCTTCGACCAGCT -3’,  Casp9 : R 5'- CAACGTACCAGGAGCCACTCTT -3'.  Bcl2 : F 5’- ATCGCCCTGTGGATGACTGAGT -3’,  Bcl2 : R 5'- GCCAGGAGAAATCAAACAGAGGC -3'.  GAPDH : F 5’- GTCTCCTCTGACTTCAACAGCG-3’  GAPDH : R 5’- ACCACCCTGTTGCTGTAGCCAA-3’ |
| --- | --- |

|  |  |  |  |  |  |  |  |  |  |  |
| --- | --- | --- | --- | --- | --- | --- | --- | --- | --- | --- |
| **Sample** | | | **Gene Expression** | | | | | | | |
|  |  |  |  |  |  |  |  |  |  |  |
|  |  |  | Control cells | | | Test cells | | | | FLD |
|  |  |  |  |  |  |  |  |  |  |  |
| **Ser** | **code** | **Conc** | **GAPDH** | **Casp3** | ΔCTC | **GAPDH** | **Casp3** | ΔCTE | ΔΔ CT | 2^ ΔΔCT |
|  |  |  | HC | TC | TC-HC | HE | TE | TE-HE | ΔCTE-ΔCTC | E=1.869 |
| **1** | **Ws5/HOP-92** |  | **22.06** | **34.18** | 12.12 | **21.93** | **31.57** | 9.64 | **-2.48** | 4.7162 |
| **2** | **Staurosporine/HOP-92** |  | **22.06** | **34.18** | 12.12 | **22.25** | **31.28** | 9.03 | **-3.09** | 6.9067 |
| **3** | **Cont.HOP-92** |  | **22.06** | **34.18** | 12.12 | **22.06** | **34.18** | 12.12 | **0** | 1 |
|  |  |  |  |  |  |  |  |  |  |  |
| **Ser** | **code** | **Conc** | **GAPDH** | **Casp9** | ΔCTC | **GAPDH** | **Casp9** | ΔCTE | ΔΔ CT | 2^ ΔΔCT |
|  |  |  | HC | TC | TC-HC | HE | TE | TE-HE | ΔCTE-ΔCTC | E=1.869 |
| **1** | **Ws5/HOP-92** |  | **22.06** | **32.69** | 10.63 | **21.93** | **30.76** | 8.83 | **-1.8** | 3.0825 |
| **2** | **Staurosporine/HOP-92** |  | **22.06** | **32.69** | 10.63 | **22.25** | **30.15** | 7.9 | **-2.73** | 5.5143 |
| **3** | **Cont.HOP-92** |  | **22.06** | **32.69** | 10.63 | **22.06** | **32.69** | 10.63 | **0** | 1 |
|  |  |  |  |  |  |  |  |  |  |  |
| **Ser** | **code** | **Conc** | **GAPDH** | **bcl2** | ΔCTC | **GAPDH** | **bcl2** | ΔCTE | ΔΔ CT | 2^ ΔΔCT |
|  |  |  | HC | TC | TC-HC | HE | TE | TE-HE | ΔCTE-ΔCTC | E=1.869 |
| **1** | **Ws5/HOP-92** |  | **22.06** | **26.81** | 4.75 | **21.93** | **28.61** | 6.68 | **1.93** | 0.2991 |
| **2** | **Staurosporine/HOP-92** |  | **22.06** | **26.81** | 4.75 | **22.25** | **28.79** | 6.54 | **1.79** | 0.3265 |
| **3** | **Cont.HOP-92** |  | **22.06** | **26.81** | 4.75 | **22.06** | **26.81** | 4.75 | **0** | 1 |
|  |  |  |  |  |  |  |  |  |  |  |
